# Cost-effectiveness of interventions for HIV/AIDS, malaria, syphilis, and tuberculosis in 128 countries: a meta-regression analysis

**DOI:** 10.1016/S2214-109X(24)00181-5

**Published:** 2024-06-12

**Authors:** Fiona Silke, Lauren Earl, Johnathan Hsu, Mark M Janko, Jonah Joffe, Aishe Memetova, Danielle Michael, Peng Zheng, Aleksandr Aravkin, Christopher J L Murray, Marcia R Weaver

**Affiliations:** a Institute for Health Metrics and Evaluation, University of Washington, Seattle, WA, USA; b Duke Global Health Institute, Duke University, Durham, NC, USA; c Department of Biostatistics, Epidemiology, and Informatics, Perelman School of Medicine, University of Pennsylvania, Philadelphia, PA, USA; d Shirland Consulting, Seattle, WA, USA; e Department of Health Metric Sciences, School of Medicine, University of Washington, Seattle, WA, USA; f Department of Applied Mathematics, University of Washington, Seattle, WA, USA

## Abstract

**Background:**

Cost-effectiveness analyses have been conducted for many interventions for HIV/AIDS, malaria, syphilis, and tuberculosis, but they have not been conducted for all interventions that are currently recommended in all countries. To support national decision makers in the effective allocation of resources, we conducted a meta-regression analysis of published incremental cost-effectiveness ratios (ICERs) for interventions for these causes, and predicted ICERs for 14 recommended interventions for Global Fund-eligible countries.

**Methods:**

In the meta-regression analysis, we used data from the Tufts University Center for the Evaluation of Value and Risk in Health (Boston, MA, USA) Cost-Effectiveness Registries (the CEA Registry beginning in 1976 and the Global Health CEA registry beginning in 1995) up to Jan 1, 2018. To create analysis files, we standardised and mapped the data, extracted additional data from published articles, and added variables from the Global Burden of Diseases, Injuries, and Risk Factors Study (GBD). Then we selected ratios for interventions with a minimum of two published articles and three published ICERs that mapped to one of five GBD causes (HIV/AIDS, malaria, syphilis, drug-susceptible tuberculosis, or multi-drug resistant tuberculosis), and to a GBD country; reported a currency year during or after 1990; and for which the comparator intervention was defined as no intervention, standard of care, or placebo. Our meta-regression analysis used all available data on 25 eligible interventions, and quantified the association between ICERs and factors at country level and intervention level. We used a five-stage statistical model that was developed to synthesise evidence on cost-effectiveness analyses, and we adapted it for smaller sample sizes by grouping interventions by cause and type (ie, prevention, diagnostics, and treatment). Using the meta-regression parameters we predicted country-specific median ICERs, IQRs, and 95% uncertainty intervals in 2019 US$ per disability-adjusted life-year (DALY) for 14 currently recommended interventions. We report ICERs in league tables with gross domestic product (GDP) per capita and country-specific thresholds.

**Findings:**

The sample for the analysis was 1273 ratios from 144 articles, of which we included 612 ICERs from 106 articles in our meta-regression analysis. We predicted ICERs for antiretroviral therapy for prevention for two age groups and pregnant women, pre-exposure prophylaxis against HIV for two risk groups, four malaria prevention interventions, antenatal syphilis screening, two tuberculosis prevention interventions, the Xpert tuberculosis test, and chemotherapy for drug-sensitive tuberculosis. At the country level, ranking of interventions and number of interventions with a predicted median ICER below the country-specific threshold varied greatly. For instance, median ICERs for six of 14 interventions were below the country-specific threshold in Sudan, whereas 12 of 14 were below the country-specific threshold in Peru. Antenatal syphilis screening had the lowest median ICER among all 14 interventions in 81 (63%) of 128 countries, ranging from $3 (IQR 2–4) per DALY averted in Equatorial Guinea to $3473 (2244–5222) in Ukraine. Pre-exposure prophylaxis for HIV/AIDS for men who have sex with men had the highest median ICER among all interventions in 116 (91%) countries, ranging from $2326 (1077–4567) per DALY averted in Lesotho to $53 559 (23 841–108 534) in Maldives.

**Interpretation:**

Country-specific league tables highlight the interventions that offer better value per DALY averted, and can support decision making at a country level that is more tailored to available resources than GDP per capita and country-specific thresholds. Meta-regression is a promising method to synthesise cost-effectiveness analysis results and transfer them across settings.

**Funding:**

Bill & Melinda Gates Foundation.

## Introduction

Globally, mortality due to HIV/AIDS^1^ and tuberculosis^2^ decreased between 2010 and 2019, and mortality due to malaria decreased between 2000 and 2017,^3^, ^4^ reflecting the success of national, bilateral, and multilateral programmes that prevent, diagnose, and treat these diseases and their funding.^5^ To continue this progress, target 3.3 of the UN Sustainable Development Goals includes ending the epidemics of HIV/AIDS, tuberculosis, and malaria by 2030,^6^ and in 2022 the Global Fund to Fight AIDS, Tuberculosis and Malaria raised US$15·7 billion towards the $18 billion sought for the Seventh Replenishment to save an estimated 20 million lives.^7^ Interventions for HIV/AIDS, tuberculosis, and malaria change over time with improvements in health practices and medical technology, as reflected in updates to national and WHO guidelines, 8, 9, 10, 11, 12 and cost-effectiveness analyses should also be updated. Cost-effectiveness analyses of the most recent interventions would help countries to prioritise interventions and complement the Global Fund's investment case.^13^

**Research in context**

**Evidence before this study**

To support prioritisation, the Disease Control Priority researchers summarised evidence from cost-effectiveness analyses from low-income and middle-income countries from 2000 to 2013 including interventions for HIV/AIDS, tuberculosis, and malaria in a single league table. In a retrospective analysis, WHO researchers produced their Choosing Interventions that are Cost-Effective estimates for the eastern sub-Saharan Africa and southeast Asia WHO regions from 2000 to 2010 for HIV/AIDS, tuberculosis, and malaria interventions. Country-specific evidence is not available for all recommended interventions to prevent, diagnose, and treat HIV/AIDS, tuberculosis, malaria, and syphilis. Meta-regression methods can be used to transfer estimates from cost-effectiveness analyses from one country to another by quantifying the association between incremental cost-effectiveness ratios (ICERs), which are the results of cost-effectiveness analyses, and factors at the country level and intervention level. We searched the Econlit and PubMed databases for peer-reviewed publications between Jan 1, 1974, and Feb 15, 2024. For Econlit, we used the terms [cost-effectiveness analysis] AND (meta-analysis OR meta-regression) AND (HIV OR AIDS OR tuberculosis OR syphilis OR malaria). For PubMed we conducted two searches, first using the meta-analysis filter for article type, and then searching with the term “meta-regression”. We used the following MeSH terms in both PubMed searches: [cost-effectiveness analysis] AND (HIV OR AIDS OR tuberculosis OR latent tuberculosis OR tuberculosis, multidrug-resistant OR syphilis OR syphilis, congenital OR malaria).

**Added value of this study**

To our knowledge, no previous meta-regression analyses have been conducted of cost-effectiveness analyses of interventions for HIV/AIDS, malaria, syphilis, and tuberculosis. We conducted meta-regression analyses of all published ICERs in the cost-effectiveness registries for 25 interventions for HIV/AIDS, malaria, syphilis, and tuberculosis, including evidence from high-income countries as well as low-income and middle-income countries. We produced the first country-specific league tables that rank ICERs from lowest (best value) to highest for 14 currently recommended interventions for 128 countries that are eligible for Global Fund support for at least one of these causes. We report the league tables with ICERs and uncertainty intervals for the estimates, and with two potential cost-effectiveness thresholds, which are measures of affordability.

**Implications of all the available evidence**

The league tables can support national decision makers to prioritise interventions even among those that are currently recommended and assess their affordability. Rankings of interventions by median ICER differ substantially across countries. Importantly, the league tables rank the allocation of funds without resorting to thresholds. Funding could be allocated to the intervention with the lowest ICER first, and intervention added according to their rank and available funds.

Although cost-effectiveness analyses have been conducted for many interventions for HIV/AIDS, tuberculosis, and malaria, they have not been conducted for all interventions in updated guidelines in all countries. Without such analyses, it is difficult for governments to prioritise interventions within the context of their national economy and epidemiology. Results of cost-effectiveness analyses are reported as incremental cost-effectiveness ratios (ICERs), which is the ratio of the additional cost per improvement in health. A lower ICER is indictive of an intervention with better value. Two research groups have summarised ICERs by WHO region^14^ and reported a league table with evidence from low-income and middle-income countries,^15^ but country-specific evidence is not available.

Our goal is to support national decision makers in effectively allocating resources from the Seventh Replenishment by synthesising all available information in published cost-effectiveness analyses that share a common health outcome, thereby enabling the comparison and prioritisation of interventions. In the absence of country-specific ICERs for all currently recommended interventions, researchers have developed methods to transfer estimates from one country to another.^16^, ^17^, ^18^, ^19^ Meta-regression analysis can be used to transfer estimates by quantifying the association between ICERs and factors at the country, intervention, and methods level.

We conducted meta-regression analyses of all published ICERs for 25 interventions for HIV/AIDS, malaria, syphilis, and tuberculosis using evidence from high-income countries as well as low-income and middle-income countries. Then, we predicted incremental cost per disability-adjusted life-year (DALY) averted for 14 currently recommended interventions that are eligible for Global Fund support^20^ for each of the 128 countries that are eligible for support for at least one of these causes.

## Methods

### Study design and data sources

**Figure 1:**
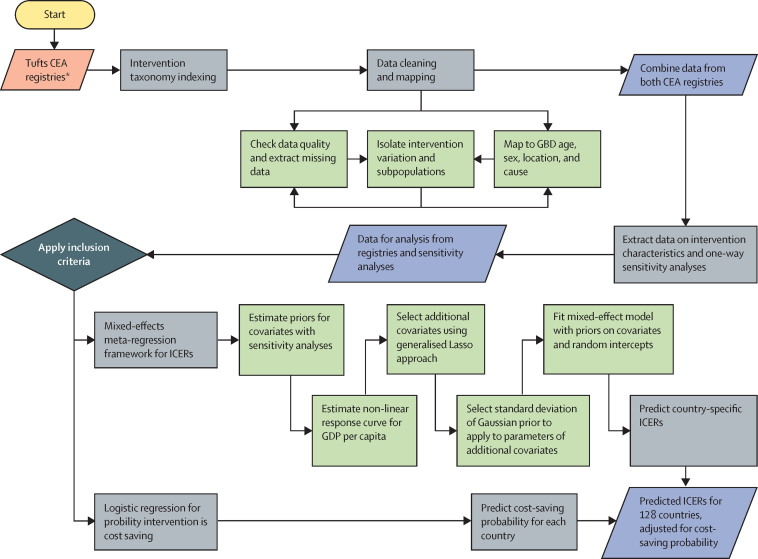
Overview of processes in the meta-regression analysis The red parallelogram is input data, the blue parallelograms are output data, grey rectangles indicate processes, green rectangles indicate a sub-component or component process, and the dark grey diamond indicates a decision. CEA=cost-effectiveness analysis. GBD=Global Burden of Disease. GDP=gross domestic product. ICER=incremental cost-effectiveness ratio. *The two registries are the CEA registry and the Global Health CEA registry.

Briefly, in this meta-regression analysis, we used published results on cost-effectiveness analyses for all interventions that met our inclusion criteria and all countries to conduct a meta-regression analysis that explains the variation in ICERs across countries. Several processes were required to create the analysis file before the selection criteria could be applied and the meta-regression analysis performed (figure 1). We used the parameters estimated in the meta-regression analysis to predict ICERs for a subset of interventions and 128 countries that are eligible for Global Fund support. This study complies with the Guidelines for Accurate and Transparent Health Estimates Reporting (GATHER) statement (appendix pp 4–5),^21^ and since it is an analysis of secondary data, we did not require ethical approval.

Our data sources were two registries created and maintained by Tufts University Center for the Evaluation of Value and Risk in Health (Boston, MA, USA). The Cost-Effectiveness Analysis (CEA) Registry^22^ reports articles on the cost per quality-adjusted life-year (QALY) gained beginning in 1976, and the Global Health CEA Registry^23^ reports articles on the cost per DALY averted beginning in 1995. We restricted data to publications up to Jan 1, 2018. The registries only contain peer-reviewed articles in the English language and research with health outcomes measured in QALYs or DALYs. Cost-effectiveness analyses with measures such as HIV cases averted or tuberculosis cases cured are excluded. Further information on the registries is published elsewhere.^24^, ^25^

### Data standardisation, mapping, extraction, and grouping

We performed tasks (ie, data standardisation, mapping, extraction, and grouping) on the registry data files to create an analysis file. We developed a health intervention taxonomy that categorised similar interventions together for each cause (detailed information is in the appendix [pp 51–54]). When registry entries were missing for key variables, such as age and sex of the target population, intervention and comparator descriptions, study location, and the disease, injury, or risk targeted by the intervention, we extracted these data from the published articles (appendix pp 55–58). Each ratio was mapped to Global Burden of Diseases, Injuries, and Risk Factors Study (GBD) 2019^26^ variables for age group, sex, location, and cause. These variables were used to obtain GBD 2019 results for DALYs per person, prevalence per person, and gross domestic product (GDP) per capita for each ratio and those GBD results were merged with the registry data files.

In previous research,^18^, ^19^ we found that ICERs were associated with cost and efficacy variables that are not in the registry data files. In this research, we distinguished the costs of traded goods, such as vaccines and drugs that are valued at relatively uniform international prices, from local goods with prices that differ by location.^27^ For the current analysis, we extracted data on the cost of traded goods and efficacy of the intervention from the published articles, and assumed that differences in local costs were measured by GDP per capita. The study currency and currency-year were used to convert all ICERs and cost of traded goods to 2019 US$. The efficacy of diagnostic interventions is reported as the sensitivity and specificity of the test. In previous research, we also found that the registries do not extract all published ratios.^18^, ^19^ For the current analysis, we extracted additional ratios and associated variables for one-way sensitivity analyses of the cost and efficacy variables. A one-way sensitivity analysis involves altering a single variable in the cost-effectiveness analysis to assess its effect on the ratio.

Ratios were also grouped by type (ie, preventive, diagnostics, and treatment), such as tuberculosis prevention. These groupings created a sample of interventions with the same cost and efficacy variables that could be meta-regressed together. Although a diagnostic test must be combined with treatment to avert DALYs, just as treatment must be combined with diagnosis, we defined a diagnostic intervention as a comparison of diagnostic tests when the treatments are the same, and a treatment intervention as a comparison of treatments when the diagnostic methods are the same.

We included all ratios in the registries that met the following five inclusion criteria: ratios that were categorised to an intervention with a minimum of two published articles and three published ratios; mapped to one of five GBD causes: HIV/AIDS, malaria, syphilis, drug-susceptible tuberculosis, or multi-drug resistant (MDR) tuberculosis; mapped to a GBD country, as opposed to a multi-country region or hypothetical location; reported a currency year during or after 1990, the earliest year with GBD 2019 estimates; and that had a comparator intervention that was no intervention, standard of care, or placebo, as opposed to being described as “other”. From the identified ratios, we excluded seven interventions in the HIV prevention group because the interventions were not defined consistently across articles, and the cost and efficacy variables were not the same across articles. The one remaining HIV prevention intervention was pre-exposure prophylaxis (PREP), so this cause-type group is hereafter referred to as HIV/AIDS PREP. Additional exclusions are described in the appendix (pp 66–68). Applying the criteria was an iterative process of running code and visual inspection to check the results. Code was run by JJ, DM, and FS. Visual inspection was done by LE, AM, DM, FS, and MRW.

### Covariates

We used location-specific variables and intervention characteristics to explain true variation across ratios. The two location-specific variables were: GDP per capita and disease burden measured in DALYs per person or disease prevalence per person. For antenatal syphilis screening and intermittent preventive treatment for malaria for pregnant women, we used the burden for infants (ie, age 0–11 months), which is the population that benefits. For HIV/AIDS screening for pregnant women and lifelong antiretroviral therapy (ART; hereafter referred to as option B+), we used the combined burden for both women aged 10–49 years and infants. GDP per capita measures differences in local costs for each country, including the labour cost of the intervention and savings in treatment costs. The burden variable measures the number needed to treat in prevention and diagnostic models. In the treatment models, in which everyone who is diagnosed benefits from treatment, the burden variable might measure economies of scale or the benefits of practice on the quality of care.^28^ Two intervention characteristic covariates are cost of traded goods and efficacy. The methods variables used in previous research^18^, ^19^ were not used in this analysis because they did not necessarily vary across ratios. For example, one of the methods variables indicates whether health outcomes are measured in QALYs or DALYs; all malaria prevention and syphilis diagnostics ratios are reported as cost per DALY averted.

### Statistical analysis

We used a Bayesian mixed-effects meta-regression framework,^29^ and conducted the analysis in five stages, as previously reported,^18^, ^19^ with adaptations for the unique features of the data. The rationale for the Bayesian perspective is that we used informative priors from the crosswalks in stage 1, and shape information in stage 2, which we consider to be a prior. We systematically compared estimates for each cause-type group from multiple model specifications and selected the model with the best fit. The criteria for the best fit were the R^2^, root mean-squared error, and ratio of the upper to lower bound of the 95% uncertainty interval (UI) of the predictions. The five stages are briefly described below, and more information is in the appendix on the five stages (pp 59–64) and adaptations (pp 65–66).

In stage 1, we conducted a crosswalk analysis using one-way sensitivity analyses to estimate the association between the ICER and each intervention characteristic. The crosswalk calculated how the ICER changes when the characteristic changes, and the resulting estimated coefficient serves as the prior. In stage 2, we estimated the association between log-ICER and log-GDP per capita. This stage uses a spline to model log-ICERs as a non-linear function of log-GDP per capita, a spline ensemble to make the model less sensitive to model specification, and a robust statistical approach to detecting outliers. In stage 3, we use the generalised Lasso approach for linear mixed-effects models to select potential covariates controlling for the non-linear response curve estimated in stage 2. In stage 4, we use 10-fold cross-validation to select the standard deviation of a Gaussian prior to apply to all covariates other than those analysed in the crosswalk analysis (stage 1). In stage 5, we include covariates that were selected in stage 3, along with the non-linear response curve, and estimate a mixed-effects model with random intercepts for each article.

Ratios were excluded from the analysis sample during the statistical analysis. Ratios for sensitivity analyses were excluded when the model with the crosswalk analysis (stage 1) did not have the best fit, or the cost or efficacy covariate was not selected in the final model (stage 3). Ratios were excluded (ie, trimmed) as a consequence of detecting outliers (stage 2). Cost-saving results and ratios with missing values for covariates were also excluded.

We used meta-regression parameters to predict country-specific ICERs for 14 interventions recommended in recent WHO guidelines, with dates ranging from 2020 to 2023,^8^, ^9^, ^10^, ^11^, ^12^ using the GBD 2019 values for GDP per capita and burden,^26^ and intervention cost and efficacy when selected. We did not predict ICERs for 12 interventions, because they are no longer recommended (eight interventions), or they were not selected as covariates in the models with the best fit (four interventions; appendix p 104). For example, guidelines currently recommend initiating ART for everyone who is diagnosed with HIV/AIDS, so we did not predict ICERs for interventions that restricted access to ART, such as ART with initiation criteria. We predicted ICERs for ART for prevention for two different age groups by using the appropriate values of burden and cost for each age group. The cost of all required doses is based on four sources of the cost per dose: (1) 2022 price in the Global Fund Procurement Mechanism for HIV/AIDS and malaria drugs and other commodities, (2) 2022 price in the 2022 Stop TB Partnership Global Drug Facility catalogues for tuberculosis drugs and diagnostic tests, (3) 2020 price in the WHO Global Vaccine Market Report for the BCG vaccine against tuberculosis, and (4) current price in the UNICEF Supply Catalogue for the rapid syphilis test (appendix pp 71–73). Efficacy parameters are from WHO guidelines when available, Cochrane reviews, or the literature (appendix pp 74–76).

ICERs and covariates from two or more published cost-effectiveness analyses are never the same, which is one source of uncertainty in the estimates. UIs for covariates, such as GDP per capita and burden of disease, are also reflected in the predictions. We report predicted ICERs for each intervention as the median, IQR, and the 95% UI to show this uncertainty.

In some cost-effectiveness analyses, the intervention decreases cost and improves health outcomes, termed a cost-saving result. We did not calculate ICERs for these results because a ranking among cost-saving interventions is not meaningful.^30^ A cost-saving intervention is always preferred to one that increases costs and improves health outcomes, termed an ICER. We estimated the probability that an intervention has a cost-saving result as opposed to an ICER (appendix p 77). We estimated this probability for three interventions that had at least two cost-saving results and complete data for the covariates: ART for prevention, intermittent preventive treatment for malaria for infants, and bed nets. For these three interventions, the reported ICER was the product of the predicted ICERs and 1 –the predicted probability of being cost saving.

An ICER is just one element of a decision to adopt an intervention, where the cost per DALY averted is a unit cost with a common denominator that supports comparisons. Another element is affordability.^31^ We report league tables in the context of two potential thresholds as measures of affordability: GDP per capita, and a country-specific opportunity cost of health-care expenditures. We used estimates from Pichon-Riviere et al^32^ of the opportunity cost based on health-care expenditures per capita, life-expectancy, and the percentage increase in health-care expenditure per capita per change in life expectancy; the percentage increase differs across World Bank income groups.^32^ We recalculated the opportunity cost with 2019 data from GBD 2019,^33^ and the Financing Global Health series,^34^ and report the country-specific opportunity cost.

We did the meta-regression analysis using an open-source mixed-effects package, LimeTr.^29^ We predicted ICERs for 128 countries, with adjustments for cost-saving results as described earlier, using Python version 3.0. We did the logistic regression analysis using the open-source software lme4 in R (version 4.0.5)**.**

### Role of the funding source

The funder of the study had no role in the study design, data collection, data analysis, data interpretation, or writing of the report.

## Results

**Figure 2:**
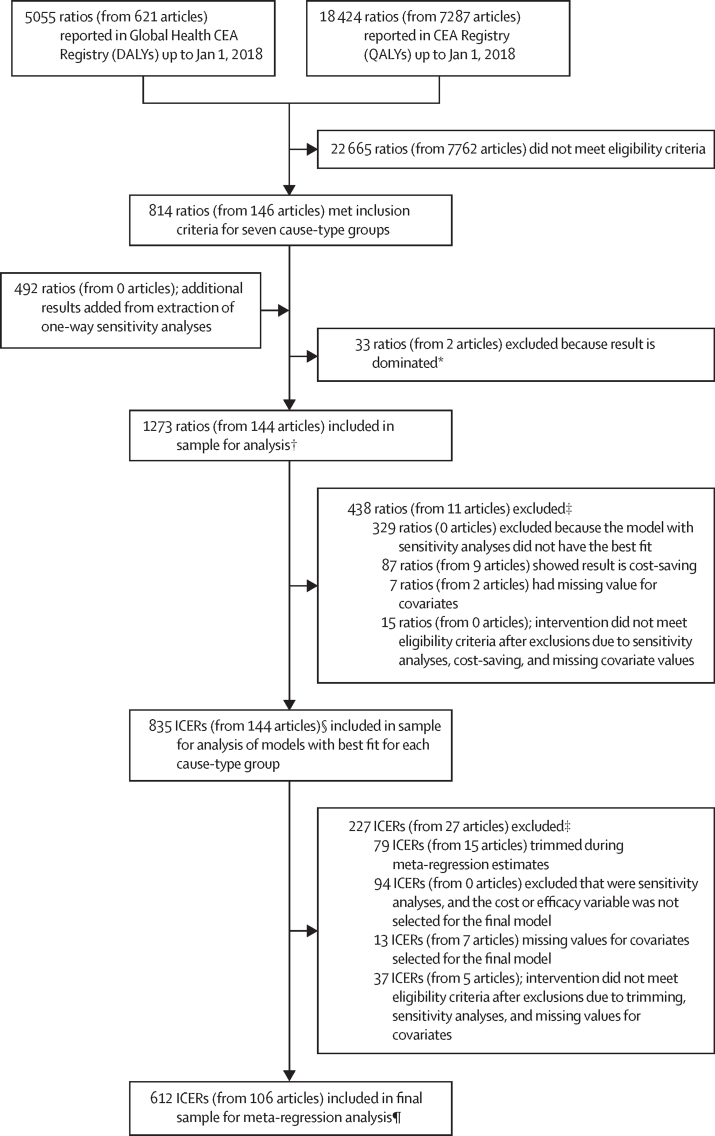
Flow diagram for meta-regression analysis based on data from cost-effectiveness analyses registries CEA=cost-effectiveness analysis. DALY=disability-adjusted life-year. ICER=incremental cost-effectiveness ratio. QALY=quality-adjusted life-year. *Dominated means that the intervention was costlier and led to worse outcomes than the chosen reference ratio. †Descriptive statistics for each cause-type group are in table 1. ‡Exclusions for each cause-type group are in the appendix (p 68). §In the summary of articles presented in the appendix (pp 6–40), the number of articles is 136 rather than 144; this is because we tracked articles by cause-type group, and ratios from two cause-type groups were extracted from six articles, and ratios from three cause-type groups were extracted from one article. ¶Descriptive statistics for each cause-type group are in the appendix (pp 69–70).

Beginning with 5055 ratios reported in the Global Health CEA Registry and 18 424 in the CEA Registry, 814 ratios from 146 articles met our inclusion criteria (figure 2). From these articles, we extracted 492 one-way sensitivity analyses for intervention cost and efficacy. For each article, the one-way sensitivity analyses were compared with a single reference ratio chosen from the same article. We excluded 33 results from two articles for interventions that were more costly and had worse health outcomes than the comparator, termed dominated results. After adding the sensitivity analyses and removing the dominated results, the analysis sample comprised 1273 ratios from 144 articles (appendix pp 6–40), of which 1186 were ICERs and 87 were cost-saving results.

Among the seven cause-type groups, HIV/AIDS ART and syphilis diagnostics were the only ones with ratios from six of seven GBD super-regions (table). The sample for malaria prevention covered three super-regions because many countries are certified malaria-free. No ratios for syphilis diagnostics were published about countries in the high-income super-region, perhaps because the intervention is broadly adopted in those countries.

**Table:** Descriptive statistics on the analysis sample across seven cause-type groups, including 1273 ratios from 144 articles

			**HIV/AIDS ART**	**HIV/AIDS PREP**	**Malaria prevention**	**Syphilis diagnostics**	**Drug-susceptible tuberculosis treatment**	**Tuberculosis prevention**	**Tuberculosis diagnostics**
Total ratio sample size			612	86	148	222	10	62	133
	Sensitivity analysis counted in total		289	19	21	133	2	7	33
	Cost-saving ratio counted in total		29	1	20	1	2	30	4
Study characteristics									
	GBD super-region								
		Central Europe, eastern Europe, and Central Asia	16 (3%)	2 (2%)	0	0	0	0	0
		High income	453 (74%)	31 (36%)	0	3 (1%)	0	47 (76%)	39 (29%)
		Latin America and Caribbean	4 (1%)	6 (7%)	0	25 (11%)	0	4 (6%)	23 (17%)
		North Africa and Middle East	0	1 (1%)	1 (1%)	4 (2%)	0	0	0
		South Asia	9 (1%)	0	6 (4%)	2 (1%)	1 (10%)	0	16 (12%)
		Southeast Asia, east Asia, and Oceania	19 (3%)	0	0	11 (5%)	5 (50%)	0	1 (1%)
		Sub-Saharan Africa	111 (18%)	46 (53%)	141 (95%)	177 (80%)	4 (40%)	11 (18%)	54 (41%)
	Year published								
		1990–94	0	0	0	0	0	0	0
		1995–99	0	0	0	0	0	2 (3%)	0
		2000–04	55 (9%)	0	2 (1%)	1 (<1%)	4 (40%)	40 (65%)	0
		2005–09	84 (14%)	8 (9%)	11 (7%)	12 (5%)	3 (30%)	16 (26%)	32 (24%)
		2010–14	433 (71%)	66 (77%)	31 (21%)	175 (79%)	0	4 (6%)	82 (62%)
		2015–17	40 (7%)	12 (14%)	104 (70%)	33 (15%)	3 (30%)	0	19 (14%)
Methods									
	Health outcome measure								
		QALYs	505 (83%)	35 (41%)	0	0	0	54 (87%)	43 (32%)
		DALYs	107 (17%)	51 (59%)	148 (100%)	222 (100%)	10 (100%)	8 (13%)	90 (68%)
	Cost discount rate								
		<3%	32 (5%)	1 (1%)	4 (3%)	209 (94%)	0	2 (3%)	2 (2%)
		3%	381 (62%)	84 (98%)	142 (96%)	13 (6%)	10 (100%)	59 (95%)	131 (98%)
		>3%	199 (33%)	1 (1%)	2 (1%)	0	0	1 (2%)	0
	Health outcome discount rate								
		<3%	31 (5%)	2 (2%)	10 (7%)	0	0	2 (3%)	1 (1%)
		3%	382 (62%)	84 (98%)	138 (93%)	222 (100%)	10 (100%)	59 (95%)	132 (99%)
		>3%	199 (33%)	0	0	0	0	1 (2%)	0
	Perspective								
		Societal or limited societal	129 (21%)	0	105 (71%)	10 (5%)	3 (30%)	49 (79%)	10 (8%)
		Health-care payer or sector	477 (78%)	84 (98%)	43 (29%)	211 (95%)	7 (70%)	13 (21%)	123 (92%)
		Missing	6 (1%)	2 (2%)	0	1 (<1%)	0	0	0
	Time horizon								
		Lifetime	231 (38%)	5 (6%)	33 (22%)	44 (20%)	4 (40%)	47 (76%)	84 (63%)
		Shorter than lifetime	381 (62%)	81 (93%)	115 (78%)	178 (80%)	6 (60%)	15 (24%)	49 (37%)
Type of comparator									
		Placebo	15 (2%)	0	0	0	0	0	0
		Standard care	279 (46%)	49 (57%)	34 (23%)	7 (3%)	3 (30%)	2 (3%)	83 (62%)
		None	318 (52%)	37 (43%)	114 (78%)	215 (97%)	7 (70%)	60 (97%)	50 (38%)
Intervention characteristics									
Cost of intervention									
	Cost of drug per person per year (ie, ART) or per episode, US$		$9003 (515–15 498)	..	..	..	$93 (81–155)	..	..
		Cost of drug per year of protection, US$	..	$298 (189–7949)	$4 (1–6)	..	..	$68 (7–157)	..
		Cost per test, US$	..	..	..	$1 (1–1)	..	..	$20 (10–38)
	Efficacy		80 (70–90)	68 (68–70)	74 (54–91)	..	60 (21–85)	70 (70–75)	..
	Sensitivity		..	..	..	99 (99–99)	..	..	80 (73–89)
	Specificity		..	..	..	86 (83–83)	..	..	99 (98–99)

The 25 interventions were analysed in the meta-regression and are grouped into one of seven cause-type groups. Data are median (IQR), n, or n (%), with percentages calculated using the total ratio sample size as the denominator. The total of 504 sensitivity analyses includes 11 for HIV/AIDS ART and one for malaria prevention that were in the Tufts registries; all others were extracted from published articles. ART=antiretroviral therapy. DALY=disability-adjusted life-year. GBD=Global Burden of Diseases, Injuries, and Risk Factor Study. PREP=pre-exposure prophylaxis. QALY=quality-adjusted life-year.

The sample for the selected meta-regression models comprised 612 ICERs from 106 articles (figure 2). Three key exclusions made were (1) cost-saving results, (2) sensitivity analyses when the model with the crosswalk analysis did not have the best fit, or the cost or efficacy covariates were not selected, (3) and trimming of outliers (appendix p 68). The final samples for the meta-regression analyses were 258 ICERs from 57 articles for HIV/AIDS ART, 80 ICERs from nine articles for HIV/AIDS PREP, 74 ICERs from ten articles for malaria prevention, 111 ICERs from four articles for syphilis diagnostics, eight ICERs from five articles for drug-susceptible tuberculosis treatment, 20 ICERs from eight articles for tuberculosis prevention, and 61 ICERs from 13 articles for tuberculosis diagnostics. Descriptive statistics of this sample (appendix pp 69–70) are similar to those of the analysis sample (table). The sample sizes for the logistic regression analyses were 240 ratios for HIV/AIDS ART and 29 for malaria prevention (appendix pp 85, 93).

Results of our meta-regression are presented in full in the appendix (pp 80–103). In summary, among the modelling specifications, the crosswalk model had the best fit for tuberculosis diagnostics, and sensitivity analyses were included in models with the best fit for three cause-type groups: HIV/ART PREP, tuberculosis prevention, and drug-susceptible tuberculosis treatment. The burden variable was selected in five of seven groups (exceptions were malaria prevention and tuberculosis diagnostics), and the ICER was always negatively associated with it. The cost variable was selected in three groups: HIV/AIDS ART, HIV/AIDS PREP, and tuberculosis diagnostics. Efficacy was selected in four of five groups (with HIV/AIDS ART being the exception), test sensitivity was selected for the syphilis diagnostics model, and both sensitivity and specificity were selected for the tuberculosis diagnostics model.

**Figure 3:**
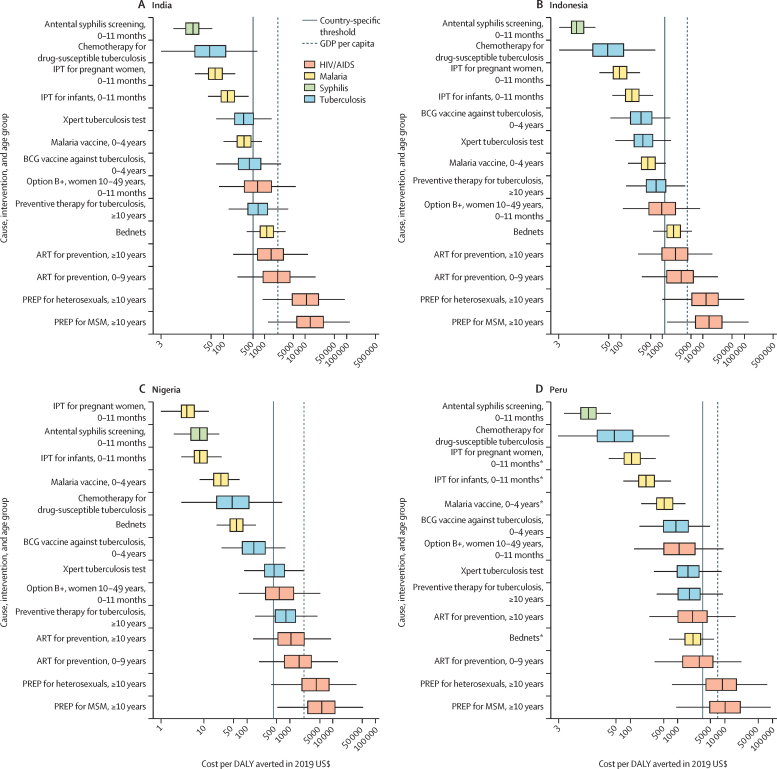

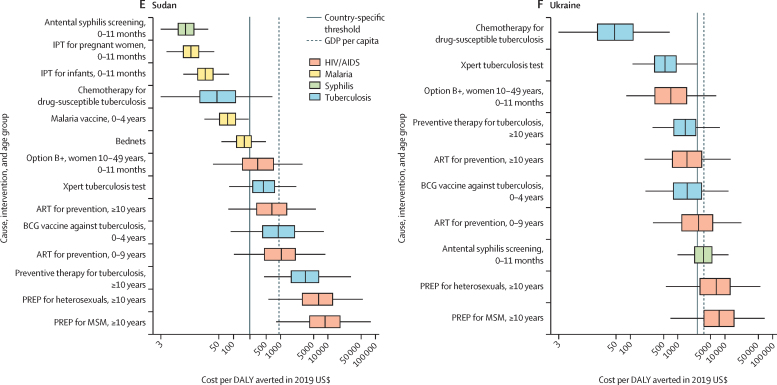
League tables of interventions to avert the burden of HIV/AIDS, malaria, syphilis, and tuberculosis ranked by median incremental cost-effectiveness ratio in India (A), Indonesia (B), Nigeria (C), Peru (D), Sudan (E), and Ukraine (F), in 2019 US$ Box plots show median estimates and IQRs, with whiskers indicating 95% uncertainty intervals. This figure presents the league tables for the country in each GBD super-region (excluding the high-income super-region) that has the highest sum of DALYs from HIV/AIDS, malaria, and tuberculosis. Note that x-axis scales vary between plots. In panel F, no malaria-based interventions are presented because Ukraine is certified malaria-free. For IPT for pregnant women, 0–11 months, the infants aged 0–11 months are the beneficiaries of the intervention. Interventions without an age range are applicable for all age groups. All interventions are eligible for Global Fund support, unless otherwise indicated. The ICERs might be lower-bound estimates when a country is not eligible for support for that cause. ART=antiretroviral therapy. DALY=disability-adjusted life-year. GBD=Global Burden of Diseases, Injuries, and Risk Factor Study. GDP=gross domestic product. ICER=incremental cost-effectiveness ratio. IPT=intermittent preventive treatment for malaria. MSM=men who have sex with men. Option B+=HIV/AIDS screening for pregnant women and lifelong ART. PREP=pre-exposure prophylaxis. *Interventions not eligible for Global Fund support.

In figure 3, we present league tables for the country with the largest sum of DALYs from HIV/AIDS, malaria, and tuberculosis for each of the six GBD super-regions where countries receive Global Fund support: India in south Asia; Indonesia in southeast Asia, east Asia, and Oceania; Nigeria in sub-Saharan Africa; Peru in Latin America and the Caribbean; Sudan in north Africa and the Middle East; and Ukraine in central Europe, eastern Europe, and central Asia. These league tables show differences in the ranking of interventions from the lowest ICER (best value) to the highest (worst value). The league tables also show the potential role of thresholds (ie, country-specific threshold and GDP per capita) in supporting the selection of interventions. The estimated country-specific thresholds range from 0·09-times GDP per capita in the Syria to 1·74 times GDP per capita in the territory of Tuvalu. Country-specific league tables for the 128 countries analysed are in the appendix (pp 105–233).

For India (figure 3A), antenatal syphilis screening has the lowest median ICER followed by chemotherapy for drug-susceptible tuberculosis, intermittent preventive treatment for malaria for pregnant women, and intermittent preventive treatment for malaria for infants. The ranking of these four interventions is the same in Indonesia and Peru (figure 3B, C). For India, the IQRs about the median ICER for the Xpert tuberculosis test, malaria vaccine, and BCG vaccine overlap, and their median ICERs are also below India's country-specific threshold. The median ICERs for five other interventions are below India's GDP per capita: Option B+, preventive therapy for tuberculosis, bed nets, and ART for prevention for individuals aged 10 years or older and aged 0–9 years. India's country-specific threshold is less than 0·3 times GDP per capita, similar to Indonesia, Nigeria, and Sudan.

Among tuberculosis interventions in Indonesia (figure 3B), the median ICER for the BCG vaccine is lower than for the Xpert tuberculosis test. By contrast with India, the median ICER for the BCG vaccine is lower than for the malaria vaccine, and the median ICER for preventive therapy for tuberculosis is below the country-specific threshold.

In Nigeria (figure 3C), intermittent preventive treatment for malaria for pregnant women has the lowest median ICER, and ICERs for all four malaria interventions are below the country-specific threshold. Among tuberculosis interventions, the median ICERs for chemotherapy for drug-susceptible tuberculosis and BCG vaccine are below the country-specific threshold, but the Xpert tuberculosis test is not.

In Peru, the country-specific threshold is 0·47 times GDP per capita (figure 3D). The median ICERs of all malaria and tuberculosis interventions, and Option B+ and ART for prevention are below the country-specific threshold.

In Sudan (figure 3E), the median ICERs for intermittent preventive treatment for malaria for pregnant women and for infants are lower than chemotherapy for drug-susceptible tuberculosis. The median ICERs for only six interventions are below the country-specific threshold. The median ICERs for ART for prevention for individuals aged 0–9 years and preventive therapy for tuberculosis are above GDP per capita.

In Ukraine (figure 3F), chemotherapy for drug-susceptible tuberculosis has the lowest median ICER, followed by Xpert tuberculosis test, Option B+, and preventive therapy for tuberculosis. Ukraine is certified malaria-free, so no ICERs are predicted for malaria interventions. The median ICERs for six interventions are below the country-specific threshold, which is 0·71 times GDP per capita. Using GDP per capita as the threshold, ART for prevention for individuals aged 0–9 years and antenatal syphilis screening would also be supported.

**Figure 4:**
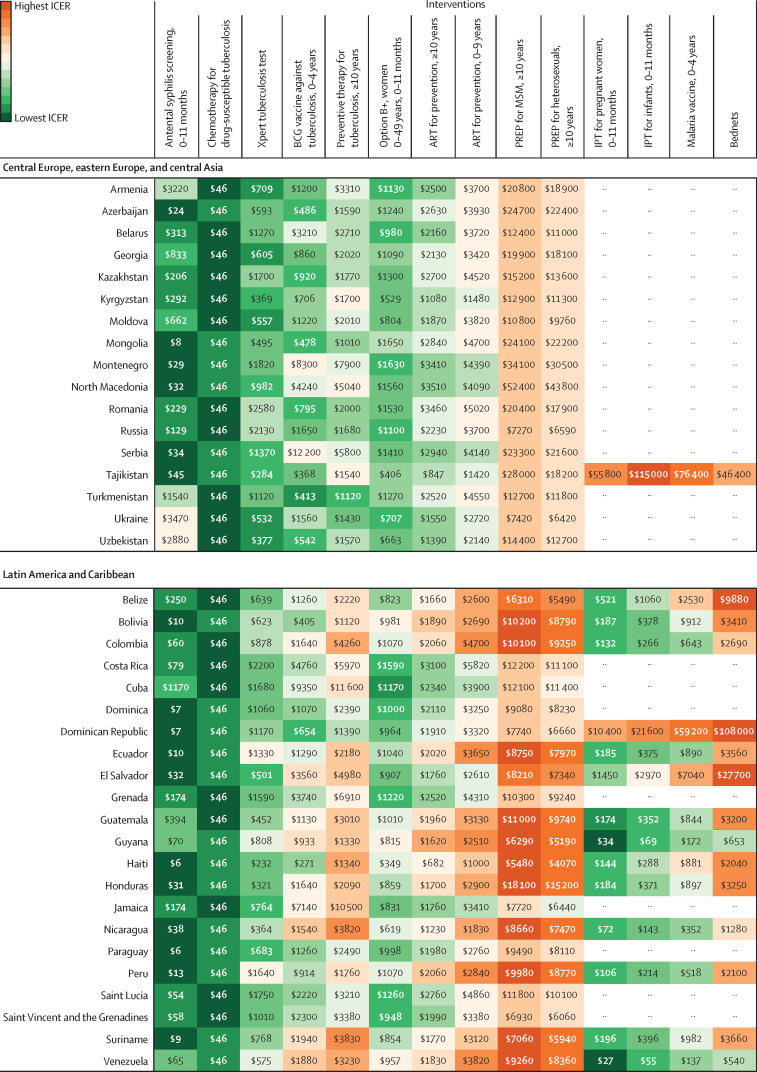

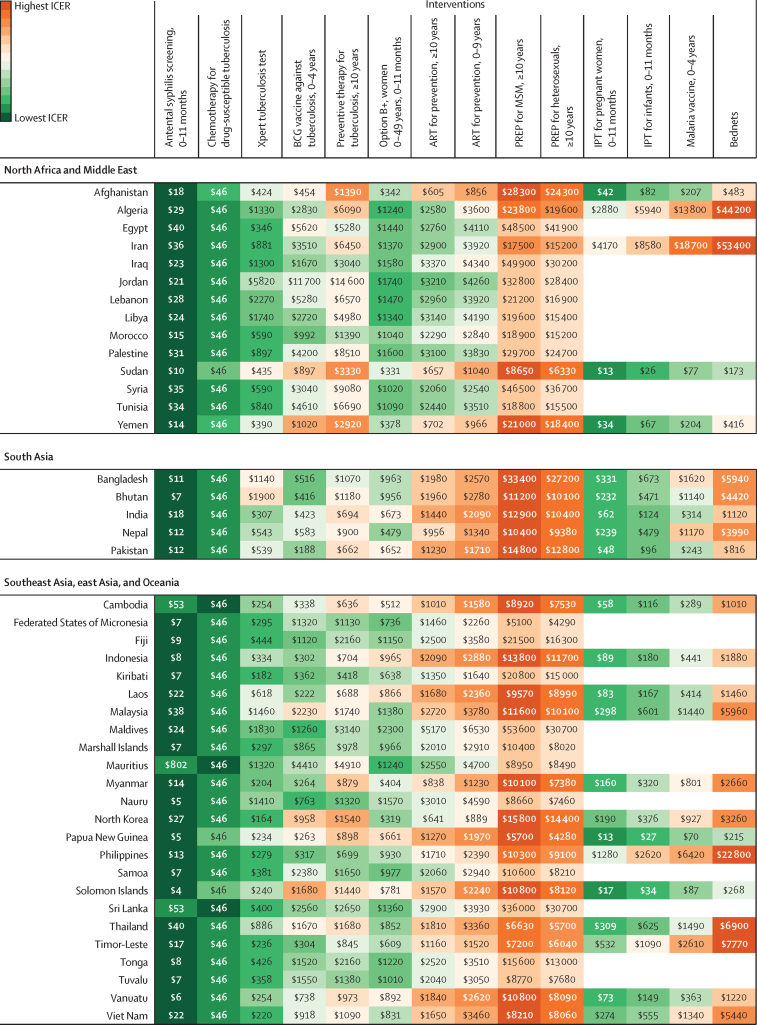

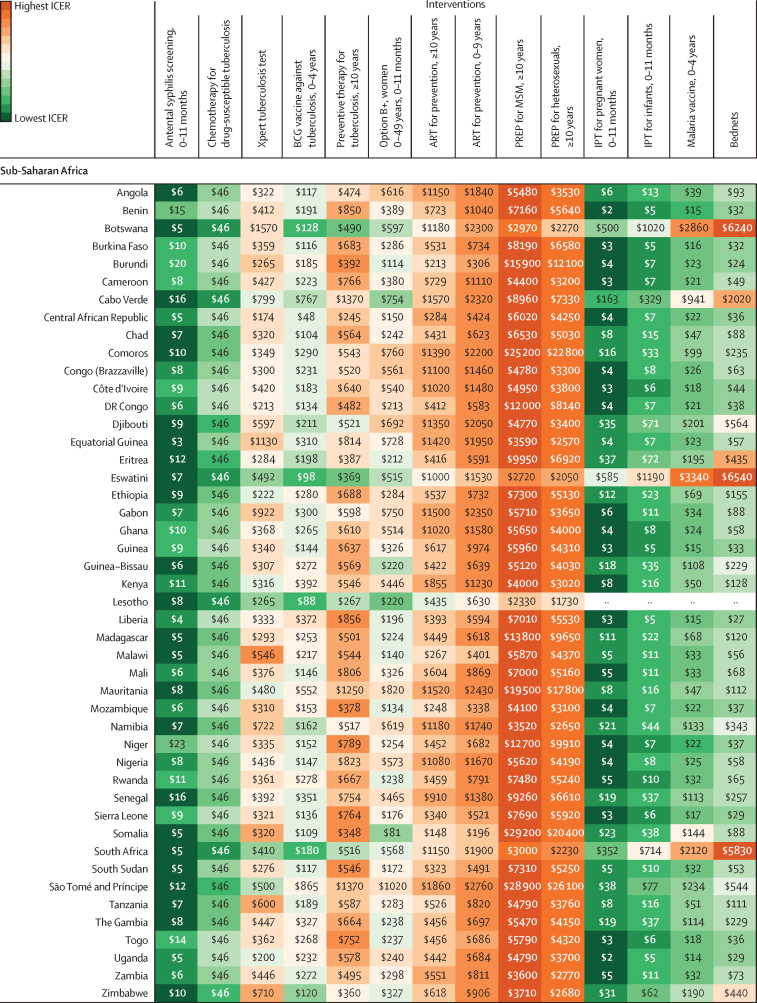
Heat map of 14 interventions by median predicted ICER, in 2019 US$ per DALY averted, for 128 countries, by GBD super-region Data are provided as integers to a maximum of three significant figures; results with all integers are available through the GHDx website. Data for malaria interventions are not provided for 45 countries that are certified as being malaria-free. Interventions without an age range are applicable for all age groups. The median ICERs are reported with IQRs and 95% uncertainty intervals for 128 countries in the appendix (pp 105–233). ART=antiretroviral therapy. DALY=disability-adjusted life-year. GBD=Global Burden of Diseases, Injuries, and Risk Factor Study. ICER=incremental cost-effectiveness ratio. IPT=intermittent preventive treatment for malaria. MSM=men who have sex with men. Option B+=HIV/AIDS screening for pregnant women and lifelong ART. PREP=pre-exposure prophylaxis.

Median ICERs for 128 countries are summarised in figure 4, ranking interventions from lowest median ICER to highest among 14 interventions for 83 countries with malaria and among ten interventions for 45 countries that are certified malaria-free. Antenatal syphilis screening ranks as the lowest median ICER in 81 (63%) of 128 countries, with median ICERs ranging from $3 (IQR 2–4) per DALY averted in Equatorial Guinea to $3473 (2244–5222) in Ukraine. The second lowest is chemotherapy for drug-susceptible tuberculosis, which has the lowest median ICER in 23 (18%) countries and the second lowest in 59 (46%) countries. The median ICER is $46 (IQR 20–113) per DALY averted in every country because we could only include one covariate in the sample of six ICERs (appendix p 103). The selected covariate was efficacy and has the same value for every country.

In the middle range, the rankings vary substantially across countries, depending on GDP per capita, burden of disease, and other parameter estimates. For example, Option B+ has the third lowest median ICER in 15 (12%) of 128 countries, fourth in 23 (18%) countries, ninth in 15 (12%) countries, and tenth in four (3%) countries; median ICERs range from $81 (IQR 36–174) per DALY averted in Somalia to $2296 (1114–4895) in Maldives.

Among the highest ICER values, PREP for men who have sex with men (MSM) has the highest median ICER in 116 (91%) of 128 countries; median ICERs range from $2326 (IQR 1077–4567) per DALY averted in Lesotho to $53 559 (23 841–108 534) in Maldives. PREP for heterosexuals aged 10 years or older has the second highest median ICER in 115 (90%) countries; median ICERs range from $1729 (IQR 822–3472) per DALY averted in Lesotho to $43 765 (19 446–87 245) in North Macedonia.

## Discussion

To our knowledge, in this study we produced the first country-specific league tables with ICERs for multiple countries, interventions, and causes, and found that the rankings differ substantially across economic and epidemiological contexts. The country-specific league tables can help national decision makers to prioritise currently recommended interventions for HIV, malaria, syphilis, and tuberculosis. The results call attention to interventions, such as antenatal syphilis screening, that have a low cost per DALY averted in the majority of countries and should be broadly adopted. Such ranking also puts interventions in context, such as PREP for MSM and heterosexuals aged 10 years and older in Botswana, Namibia, and South Africa, where the median ICER is below the country-specific threshold, and intermittent preventive treatment for malaria for pregnant women in Bolivia and Ecuador, where the median ICER is the third lowest among 14 interventions despite the burden of malaria being relatively low.

The ultimate goal of cost-effectiveness analyses is to rank interventions by priority. In this meta-regression analyses we synthesised model-based results with a focus on this goal. The models that underlie cost-effectiveness analyses are at odds with this goal because they are usually disease specific, with a focus on a few interventions in a single location or a single intervention in multiple locations. The meta-regression parameters can be readily used to predict ICERs across settings, by contrast with other methods to transfer results of cost-effectiveness analyses. For example, expert opinion does not lend itself to country-specific predictions,^15^ and case-by-case methods are often unsuccessful.^16^, ^17^

In our league tables we clearly present the uncertainty in the estimates by reporting IQRs and 95% UIs. Decision makers can readily see when the range of the ICERs are similar, even if one intervention has a lower median ICER than another.

When health maximisation with available funds is a criterion for prioritising interventions, ICERs ranked in country-specific league tables are a better guide for the allocation of funds than a single threshold. Funds could be first spent on the intervention that has the lowest ICER. Following that, other interventions could be funded in order of their ICER rankings, as long as there are available funds.^31^ This process eliminates the need for a single threshold for all health-care funding decisions. For example, our league tables support selecting among interventions for HIV/AIDS, malaria, syphilis, and tuberculosis, which have relatively stable donor funding. By contrast, the country-specific thresholds would suggest that three ART interventions would not be supported in India, Indonesia, and Sudan if the national budget was the only funding source. Although cost-effectiveness is an important criterion when deciding on interventions at the national level, it should not be the only criterion. Other criteria that should be considered include enhancing equity and providing financial risk protection.

The field of meta-regression analysis of cost-effectiveness analyses results is relatively new, and this research has limitations. First is the paucity of published cost-effectiveness analyses for key interventions with health outcomes measured in QALYs or DALYs. For voluntary medical male circumcision, several published cost-effectiveness analyses report ICERs calculated with HIV infections averted as the outcome. Pilot research has been conducted to convert ICERs from cost per HIV infection averted to cost per DALY averted (Dan Ollendorf, Tufts University, Boston, MA, USA, personal communication). These converted ICERs could be included in future analyses. For chemotherapy for drug-susceptible tuberculosis, several published cost-effectiveness analyses report ICERs calculated with cases cured or treatment completed as the outcome; these ICERs could also be converted to cost per DALY averted and included in future analyses. The number of cost-effectiveness analyses in the Tufts University registries increases each year, which will increase the sample size and provide an opportunity to expand the number of currently recommended interventions in future meta-regression analyses.

Second, we excluded dominated cost-effectiveness analyses results and were unable to use some of the cost-saving results in our probability estimates. The net-benefits approach is an alternative approach that assigns a health value to costs^30^ or monetary value to health benefits, where the value is based on a threshold. This approach would produce estimates for MDR-tuberculosis treatment and post-exposure prophylaxis for MSM with known exposure to HIV/AIDS, for which most published results were cost-saving. However, that approach is based on a threshold, and therefore has the limitations of thresholds as discussed earlier. We also excluded several population-based prevention interventions because of inconsistencies across articles in the definition of interventions and cost and efficacy variables.

Third, we predicted ICERs for three interventions for key populations (PREP for MSM, intermittent preventive treatment for malaria for pregnant women, and intermittent preventive treatment for malaria for infants), but not all interventions for key populations, such as preventive therapy for tuberculosis for people infected with HIV. The burden of disease is generally higher for key populations than the general population, so ICERs for the other 11 interventions might be an upper bound for the same interventions for key populations. For PREP for MSM and heterosexual aged 10 years and older, research to target it more effectively is ongoing, and future published cost-effectiveness analyses might report lower ICERs.

Fourth, there is unexplained heterogeneity in the published ICERs, as shown by the model fit statistics. In future analyses, researchers could seek covariates that are specific to a cause-type group, such as the key population variables when they are relevant to all the models, or intervention coverage, which was reported for the majority of malaria prevention ratios but missing for other groups.

League tables are an essential tool to guide the allocation of health resources. This research demonstrates that they must be created at the national level because ICERs and the rank of interventions differ across countries. Meta-regression analysis is a promising method to synthesise the results of cost-effectiveness analyses and provide the foundation for creating country-specific league tables.

### Contributors

### Data sharing

To download the data used in these analyses after publication, please visit the Global Health Data Exchange website. Data from GBD 2019 are available online.

## Declaration of interests

We declare no competing interests.
