# Supplementary material for: Cost-effectiveness of interventions for HIV/AIDS, malaria, syphilis, and tuberculosis in 128 countries: a meta-regression analysis
Source: Lancet Glob Health. 2024 Jun 12;12(7):e1159–73. doi: 10.1016/S2214-109X(24)00181-5 (PMC11194165; doi:10.1016/S2214-109X(24)00181-5)

THE LANCET
Global Health

Supplementary appendix

This appendix formed part of the original submission and has been peer reviewed. We post it as supplied by the authors.

Supplement to: Silke F, Earl L, Hsu J, et al. Cost-effectiveness of interventions for HIV/AIDS, malaria, syphilis, and tuberculosis in 128 countries: a meta-regression analysis. *Lancet Glob Health* 2024; **12:** e1159–73.

Methods appendix: Cost-effectiveness of interventions for HIV/AIDS, malaria, syphilis, and TB in 128 countries: A meta-regression analysis

Foreword

Sections of this supplementary material have been adapted from Cost-effectiveness of HPV vaccination in 195 countries: A meta-regression analysis [1]. Our goal as a research team is to provide evidence that will help stakeholders to make more informed and better decisions on the best value technologies to be adopted to address demand, and to support delivery of those interventions through efficient health systems.

To build on available evidence, we are conducting meta-regression analyses of published CEAs in the Tufts University CEA registries to quantify the effects of factors at the method, intervention, and county-level, and generate estimates of incremental CE ratios (ICERs) for multiple interventions in 128 countries. Therefore, we have endeavored to standardize our approach and processes where possible and to improve our processes where necessary. Hence, some of the information contained herein is largely adapted from Cost-effectiveness of HPV vaccination in 195 countries: A meta-regression analysis [1].

At the beginning of each section which largely bares resemblance to Cost-effectiveness of HPV vaccination in 195 countries: A meta-regression analysis [1], there will be an appropriate citation should you be interested in retrieving previous information.

Reference

1. Rosettie KL, Joffe JN, Sparks GW, Aravkin A, Chen S, Compton K, et al. Cost-effectiveness of HPV vaccination in 195 countries: A meta-regression analysis. *PLOS One* 2021;**16**: e0260808.

Table of Contents

Foreword 1

Section 1. GATHER Checklist of information that should be included in new reports of global health estimates........ 4

Section 2 Descriptive statistics on CEA articles in the analysis sample........ 6

Table S2 references 41

Section 3. Intervention Taxonomy 51

3.1: Intervention Taxonomy Overview 51

3.2. Guiding principles for intervention taxonomy 51

Section 3 References 54

Section 4. Data extractions and mapping 55

4.1. Overview 55

4.2. Null comparator 55

4.3. Time horizon 55

4.4. Discount rate 55

4.5. Age 55

4.6. Sex 56

4.7. Causes 56

4.8. Locations 56

4.9. Intervention Type 56

4.10. Efficacy 57

4.11. Sensitivity and Specificity 57

4.12: Commodity Costs 57

Section 4 References 58

Section 5. Background on five-stage meta-regression analysis 59

5.1. Introduction 59

5.2. Crosswalk analyses of sensitivity analysis covariates 59

5.3. Estimation of Nonlinear log-GDP per capita response curve 60

5.3.1. B-splines linear tails 60

5.3.2 Robust Trimming Strategy 61

5.3.3 Spline Ensemble 61

5.3.4 Sampling Knots from Simplex 61

5.3.5 Scoring 62

5.3.6 New nonlinear 'signal' covariate 62

5.4. Covariate Selection 63

5.5. Gaussian prior cross-validation 63

5.6. Meta-Regression Analysis 63
Section 5 References 64
Section 6. Implementation of the five-stage meta-regression analysis 65
6.1 Models 65
6.1.1 Grouping interventions by cause and type 65
6.1.2 Minimum of eight models estimated for each cause-type group 65
6.1.3 Crosswalk analysis for the HIV, malaria, and TB samples 65
6.1.4 Including sensitivity analyses in main analysis 66
6.2 Inclusion criteria and exclusions from the analysis sample and final meta-regression models 66
6.2.1 Inclusion criteria and exclusions from the analysis sample 66
6.2.2. Exclusions from the final meta-regression models 68
6.3 Descriptive statistics on final sample for meta-regression estimates 69
6.4 WHO guidelines, community costs, and efficacy of interventions with predicted ICERs 71
6.5 Logistic regression estimates 77
Section 6 References 77
Section 7. Results 80
7.1 Introduction 80
7.2 HIV/AIDS – antiretroviral therapy (ART) 81
7.3. HIV/AIDS pre-exposure prophylaxis (PREP) 86
7.4 Syphilis diagnostics 88
7.5 Malaria prevention 90
7.6 Tuberculosis prevention 94
7.7 Tuberculosis diagnostics 98
7.8 Tuberculosis treatment 101
7.9 List of 25 interventions included in the meta-regression analysis sample and 14 with predictions 104
Section 8. League tables 105

Section 1. GATHER Checklist of information that should be included in new reports of global health estimates

#	Checklist item	Reported on page
**Objectives and funding**		
**1**	Define the indicator(s), populations (including age, sex, and geographic entities), and time period(s) for which estimates were made.	method p 3-4
**2**	List the funding sources for the work.	abstract p 1, acknowledgments p15
**Data Inputs**		
For all data inputs from multiple sources that are synthesized as part of the study:		
**3**	Describe how the data were identified and how the data were accessed.	methods p 3-4
**4**	Specify the inclusion and exclusion criteria. Identify all ad-hoc exclusions.	methods p 4, Figure 2, results p 6, appendix section 6.2
**5**	Provide information on all included data sources and their main characteristics. For each data source used, report reference information or contact name/institution, population represented, data collection method, year(s) of data collection, sex and age range, diagnostic criteria or measurement method, and sample size, as relevant.	methods p 3, Table 1, appendix Tables S2 and S6.4
**6**	Identify and describe any categories of input data that have potentially important biases (e.g., based on characteristics listed in item 5).	methods p 4, Table 1, appendix Tables S2 and S6.4
For data inputs that contribute to the analysis but were not synthesized as part of the study:		
**7**	Describe and give sources for any other data inputs.	GBD: methods p 4; predictions: statistical analysis p 5 and appendix Tables S6.5 and S6.6; thresholds: statistical analysis p 5
For all data inputs:		
**8**	Provide all data inputs in a file format from which data can be efficiently extracted (e.g., a spreadsheet rather than a PDF), including all relevant meta-data listed in item 5. For any data inputs that cannot be shared because of ethical or legal reasons, such as third-party ownership, provide a contact name or the name of the institution that retains the right to the data.	To download data used in these analyses after publication, please visit the Global Health Data Exchange. https://ghdx.healthdata.org/record/ihme-data/global-fund-country-hiv-aids-malaria-syphilis-tuberculosis-intervention-cost-effectiveness-estimates
**Data analysis**		
**9**	Provide a conceptual overview of the data analysis method. A diagram may be helpful.	Figure 1
**10**	Provide a detailed description of all steps of the analysis, including mathematical formulae. This description should cover, as relevant, data cleaning, data pre-processing, data adjustments and weighting of data sources, and mathematical or statistical model(s).	methods p 4-5, appendix sections 3-6
**11**	Describe how candidate models were evaluated and how the final model(s) were selected.	statistical analysis p 5, appendix sections 5-7
**12**	Provide the results of an evaluation of model performance, if done, as well as the results of any relevant sensitivity analysis.	appendix section 7

**13**	Describe methods for calculating uncertainty of the estimates. State which sources of uncertainty were, and were not, accounted for in the uncertainty analysis.	statistical analysis p 5
**14**	State how analytic or statistical source code used to generate estimates can be accessed.	statistical analysis p5
**Results and Discussion**		
**15**	Provide published estimates in a file format from which data can be efficiently extracted.	To download results after publication, please visit the Global Health Data Exchange. https://ghdx.healthdata.org/record/ihme-data/global-fund-country-hiv-aids-malaria-syphilis-tuberculosis-intervention-cost-effectiveness-estimates
**16**	Report a quantitative measure of the uncertainty of the estimates (e.g. uncertainty intervals).	Figures 2a-2f, appendix section 8
**17**	Interpret results in light of existing evidence. If updating a previous set of estimates, describe the reasons for changes in estimates.	Research in Context
**18**	Discuss limitations of the estimates. Include a discussion of any modelling assumptions or data limitations that affect interpretation of the estimates.	Discussion p 14

Section 2 Descriptive statistics on CEA articles in the analysis sample

Table S2. Summary of articles in the analysis sample															
**Cause and intervention type/Title**	**PubMedID**	**Intervention or keyword**	**Minimum ICER (2019 US$)**	**Maximum ICER (2019 US$)**	**Year**	**Countries**	**Num-ber of ratios**	**Cost (2019 US$)**	**Efficacy**	**Time horizon**	**DALY or QALY**	**Out-come discount rate**	**Costs discount rate**	**Pers-pective**
**HIV/AIDS: Antiretroviral treatment (ART)**														
Determining A Cost Effective Intervention Response To HIV/AIDS In Peru. (2)	19765304	prevention of mother to child hiv transmission, antiretroviral therapy for hiv for prevention	2,781	7,176	2009	Peru	4	4,090.32, 4,590.26	23.3, missing	5 years	DALYs	3	0	payer (2)
Effectiveness And Cost Effectiveness Of Expanding Harm Reduction And Antiretroviral Therapy In A Mixed HIV Epidemic: A Modeling Analysis For Ukraine. (3)	21390264	antiretroviral therapy for hiv for prevention, methadone maintenance therapy	537	1,113	2011	Ukraine	3	967.53, 1,138.63	85, 87.1, 90	20 years	QALYs	3	3	payer (2)
Effectiveness And Cost Effectiveness Of Oral Pre-Exposure Prophylaxis In A Portfolio Of Prevention Programs For Injection Drug Users In Mixed HIV Epidemics. (4)	24489747	antiretroviral therapy for hiv for prevention, methadone maintenance therapy	388	863	2014	Ukraine	9	335.91, 559.85, 709.14, 1,045.04	73.6, 76.3, 78.1, 82.3, 82.7, 85, 89.6, 96	20 years	QALYs	3	3	health sector (4)
When To Initiate Highly Active Antiretroviral Therapy In Sub-Saharan Africa? A South African Cost-Effectiveness Study. (7)	16518961	antiretroviral therapy for hiv	65	824	2006	South Africa	6	354.47	missing	Lifetime	QALYs	0, 8	0, 8	payer (2)

Table S2. Summary of articles in the analysis sample														
**Cause and intervention type/Title**	**PubMedID**	**Intervention or keyword**	**Minimum ICER (2019 US$)**	**Maximum ICER (2019 US$)**	**Year**	**Countries**	**Num-ber of ratios**	**Cost (2019 US$)**	**Efficacy**	**Time horizon**	**DALY or QALY**	**Out-come discount rate**	**Costs discount rate**	**Pers-pective**
Cost-Effectiveness Of Newer Antiretroviral Drugs In Treatment-Experienced Patients With Multidrug-Resistant HIV Disease. (8)	24129369	antiretroviral therapy for hiv	58,891	86,413	2013	United States	2	20,599.11	35	Lifetime	QALYs	0,3	0,3	payer (2)
Cost-effectiveness of antiretroviral regimens in the World Health Organization's treatment guidelines: a South African analysis (10)	21124202	antiretroviral therapy for hiv	1,057	dominated	2011	South Africa	4	122.45, 388.61, 544.46, 683.11	2.6, 8.5, 14.7	Lifetime	QALYs	3	3	payer (2)
The Cost Effectiveness Of Antiretroviral Treatment Strategies In Resource-Limited Settings. (11)	17545710	antiretroviral therapy for hiv	507	4,309	2007	United States	20	128.84, 257.67, 386.51, 515.35, 644.18, 773.02, 901.86, 1,030.69, 1159.53, 1,288.37	51, 74	10 years	QALYs	3	3	societal (1)
Cost effectiveness of atazanavir-ritonavir versus lopinavir-ritonavir in treatment-naïve human immunodeficiency virus-infected patients in the United States (13)	21288058	antiretroviral therapy for hiv	15,524	69,790	2010	United States	3	5,497.79	9, 18.5, 29	Lifetime	QALYs	3	3	payer (2)
Cost effectiveness of darunavir/ritonavir combination antiretroviral therapy for treatment-naive adults with HIV-1 infection in Canada (14)	24906477	antiretroviral therapy for hiv	cost-saving	dominated	2014	Canada	6	15,315.34	3.4	5, 10 years, Lifetime	QALYs	0, 3, 5, 6	0, 3, 5, 6	payer (2)

Table S2. Summary of articles in the analysis sample														
**Cause and intervention type/Title**	**PubMedID**	**Intervention or keyword**	**Minimum ICER (2019 US$)**	**Maximum ICER (2019 US$)**	**Year**	**Countries**	**Num-ber of ratios**	**Cost (2019 US$)**	**Efficacy**	**Time horizon**	**DALY or QALY**	**Out-come discount rate**	**Costs discount rate**	**Pers-pective**
Cost Effectiveness Of Screening Strategies For Early Identification Of HIV And HCV Infection In Injection Drug Users. (18)	23028828	antiretroviral therapy for hiv for prevention, hiv testing	13,263	52,990	2012	United States	7	26,072.86	50	20 years	QALYs	3	3	payer (2)
The cost-effectiveness of antiretroviral treatment in Khayelitsha, South Africa--a primary data analysis (19)	17147833	antiretroviral therapy for hiv	1,097	1,175	2006	South Africa	2	6,692	76.2	Lifetime	QALYs	3	3	payer (2)
Assessing Efficiency And Costs Of Scaling Up HIV Treatment. (20)	18664951	antiretroviral therapy for hiv	1,097	1,614	2008	South Africa	4	1,410.14, 1,906.89	missing	Lifetime	QALYs	0, 3	0, 3	payer (2)
The Cost-Effectiveness Of Expanded Testing For Primary HIV Infection.(21)	16189054	antiretroviral therapy for hiv, hiv testing	6,469	dominated	2005	United States	3	26.72, 34.35, 165.69	60	Lifetime	QALYs	3	3	societal (1)

Table S2. Summary of articles in the analysis sample														
**Cause and intervention type/Title**	**PubMedID**	**Intervention or keyword**	**Minimum ICER (2019 US$)**	**Maximum ICER (2019 US$)**	**Year**	**Countries**	**Num-ber of ratios**	**Cost (2019 US$)**	**Efficacy**	**Time horizon**	**DALY or QALY**	**Out-come discount rate**	**Costs discount rate**	**Pers-pective**
Cost-Effectiveness Analysis Of Initial HIV Treatment Under Italian Guidelines. (22)	22163167	antiretroviral therapy for hiv	22,328	64,484	2011	Italy	77	7,137.41, 7,206.54, 7,376.71, 7,685.14, 8,921.77, 9,008.18, 9,110.28, 9,220.89, 9,405.94, 9,588.87, 9,606.42, 9,884.54, 10,706.12, 10,809.81, 10,813.01, 11,065.06, 11,387.85, 11,527.70, 11,757.43, 11,986.09, 12,355.67, 13,516.26, 13,665.42, 13,991.92, 14,108.91, 14,383.31, 14,826.80, 16,219.51, 17,489.91, 20,987.89	47.2, 48, 51.2, 53.4, 54.4, 55.8, 59, 59.2, 60, 63.2, 64, 64.8, 66.8, 68, 69.7, 70.8, 72, 74, 76.8, 79, 80.2, 81, 81.6, 83.64, 88.8, 94.8, 97.2	10 years	QALYs	0, 3.5, 5	0, 3.5, 5	payer (2)

Table S2. Summary of articles in the analysis sample														
**Cause and intervention type/Title**	**PubMedID**	**Intervention or keyword**	**Minimum ICER (2019 US$)**	**Maximum ICER (2019 US$)**	**Year**	**Countries**	**Num-ber of ratios**	**Cost (2019 US$)**	**Efficacy**	**Time horizon**	**DALY or QALY**	**Out-come discount rate**	**Costs discount rate**	**Pers-pective**
Economic evaluation of initial antiretroviral therapy for HIV-infected patients: an update of Italian guidelines (23)	24124383	antiretroviral therapy for hiv	1,383	37,757	2013	Italy	40	884.14, 8,376.32, 8,407.74, 8,617.17, 8,760.16, 9,307.02, 9,341.93, 9,574.64, 9,636.18, 9,730.68, 10,237.73, 10,254.27, 10,276.12, 10,456.73, 10,532.10, 10,811.87, 11,393.64, 11,618.59, 11,893.06, 12,356.79, 12,533.00, 12,780.45, 13,729.77, 15,102.74	60.8, 67.5, 74.2, 74.7, 75.6, 76.5, 80.1, 81, 83. 83.7, 84, 85, 89, 90, 91.3, 92.4, 93, 93.5, 97.9, 99. 102.3	10 years	QALYs	3.5	3.5	payer (2)
Antiretroviral Therapy In HIV-Infected Patients: A Proposal To Assess The Economic Value Of The Single-Tablet Regimen. (24)	23430273	antiretroviral therapy for hiv	27,128	34,999	2013	Italy	64	6,534.38, 6,628.74, 6,723.10, 6,818.77, 6,913.13, 7,007.49, 7,103.16, 7,197.52, 7,291.88, 7,386.24, 7,481.91, 7,576.27, 7,670.63, 7,764.99, 7,860.65, 7,955.01, 8,049.37, 8,143.73, 8,239.40, 8,333.76, 8,428.12, 8,522.48,	80	10 years	QALYs	3.5	3.5	payer (2)

Table S2. Summary of articles in the analysis sample

Cause and intervention type/Title	PubMedID	Intervention or keyword	Minimum ICER (2019 US$)	Maximum ICER (2019 US$)	Year	Countries	Num-ber of ratios	Cost (2019 US$)	Efficacy	Time horizon	DALY or QALY	Out-come discount rate	Costs discount rate	Pers-pective
								8,618.15,<br>8,712.51,<br>8,806.87,<br>8,901.23,<br>8,996.90,<br>9,091.26,<br>9,185.62,<br>9,279.98,<br>9,375.65,<br>9,470.01,<br>9,470.01,<br>9,564.36,<br>9,660.03,<br>9,754.39,<br>9,848.75,<br>9,943.11,<br>10,038.78,<br>10,133.14,<br>10,227.50,<br>10,321.86,<br>10,417.53,<br>10,511.89,<br>10,606.25,<br>10,700.61,<br>10,796.28,<br>10,890.64,<br>10,985.00,<br>11,079.36,<br>11,175.03,<br>11,269.39,<br>11,363.74,<br>11,458.10,<br>11,553.77,<br>11,648.13,<br>11,742.49,<br>11,838.16,<br>11,932.52,<br>12,026.88,<br>12,121.24,<br>12,211.67,<br>12,216.91,<br>12,311.27						

Table S2. Summary of articles in the analysis sample														
**Cause and intervention type/Title**	**PubMedID**	**Intervention or keyword**	**Minimum ICER (2019 US$)**	**Maximum ICER (2019 US$)**	**Year**	**Countries**	**Num-ber of ratios**	**Cost (2019 US$)**	**Efficacy**	**Time horizon**	**DALY or QALY**	**Out-come discount rate**	**Costs discount rate**	**Pers-pective**
Cost-Effectiveness Of Dolutegravir In HIV-1 Treatment-Naive And Treatment-Experienced Patients In Canada. (28)	26099626	antiretroviral therapy for hiv	cost-saving	cost-saving	2015	Canada	8	12,834.69, 13,141.13, 14,027.28, 14,698.43, 15,071.86, 15,397.96, 16,268.30, 21,123.17	2.9, 5.7, 6, 6.9, 8.2, 10.1, 11.7, 15.1	Lifetime	QALYs	5	5	payer (2)
Cost-Effectiveness Of Targeted Human Immunodeficiency Virus Screening In An Urban Emergency Department. (31)	21762236	antiretroviral therapy for hiv for prevention, hiv testing	130	130	2011	United States	1	2,146.27	missing	1.3 years	QALYs	3	3	payer (2)
Health benefits, costs, and cost-effectiveness of earlier eligibility for adult antiretroviral therapy and expanded treatment coverage: a combined analysis of 12 mathematical models (34)	25083415	antiretroviral therapy for hiv	cost-saving	16,924	2013	Inida, South Africa, Vietnam, Zambia	234	89.4, 108.67, 115.57, 117.55	90	5, 10, 20 years	DALYs	0, 3, 6	0, 3, 6	health sector (4)
The Cost Effectiveness Of Combination Antiretroviral Therapy For HIV Disease. (35)	11248160	antiretroviral therapy for hiv	19,537	25,548	2001	United States	3	13,435.45, 15,629.61, 17,44805	53	Lifetime	QALYs	3	3	societal (1)
Projecting The Cost-Effectiveness Of Adherence Interventions In Persons With Human Immunodeficiency Virus Infection. (37)	14656616	antiretroviral therapy for hiv	32,098	105,251	2003	United States	36	14,348.40, 21,140.55, 29,630.72	64, 68, 73, 76, 82, 84, 88, 92, 94, 100	Lifetime	QALYs	3	3	societal (1)

Table S2. Summary of articles in the analysis sample														
**Cause and intervention type/Title**	**PubMedID**	**Intervention or keyword**	**Minimum ICER (2019 US$)**	**Maximum ICER (2019 US$)**	**Year**	**Countries**	**Num-ber of ratios**	**Cost (2019 US$)**	**Efficacy**	**Time horizon**	**DALY or QALY**	**Out-come discount rate**	**Costs discount rate**	**Pers-pective**
Expanding ART For Treatment And Prevention Of HIV In Sou0th Africa: Estimated Cost And Cost-Effectiveness 2011-2050. (39)	22348000	antiretroviral therapy for hiv for prevention	cost-saving	147	2012	South Africa	6	154.57	92	5, 40 years	DALYs	3	3	payer (2)
Impact And Cost-Effectiveness Of Point-Of-Care Cd4 Testing On The HIV Epidemic In South Africa. (42)	27391129	antiretroviral therapy for hiv for prevention, hiv testing	3,138	10,485	2016	South Africa	4	130.15	1.3, 1.7, 3.1	1, 3 years	DALYs	3	3	payer (2)
Cost-Effectiveness Of Enfuvirtide For Treatment-Experienced Patients With HIV In Italy. (44)	15983893	antiretroviral therapy for hiv	40,833	44,151	2005	Italy	5	46,538.52	73.2	7, 10, 20 years	QALYs	0, 3, 5	0, 3, 5	payer (2)
Cost-Effectiveness Of Enfuvirtide In HIV Therapy For Treatment-Experienced Patients In The United States. (45)	16545010	antiretroviral therapy for hiv	19,718	36,102	2006	United States	3	28,149.50	63.2	7, 10, 20 years	QALYs	3	3	payer (2)
Cost-Effectiveness Of Pooled Nucleic Acid Amplification Testing For Acute HIV Infection After Third-Generation HIV Antibody Screening And Rapid Testing In The United States: A Comparison Of Three Public Health Settings. (47)	20927354	pooled hiv testing, antiretroviral therapy for hiv for prevention	cost-saving	1,521,446	2010	United States	14	6,639.89, 13,279.78, 19,919.66	missing	Lifetime	QALYs	3	3	payer (2)

Table S2. Summary of articles in the analysis sample															
**Cause and intervention type/Title**	**PubMedID**	**Intervention or keyword**	**Minimum ICER (2019 US$)**	**Maximum ICER (2019 US$)**	**Year**	**Countries**	**Num-ber of ratios**	**Cost (2019 US$)**	**Efficacy**	**Time horizon**	**DALY or QALY**	**Out-come discount rate**	**Costs discount rate**	**Pers-pective**
Cost And Cost-Effectiveness Of Switching From D4T Or Azt To A Tdf-Based First-Line Regimen In A Resource-Limited Setting In Rural Lesotho. (49)	21765366	antiretroviral therapy for hiv	873	913	2011	Lesotho	2	152.98, 185.88	missing	1 year	QALYs	0	0	payer (2)
The Cost-Effectiveness Of Symptom-Based Testing And Routine Screening For Acute HIV Infection In Men Who Have Sex With Men In The Usa. (50)	21716076	antiretroviral therapy for hiv for prevention, hiv testing	14,911	45,982	2011	United States	5	18,339.89	90	20 years	QALYs	3	3	payer (2)
Cd4 Cell Count And Viral Load Monitoring In Patients Undergoing Antiretroviral Therapy In Uganda: Cost Effectiveness Study. (52)	22074713	antiretroviral therapy for hiv	111	1,085	2011	Uganda	4	150.49, 154.35	36.2, 45.9, 56.4	15 years	DALYs	missing	missing	payer (2)
Evaluating The Cost-Effectiveness Of Combination Antiretroviral Therapy For The Prevention Of Mother-To-Child Transmission Of HIV In Uganda. (58)	22893743	prevention of mother to child hiv transmission, antiretroviral therapy for hiv for prevention	18	413	2012	Uganda	10	7.85, 10.33, 23.55, 70.95, 132.92, 283.80	90.5	1.5 years	DALYs	0, 3, 6	0, 3, 6	payer (2)
The Epidemiological Impact And Cost-Effectiveness Of HIV Testing, Antiretroviral Treatment And Harm Reduction Programs. (63)	22781221	antiretroviral therapy for hiv for prevention, antiretroviral therapy for hiv for prevention, hiv testing	6,284	73,284	2012	China	5	6,207.87	90	30 years	QALYs	3	3	payer (2)

Table S2. Summary of articles in the analysis sample															
**Cause and intervention type/Title**	**PubMedID**	**Intervention or keyword**	**Minimum ICER (2019 US$)**	**Maximum ICER (2019 US$)**	**Year**	**Countries**	**Num-ber of ratios**	**Cost (2019 US$)**	**Efficacy**	**Time horizon**	**DALY or QALY**	**Out-come discount rate**	**Costs discount rate**	**Pers-pective**
HIV Screening Via Fourth-Generation Immunoassay Or Nucleic Acid Amplification Test In The United States: A Cost-Effectiveness Analysis. (66)	22110698	antiretroviral therapy for hiv for prevention, hiv testing, pooled hiv testing, antiretroviral therapy for hiv for prevention	6,874	239,159	2012	United States	24	17,746.14	90	20 years	QALYs	3	3	societal (1)
The Cost-Effectiveness And Population Outcomes Of Expanded HIV Screening And Antiretroviral Treatment In The United States. (67)	21173412	antiretroviral therapy for hiv for prevention, antiretroviral therapy for hiv for prevention, hiv testing	24,058	170,576	2010	United States	8	18,453.66	2.3, 4.8, 6.7, 7.3, 7.7, 10.3, 65	20 years	QALYs	3	3	payer (2)
Expanded HIV Testing In Low-Prevalence, High-Income Countries: A Cost-Effectiveness Analysis For The United Kingdom. (68)	24763373	antiretroviral therapy for hiv for prevention, hiv testing, antiretroviral therapy for hiv for prevention	26,250	144,298	2014	United Kingdom	7	21,440.72	96	10 years	QALYs	3	3	payer (2)
Portfolios of biomedical HIV interventions in South Africa: a cost-effectiveness analysis (69)	23588668	antiretroviral therapy for hiv for prevention, hiv testing, antiretroviral therapy for hiv for prevention	76	591	2013	South Africa	2	410.92	96	Lifetime	QALYs	0	0	missing
The Costs And Benefits Of Private Sector Provision Of Treatment To HIV-Infected Employees In Kampala, Uganda. (72)	16549976	antiretroviral therapy for hiv	cost-saving	cost-saving	2006	Uganda	1	1,769.78	85	5 years	DALYs	3	3	limited societal (3)

Table S2. Summary of articles in the analysis sample														
**Cause and intervention type/Title**	**PubMedID**	**Intervention or keyword**	**Minimum ICER (2019 US$)**	**Maximum ICER (2019 US$)**	**Year**	**Countries**	**Num-ber of ratios**	**Cost (2019 US$)**	**Efficacy**	**Time horizon**	**DALY or QALY**	**Out-come discount rate**	**Costs discount rate**	**Pers-pective**
Cost Effectiveness of Darunavir/Ritonavir 600/100mg bid in Protease Inhibitor-Experienced, HIV-1-Infected Adults in Belgium, Italy, Sweden and the UK (76)	21182347	antiretroviral therapy for hiv	11,147	24,679	2010	Belgium, Italy, Sweden, United Kingdom	12	19,831.53, 20,690.35, 26,442.04, 27,587.13, 28,082.14, 29,598.75, 33,052.54, 34,483.91, 37,442.85, 39,465.00, 46,803.56, 49,331.24	73.1	Lifetime	QALYs	3	3	payer (2)
Cost Effectiveness of Darunavir/Ritonavir 600/100mg bid in Treatment-Experienced, Lopinavir-Naive, Protease Inhibitor-Resistant, HIV-Infected Adults in Belgium, Italy, Sweden and the UK (77)	21182349	antiretroviral therapy for hiv	3,746	22,922	2010	Belgium, Italy, Sweden, United Kingdom	12	12,963.98, 15,055.24, 17,285.3, 18,510.28, 18,561.11, 20,073.65, 21,606.63, 24,680.37, 25,092.06, 30,805.47, 30,935.18	34.7	Lifetime	QALYs	1.5, 3	3	payer (2)
Cost-Effectiveness Of Optimized Background Therapy Plus Maraviroc For Previously Treated Patients With R5 HIV-1 Infection From The Perspective Of The Spanish Health Care System. (78)	21316539	antiretroviral therapy for hiv	cost-saving	422,340	2010	Spain	5	27,511.84	60	5, 10 years, Lifetime	QALYs	3	3	payer (2)

Table S2. Summary of articles in the analysis sample														
**Cause and intervention type/Title**	**PubMedID**	**Intervention or keyword**	**Minimum ICER (2019 US$)**	**Maximum ICER (2019 US$)**	**Year**	**Countries**	**Num-ber of ratios**	**Cost (2019 US$)**	**Efficacy**	**Time horizon**	**DALY or QALY**	**Out-come discount rate**	**Costs discount rate**	**Pers-pective**
Cost-Effectiveness Of Adding An Agent That Improves Immune Responses To Initial Antiretroviral Therapy (Art) In HIV-Infected Patients: Guidance For Drug Development. (79)	22306583	antiretroviral therapy for hiv	39,182	40,855	2012	United States	22	20,336.83	86	Lifetime	QALYs	3	3	payer (2)
Increasing The Use Of Second-Line Therapy Is A Cost-Effective Approach To Prevent The Spread Of Drug-Resistant HIV: A Mathematical Modelling Study. (81)	25491351	antiretroviral therapy for hiv for prevention	1,921	dominated	2014	Uganda	6	191.80, 230.50	8.7, 9.4, 95	10 years	QALYs	3	3	missing
Cost Effectiveness Of Protease Inhibitor Monotherapy Versus Standard Triple Therapy In The Long-Term Management Of HIV Patients: Analysis Using Evidence From The Pivot Trial. (82)	26966125	antiretroviral therapy for hiv	cost-saving	cost-saving	2016	United Kingdom	1	7,167.56	87.8	3 years	QALYs	3.5	3.5	payer (2)
Cost-Effectiveness Analysis Of Antiretroviral Drug Treatment And HIV-1 Vaccination In Thailand. (83)	16785697	antiretroviral therapy for hiv	400	7,643	2006	Thailand	5	63.23, 632.27, 6,322.66	missing	Lifetime	DALYs	0, 3, 6	0, 3, 6	limited societal (3)

Table S2. Summary of articles in the analysis sample														
**Cause and intervention type/Title**	**PubMedID**	**Intervention or keyword**	**Minimum ICER (2019 US$)**	**Maximum ICER (2019 US$)**	**Year**	**Countries**	**Num-ber of ratios**	**Cost (2019 US$)**	**Efficacy**	**Time horizon**	**DALY or QALY**	**Out-come discount rate**	**Costs discount rate**	**Pers-pective**
Cost-Effectiveness Of Integrated Routine Offering Of Prenatal HIV And Syphilis Screening In China. (84)	24413489	prevention of mother to child hiv transmission, antiretroviral therapy for hiv for prevention	7,318	7,318	2014	China	1	148.39	73	Lifetime	DALYs	3	3	limited societal (3)
Expanded HIV Screening In The United States: Effect On Clinical Outcomes, HIV Transmission, And Costs. (85)	17146064	antiretroviral therapy for hiv for prevention, hiv testing	40,958	77,509	2006	United States	4	9.31	80	Lifetime	QALYs	3	3	societal (1)
Expanded Screening For HIV In The United States--An Analysis Of Cost-Effectiveness. (86)	15703423	antiretroviral therapy for hiv, hiv testing	50,941	1,788,598	2005	United States	12	17,232.23	70	Lifetime	QALYs	3	3	societal (1)
Cost-Effectiveness Of Dtg + Abc/3Tc Versus Efv/Tdf/Ftc For First-Line Treatment Of HIV-1 In The United States. (87)	25934146	antiretroviral therapy for hiv	176,926	2,530,437	2015	United States	4	31,542.35, 44,334.43	10.8	5, 20 years, Lifetime	QALYs	3	3	payer (2)
Estimating The Cost-Effectiveness Of HIV Prevention Programmes In Vietnam, 2006-2010: A Modelling Study. (89)	26196290	antiretroviral therapy for hiv for prevention	173	173	2015	Vietnam	1	363.25	96	5 years	DALYs	3	3	payer (2)
The Cost-Effectiveness Of Expanded HIV Counselling And Testing In Primary Care Settings: A First Look. (90)	11061658	antiretroviral therapy for hiv, hiv testing	34,529	34,970	2000	United States	2	50,231.77	missing	1 year	QALYs	3	3	societal (1)

Table S2. Summary of articles in the analysis sample														
**Cause and intervention type/Title**	**PubMedID**	**Intervention or keyword**	**Minimum ICER (2019 US$)**	**Maximum ICER (2019 US$)**	**Year**	**Countries**	**Num-ber of ratios**	**Cost (2019 US$)**	**Efficacy**	**Time horizon**	**DALY or QALY**	**Out-come discount rate**	**Costs discount rate**	**Pers-pective**
Cost-Effectiveness Of Dolutegravir In HIV-1 Treatment-Experienced (Te) Patients In France. (91)	26714188	antiretroviral therapy for hiv	21,492	35,028	2015	France	3	24,243.47	10.2	Lifetime	QALYs	0, 4, 6	0, 4, 6	payer (2)
Cost-Effectiveness Of HIV Screening In STD Clinics, Emergency Departments, And Inpatient Units: A Model-Based Analysis. (92)	21625489	antiretroviral therapy for hiv for prevention, hiv testing	cost-saving	cost-saving	2011	United States	2	4,909.99	80.5	Lifetime	QALYs	3	3	payer (2)
The Clinical Role And Cost-Effectiveness Of Long-Acting Antiretroviral Therapy. (95)	25583979	antiretroviral therapy for hiv	cost-saving	2,149,546	2015	United States	9	27.033.59, 30,412,79, 54,067.17	91	Lifetime	QALYs	3	3	payer (2)
Cost-effectiveness of HIV screening in patients older than 55 years of age. (96)	18559840	antiretroviral therapy for hiv, hiv testing	36,770	262,604	2008	United States	12	3,789.03	80	Lifetime	QALYs	3	3	societal (1)
Cost-Effectiveness Of Screening For HIV In The Era Of Highly Active Antiretroviral Therapy. (97)	15703422	antiretroviral therapy for hiv for prevention, hiv testing	19,845	33,245	2005	United States	2	6,776.73	20	Lifetime	QALYs	3	3	payer (2)
Cost-Effectiveness Of Enfuvirtide In Treatment-Experienced Patients With Advanced HIV Disease. (98)	15851916	antiretroviral therapy for hiv	42,026	138,531	2005	United States	9	27,168.57, 52,073.1, 65,232.87	43.4, 53.5, 60.5, 69.7, 75.4, 79.3, 83.2	Lifetime	QALYs	3	3	payer (2)

**Table S2. Summary of articles in the analysis sample**															
**Cause and intervention type/Title**	**PubMedID**	**Intervention or keyword**	**Minimum ICER (2019 US$)**	**Maximum ICER (2019 US$)**	**Year**	**Countries**	**Num-ber of ratios**	**Cost (2019 US$)**	**Efficacy**	**Time horizon**	**DALY or QALY**	**Out-come discount rate**	**Costs discount rate**	**Pers-pective**
Early Antiretroviral Therapy For Patients With Acute AIDS-Related Opportunistic Infections: A Cost-Effectiveness Analysis Of Actg A5164. (99)	21126955	antiretroviral therapy for hiv	45,681	45,685	2010	United States	3	1,707.61	58, 62, 70	Lifetime	QALYs	3	3	societal (1)
Cost-Effectiveness Of Earlier Initiation Of Antiretroviral Therapy For Uninsured HIV-Infected Adults.(100)	11527782	antiretroviral therapy for hiv	14,578	37,571	2001	United States	5	7,412.04, 14,824.08, 22,236.12	60, 70	Lifetime	QALYs	3	3	societal (1)
Cost-Effectiveness Implications Of The Timing Of Antiretroviral Therapy In HIV-Infected Adults.(101)	12437408	antiretroviral therapy for hiv	19,573	38,197	2002	United States	8	17,007.40	70	Lifetime	QALYs	3	3	payer (2)
Cost-Effectiveness Of Cyp2B6 Genotyping To Optimize Efavirenz Dosing In HIV Clinical Practice. (102)	26607811	antiretroviral therapy for hiv	cost-saving	dominated	2015	United States	8	17,622.10, 22,061.76	(-10.7, -7.4, -4.0, 91	Lifetime	QALYs	3	3	payer (2)
Potential Risks And Benefits Of HIV Treatment Simplification: A Simulation Model Of A Proposed Clinical Trial. (104)	17879926	antiretroviral therapy for hiv	cost-saving	932,778	2007	United States	2	17,315.65, 26,591.89	0, 24.7	Lifetime	QALYs	3	3	payer (2)
The Cost-Effectiveness Of Hla-B*5701 Genetic Screening To Guide Initial Antiretroviral Therapy For HIV. (105)	18784465	antiretroviral therapy for hiv	41,248	41,248	2008	United States	1	17.084.35	81	Lifetime	QALYs	3	3	payer (2)

Table S2. Summary of articles in the analysis sample														
**Cause and intervention type/Title**	**PubMedID**	**Intervention or keyword**	**Minimum ICER (2019 US$)**	**Maximum ICER (2019 US$)**	**Year**	**Countries**	**Num-ber of ratios**	**Cost (2019 US$)**	**Efficacy**	**Time horizon**	**DALY or QALY**	**Out-come discount rate**	**Costs discount rate**	**Pers-pective**
The potential cost and benefits of raltegravir in simplified second-line therapy among HIV infected patients in Nigeria and South Africa (107)	23457450	antiretroviral therapy for hiv	7,820	22,076	2013	Nigeria, South Africa	6	1,490.05, 9,218.74	76.9	50 years	QALYs	0, 3, 5	0, 3, 5	2
The Epidemiologic And Economic Impact Of Improving HIV Testing, Linkage, And Retention In Care In The United States. (110)	26362321	antiretroviral therapy for hiv for prevention, hiv testing, antiretroviral therapy for hiv for prevention	36,761	118,696	2015	United States	6	17,709.65, 18,036.34, 19,125.29, 26.203.49	93	20 years	QALYs	3	3	health sector (4)
Economics Of Switching To Second-Line Antiretroviral Therapy With Lopinavir/Ritonavir In Africa: Estimates Based On Dart Trial Results And Costs For Uganda And Kenya. (112)	22152173	antiretroviral therapy for hiv	1,815	2,083	2011	Kenya, Uganda	5	553.72, 692.16, 830.59, 1,005.22	27.9	Lifetime	QALYs	0, 3	0, 3	payer (2)
Lopinavir/Ritonavir Versus Darunavir Plus Ritonavir For HIV Infection: A Cost-Effectiveness Analysis For The United States. (113)	23620210	antiretroviral therapy for hiv	cost-saving	cost-saving	2013	United States	1	23,694.74	7.1	Lifetime	QALYs	3	3	payer (2)
Cost-Effectiveness Of Voluntary HIV Screening In Russia (119)	19103893	antiretroviral therapy for hiv, hiv testing	7,149	18,270	2009	Russia	4	1,736.26	20	Lifetime	QALYs	3	3	payer (2)

Table S2. Summary of articles in the analysis sample														
**Cause and intervention type/Title**	**PubMedID**	**Intervention or keyword**	**Minimum ICER (2019 US$)**	**Maximum ICER (2019 US$)**	**Year**	**Countries**	**Num-ber of ratios**	**Cost (2019 US$)**	**Efficacy**	**Time horizon**	**DALY or QALY**	**Out-come discount rate**	**Costs discount rate**	**Pers-pective**
Cost-Effectiveness Of Antiretroviral Therapy Expansion Strategies In Vietnam. (120)	24983389	antiretroviral therapy for hiv	484	484	2014	Vietnam	1	2,505.03	76.6	10 years	QALYs	3	3	payer (2)
Comparative Cost-Effectiveness Of Option B+ For Prevention Of Mother-To-Child Transmission Of HIV In Malawi. (121)	26691682	prevention of mother to child hiv transmission, antiretroviral therapy for hiv for prevention	731	731	2016	Malawi	1	190.02	12.8	Lifetime	DALYs	3	3	payer (2)
Resource Utilization And Cost-Effectiveness Of Counselor- Vs. Provider-Based Rapid Point-Of-Care HIV Screening In The Emergency Department. (126)	22022415	antiretroviral therapy for hiv, hiv testing	76,796	85,329	2011	United States	2	20,336.83	5, 9.9	Lifetime	QALYs	3	3	payer (2)
Economic Savings Versus Health Losses: The Cost-Effectiveness Of Generic Antiretroviral Therapy In The United States. (127)	23318310	antiretroviral therapy for hiv	22,347	30,248	2013	United States	9	2,725.80, 3,436.88, 5,451.60, 6,873.75, 8,177.40, 10,310.63, 10,903.20, 13,747.51, 18,132.49	78, 84, 85	1 year	QALYs	3	3	payer (2)
The Clinical Impact And Cost-Effectiveness Of Routine, Voluntary HIV Screening In South Africa. (128)	21068674	antiretroviral therapy for hiv, hiv testing	1,475	2,835	2011	South Africa	7	222.47	75	Lifetime	QALYs	3	3	payer (2)
Routine HIV Screening In France: Clinical Impact And Cost-Effectiveness. (134)	20976112	antiretroviral therapy for hiv for prevention, hiv testing	77,812	dominated	2010	France	4	12,710.88	91.8	Lifetime	QALYs	3	3	payer (2)

Table S2. Summary of articles in the analysis sample														
**Cause and intervention type/Title**	**PubMedID**	**Intervention or keyword**	**Minimum ICER (2019 US$)**	**Maximum ICER (2019 US$)**	**Year**	**Countries**	**Num-ber of ratios**	**Cost (2019 US$)**	**Efficacy**	**Time horizon**	**DALY or QALY**	**Out-come discount rate**	**Costs discount rate**	**Pers-pective**
Cost-Effectiveness Of Pre-Exposure Prophylaxis Targeted To High-Risk Serodiscordant Couples As A Bridge To Sustained Art Use In Kampala, Uganda. (135)	26198348	antiretroviral therapy for hiv for prevention	925	925	2015	Uganda	1	279.52	75	Lifetime	DALYs	3	3	payer (2)
**HIV/AIDS Prevention**														
Effectiveness And Cost Effectiveness Of Oral Pre-Exposure Prophylaxis In A Portfolio Of Prevention Programs For Injection Drug Users In Mixed HIV Epidemics. (4)	24489747	pre-exposure prophylaxis for hiv	388	863	2014	Ukraine	9	335.91, 559.85, 709.14, 1,045.04	73.6, 76.3, 78.1, 82.3, 82.7, 85, 89.6, 96	20 years	QALYs	3	3	health sector (4)
Clinical effectiveness and cost-effectiveness of HIV pre-exposure prophylaxis in men who have sex with men: risk calculators for real-world decision-making (16)	25285793	pre-exposure prophylaxis for hiv	3,379	360,448	2014	United States	3	10,489.03	28, 44, 92	Lifetime	QALYs	3	3	payer (2)
Modeling The Impact Of HIV Chemoprophylaxis Strategies Among Men Who Have Sex With Men In The United States: HIV Infections Prevented And Cost-Effectiveness. (27)	18753932	pre-exposure prophylaxis for hiv	16,987	62,498	2008	United States	8	10,493.35, 13,686.98, 14,143.21, 17,793.07, 23,267.87, 29,655.12, 41,973.41	50, 70	5 years	QALYs	3	3	payer (2)

Table S2. Summary of articles in the analysis sample														
**Cause and intervention type/Title**	**PubMedID**	**Intervention or keyword**	**Minimum ICER (2019 US$)**	**Maximum ICER (2019 US$)**	**Year**	**Countries**	**Num-ber of ratios**	**Cost (2019 US$)**	**Efficacy**	**Time horizon**	**DALY or QALY**	**Out-come discount rate**	**Costs discount rate**	**Pers-pective**
The Potential Impact Of Pre-Exposure Prophylaxis For HIV Prevention Among Men Who Have Sex With Men And Transwomen In Lima, Peru: A Mathematical Modelling Study. (38)	22508731	pre-exposure prophylaxis for hiv	1,227	2,176	2012	Peru	6	457.81, 654.02	92	2, 5, 10 years	DALYs	3	3	payer (2)
The Cost-Effectiveness Of Preexposure Prophylaxis For HIV Prevention In The United States In Men Who Have Sex With Men. (51)	23055836	pre-exposure prophylaxis for hiv	202,172	46,456	2012	United States	2	10,902.70	44	Lifetime	QALYs	3	3	payer (2)
Cost-Effectiveness Of Pre-Exposure Prophylaxis Targeted To High-Risk Serodiscordant Couples As A Bridge To Sustained Art Use In Kampala, Uganda. (135)	26198348	pre-exposure prophylaxis for hiv	925	925	2015	Uganda	1	279.52	75	Lifetime	DALYs	3	3	payer (2)
Implementation and Operational Research: A Cost-Effective, Clinically Actionable Strategy for Targeting HIV Preexposure Prophylaxis to High-Risk Men Who Have Sex With Men (94)	26977749	pre-exposure prophylaxis for hiv	1,994	192,901	2016	United States	0	7,949.36	58	20 years	QALYs	3	3	payer (2)

Table S2. Summary of articles in the analysis sample														
**Cause and intervention type/Title**	**PubMedID**	**Intervention or keyword**	**Minimum ICER (2019 US$)**	**Maximum ICER (2019 US$)**	**Year**	**Countries**	**Num-ber of ratios**	**Cost (2019 US$)**	**Efficacy**	**Time horizon**	**DALY or QALY**	**Out-come discount rate**	**Costs discount rate**	**Pers-pective**
A Cost-Effectiveness Analysis Of HIV Preexposure Prophylaxis For Men Who Have Sex With Men In Australia. (106)	24385445	pre-exposure prophylaxis for hiv	-1,807	12,627	2014	Australia	13	2,453.58, 4,907.15, 7,848.97, 9,814.31	81.3	10 years	QALYs	0, 3, 5	0, 3, 5	payer (2)
Where to deploy pre-exposure prophylaxis (PrEP) in sub-Saharan Africa (124)	23588668	pre-exposure prophylaxis for hiv	399	93,665	2013	Angola, Benin, Botswana, Burkina Faso, Burundi, Cameroon, Central African Republic, Chad, Congo, Cote D'Ivoire, Djibouti, Dr Congo, Equatorial Guinea, Eritrea, Ethiopia, Gabon, Ghana, Guinea, Guinea-Bissau, Kenya, Lesotho, Liberia, Malawi, Mali, Mauritania, Mozambique, Namibia, Niger, Nigeria, Rwanda, Senegal, Sierra Leone, Somalia, South Africa, Sudan, Swaziland,	42	121.54, 126.28, 140.44, 142.78, 145.79, 153.08, 154.18, 155.66, 157.72, 158.13, 159.47, 162.88, 171.65, 175.46, 181.29, 181.83, 183.18, 184.30, 185.0278411 186.3846418 187.663932 193.85, 195.32, 195.52, 196.35, 196.88, 197.90, 199.03, 204.16, 211.08, 214.73, 220.32, 224.21, 229.17, 246.07, 246.19, 250.24, 277.67,	68	5 years	DALYs	3	3	payer (2)

Table S2. Summary of articles in the analysis sample														
**Cause and intervention type/Title**	**PubMedID**	**Intervention or keyword**	**Minimum ICER (2019 US$)**	**Maximum ICER (2019 US$)**	**Year**	**Countries**	**Num-ber of ratios**	**Cost (2019 US$)**	**Efficacy**	**Time horizon**	**DALY or QALY**	**Out-come discount rate**	**Costs discount rate**	**Pers-pective**
						Tanzania, The Gambia, Togo, Uganda, Zambia, Zimbabwe		288.62, 306.57, 358.27, 420.02						
**Syphilis Diagnostics**														
Cost-Effectiveness Of HIV And Syphilis Antenatal Screening: A Modelling Study.(12)	26920867	POC test for antenatal syphilis screening	cost-saving	87	2016	Malawi	3	2.03	12.3	Lifetime	DALYs	3	3	limited societal (3)
Congenital syphilis: an economic evaluation of a prevention program in China (43)	19734825	Laboratory test for antenatal syphilis screening	215	215	2010	China	1	636748	30.9	3 Years	DALYs	3	3	limited societal (3)
Antenatal Syphilis Screening Using Point-Of-Care Testing In Sub-Saharan African Countries: A Cost-Effectiveness Analysis. (59)	24223524	antibiotics for syphilis, syphilis testing	2	68	2013	Angola, Benin, Botswana, Burkina Faso, Burundi, Cameroon, Cape Verde, Central African Republic, Chad, Comoros, Cote D'Ivoire, Djibouti, Dr Congo, Equatorial Guinea, Eritrea, Ethiopia, Gabon, Ghana, Guinea, Guinea-Bissau, Kenya, Lesotho, Liberia, Madagascar, Malawi, Mali,	258	3.63, 4.04, 4.11, 4.19, 4.4, 4.44, 4.48, 4.54, 4.55, 4.59, 4.69, 4.94, 5.05, 5.07, 5.22, 5.23, 5.27, 5.3, 5.32, 5.36, 5.4, 5.58, 5.61, 5.62, 5.63, 5.65, 5.67, 5.68, 5.69, 5.72, 5.73, 5.87, 6.07, 6.18, 6.31, 6.34, 6.42, 6.45, 6.56, 6.6, 6.88, 6.93, 7, 7.08, 7.09, 7.11, 7.17, 7.2, 7.32, 7.72, 7.89, 7.92, 7.99, 8.15, 8.18, 8.24, 8.29, 8.31, 8.32,	12.3	1 year	3, 6	3, 6	0	payer (2)

Table S2. Summary of articles in the analysis sample

Cause and intervention type/Title	PubMedID	Intervention or keyword	Minimum ICER (2019 US$)	Maximum ICER (2019 US$)	Year	Countries	Num-ber of ratios	Cost (2019 US$)	Efficacy	Time horizon	DALY or QALY	Out-come discount rate	Costs discount rate	Pers-pective
						Mauritania, Mozambique, Namibia, Niger, Nigeria, Rwanda, Senegal, Sierra Leone, South Africa, Sudan, Swaziland, Tanzania, The Gambia, Togo, Uganda, Zambia, Zimbabwe		8.38, 8.44, 8.72, 8.77, 8.78, 8.79, 8.83, 8.85, 8.9, 8.94, 8.95, 9.18, 9.49, 9.66, 9.91, 10.08, 10.31, 11.07, 11.26, 12.09, 12.49, 12.98, 16.12, 18.9						
Antenatal Syphilis Screening Using Point-Of-Care Testing In Low- And Middle-Income Countries In Asia And Latin America: A Cost-Effectiveness Analysis. (60)	26010366	POC test for antenatal syphilis screening	5	287	2015	Argentina, Bangladesh, Belize, Bolivia, Brazil, Cambodia, Chile, China, Colombia, Costa Rica, Cuba, Dominican Republic, Ecuador, El Salvador, Guatemala, Haiti, Honduras, India, Indonesia, Laos, Malaysia, Mexico, Myanmar, Nicaragua, Paraguay, Peru, Philippines, Thailand, Uruguay, Venezuela, Vietnam	31	2.82, 4.14, 4.34, 4.85, 4.89, 4.98, 4.99, 5.12, 5.13, 5.23, 5.35, 5.55, 5.60, 5.66, 5.77, 5.85, 5.96, 5.99, 6.09, 6.09, 6.15, 6.16, 6.23, 6.25, 6.34, 6.40, 6.51, 6.65, 6.86, 7.54, 8.05	12.3	Lifetime	DALYs	3	0	payer (2)

Table S2. Summary of articles in the analysis sample														
**Cause and intervention type/Title**	**PubMedID**	**Intervention or keyword**	**Minimum ICER (2019 US$)**	**Maximum ICER (2019 US$)**	**Year**	**Countries**	**Num-ber of ratios**	**Cost (2019 US$)**	**Efficacy**	**Time horizon**	**DALY or QALY**	**Out-come discount rate**	**Costs discount rate**	**Pers-pective**
Finding A Needle In The Haystack: The Costs And Cost-Effectiveness Of Syphilis Diagnosis And Treatment During Pregnancy To Prevent Congenital Syphilis In Kalomo District Of Zambia. (61)	25478877	POC test for antenatal syphilis screening	484	484	2014	Zambia	1	0.42	37.9	Lifetime	DALYs	3	3	payer (2)
Rapid Syphilis Testing Is Cost-Effective Even in Low-Prevalence Settings: The CISNE-PERU Experience (70)	26949941	POC test for antenatal syphilis screening, Laboratory test for antenatal syphilis screening	46	109	2016	Peru	2	missing	missing	Lifetime	DALYs	3	3	payer (2)
Cost-Effectiveness Of Rapid Syphilis Screening In Prenatal HIV Testing Programs In Haiti. (103)	17535105	POC test for antenatal syphilis screening	7	23	2007	Haiti	10	0.25	15.6	Lifetime	DALYs	3	0	limited societal (3)
Is antenatal syphilis screening still cost effective in sub-Saharan Africa (117)	14573832	Laboratory test for antenatal syphilis screening	70	196	2003	Tanzania	5	missing	52	Lifetime	DALYs	3	3	limited societal (3)
Antenatal syphilis screening in sub-Saharan Africa: lessons learned from Tanzania. (129)	16135202	Laboratory test for antenatal syphilis screening	10.56	10.56	2005	Tanzania	1	missing	96	Lifetime	DALYs	3	3	payer (2)

Table S2. Summary of articles in the analysis sample															
**Cause and intervention type/Title**	**PubMedID**	**Intervention or keyword**	**Minimum ICER (2019 US$)**	**Maximum ICER (2019 US$)**	**Year**	**Countries**	**Num-ber of ratios**	**Cost (2019 US$)**	**Efficacy**	**Time horizon**	**DALY or QALY**	**Out-come discount rate**	**Costs discount rate**	**Pers-pective**	
Modelling the cost-effectiveness of introducing rapid syphilis tests into an antenatal syphilis screening programme in Mwanza, Tanzania (135)	17215276	POC test for antenatal syphilis screening	12	17	2006	Tanzania	5	missing	missing	missing	DALYs	3	3	payer (2)	
**Tuberculosis Prevention**															
Targeted BCG Vaccination Against Severe Tuberculosis In Low-Prevalence Settings: Epidemiologic And Economic Assessment. (5)	19295437	tuberculosis vaccines	3,877	7,846	2009	Netherlands	4	11.14	73, 79	7 years	DALYs	0, 1.5	0	payer (2)	
Cost-Effectiveness Of Tuberculosis Screening And Isoniazid Treatment In The TB/HIV In Rio (Thrio) Study. (6)	25517809	prophylaxis for people without active tb, tuberculosis screening with tuberculin skin test	2,303	2,303	2014	Brazil	1	27.38	27	20 years	DALYs	3	3	payer (2)	
Targeted Screening And Treatment For Latent Tuberculosis Infection Using Quantiferon-TB Gold Is Cost-Effective In Mexico. (15)	19723375	prophylaxis for people without active tb, tuberculosis screening with tuberculin skin test	19	1,060	2009	Mexico	9	1.97	69	20 years	QALYs	2, 3, 10	2, 3, 10	payer (2)	
Cost, Affordability And Cost-Effectiveness Of Strategies To Control Tuberculosis In Countries With High HIV Prevalence. (26)	16343345	prophylaxis for people without active tb	205	1,585	2005	Kenya	4	159.40, 3,187.97, 6.375.93	none	10, 20 years	DALYs	3	3	payer (2)	

Table S2. Summary of articles in the analysis sample														
**Cause and intervention type/Title**	**PubMedID**	**Intervention or keyword**	**Minimum ICER (2019 US$)**	**Maximum ICER (2019 US$)**	**Year**	**Countries**	**Num-ber of ratios**	**Cost (2019 US$)**	**Efficacy**	**Time horizon**	**DALY or QALY**	**Out-come discount rate**	**Costs discount rate**	**Pers-pective**
Making wider use of the world's most widely used vaccine: Bacille Calmette-Guerin revaccination reconsidered. (33)	23904584	tuberculosis vaccines	66	89	2013	South Africa	3	0.77, 1.53	30, 50, 80	10 years	DALYs	3	3	payer (2)
Global Drug-Resistance Patterns And The Management Of Latent Tuberculosis Infection In Immigrants To The United States. (54)	12466510	prophylaxis for people without active tb, tuberculosis screening with tuberculin skin test	Cost-saving	23,875	2002	United States	40	472.14, 20.24, 565.60	70, 75	Lifetime	QALYs	3	3	societal (1)
Cost-Effectiveness Of Screening Compared To Case-Finding Approaches To Tuberculosis In Long-Term Care Facilities For The Elderly. (71)	10405865	prophylaxis for people without active tb, tuberculosis screening with tuberculin skin test	7,955	7,955	1999	Canada	1	32.94	65	15 years	QALYs	5	5	payer (2)
Short-Course Prophylaxis Against Tuberculosis In HIV-Infected Persons. A Decision And Cost-Effectiveness Analysis. (93)	9841583	prophylaxis for people without active tb	1,975	dominated	1998	United States	7	548.42, 288.64, 1,329.27, 692.74, 1350.54, 710.97, 706.41	49, 60, 67, 73, 75, 82, 83	Lifetime	QALYs	3	3	payer (2)
Cost-Utility Of Tuberculosis Prevention Among HIV-Infected Adults In Kampala, Uganda. (111)	17609049	prophylaxis for people without active tb, tuberculosis screening with tuberculin skin test	129	322	2007	Uganda	10	94.67, 135.38, 231.09	61.7, 76.6	Lifetime	QALYs	0, 3, 7	0, 3, 7	societal (1)

Table S2. Summary of articles in the analysis sample														
**Cause and intervention type/Title**	**PubMedID**	**Intervention or keyword**	**Minimum ICER (2019 US$)**	**Maximum ICER (2019 US$)**	**Year**	**Countries**	**Num-ber of ratios**	**Cost (2019 US$)**	**Efficacy**	**Time horizon**	**DALY or QALY**	**Out-come discount rate**	**Costs discount rate**	**Pers-pective**
Cost-Effectiveness Of LTBI Treatment For TB Contacts In British Columbia. (116)	18489519	prophylaxis for people without active tb, tuberculosis screening with tuberculin skin test	Cost-saving	dominated	2008	Canada	4	285.52	82.7	6 years	QALYs	3	3	societal (1)
**Tuberculosis Diagnostics**														
Cost-Effectiveness Analysis Of Introduction Of Rapid, Alternative Methods To Identify Multidrug-Resistant Tuberculosis In Middle-Income Countries. (1)	18636955	tuberculosis testing	139	443	2008	Peru	10	32.90, 35.97, 36.75, 38.00, 40.14, 49.48, 166.77	Sensitivity: 83.5, 84.3, 92.5, 92.8, 93.5, 93.6, 98 Specificity: 95.3, 96.8, 97.8, 98, 98.1, 98.5, 99, 99.2	Lifetime	DALYs	3	3	payer (2)
Cost-Effectiveness Of Novel Diagnostic Tools For The Diagnosis Of Tuberculosis. (29)	18713499	tuberculosis screening with smear microscopy	70	289	2008	Brazil, Kenya, South Africa	12	2.48, 3.57, 4.44, 13.33, 19.15, 20.6, 23.08, 23.84, 29.59, 33.16, 36.84 41.28	Sensitivity: 0, 70, 73 Specificity: 95, 97, 99	1 year	DALYs	3	3	payer (2)
Impact And Cost-Effectiveness Of Culture For Diagnosis Of Tuberculosis In HIV-Infected Brazilian Adults.(30)	19129940	tuberculosis screening with smear microscopy	1,019	1,423	2008	Brazil	3	5.46, 10.33, 10.64	Sensitivity: 71.9, 75 Specificity: 90, 94	Lifetime	DALYs	3	3	payer (2)
Serological Testing Versus Other Strategies For Diagnosis Of Active Tuberculosis In India: A Cost-Effectiveness Analysis. (32)	21857810	tuberculosis screening with smear microscopy	18	339	2011	India	4	3.45, 19.1, 22.56	Sensitivity: 68, 73, 76 Specificity: 87, 97, 99	Lifetime	DALYs	3	3	payer (2)

Table S2. Summary of articles in the analysis sample

Cause and intervention type/Title	PubMedID	Intervention or keyword	Minimum ICER (2019 US$)	Maximum ICER (2019 US$)	Year	Countries	Num-ber of ratios	Cost (2019 US$)	Efficacy	Time horizon	DALY or QALY	Out-come discount rate	Costs discount rate	Pers-pective
Cost-Utility Analysis Of Led Fluorescence Microscopy In The Diagnosis Of Pulmonary Tuberculosis In Indian Settings. (53)	25946362	tuberculosis screening with smear microscopy	14	14	2015	India	1	0.3	Sensitivity: 88 Specificity: 98	1 year	DALYs	0	0	payer (2)
Cost-Effectiveness Of Tobacco Cessation Support Combined With Tuberculosis Screening Among Contacts Who Smoke. (55)	26056114	tuberculosis screening with interferon gamma release assays, tuberculosis testing with tuberculin skin test	333	356	2015	Japan	3	11.77, 46.33	Sensitivity: 77, 84, 89 Specificity: 97, 98, 99	50 years	QALYs	3	3	payer (2)
Cost-effectiveness of interferon-γ release assay versus chest X-ray for tuberculosis screening of employees (56)	21839543	tuberculosis screening with interferon gamma release assays, tuberculosis testing	cost-saving	8,164,122	2011	Japan	2	34.76, 55.36	Sensitivity: 70, 84 Specificity: 60, 99	Lifetime	QALYs	3	3	payer (2)
Cost Effectiveness Of Interferon-Gamma Release Assay Versus Chest X-Ray For Tuberculosis Screening Of BCG-Vaccinated Elderly Populations. (57)	20799765	tuberculosis screening with interferon gamma release assays, tuberculosis testing	51,008	687,792	2010	Japan	2	36.18, 40.71	Sensitivity: 70, 81 Specificity: 60, 99	Lifetime	QALYs	3	3	societal (1)

Table S2. Summary of articles in the analysis sample														
**Cause and intervention type/Title**	**PubMedID**	**Intervention or keyword**	**Minimum ICER (2019 US$)**	**Maximum ICER (2019 US$)**	**Year**	**Countries**	**Num-ber of ratios**	**Cost (2019 US$)**	**Efficacy**	**Time horizon**	**DALY or QALY**	**Out-come discount rate**	**Costs discount rate**	**Pers-pective**
Priorities For Screening And Treatment Of Latent Tuberculosis Infection In The United States. (64)	21562129	tuberculosis screening with interferon gamma release assays, tuberculosis screening with tuberculin skin test	7,112	dominated	2011	United States	38	2.72, 34.76, 55.36, 59.39	Sensitivity: 70, 83, 84, 89 Specificity: 60, 92, 98, 99	Lifetime	QALYs	3	3	payer (2)
Costs and Consequences of Using Interferon-γ Release Assays for the Diagnosis of Active Tuberculosis in India (65)	25918999	tuberculosis screening with smear microscopy, xpert rapid tuberculosis test, tuberculosis screening with interferon gamma release assays	22	dominated	2014	India	3	3.04, 25.36, 30.44	Sensitivity: 53, 84, 94 Specificity: 52, 98, 97 Efficacy: 94	Lifetime	DALYs	3	3	payer (2)
Population Health Impact And Cost-Effectiveness Of Tuberculosis Diagnosis With Xpert Mtb/Rif: A Dynamic Simulation And Economic Evaluation. (74)	23185139	xpert rapid tuberculosis test	626	1,106	2012	Botswana, Lesotho, Namibia, South Africa, Swaziland	5	22.96, 23.72, 23.92, 24.43, 25.75	Sensitivity: 72.5 Specificity: 99.2	10 years	DALYs	3	3	payer (2)
Cost-Effectiveness And Resource Implications Of Aggressive Action On Tuberculosis In China, India, And South Africa: A Combined Analysis Of Nine Models. (75)	27720689	xpert rapid tuberculosis test	337	23,891	2016	China, India	2	16.77, 19.42	none	20 years	DALYs	3	3	limited societal (3)

Table S2. Summary of articles in the analysis sample															
**Cause and intervention type/Title**	**PubMedID**	**Intervention or keyword**	**Minimum ICER (2019 US$)**	**Maximum ICER (2019 US$)**	**Year**	**Countries**	**Num-ber of ratios**	**Cost (2019 US$)**	**Efficacy**	**Time horizon**	**DALY or QALY**	**Out-come discount rate**	**Costs discount rate**	**Pers-pective**
Cost-Effectiveness Of Improvements In Diagnosis And Treatment Accessibility For Tuberculosis Control In India. (115)	26260835	xpert rapid tuberculosis test	79	708	2015	India	4	18.57	Sensitivity: 90, 94 Specificity: 97, 98	Lifetime	QALYs	3	3	limited societal (3)
Modeling The Patient And Health System Impacts Of Alternative Xpert(R) Mtb/Rif Algorithms For The Diagnosis Of Pulmonary Tuberculosis In Addis Ababa, Ethiopia.(118)	28464797	xpert rapid tuberculosis test, tuberculosis screening with interferon gamma release assays	cost-saving	124	2017	Ethiopia	4	2.93, 4.39, 9.74	Sensitivity: 78.3, 89 Specificity: 99, 99.4	10 years	DALYs	3	3	payer (2)
Cost-Effectiveness Of Xpert Mtb/Rif For Tuberculosis Diagnosis In South Africa: A Real-World Cost Analysis And Economic Evaluation. (122)	28619229	xpert rapid tuberculosis test	cost-saving	cost-saving	2017	South Africa	1	23.68	Sensitivity: 88 Specificity: 89	0.5 years	DALYs	3	3	limited societal (3)
Rapid Diagnosis Of Tuberculosis With The Xpert Mtb/Rif Assay In High Burden Countries: A Cost-Effectiveness Analysis.(123)	22087078	xpert rapid tuberculosis test	39	113	2011	South Africa, Uganda, India	6	15.95, 17.25, 18.52, 20.08, 21.61, 22.69	Sensitivity: 77.8, 94.4 Specificity: 98.3, 99	Lifetime	DALYs	3	3	payer (2)
Microscopic Observation Drug-Susceptibility Assay Vs Xpert(R) Mtb/Rif For The Diagnosis Of Tuberculosis In A Rural African Setting: A Cost-Utility Analysis. (130)	28380276	xpert rapid tuberculosis test, tuberculosis screening with smear microscopy	84	3,876	2017	Mozambique	4	4.08, 4.83, 8.53, 9.28	Sensitivity: 61, 88, 88.2, 96 Specificity: 100	Lifetime	DALYs	3	3	payer (2)
**TB Treatment**														

Table S2. Summary of articles in the analysis sample														
**Cause and intervention type/Title**	**PubMedID**	**Intervention or keyword**	**Minimum ICER (2019 US$)**	**Maximum ICER (2019 US$)**	**Year**	**Countries**	**Num-ber of ratios**	**Cost (2019 US$)**	**Efficacy**	**Time horizon**	**DALY or QALY**	**Out-come discount rate**	**Costs discount rate**	**Pers-pective**
Cost, Affordability And Cost-Effectiveness Of Strategies To Control Tuberculosis In Countries With High HIV Prevalence. (26)	16343345	chemotherapy for DS-TB	205	1,585	2005	Kenya	4	159.40, 3,187.97, 6.375.93	none	10, 20 years	DALYs	3	3	payer (2)
Setting Priorities For The Health Care Sector In Zimbabwe Using Cost-Effectiveness Analysis And Estimates Of The Burden Of Disease. (40)	18662389	chemotherapy for DS-TB	267	267	2008	Zimbabwe	1	missing	none	Lifetime	DALYs	3	3	payer (2)
Cost-Effectiveness And Resource Implications Of Aggressive Action On Tuberculosis In China, India, And South Africa: A Combined Analysis Of Nine Models. (75)	27720689	chemotherapy for DS-TB	337	23,891	2016	China, India	2	16.77, 19.42	none	20 years	DALYs	3	3	limited societal (3)
Cost effectiveness of DOTS and non-DOTS strategies for smear-positive pulmonary tuberculosis in Beijing (132)	11351865	chemotherapy for DS-TB	237	237	2000	China	1	94	50	Lifetime	DALYs	3	3	payer (2)
Tuberculosis control priorities defined by using cost-effectiveness and burden of disease (133)	12244758	chemotherapy for DS-TB	13	13	2002	China	1	93	90	Lifetime	DALYs	3	3	payer (2)
**Malaria prevention**														

Table S2. Summary of articles in the analysis sample														
**Cause and intervention type/Title**	**PubMedID**	**Intervention or keyword**	**Minimum ICER (2019 US$)**	**Maximum ICER (2019 US$)**	**Year**	**Countries**	**Num-ber of ratios**	**Cost (2019 US$)**	**Efficacy**	**Time horizon**	**DALY or QALY**	**Out-come discount rate**	**Costs discount rate**	**Pers-pective**
Cost-Effectiveness Of Adding Bed Net Distribution For Malaria Prevention To Antenatal Services In Kinshasa, Democratic Republic Of The Congo. (9)	19706921	bed nets	15	19	2009	Congo	4	6.64, 8.83	28	1.75 years	DALYs	3	3	payer (2)
Cost-Effectiveness Of Malaria Preventive Treatment For HIV-Infected Pregnant Women In Sub-Saharan Africa. (17)	28985732	malaria intermittent preventive treatment for pregnant women	Cost-saving	72	2017	Ghana, Kenya, Malawi, Mozambique, Tanzania	15	0.51, 0.55, 0.61, 0.70, 0.71, 5.34, 5.78, 6.40, 7.35, 7.39	56, 74	9 months	DALYs	3	3	limited societal (3)
The Cost-Effectiveness Of Intermittent Preventive Treatment For Malaria In Infants In Sub-Saharan Africa. (25)	20559558	malaria intermittent preventive treatment	Cost-saving	dominated	2010	Gabon, Ghana, Kenya, Mozambique, Tanzania	15	0.31, 0.39, 0.47, 0.51, 0.52, 0.58, 2.10, 2.48, 2.86, 7.11	(-77.2, -52.5, -6.7, 10.5, 10.8, 17, 20.3, 22.2, 22.5, 22.6, 24.7, 24.9, 38.1, 50.2, 62.3	Lifetime	DALYs	3	3	limited societal (3)

Table S2. Summary of articles in the analysis sample														
**Cause and intervention type/Title**	**PubMedID**	**Intervention or keyword**	**Minimum ICER (2019 US$)**	**Maximum ICER (2019 US$)**	**Year**	**Countries**	**Num-ber of ratios**	**Cost (2019 US$)**	**Efficacy**	**Time horizon**	**DALY or QALY**	**Out-come discount rate**	**Costs discount rate**	**Pers-pective**
Country specific predictions of the cost-effectiveness of malaria vaccine RTS,S/AS01 in endemic Africa (36)	27890400	malaria vaccines	79	13,863	2017	Angola, Benin, Botswana, Burkina Faso, Burundi, Cameroon, Central African Republic, Chad, Comoros, Congo, Cote d'Ivoire, Djibouti, DR Congo, Equatorial Guinea, Eritrea, Ethiopia, Gabon, Ghana, Guinea, Guinea-Bissau, Kenya, Liberia, Madagascar, Malawi, Mali, Mauritania, Mozambique, Namibia, Niger, Nigeria, Rwanda, Sao Tome and Principe, Senegal, Sierra Leone, Somalia, South Sudan, Sudan, Tanzania, The Gambia, Togo, Uganda, Zambia, Zimbabwe	43	4.86, 10.29, 10.32, 10.63, 11.04, 11.89, 12.30, 12.34, 12.44, 12.75, 12.77, 12.83, 12.95, 13.23, 13.33, 13.62, 13.74, 13.75, 13.97, 14.06, 14.27, 14.37, 14.49, 14.71, 14.72, 14.75, 14.84, 14.99, 15.13, 15.34, 15.42, 15.62, 16.11, 16.29, 16.40, 16.57, 17.50, 17.59, 17.61, 17.74, 18.25, 25.86, 26.18	91.1	10 years	DALYs	3	3	limited societal (3)

Table S2. Summary of articles in the analysis sample														
**Cause and intervention type/Title**	**PubMedID**	**Intervention or keyword**	**Minimum ICER (2019 US$)**	**Maximum ICER (2019 US$)**	**Year**	**Countries**	**Num-ber of ratios**	**Cost (2019 US$)**	**Efficacy**	**Time horizon**	**DALY or QALY**	**Out-come discount rate**	**Costs discount rate**	**Pers-pective**
Setting Priorities For The Health Care Sector In Zimbabwe Using Cost-Effectiveness Analysis And Estimates Of The Burden Of Disease. (40)	18662389	indoor residual spraying	267	267	2008	Zimbabwe	1	missing	none	Lifetime	DALYs	3	3	payer (2)
Cost-Effectiveness Of Social Marketing Of Insecticide-Treated Nets For Malaria Control In The United Republic Of Tanzania. (41)	12764493	bed nets	54	83	2003	Tanzania	2	5.46, 7.27	19, 27	5 years	DALYs	3	3	limited societal (3)
Cost-Effectiveness Of Adding Indoor Residual Spraying To Case Management In Afghan Refugee Settlements In Northwest Pakistan During A Prolonged Malaria Epidemic. (46)	29059179	indoor residual spraying	305	686	2017	Pakistan	6	0.5, 0.72, 0.76, 1.09, 1.26, 1.81	70.3, 70.4	5 years	DALYs	3	3	limited societal (3)
Cost-Effectiveness Of Malaria Intermittent Preventive Treatment In Infants (Ipti) In Mozambique And The United Republic Of Tanzania. (48)	19274364	malaria intermittent preventive treatment	5	11	2009	Mozambique, Tanzania	4	5.7, 9.45	22.2, 30, 63.2	Lifetime	DALYs	3	3	payer (2)

Table S2. Summary of articles in the analysis sample														
**Cause and intervention type/Title**	**PubMedID**	**Intervention or keyword**	**Minimum ICER (2019 US$)**	**Maximum ICER (2019 US$)**	**Year**	**Countries**	**Num-ber of ratios**	**Cost (2019 US$)**	**Efficacy**	**Time horizon**	**DALY or QALY**	**Out-come discount rate**	**Costs discount rate**	**Pers-pective**
The Economic Value Of Long-Lasting Insecticidal Nets And Indoor Residual Spraying Implementation In Mozambique. (62)	28719286	bed nets, indoor residual spraying	Cost-saving	dominated	2017	Mozambique	18	1.92, 4.35	(-46.2, -19.5, -12.4, 3, 4.2, 10.6, 12.3, 12.4, 17, 29.2, 33.6, 43.1, 51.9, 53.6, 68.9, 73.7, 109.4, 133	3 years	DALYs	3	3	limited societal (3)
Intermittent Preventive Treatment Of Malaria In Pregnancy: The Incremental Cost-Effectiveness Of A New Delivery System In Uganda. (73)	18513767	malaria intermittent preventive treatment for pregnant women	0	18	2008	Uganda	7	0.52	27	0.5 years	DALYs	0, 3, 6, 10	3, 6, 10, 15	limited societal (3)
Cost-Effectiveness Analysis Of Insecticide-Treated Net Distribution As Part Of The Togo Integrated Child Health Campaign. (80)	18445255	bed nets	23	23	2008	Togo	1	8.25	17	Lifetime	DALYs	3	5	payer (2)
Public Health Impact And Cost-Effectiveness Of The Rts,S/As01 Malaria Vaccine: A Systematic Comparison Of Predictions From Four Mathematical Models. (88)	26549466	malaria vaccines	38	167	2016	Ethiopia	6	6.52, 8.69, 16.29, 21.72, 32.58, 43.44	19.4, 83.5	15 years	DALYs	0	3	payer (2)

Table S2. Summary of articles in the analysis sample															
**Cause and intervention type/Title**	**PubMedID**	**Intervention or keyword**	**Minimum ICER (2019 US$)**	**Maximum ICER (2019 US$)**	**Year**	**Countries**	**Num-ber of ratios**	**Cost (2019 US$)**	**Efficacy**	**Time horizon**	**DALY or QALY**	**Out-come discount rate**	**Costs discount rate**	**Pers-pective**
Cost-Effectiveness Analysis Of Vaccinating Children In Malawi With Rts,S Vaccines In Comparison With Long-Lasting Insecticide-Treated Nets. (108)	24564883	bed nets, malaria vaccines	Cost-saving	461	2014	Malawi	29	2.56, 2.70, 7.69, 12.81, 17.94, 23.06, 28.19, 33.31, 38.44	50, 53.9	1, 5, 10, 20, 30, 40, 50, 60 years, Lifetime	DALYs	0, 3, 5, 6	0, 3, 5, 6	limited societal (3), health sector (4)
Modeling The Cost Effectiveness Of Malaria Control Interventions In The Highlands Of Western Kenya. (114)	25290939	indoor residual spraying, malaria treatment, bed nets	5	11	2014	Kenya	4	15.28, 19.55	96, 98, 99	5 years	DALYs	0	0	limited societal (3)

Table S2 references

1. Acuna-Villaorduna C, Vassall A, Henostroza G, Seas C, Guerra H, Vasquez L, et al. Cost-effectiveness analysis of introduction of rapid, alternative methods to identify multidrug-resistant tuberculosis in middle-income countries. Clin Infect Dis. 2008 Aug 15;47(4):487–95.

2. Aldridge RW, Iglesias D, Cáceres CF, Miranda JJ. Determining a cost effective intervention response to HIV/AIDS in Peru. BMC Public Health. 2009 Sep 18;9:352.

3. Alistar SS, Owens DK, Brandeau ML. Effectiveness and cost effectiveness of expanding harm reduction and antiretroviral therapy in a mixed HIV epidemic: a modeling analysis for Ukraine. PLoS Med. 2011 Mar;8(3):e1000423.

4. Alistar SS, Owens DK, Brandeau ML. Effectiveness and cost effectiveness of oral pre-exposure prophylaxis in a portfolio of prevention programs for injection drug users in mixed HIV epidemics. PLoS One. 2014;9(1):e86584.

5. Altes HK, Dijkstra F, Lugnèr A, Cobelens F, Wallinga J. Targeted BCG vaccination against severe tuberculosis in low-prevalence settings: epidemiologic and economic assessment. Epidemiology. 2009 Jul;20(4):562–8.

6. Azadi M, Bishai DM, Dowdy DW, Moulton LH, Cavalcante S, Saraceni V, et al. Cost-effectiveness of tuberculosis screening and isoniazid treatment in the TB/HIV in Rio (THRio) Study. Int J Tuberc Lung Dis. 2014 Dec;18(12):1443–8.

7. Badri M, Cleary S, Maartens G, Pitt J, Bekker LG, Orrell C, et al. When to initiate highly active antiretroviral therapy in sub-Saharan Africa? A South African cost-effectiveness study. Antivir Ther. 2006;11(1):63–72.

8. Bayoumi AM, Barnett PG, Joyce VR, Griffin SC, Sun H, Bansback NJ, et al. Cost-effectiveness of newer antiretroviral drugs in treatment-experienced patients with multidrug-resistant HIV disease. J Acquir Immune Defic Syndr. 2013 Dec 1;64(4):382–91.

9. Becker-Dreps SI, Biddle AK, Pettifor A, Musuamba G, Imbie DN, Meshnick S, et al. Cost-effectiveness of adding bed net distribution for malaria prevention to antenatal services in Kinshasa, Democratic Republic of the Congo. Am J Trop Med Hyg. 2009 Sep;81(3):496–502.

10. Bendavid E, Grant P, Talbot A, Owens DK, Zolopa A. Cost-effectiveness of antiretroviral regimens in the World Health Organization's treatment guidelines: a South African analysis. AIDS. 2011 Jan 14;25(2):211–20.

11. Bishai D, Colchero A, Durack DT. The cost effectiveness of antiretroviral treatment strategies in resource-limited settings. AIDS. 2007 Jun 19;21(10):1333–40.

12. Bristow CC, Larson E, Anderson LJ, Klausner JD. Cost-effectiveness of HIV and syphilis antenatal screening: a modelling study. Sex Transm Infect. 2016 Aug;92(5):340–6.

13. Broder MS, Chang EY, Bentley TGK, Juday T, Uy J. Cost effectiveness of atazanavir-ritonavir versus lopinavir-ritonavir in treatment-naïve human immunodeficiency virus-infected patients in the United States. J Med Econ. 2011;14(2):167–78.

14. Brogan AJ, Smets E, Mauskopf JA, Manuel SAL, Adriaenssen I. Cost effectiveness of darunavir/ritonavir combination antiretroviral therapy for treatment-naive adults with HIV-1 infection in Canada. Pharmacoeconomics. 2014 Sep;32(9):903–17.

15. Burgos JL, Kahn JG, Strathdee SA, Valencia-Mendoza A, Bautista-Arredondo S, Laniado-Laborin R, et al. Targeted screening and treatment for latent tuberculosis infection using QuantiFERON-TB Gold is cost-effective in Mexico. Int J Tuberc Lung Dis. 2009 Aug;13(8):962–8.

16. Chen A, Dowdy DW. Clinical effectiveness and cost-effectiveness of HIV pre-exposure prophylaxis in men who have sex with men: risk calculators for real-world decision-making. PLoS One. 2014;9(10):e108742.

17. Choi SE, Brandeau ML, Bendavid E. Cost-effectiveness of malaria preventive treatment for HIV-infected pregnant women in sub-Saharan Africa. Malar J. 2017 Oct 6;16(1):403.

18. Cipriano LE, Zaric GS, Holodniy M, Bendavid E, Owens DK, Brandeau ML. Cost effectiveness of screening strategies for early identification of HIV and HCV infection in injection drug users. PLoS One. 2012;7(9):e45176.

19. Cleary SM, McIntyre D, Boulle AM. The cost-effectiveness of antiretroviral treatment in Khayelitsha, South Africa--a primary data analysis. Cost Eff Resour Alloc. 2006 Dec 6;4:20.

20. Cleary SM, McIntyre D, Boulle AM. Assessing efficiency and costs of scaling up HIV treatment. AIDS. 2008 Jul;22 Suppl 1:S35-42.

21. Coco A. The cost-effectiveness of expanded testing for primary HIV infection. Ann Fam Med. 2005 Oct;3(5):391–9.

22. Colombo GL, Colangeli V, Di Biagio A, Di Matteo S, Viscoli C, Viale P. Cost-effectiveness analysis of initial HIV treatment under Italian guidelines. Clinicoecon Outcomes Res. 2011;3:197–205.

23. Colombo GL, Di Matteo S, Antinori A, Medaglia M, Murachelli S, Rizzardini G. Economic evaluation of initial antiretroviral therapy for HIV-infected patients: an update of Italian guidelines. Clinicoecon Outcomes Res. 2013 Oct 3;5:489–96.

24. Colombo GL, Di Matteo S, Maggiolo F. Antiretroviral therapy in HIV-infected patients: a proposal to assess the economic value of the single-tablet regimen. Clinicoecon Outcomes Res. 2013;5:59–68.

25. Conteh L, Sicuri E, Manzi F, Hutton G, Obonyo B, Tediosi F, et al. The cost-effectiveness of intermittent preventive treatment for malaria in infants in Sub-Saharan Africa. PLoS One. 2010 Jun 15;5(6):e10313.

26. Currie CSM, Floyd K, Williams BG, Dye C. Cost, affordability and cost-effectiveness of strategies to control tuberculosis in countries with high HIV prevalence. BMC Public Health. 2005 Dec 12;5:130.

27. Desai K, Sansom SL, Ackers ML, Stewart SR, Hall HI, Hu DJ, et al. Modeling the impact of HIV chemoprophylaxis strategies among men who have sex with men in the United States: HIV infections prevented and cost-effectiveness. AIDS. 2008 Sep 12;22(14):1829–39.

28. Despiégel N, Anger D, Martin M, Monga N, Cui Q, Rocchi A, et al. Cost-Effectiveness of Dolutegravir in HIV-1 Treatment-Naive and Treatment-Experienced Patients in Canada. Infect Dis Ther. 2015 Sep;4(3):337–53.

29. Dowdy DW, O'Brien MA, Bishai D. Cost-effectiveness of novel diagnostic tools for the diagnosis of tuberculosis. Int J Tuberc Lung Dis. 2008 Sep;12(9):1021–9.

30. Dowdy DW, Lourenço MC, Cavalcante SC, Saraceni V, King B, Golub JE, et al. Impact and cost-effectiveness of culture for diagnosis of tuberculosis in HIV-infected Brazilian adults. PLoS One. 2008;3(12):e4057.

31. Dowdy DW, Rodriguez RM, Hare CB, Kaplan B. Cost-effectiveness of targeted human immunodeficiency virus screening in an urban emergency department. Acad Emerg Med. 2011 Jul;18(7):745–53.

32. Dowdy DW, Steingart KR, Pai M. Serological testing versus other strategies for diagnosis of active tuberculosis in India: a cost-effectiveness analysis. PLoS Med. 2011 Aug;8(8):e1001074.

33. Dye C. Making wider use of the world's most widely used vaccine: Bacille Calmette-Guerin revaccination reconsidered. J R Soc Interface. 2013 Oct 6;10(87):20130365.

34. Eaton JW, Menzies NA, Stover J, Cambiano V, Chindelevitch L, Cori A, et al. Health benefits, costs, and cost-effectiveness of earlier eligibility for adult antiretroviral therapy and expanded treatment coverage: a combined analysis of 12 mathematical models. Lancet Glob Health. 2013 Dec 10;2(1):23–34.

35. Freedberg KA, Losina E, Weinstein MC, Paltiel AD, Cohen CJ, Seage GR, et al. The cost effectiveness of combination antiretroviral therapy for HIV disease. N Engl J Med. 2001 Mar 15;344(11):824–31.

36. Galactionova K, Tediosi F, Camponovo F, Smith TA, Gething PW, Penny MA. Country specific predictions of the cost-effectiveness of malaria vaccine RTS,S/AS01 in endemic Africa. Vaccine. 2017 Jan 3;35(1):53–60.

37. Goldie SJ, Paltiel AD, Weinstein MC, Losina E, Seage GR, Kimmel AD, et al. Projecting the cost-effectiveness of adherence interventions in persons with human immunodeficiency virus infection. Am J Med. 2003 Dec 1;115(8):632–41.

38. Gomez GB, Borquez A, Caceres CF, Segura ER, Grant RM, Garnett GP, et al. The potential impact of pre-exposure prophylaxis for HIV prevention among men who have sex with men and transwomen in Lima, Peru: a mathematical modelling study. PLoS Med. 2012;9(10):e1001323.

39. Granich R, Kahn JG, Bennett R, Holmes CB, Garg N, Serenata C, et al. Expanding ART for treatment and prevention of HIV in South Africa: estimated cost and cost-effectiveness 2011-2050. PLoS One. 2012;7(2):e30216.

40. Hansen KS, Chapman G. Setting priorities for the health care sector in Zimbabwe using cost-effectiveness analysis and estimates of the burden of disease. Cost Eff Resour Alloc. 2008 Jul 28;6:14.

41. Hanson K, Kikumbih N, Armstrong Schellenberg J, Mponda H, Nathan R, Lake S, et al. Cost-effectiveness of social marketing of insecticide-treated nets for malaria control in the United Republic of Tanzania. Bull World Health Organ. 2003;81(4):269–76.

42. Heffernan A, Barber E, Thomas R, Fraser C, Pickles M, Cori A. Impact and Cost-Effectiveness of Point-Of-Care CD4 Testing on the HIV Epidemic in South Africa. PLoS One. 2016;11(7):e0158303.

43. Hong FC, Liu JB, Feng TJ, Liu XL, Pan P, Zhou H, et al. Congenital syphilis: an economic evaluation of a prevention program in China. Sex Transm Dis. 2010 Jan;37(1):26–31.

44. Hornberger J, Green J, Wintfeld N, Cavassini M, Rockstroh J, Giuliani G, et al. Cost-effectiveness of enfuvirtide for treatment-experienced patients with HIV in Italy. HIV Clin Trials. 2005 Apr;6(2):92–102.

45. Hornberger J, Kilby JM, Wintfeld N, Green J. Cost-effectiveness of enfuvirtide in HIV therapy for treatment-experienced patients in the United States. AIDS Res Hum Retroviruses. 2006 Mar;22(3):240–7.

46. Howard N, Guinness L, Rowland M, Durrani N, Hansen KS. Cost-effectiveness of adding indoor residual spraying to case management in Afghan refugee settlements in Northwest Pakistan during a prolonged malaria epidemic. PLoS Negl Trop Dis. 2017 Oct;11(10):e0005935.

47. Hutchinson AB, Patel P, Sansom SL, Farnham PG, Sullivan TJ, Bennett B, et al. Cost-effectiveness of pooled nucleic acid amplification testing for acute HIV infection after third-generation HIV antibody screening and rapid testing in the United States: a comparison of three public health settings. PLoS Med. 2010 Sep 28;7(9):e1000342.

48. Hutton G, Schellenberg D, Tediosi F, Macete E, Kahigwa E, Sigauque B, et al. Cost-effectiveness of malaria intermittent preventive treatment in infants (IPTi) in Mozambique and the United Republic of Tanzania. Bull World Health Organ. 2009 Feb;87(2):123–9.

49. Jouquet G, Bygrave H, Kranzer K, Ford N, Gadot L, Lee J, et al. Cost and cost-effectiveness of switching from d4T or AZT to a TDF-based first-line regimen in a resource-limited setting in rural Lesotho. J Acquir Immune Defic Syndr. 2011 Nov 1;58(3):e68-74.

50. Juusola JL, Brandeau ML, Long EF, Owens DK, Bendavid E. The cost-effectiveness of symptom-based testing and routine screening for acute HIV infection in men who have sex with men in the USA. AIDS. 2011 Sep 10;25(14):1779–87.

51. Juusola JL, Brandeau ML, Owens DK, Bendavid E. The cost-effectiveness of preexposure prophylaxis for HIV prevention in the United States in men who have sex with men. Ann Intern Med. 2012 Apr 17;156(8):541–50.

52. Kahn JG, Marseille E, Moore D, Bunnell R, Were W, Degerman R, et al. CD4 cell count and viral load monitoring in patients undergoing antiretroviral therapy in Uganda: cost effectiveness study. BMJ. 2011 Nov 9;343:d6884.

53. Kelly V, Sagili KD, Satyanarayana S, Reza LW, Chadha SS, Wilson NC. Cost-utility analysis of LED fluorescence microscopy in the diagnosis of pulmonary tuberculosis in Indian settings. Int J Tuberc Lung Dis. 2015 Jun;19(6):696–701.

54. Khan K, Muennig P, Behta M, Zivin JG. Global drug-resistance patterns and the management of latent tuberculosis infection in immigrants to the United States. N Engl J Med. 2002 Dec 5;347(23):1850–9.

55. Kowada A. Cost-effectiveness of tobacco cessation support combined with tuberculosis screening among contacts who smoke. Int J Tuberc Lung Dis. 2015 Jul;19(7):857–63.

56. Kowada A. Cost-effectiveness of interferon-γ release assay versus chest X-ray for tuberculosis screening of employees. Am J Infect Control. 2011 Dec;39(10):e67-72.

57. Kowada A, Deshpande GA, Takahashi O, Shimbo T, Fukui T. Cost effectiveness of interferon-gamma release assay versus chest X-ray for tuberculosis screening of BCG-vaccinated elderly populations. Mol Diagn Ther. 2010 Aug 1;14(4):229–36.

58. Kuznik A, Lamorde M, Hermans S, Castelnuovo B, Auerbach B, Semeere A, et al. Evaluating the cost-effectiveness of combination antiretroviral therapy for the prevention of mother-to-child transmission of HIV in Uganda. Bull World Health Organ. 2012 Aug 1;90(8):595–603.

59. Kuznik A, Lamorde M, Nyabigambo A, Manabe YC. Antenatal syphilis screening using point-of-care testing in Sub-Saharan African countries: a cost-effectiveness analysis. PLoS Med. 2013 Nov;10(11):e1001545.

60. Kuznik A, Muhumuza C, Komakech H, Marques EMR, Lamorde M. Antenatal syphilis screening using point-of-care testing in low- and middle-income countries in Asia and Latin America: a cost-effectiveness analysis. PLoS One. 2015;10(5):e0127379.

61. Larson BA, Lembela-Bwalya D, Bonawitz R, Hammond EE, Thea DM, Herlihy J. Finding a needle in the haystack: the costs and cost-effectiveness of syphilis diagnosis and treatment during pregnancy to prevent congenital syphilis in Kalomo District of Zambia. PLoS One. 2014;9(12):e113868.

62. Lee BY, Bartsch SM, Stone NTB, Zhang S, Brown ST, Chatterjee C, et al. The Economic Value of Long-Lasting Insecticidal Nets and Indoor Residual Spraying Implementation in Mozambique. Am J Trop Med Hyg. 2017 Jun;96(6):1430–40.

63. Li J, Gilmour S, Zhang H, Koyanagi A, Shibuya K. The epidemiological impact and cost-effectiveness of HIV testing, antiretroviral treatment and harm reduction programs. AIDS. 2012 Oct 23;26(16):2069–78.

64. Linas BP, Wong AY, Freedberg KA, Horsburgh CR. Priorities for screening and treatment of latent tuberculosis infection in the United States. Am J Respir Crit Care Med. 2011 Sep 1;184(5):590–601.

65. Little KM, Pai M, Dowdy DW. Costs and Consequences of Using Interferon-γ Release Assays for the Diagnosis of Active Tuberculosis in India. PLoS One. 2014;10(4):e0124525.

66. Long EF. HIV screening via fourth-generation immunoassay or nucleic acid amplification test in the United States: a cost-effectiveness analysis. PLoS One. 2011;6(11):e27625.

67. Long EF, Brandeau ML, Owens DK. The cost-effectiveness and population outcomes of expanded HIV screening and antiretroviral treatment in the United States. Ann Intern Med. 2010 Dec 21;153(12):778–89.

68. Long EF, Mandalia R, Mandalia S, Alistar SS, Beck EJ, Brandeau ML. Expanded HIV testing in low-prevalence, high-income countries: a cost-effectiveness analysis for the United Kingdom. PLoS One. 2014;9(4):e95735.

69. Long EF, Stavert RR. Portfolios of biomedical HIV interventions in South Africa: a cost-effectiveness analysis. J Gen Intern Med. 2013 Oct;28(10):1294–301.

70. Mallma P, Garcia P, Carcamo C, Torres-Rueda S, Peeling R, Mabey D, et al. Rapid Syphilis Testing Is Cost-Effective Even in Low-Prevalence Settings: The CISNE-PERU Experience. PLoS One. 2016;11(3):e0149568.

71. Marchand R, Tousignant P, Chang H. Cost-effectiveness of screening compared to case-finding approaches to tuberculosis in long-term care facilities for the elderly. Int J Epidemiol. 1999 Jun;28(3):563–70.

72. Marseille E, Saba J, Muyingo S, Kahn JG. The costs and benefits of private sector provision of treatment to HIV-infected employees in Kampala, Uganda. AIDS. 2006 Apr 4;20(6):907–14.

73. Mbonye AK, Hansen KS, Bygbjerg IC, Magnussen P. Intermittent preventive treatment of malaria in pregnancy: the incremental cost-effectiveness of a new delivery system in Uganda. Trans R Soc Trop Med Hyg. 2008 Jul;102(7):685–93.

74. Menzies NA, Cohen T, Lin HH, Murray M, Salomon JA. Population health impact and cost-effectiveness of tuberculosis diagnosis with Xpert MTB/RIF: a dynamic simulation and economic evaluation. PLoS Med. 2012;9(11):e1001347.

75. Menzies NA, Gomez GB, Bozzani F, Chatterjee S, Foster N, Baena IG, et al. Cost-effectiveness and resource implications of aggressive action on tuberculosis in China, India, and South Africa: a combined analysis of nine models. Lancet Glob Health. 2016 Nov;4(11):e816–26.

76. Moeremans K, Annemans L, Löthgren M, Allegri G, Wyffels V, Hemmet L, et al. Cost Effectiveness of Darunavir/Ritonavir 600/100mg bid in Protease Inhibitor-Experienced, HIV-1-Infected Adults in Belgium, Italy, Sweden and the UK. Pharmacoeconomics [Internet]. 2010 Dec 1 [cited 2023 Jun 26];28(1):107–28. Available from: https://doi.org/10.2165/11587480-000000000-00000

77. Moeremans K, Hemmett L, Hjelmgren J, Allegri G, Smets E. Cost Effectiveness of Darunavir/Ritonavir 600/100mg bid in Treatment-Experienced, Lopinavir-Naive, Protease Inhibitor-Resistant, HIV-Infected Adults in Belgium, Italy, Sweden and the UK. Pharmacoeconomics [Internet]. 2010 Dec 1 [cited 2023 Jun 26];28(1):147–67. Available from: https://doi.org/10.2165/11587500-000000000-00000

78. Moreno S, González J, Lekander I, Martí B, Oyagüez I, Sánchez-de la Rosa R, et al. Cost-effectiveness of optimized background therapy plus maraviroc for previously treated patients with R5 HIV-1 infection from the perspective of the Spanish health care system. Clin Ther. 2010 Dec;32(13):2232–45.

79. Morris BL, Scott CA, Wilkin TJ, Sax PE, Gulick RM, Freedberg KA, et al. Cost-effectiveness of adding an agent that improves immune responses to initial antiretroviral therapy (ART) in HIV-infected patients: guidance for drug development. HIV Clin Trials. 2012 Feb;13(1):1–10.

80. Mueller DH, Wiseman V, Bakusa D, Morgah K, Daré A, Tchamdja P. Cost-effectiveness analysis of insecticide-treated net distribution as part of the Togo Integrated Child Health Campaign. Malar J. 2008 Apr 29;7:73.

81. Nichols BE, Sigaloff KCE, Kityo C, Hamers RL, Baltussen R, Bertagnolio S, et al. Increasing the use of second-line therapy is a cost-effective approach to prevent the spread of drug-resistant HIV: a mathematical modelling study. J Int AIDS Soc. 2014;17:19164.

82. Oddershede L, Walker S, Stöhr W, Dunn DT, Arenas-Pinto A, Paton NI, et al. Cost Effectiveness of Protease Inhibitor Monotherapy Versus Standard Triple Therapy in the Long-Term Management of HIV Patients: Analysis Using Evidence from the PIVOT Trial. Pharmacoeconomics. 2016 Aug;34(8):795–804.

83. Ono S, Kurotaki T, Nakasone T, Honda M, Boon-Long J, Sawanpanyalert P, et al. Cost-effectiveness analysis of antiretroviral drug treatment and HIV-1 vaccination in Thailand. Jpn J Infect Dis. 2006 Jun;59(3):168–73.

84. Owusu-Edusei K, Tao G, Gift TL, Wang A, Wang L, Tun Y, et al. Cost-effectiveness of integrated routine offering of prenatal HIV and syphilis screening in China. Sex Transm Dis. 2014 Feb;41(2):103–10.

85. Paltiel AD, Walensky RP, Schackman BR, Seage GR, Mercincavage LM, Weinstein MC, et al. Expanded HIV screening in the United States: effect on clinical outcomes, HIV transmission, and costs. Ann Intern Med. 2006 Dec 5;145(11):797–806.

86. Paltiel AD, Weinstein MC, Kimmel AD, Seage GR, Losina E, Zhang H, et al. Expanded screening for HIV in the United States--an analysis of cost-effectiveness. N Engl J Med. 2005 Feb 10;352(6):586–95.

87. Peng S, Tafazzoli A, Dorman E, Rosenblatt L, Villasis-Keever A, Sorensen S. Cost-effectiveness of DTG + ABC/3TC versus EFV/TDF/FTC for first-line treatment of HIV-1 in the United States. J Med Econ. 2015;18(10):763–76.

88. Penny MA, Verity R, Bever CA, Sauboin C, Galactionova K, Flasche S, et al. Public health impact and cost-effectiveness of the RTS,S/AS01 malaria vaccine: a systematic comparison of predictions from four mathematical models. Lancet. 2016 Jan 23;387(10016):367–75.

89. Pham QD, Wilson DP, Kerr CC, Shattock AJ, Do HM, Duong AT, et al. Estimating the Cost-Effectiveness of HIV Prevention Programmes in Vietnam, 2006-2010: A Modelling Study. PLoS One. 2015;10(7):e0133171.

90. Phillips KA, Fernyak S. The cost-effectiveness of expanded HIV counselling and testing in primary care settings: a first look. AIDS. 2000 Sep 29;14(14):2159–69.

91. Pialoux G, Marcelin AG, Despiégel N, Espinas C, Cawston H, Finkielsztejn L, et al. Cost-Effectiveness of Dolutegravir in HIV-1 Treatment-Experienced (TE) Patients in France. PLoS One. 2015;10(12):e0145885.

92. Prabhu VS, Farnham PG, Hutchinson AB, Soorapanth S, Heffelfinger JD, Golden MR, et al. Cost-effectiveness of HIV screening in STD clinics, emergency departments, and inpatient units: a model-based analysis. PLoS One. 2011;6(5):e19936.

93. Rose DN. Short-course prophylaxis against tuberculosis in HIV-infected persons. A decision and cost-effectiveness analysis. Ann Intern Med. 1998 Nov 15;129(10):779–86.

94. Ross EL, Cinti SK, Hutton DW. Implementation and Operational Research: A Cost-Effective, Clinically Actionable Strategy for Targeting HIV Preexposure Prophylaxis to High-Risk Men Who Have Sex With Men. J Acquir Immune Defic Syndr. 2016 Jul 1;72(3):e61-67.

95. Ross EL, Weinstein MC, Schackman BR, Sax PE, Paltiel AD, Walensky RP, et al. The clinical role and cost-effectiveness of long-acting antiretroviral therapy. Clin Infect Dis. 2015 Apr 1;60(7):1102–10.

96. Sanders GD, Bayoumi AM, Holodniy M, Owens DK. Cost-effectiveness of HIV screening in patients older than 55 years of age. Ann Intern Med. 2008 Jun 17;148(12):889–903.

97. Sanders GD, Bayoumi AM, Sundaram V, Bilir SP, Neukermans CP, Rydzak CE, et al. Cost-effectiveness of screening for HIV in the era of highly active antiretroviral therapy. N Engl J Med. 2005 Feb 10;352(6):570–85.

98. Sax PE, Losina E, Weinstein MC, Paltiel AD, Goldie SJ, Muccio TM, et al. Cost-effectiveness of enfuvirtide in treatment-experienced patients with advanced HIV disease. J Acquir Immune Defic Syndr. 2005 May 1;39(1):69–77.

99. Sax PE, Sloan CE, Schackman BR, Grant PM, Rong J, Zolopa AR, et al. Early antiretroviral therapy for patients with acute aids-related opportunistic infections: a cost-effectiveness analysis of ACTG A5164. HIV Clin Trials. 2010 Oct;11(5):248–59.

100. Schackman BR, Goldie SJ, Weinstein MC, Losina E, Zhang H, Freedberg KA. Cost-effectiveness of earlier initiation of antiretroviral therapy for uninsured HIV-infected adults. Am J Public Health. 2001 Sep;91(9):1456–63.

101. Schackman BR, Freedberg KA, Weinstein MC, Sax PE, Losina E, Zhang H, et al. Cost-effectiveness implications of the timing of antiretroviral therapy in HIV-infected adults. Arch Intern Med. 2002 Nov 25;162(21):2478–86.

102. Schackman BR, Haas DW, Park SS, Li XC, Freedberg KA. Cost-effectiveness of CYP2B6 genotyping to optimize efavirenz dosing in HIV clinical practice. Pharmacogenomics. 2015 Dec;16(18):2007–18.

103. Schackman BR, Neukermans CP, Fontain SNN, Nolte C, Joseph P, Pape JW, et al. Cost-effectiveness of rapid syphilis screening in prenatal HIV testing programs in Haiti. PLoS Med. 2007 May;4(5):e183.

104. Schackman BR, Scott CA, Sax PE, Losina E, Wilkin TJ, McKinnon JE, et al. Potential risks and benefits of HIV treatment simplification: a simulation model of a proposed clinical trial. Clin Infect Dis. 2007 Oct 15;45(8):1062–70.

105. Schackman BR, Scott CA, Walensky RP, Losina E, Freedberg KA, Sax PE. The cost-effectiveness of HLA-B*5701 genetic screening to guide initial antiretroviral therapy for HIV. AIDS. 2008 Oct 1;22(15):2025–33.

106. Schneider K, Gray RT, Wilson DP. A cost-effectiveness analysis of HIV preexposure prophylaxis for men who have sex with men in Australia. Clin Infect Dis. 2014 Apr;58(7):1027–34.

107. Schneider K, Nwizu C, Kaplan R, Anderson J, Wilson DP, Emery S, et al. The potential cost and benefits of raltegravir in simplified second-line therapy among HIV infected patients in Nigeria and South Africa. PLoS One. 2013;8(2):e54435.

108. Seo MK, Baker P, Ngo KNL. Cost-effectiveness analysis of vaccinating children in Malawi with RTS,S vaccines in comparison with long-lasting insecticide-treated nets. Malar J. 2014 Feb 24;13:66.

109. Shah M, Dowdy D, Joloba M, Ssengooba W, Manabe YC, Ellner J, et al. Cost-effectiveness of novel algorithms for rapid diagnosis of tuberculosis in HIV-infected individuals in Uganda. AIDS. 2013 Nov 28;27(18):2883–92.

110. Shah M, Risher K, Berry SA, Dowdy DW. The Epidemiologic and Economic Impact of Improving HIV Testing, Linkage, and Retention in Care in the United States. Clin Infect Dis. 2016 Jan 15;62(2):220–9.

111. Shrestha RK, Mugisha B, Bunnell R, Mermin J, Odeke R, Madra P, et al. Cost-utility of tuberculosis prevention among HIV-infected adults in Kampala, Uganda. Int J Tuberc Lung Dis. 2007 Jul;11(7):747–54.

112. Simpson KN, Baran RW, Kirbach SE, Dietz B. Economics of switching to second-line antiretroviral therapy with lopinavir/ritonavir in Africa: estimates based on DART trial results and costs for Uganda and Kenya. Value Health. 2011 Dec;14(8):1048–54.

113. Simpson KN, Pei PP, Möller J, Baran RW, Dietz B, Woodward W, et al. Lopinavir/ritonavir versus darunavir plus ritonavir for HIV infection: a cost-effectiveness analysis for the United States. Pharmacoeconomics. 2013 May;31(5):427–44.

114. Stuckey EM, Stevenson J, Galactionova K, Baidjoe AY, Bousema T, Odongo W, et al. Modeling the cost effectiveness of malaria control interventions in the highlands of western Kenya. PLoS One. 2014;9(10):e107700.

115. Suen SC, Bendavid E, Goldhaber-Fiebert JD. Cost-effectiveness of improvements in diagnosis and treatment accessibility for tuberculosis control in India. Int J Tuberc Lung Dis. 2015 Sep;19(9):1115–24, i–xv.

116. Tan MC, Marra CA, Sadatsafavi M, Marra F, Morán-Mendoza O, Moadebi S, et al. Cost-effectiveness of LTBI treatment for TB contacts in British Columbia. Value Health. 2008 Oct;11(5):842–52.

117. Terris-Prestholt F, Watson-Jones D, Mugeye K, Kumaranayake L, Ndeki L, Weiss H, et al. Is antenatal syphilis screening still cost effective in sub-Saharan Africa. Sex Transm Infect. 2003 Oct;79(5):375–81.

118. Tesfaye A, Fiseha D, Assefa D, Klinkenberg E, Balanco S, Langley I. Modeling the patient and health system impacts of alternative xpert® MTB/RIF algorithms for the diagnosis of pulmonary tuberculosis in Addis Ababa, Ethiopia. BMC Infect Dis. 2017 May 2;17(1):318.

119. Tole SP, Sanders GD, Bayoumi AM, Galvin CM, Vinichenko TN, Brandeau ML, et al. Cost-effectiveness of voluntary HIV screening in Russia. Int J STD AIDS. 2009 Jan;20(1):46–51.

120. Tran DA, Wilson DP, Shakeshaft A, Ngo AD, Reyes J, Doran C, et al. Cost-effectiveness of antiretroviral therapy expansion strategies in Vietnam. AIDS Patient Care STDS. 2014 Jul;28(7):365–71.

121. Tweya H, Keiser O, Haas AD, Tenthani L, Phiri S, Egger M, et al. Comparative cost-effectiveness of Option B+ for prevention of mother-to-child transmission of HIV in Malawi. AIDS. 2016 Mar 27;30(6):953–62.

122. Vassall A, Siapka M, Foster N, Cunnama L, Ramma L, Fielding K, et al. Cost-effectiveness of Xpert MTB/RIF for tuberculosis diagnosis in South Africa: a real-world cost analysis and economic evaluation. Lancet Glob Health. 2017 Jul;5(7):e710–9.

123. Vassall A, van Kampen S, Sohn H, Michael JS, John KR, den Boon S, et al. Rapid diagnosis of tuberculosis with the Xpert MTB/RIF assay in high burden countries: a cost-effectiveness analysis. PLoS Med. 2011 Nov;8(11):e1001120.

124. Verguet S, Stalcup M, Walsh JA. Where to deploy pre-exposure prophylaxis (PrEP) in sub-Saharan Africa? Sex Transm Infect. 2013 Dec;89(8):628–34.

125. Vickerman P, Peeling RW, Terris-Prestholt F, Changalucha J, Mabey D, Watson-Jones D, et al. Modelling the cost-effectiveness of introducing rapid syphilis tests into an antenatal syphilis screening programme in Mwanza, Tanzania. Sexually Transmitted Infections [Internet]. 2006 Dec [cited 2023 Jun 26];82(Suppl 5):v38. Available from: https://www.ncbi.nlm.nih.gov/pmc/articles/PMC2563909/

126. Walensky RP, Morris BL, Reichmann WM, Paltiel AD, Arbelaez C, Donnell-Fink L, et al. Resource utilization and cost-effectiveness of counselor- vs. provider-based rapid point-of-care HIV screening in the emergency department. PLoS One. 2011;6(10):e25575.

127. Walensky RP, Sax PE, Nakamura YM, Weinstein MC, Pei PP, Freedberg KA, et al. Economic savings versus health losses: the cost-effectiveness of generic antiretroviral therapy in the United States. Ann Intern Med. 2013 Jan 15;158(2):84–92.

128. Walensky RP, Wood R, Fofana MO, Martinson NA, Losina E, April MD, et al. The clinical impact and cost-effectiveness of routine, voluntary HIV screening in South Africa. J Acquir Immune Defic Syndr. 2011 Jan 1;56(1):26–35.

129. Watson-Jones D, Oliff M, Terris-Prestholt F, Changalucha J, Gumodoka B, Mayaud P, et al. Antenatal syphilis screening in sub-Saharan Africa: lessons learned from Tanzania. Tropical Medicine & International Health [Internet]. 2005 [cited 2023 Jun 26];10(9):934–43. Available from: https://onlinelibrary.wiley.com/doi/abs/10.1111/j.1365-3156.2005.01473.x

130. Wikman-Jorgensen PE, Llenas-García J, Pérez-Porcuna TM, Hobbins M, Ehmer J, Mussa MA, et al. Microscopic observation drug-susceptibility assay vs. Xpert® MTB/RIF for the diagnosis of tuberculosis in a rural African setting: a cost-utility analysis. Trop Med Int Health. 2017 Jun;22(6):734–43.

131. Winskill P, Slater HC, Griffin JT, Ghani AC, Walker PGT. The US President's Malaria Initiative, Plasmodium falciparum transmission and mortality: A modelling study. PLoS Med. 2017 Nov;14(11):e1002448.

132. Xu Q, Jin SG, Zhang LX. Cost effectiveness of DOTS and non-DOTS strategies for smear-positive pulmonary tuberculosis in Beijing. Biomed Environ Sci. 2000 Dec;13(4):307–13.

133. Xu Q, Wu ZL, Jin SG, Zhang LX. Tuberculosis control priorities defined by using cost-effectiveness and burden of disease. Biomed Environ Sci. 2002 Jun;15(2):172–6.

134. Yazdanpanah Y, Sloan CE, Charlois-Ou C, Le Vu S, Semaille C, Costagliola D, et al. Routine HIV screening in France: clinical impact and cost-effectiveness. PLoS One. 2010 Oct 1;5(10):e13132.

135. Ying R, Sharma M, Heffron R, Celum CL, Baeten JM, Katabira E, et al. Cost-effectiveness of pre-exposure prophylaxis targeted to high-risk serodiscordant couples as a bridge to sustained ART use in Kampala, Uganda. J Int AIDS Soc. 2015;18(4 Suppl 3):20013.

136. Zhuang X, Peng P, Sun H, Chu M, Jiang S, Jiang L, et al. Scaling Up Human Immunodeficiency Virus Screening and Antiretroviral Therapy Among Men Who Have Sex With Men to Achieve the 90-90-90 Targets in China. Sex Transm Dis. 2018 May;45(5):343–9.

Section 3. Intervention Taxonomy[1]:

3.1: Intervention Taxonomy Overview

In order to retrieve relevant interventions from the Tufts University Cost Effectiveness Analysis (CEA) Registry, and the Global Health Cost Effectiveness (GHCEA) Registry we had to devise a searchable standard. These registries include a phrase known as the "intervention phrase" which describes the content of each entry. This variable is unstandardized. For example, phrases related to antiretroviral therapy include:

- tenofovir/emtricitabine + efavirenz (tdf/ftc + efv)
- antiretroviral therapy
- abacavir sulfate (abc) treatment regime
- newly diagnosed people who inject drugs in the early stages of hiv receive prompt, sustained antiretroviral therapy (art)
- antiretroviral drugs

We reviewed existing intervention taxonomies to adapt for this analysis, including the World Health Organization International Classification of Health Interventions (ICHI), the ITAX taxonomy focused on capturing intervention features such as adaptability and mechanism of action [2], and Cochrane's EPOC taxonomy [3]. After reviewing these existing taxonomies, we thought that they would not allow us to classify health interventions with sufficient detail to distinguish between drivers of ICERs. Therefore, we leveraged the IHME Global Health Data Exchange (GHDx) platform to develop an intervention taxonomy to standardize and categorize each intervention represented in the Tufts registries.

We used Open Refine to group similar interventions together using its natural language processing and text filtering tools [4]. Previous iterations of this taxonomy utilized a component known as 'intervention details'. The details represented attributes such as vaccine type, drug dosage, or target population. However that process has since been retired, and the intervention details are no longer utilized in the taxonomy. We created separate taxonomies for intervention keywords. Intervention keywords represented unique intervention components. For each term in the taxonomy, we added synonyms from each intervention description that were unique to that term to the taxonomy. These synonyms were pulled from the Tufts "Intervention Phrase" or "Intervention Paragraph" variables. In the case of the GHCEA registry the "Blurblong" column was also used to identify key variable components. When these variables were either too broad to add as synonyms or too vague to determine which keyword to assign them, we returned to the articles to extract additional details on the interventions and updated the intervention descriptions accordingly. We ran a SQL query to export all of the taxonomy terms and synonyms, and created a Python dictionary where the taxonomy terms were keys and the synonyms were values. We used this dictionary to map all of the ratios in the Tufts registries to one or more intervention keywords.

3.2. Guiding principles for intervention taxonomy

When building out the intervention taxonomy, we developed several guiding principles, including the following:

1. For pharmaceutical interventions, we chose to use the drug class as the most granular level of detail.

2. We excluded information about the specific target population (e.g. age, sex, risk group) and delivery platform from the intervention keywords, as we had processes for capturing these details in additional variables.

3. The majority of intervention keywords in our taxonomy will have one of two parents: (1) DALY gross interventions, or (2) QALY gross interventions. There are two exceptions to this structure, which necessitate adding a lower level to the taxonomy. These include the following:
    a. Polyhierarchy structure: For interventions that were shared across causes, we used a polyhierarchical approach wherein the same keyword had multiple parents (Figure S3.1). This was limited to chemotherapies and immunotherapies where the same drug classes were used to treat multiple causes, as well as imaging techniques that were used to screen for a variety of conditions. For example, "alkylating antineoplastic agents" had several parent keywords, such as "chemotherapy for biliary tract cancer" and "chemotherapy for B-cell lymphoma."
    b. Variations on the level of detail provided for a given intervention: We added lower levels to the taxonomy when interventions were described using varying levels of detail in order to retain information on these distinctions (Figure S3.2). In these instances, every ratio would be mapped to the parent, and only those ratios with more detail would also be mapped to the lower level. For example, every ratio that involves antiplatelet therapy is mapped to the intervention keyword "antiplatelet therapy." In addition, when articles specify the drugs they are evaluating, their corresponding ratios are also mapped to the drug classes for these drugs (e.g. "antiplatelet therapy with COX inhibitors).

4. If a particular intervention is used across multiple causes and the specific intervention did not differ by cause, we did not include the cause name in the intervention keyword. On the other hand, when the same category of intervention is across causes, yet the cause has a strong influence on the specifics of the intervention (e.g. dosage, timing, frequency), we included the cause name in the intervention keyword.

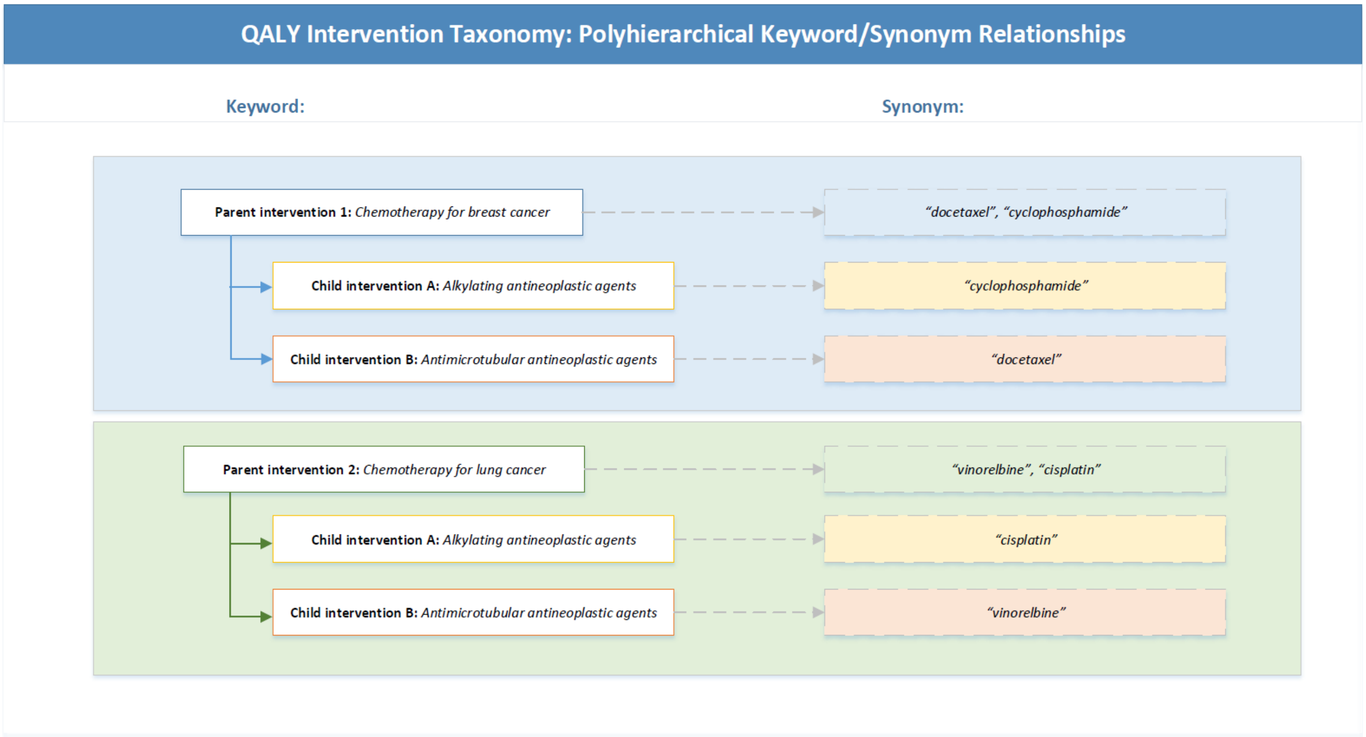

Figure S3.1. Polyhierarchical relationships in intervention taxonomy. Each parent intervention keyword has child keywords that represent specific drug classes for each type of chemotherapy. These drug classes are used to treat multiple cancer types, and thus have multiple parents. For example, "Alkylating antineoplastic agents" is a child with parents "Chemotherapy for breast cancer" and "Chemotherapy for lung cancer." Therefore, interventions descriptions in the Tufts registries that contain the word "cisplatin" will be mapped to "Child intervention A" and "Parent intervention 2." This allows us to meta-regress cost-effectiveness ratios for all chemotherapy interventions for a particular type of cancer (e.g. chemotherapy for breast cancer), or to meta-regress cost-effectiveness ratios across cancer types for a particular drug class (e.g. alkylating antineoplastic agents).

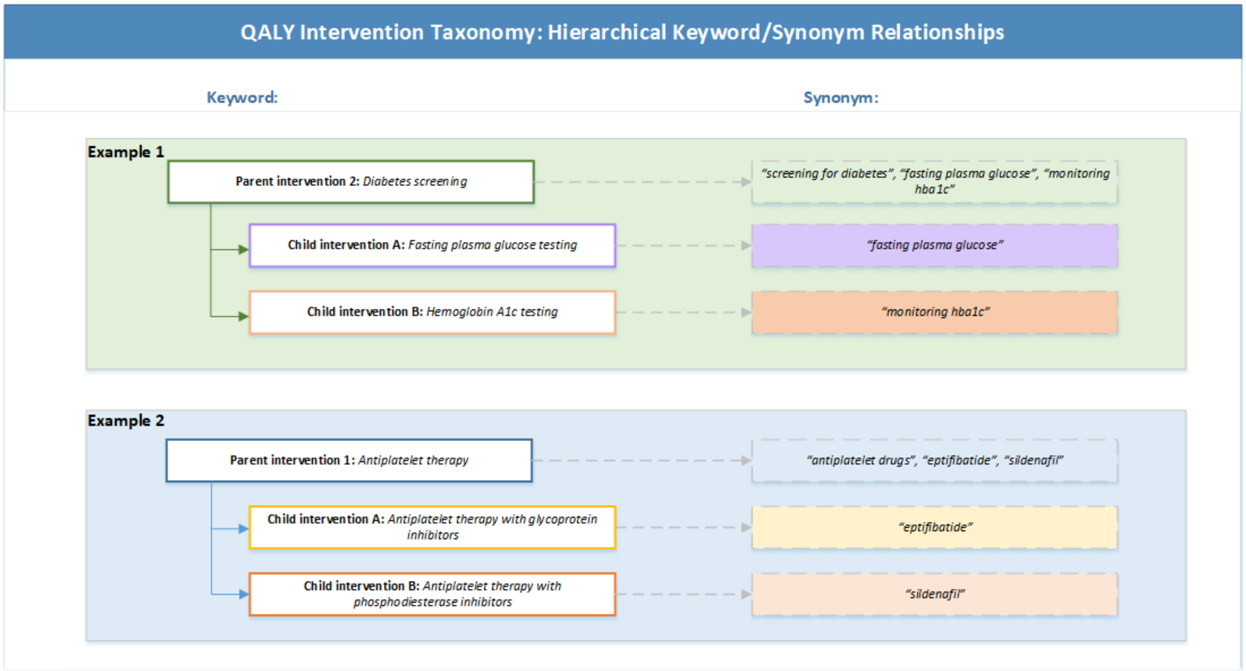

Figure S2. Hierarchical relationships in intervention taxonomy to accommodate varying levels of intervention detail. Articles describe interventions with varying levels of detail. In order to retain as much data as possible, we create parent-child relationships that allow for different degrees of intervention specificity. For example, when articles only describe interventions as "diabetes screening" without reference to the specific type of screening, we include synonyms for the parent "Diabetes screening" only. Interventions that include fasting plasma glucose testing or hemoglobin A1c testing are mapped to the parent and the more specific child. This allows us to run a regression for the parent intervention (e.g. all diabetes screening interventions), or to filter down to specific types of screening (e.g. all fasting plasma glucose testing interventions).

Section 3 References

1. Rosettie KL, Joffe JN, Sparks GW, Aravkin A, Chen S, Compton K, et al. Cost-effectiveness of HPV vaccination in 195 countries: A meta-regression analysis. *PLOS One* 2021;**16**: e0260808.

2. Schulz R, Czaja SJ, McKay JR, Ory MG, Belle SH. Intervention Taxonomy (ITAX): Describing Essential Features of Interventions (HMC). *Am J Health Behav* 2010; **34**: 811–21.

3. EPOC Taxonomy. https://epoc.cochrane.org/epoc-taxonomy (accessed Jun 14, 2023).

4. openrefine.github.com. http://openrefine.org/ (accessed Jun 14, 2023).

Section 4. Data extractions and mapping [1]

4.1. Overview

We performed data extraction, and mapping tasks to create a data file for the meta-regression analysis. When entries were missing for nine key variables in the registries, we extracted them from the articles: 1) age and 2) sex of the target population, 3) comparator and 4) intervention descriptions, 5) cost discount rate, 6) discount rate for health outcome, 7) study time horizon, 8) diseases, injuries or risks targeted by the intervention, and 9) study locations. Additional variable definitions and mapping tasks are described below.

4.2. Null comparator

The Tufts registries include a categorical variable for the comparator for each ICER, including none, placebo, standard of care, or other. They also included a text variable that described the comparator in more detail. When these data were missing, we returned to the articles to extract the comparator description and map the comparator to one of the aforementioned four categories.

We defined the null comparator as no intervention, standard of care, or placebo. When possible, we re-calculated ICERs compared to “other” such that they were compared to “null”. For articles that did not report sufficient data to re-calculate ICERs relative to no intervention, we re-calculated them compared to either “placebo” or “standard of care”. We used data in the Tufts registries on total or per person costs and total or per person health benefits. For ratios without these data reported in the registries, we went back to the articles to extract the necessary data to re-calculate the ICERs relative to the null comparator (i.e. “none”, “placebo”, or “standard of care”). We removed ratios that could not be re-calculated to the null comparator, as well as ratios that were compared to “other” if we had another ratio for the same intervention compared to the null.

4.3. Time horizon

For ratios missing the time horizon variables in the Tufts registries, we returned to the papers to extract the time horizon magnitude and units (weeks, months, years, lifetime). For our analysis, we created a dichotomous variable: lifetime vs. not lifetime. If the article did not clearly state the time horizon, but the authors explicitly cite including mortality as an outcome of their analysis, we coded the corresponding ratios as having a lifetime time horizon. The time horizon was not missing for any ratios thus no ratios were omitted due to time horizon.

4.4. Discount rate

The Tufts registries include variables that capture the discount rate for costs and health outcomes (QALYs or DALYs). For those ratios missing discount rate data, we returned to the papers to extract these values. When articles referenced standard methods, we extracted the discount rates cited in those methods (e.g. WHO-CHOICE). We removed ratios from our analysis if the discount rates were not clearly stated in the article.

4.5. Age

The Tufts registries include several categorical variables for age. When these data were available, we mapped the Tufts age variables to GBD age variables. For those ratios with missing age information in the Tufts data (52% of all ratios), we used the abstract, target population description, or returned to the articles to extract the age information. If there was no description of the age in the article, we defaulted to the GBD age groups used for modeling the cause(s) targeted by that particular intervention. We made the following additional assumptions when the age was not explicitly stated in the article: (1) policies, laws, and infrastructural interventions affect all ages; (2) interventions targeting pregnant women were mapped to GBD’s definition for reproductive-aged women (ages 15-49 years); (3) interventions targeting adults were assumed target individuals ages 15 and older. Finally, we adjusted all age-mapping such that the age variable captured the beneficiary target population in instances

where the target population and beneficiary population differed. These primarily including prevention of mother to child transmission interventions where the mother is targeted and the child benefits.

4.6. Sex

For ratios missing sex information, we created a Python dictionary to map interventions to male, female, or both. This dictionary included causes that are sex-specific (e.g. prostate cancer, ovarian cancer), as well as other sex-specific interventions (e.g. male circumcision, tubal ligation). For ratios that were not mapped using the Python dictionary approach, we used the Tufts' target population descriptions to map ratios to sex.

4.7. Causes

In the Tufts registries, articles are mapped to one or more causes or risk factors, yet neither registry used the most recent GBD 2017 cause hierarchy. We needed each ratio to be mapped to one or more GBD 2017 causes, etiologies, or impairments in order to leverage the most recent GBD data. We first developed a mapping of Tufts causes to GBD 2017 causes. For articles with only one cause, we mapped all of the ratios in that article to that cause. For articles with multiple ratios, we first created a Python dictionary with cause-specific keywords to map each ratio to the most detailed cause(s) possible. Next, for those we could not map with the dictionary approach, we used Doctor Evidence software (https://drevidence.com/). We uploaded article titles to Doctor Evidence, which used a mapping algorithm to map keywords in those titles to ICD9 and ICD10 codes. We then used the ICD9 and ICD10 to GBD cause mapping algorithm to map these articles to GBD causes. Because these two processes (dictionary mapping and DRE) were automated, we validated them by reviewing the causes each ratio was mapped to and made changes to an erroneous mappings. For the remaining ratios that we were unable to map through the processes outlined above, we used the abstract or returned to the articles to map each of these ratios to GBD causes. Finally, we removed any ratios that could not be mapped to GBD causes.

4.8. Locations

The Tufts registries included a text variable for the target population country of the intervention. We mapped this variable with the GBD location hierarchy. For locations that did not readily merge with GBD locations, we manually mapped them. For ratios that were mapped to regions or super-regions rather than countries, such as a series of WHO Choice articles, we returned to the articles to extract country-level ratios if they were present [1]. If there were no country-level results, we excluded these articles.

4.9. Intervention Type

To increase the sample size for the estimates, we grouped the interventions keywords into 'Prevention', 'Treatment', and 'Diagnostic'. The Tufts registries include PrimaryPrevention, PreventionSecondary, PreventionTertiary, and Diagnostic at the RatioID level and these columns were used as starting points to flag each intervention type. Tufts registries defines these terms according to the Encyclopedia of Public Health, as explained in the CEA Registry User Manual.[2] Prevention Primary is "aimed at altering behavioral and/or clinical risk factors *before* a targeted condition has evolved among persons who are asymptomatic." Prevention Secondary is "aimed at identifying persons who may have begun, but have not shown apparent clinical signs and symptoms of the illness (for screening purpose) or have not formally diagnosed (for diagnostic purpose)." Prevention Tertiary is "aimed at preventing progression of a targeted disease among persons with clinical diagnosis to alleviate symptoms and reduce complications after initial clinical diagnosis." The completion of these columns allowed for ratios to have more than one intervention type, and multiple intervention types were flagged at the beginning of the process.

During the extraction process, the data intervention type was reclassified based on the measures of outcomes and efficacy. An intervention was classified as prevention when the outcome focused on reducing incidence or transmission in the population. It was classified as treatment when the outcome focused on improving the condition of an individual. It was classified as diagnostic when the outcome was the sensitivity and/or specificity

testing. Ratios with multiple types in the Tufts registries were reclassified to a single type that represented the incremental intervention relative to a standard of care comparator.

4.10. Efficacy

Although the source of efficacy is reported in the Tufts registries, the numerical values are not. We extracted this information for ratios classified as prevention or treatment. Several columns were created to report the values consistently and accurately. The ‘efficacy_intervention’ variable reports the efficacy measure such as culture conversions, mortality reduced, disease incidence reduced, etc. The ‘efficacy_comparator’ variable is the same information for the comparator. ‘Efficacy_length’ reports the length of effect if relevant. ‘Efficacy’ is the effect size used in the CEA model. When it is not reported in the article and the comparator is placebo or no intervention, efficacy is calculated as one minus the relative risk. When comparator is standard of care, the effect size is calculated as the difference between the intervention and comparator.

4.11. Sensitivity and Specificity

The sensitivity and specificity are not reported in the Tufts registries and we extracted this information for ratios classified as diagnostic. The variables ‘sensitivity’, ‘specificity, ‘sensitivity_describe’, and ‘specificity_describe’ were extracted. The descriptor variables describe the specific test associated with the numerical values.

4.12: Commodity Costs

The Tufts registries report the categories of cost that are included in the CEA such as direct medical care cost vs non-health care costs, but the categories are not at the level of detail we require, and numerical values of costs within these categories are not reported. We extracted the commodity costs for each ratio, with distinct variables for each intervention type, as described for key variables below. All extracted cost data were converted to 2019 United States dollars.

Treatment costs

- tr_unit_cost – Reports commodity costs for the intervention. This was generally the drug cost, excluding cost such as hospital stays. For acute causes, this variable is the cost per episode of disease (i.e. full treatment of TB or malaria). For chronic causes, this variable is the drug cost per person per year.
- tr_cost_describe - Description column used by the data extraction analysts to explain the costs associated with the treatment, the length of treatment, factors considered when calculating treatment costs, and other information. This variable contains details about all other treatment cost columns.
- Log per year or full intervention cost (2019 USD) - For acute causes, this variable is the cost per episode of disease. For chronic causes, it is the drug cost per person per year. When the comparator is the standard of care, the variable is calculated as the difference in unit cost between the intervention and comparator.

Prevention costs

- pr_unit_cost - This is the unit cost of a drug or vaccine or the annual cost of an intervention.
- pr_number_units - This captures the number of doses or times the intervention is delivered for full protection. For vaccines this variable is the number of doses. For an annual intervention, the number of units is one.
- pr_total cost – This variable is the product of the pr_unit_cost and the pr_number of units
- pr_protection_duration – This is the duration of benefits or protection from the intervention in years.
- pr_protection_year_cost – We calculate the cost per year of protection as the pr_total_cost divided by the pr_protection duration.
- Log per year or full intervention cost (2019 USD). For prevention interventions, this variable is the pr_protection_year_cost. When the comparator is the standard of care, it is the difference in pr_protection_year_cost between the intervention and comparator.

Diagnostic costs

- d_unit_cost – the cost of a diagnostic test. It could include a chemical and any specific requirements for the testing, such as decontamination lab prep work. This does not include the cost of personnel such as a laboratory technician.
- d_number_units - the number of times the tests are performed to complete the diagnoses.
- Log per year or full intervention cost (2019 USD) – The total cost of each screening or testing intervention calculated as the unit cost multiplied by the number of units.

Section 4 References

1. Rosettie KL, Joffe JN, Sparks GW, Aravkin A, Chen S, Compton K, et al. Cost-effectiveness of HPV vaccination in 195 countries: A meta-regression analysis. *PLOS One* 2021; **16**: e0260808.

2. Center for the Evaluation of Value and Risk in Health. The Cost-Effectiveness Analysis (CEA) Registry User Manual. Boston: Institute for Clinical Research and Health Policy Studies, Tufts Medical Center https://cear.tuftsmedicalcenter.org/storage/resources/CEA%20Registry%20User%20Manual%202022-1.docx (accessed Jun 14, 2023).

Section 5. Background on five-stage meta-regression analysis

5.1. Introduction

This section provides background on the statistical model and fitting procedure used to obtain the estimated ICERs, posterior uncertainty estimates, and other intermediate quantities as previously reported. [1, 2] For technical details on model fitting please see [3]. Our meta-regression analysis used the same five stages as outlined in [1, 2] with modifications for the unique features of the datasets used for these analyses. Here we reproduce much of the information in the supplementary material for Rosettie et al [1] on the five stage model. Section 6 reports the modifications and results for each of the cause-type groups that we analyzed.

Briefly, in stage 1, a crosswalk analyses of covariates could be conducted, as described in Section 5.2, when the cause-type data for a group included sensitivity analysis. The crosswalk analyses leverage the sensitivity analyses reported in the studies from the Tufts registries to calculate priors for key covariates in the models. In stage 2, we estimate a nonlinear response curve for log-GDP per capita, as detailed in Section 5.3. This analysis uses splines to represent the curve, nonlinear observation models for relative risks, a robust statistical approach for outlier detection, and a spline ensemble to make the model less sensitive to model specification. Grouping of observations with random effects is ignored in this stage of the analysis. In stage 3, we use the nonlinear response curve estimated in stage 2 to select potential bias covariates using a generalized Lasso approach for linear mixed effects models, detailed in Section 5.4. In stage 4, as described in Section 5.5, we use 10-fold cross-validation to select the standard deviation of a Gaussian prior to apply to all covariates other than those analyzed in the crosswalk analysis (stage 1). In stage 5, we include covariates that were detected in the third stage, along with the nonlinear response, and consider a mixed effects model with a random intercept as described in Section 5.6.

5.2. Crosswalk analyses of sensitivity analysis covariates

Univariate sensitivity analyses are used to estimate the effect of a variable on the ICER with all other variables held constant by definition. We analyzed the difference in log-ICERs between sensitivity analyses and the corresponding reference analyses using models which we refer to as crosswalks. Including the results of these models as priors in subsequent steps of the analysis decreases omitted variable bias by giving more influence to pairs of ICERs which we know differ in no unmeasured variables. It also stabilizes estimates in the presence of multicollinearity. See the correlation matrices for each cause-type group in Section 7 below.

We conducted crosswalk analyses covariates that had a sufficient number of sensitivity analyses for us to control for study-specific variables not included in our model by using comparisons of sensitivity analyses and reference results. For each of these covariates, we paired each sensitivity analysis with another ICER from the same study and location and which differed only in that covariate. For these covariates, we fit separate models of the form

$$\boldsymbol{y_{i,sens} - y_{i,ref} = \alpha_c \times (x_{c_i,sens} - x_{c_i,ref}) + \epsilon_i} \quad (1)$$

Where x_c denotes the crosswalk covariate of interest, which is the only covariate that differs between the sensitivity (sens) and reference (ref) analyses, $y_{i,sens}$ and $y_{i,ref}$ represent the log-ICERs for sensitivity analysis i and its corresponding reference analysis, while $x_{c_i,sens}$ and $x_{c_i,ref}$ are the respective values of x_c. Finally, $\epsilon_i \sim N(0, {\sigma_c}^2)$ are iid normal errors for each crosswalk analysis.

When crosswalk analyses could be conducted, the regression coefficients α_c, their corresponding standard errors, $\widehat{SE}[\hat{\alpha}_c]$, and samples sizes for the estimates are reported in Section 6. Note that some sensitivity analyses were

used in more than one crosswalk model. We use the coefficients and their standard errors as Gaussian priors in all subsequent model stages in which these covariates are included.

5.3. Estimation of Nonlinear log-GDP per capita response curve

The relationship between log-ICER and log-GDP per capita is modeled using a basis spline (B-spline)[4, 5]. In this section, we present B-splines, specification of constraints, discussion of trimming, and summary of spline ensembles.

5.3.1. B-splines linear tails

A spline basis is a set of piecewise polynomial functions with designated degree and domain. If we denote polynomial order by p, and the number of knots by k, we need $p + k$ basis elements s_j^p, which can be generated recursively as illustrated in Figure S5.1.

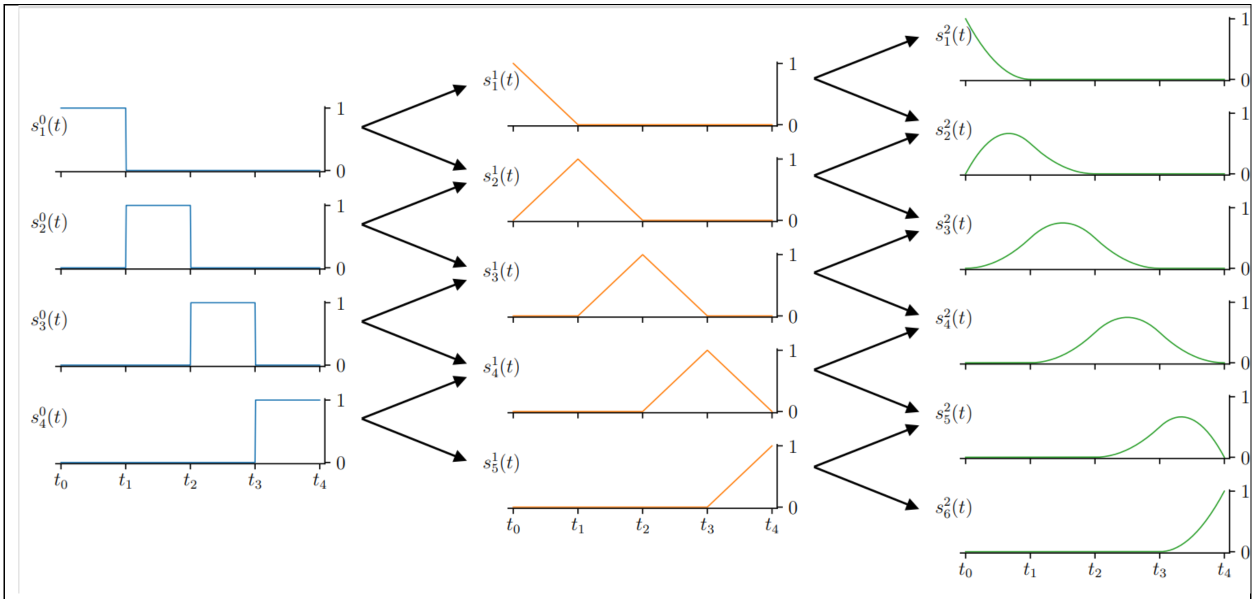

Figure S5.1. Recursive generation of b-spline basis elements (orders 0, 1, 2).

Given such a basis, we can represent any curvilinear relationship as the linear combination of the spline basis elements, with coefficients $\beta \in \mathbb{R}^{p+k}$

$$\boldsymbol{f(t) = \sum_{j=1}^{p+k} \beta_j^p s_j^p(t)} \quad \textbf{(2)}$$

An explicit representation of (2) is obtained by building a design matrix $\boldsymbol{X}$. Given a set of t values at which we have data, the jth column of $\boldsymbol{X}$ is given by the expression:

$$\boldsymbol{X_{\cdot,j}} = \begin{bmatrix} \boldsymbol{s_j^p(0)} \\ \vdots \\ \boldsymbol{s_j^p(t)} \end{bmatrix} \quad \textbf{(3)}$$

For extreme values of log-GDP per capita with little data, we need the capability to ensure that the outermost segments of the spline are linear, with slopes that match the adjacent segment at the knot. Splines with linear tails are often called natural splines.

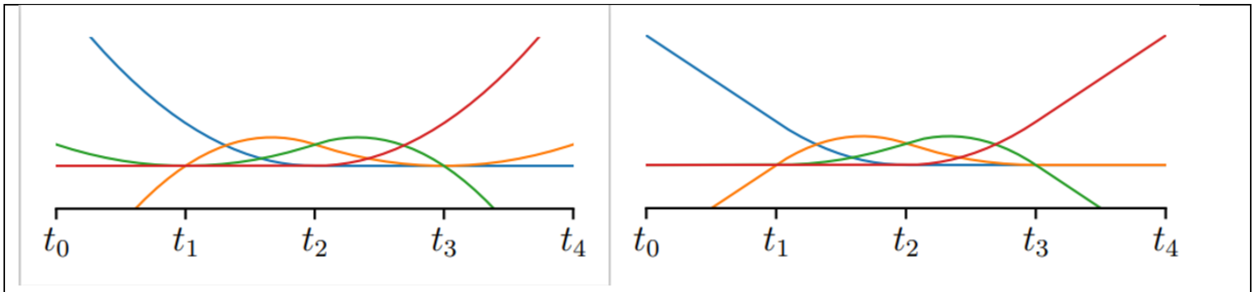

Figure S5.2. Left: spline bases. Right: spline bases with linear tails.

5.3.2 Robust Trimming Strategy

To robustify the approach against outliers, we use the trimming strategy, as discussed in [3,6]. The estimator

$$\min_{\beta \in B} \sum_i f_i(\beta) \tag{4}$$

Where f_i is as in (2) and B encodes all necessary constraints is extended to the 'trimmed' estimator

$$\min_{w \in \Delta, \beta \in B} \sum_i w_i f_i(\beta) \tag{5}$$

where each w_i is required to be between 0 and 1, and the total mass of w is constrained to equal 90% of the data volume. Specifically, this means that

$$\Delta = \{w : 0 \le w_i \le 1, \sum_i w_i = 0.9N\} \tag{6}$$

Where N is the total number of data points across studies. Thus the trimmed estimator finds the 90% most fittable data and fits them for β. Selecting a proportion of trimming has had a long history in terms of theory and recent methodological innovations [6,7]. However, thus far it has not been possible to automatically select the expected number of inliers. We chose 90% to include most of the data while remaining robust to a potential set of outliers. The same choice has been made in larger systematic analyses as well [8]. Alternative proportions are available, and we experimented with 95% and 100% inliers for the model we selected for the final estimates.

5.3.3 Spline Ensemble

Every model estimate intrinsically depends on the choice of knot placement used to generate the spline. To remove the effect of this choice on the estimates, we develop an ensemble over this knot placement, leaving only the choice of spline degree and number of knots as modeling choices.

Given the degree and number of knots, we automatically sample a set of knot placements for a feasible knot distribution. For each resulting knot placement, we fit a spline (using the trimming estimator) and then evaluate each resulting model by computing its fit and curvature, aggregating the final model as a weighted combination of the ensemble.

5.3.4 Sampling Knots from Simplex

To establish a reasonable feasible set from which to sample, we prefix a minimal set of the rules for the knot-placement and uniformly sample from this feasible set. Given a number of knots, the rules specify feasible ranges for each knot, and feasible gaps between knots. Specifically, given an interval $[t_0, t_k]$ delimited by terminal knots (which are always the minimum and maximum of the data), the feasible region of the interior knots $t_1, \ldots, t_{k-1}$ is given by

$$t_i \in [a_i, b_i], \text{for } i = 1, \dots, k-1, \qquad t_i - t_{i-1} \in [c_i, d_i] \text{ for } i = 1, \dots, k.$$

We enforce the rules

$$\boldsymbol{a_i} \geq \boldsymbol{t_0}, \boldsymbol{b_i} \leq \boldsymbol{t_k}, \quad \boldsymbol{c_i} \geq \boldsymbol{0}, \quad \sum_i \boldsymbol{c_i} \leq \boldsymbol{t_k} - \boldsymbol{t_0}. \qquad \textbf{(7)}$$

The knot placement that satisfies these four rules comprise a closed polyhedron $\{\boldsymbol{t}: \boldsymbol{Pt} \leq \boldsymbol{p}\}$, where,

$$\boldsymbol{P} = \begin{bmatrix} \boldsymbol{I} \\ -\boldsymbol{I} \\ \boldsymbol{D} \\ -\boldsymbol{D} \end{bmatrix}, \boldsymbol{p} = \begin{bmatrix} \boldsymbol{b} \\ -\boldsymbol{a} \\ \boldsymbol{d} \\ -\boldsymbol{c} \end{bmatrix}, \boldsymbol{D} = \begin{bmatrix} -\mathbf{1}\ \mathbf{1} & \cdots & \dots \\ \vdots\ \vdots & \ddots\ddots & \vdots\ \vdots \\ \dots & \cdots & -\mathbf{1}\ \mathbf{1} \end{bmatrix}, \boldsymbol{b} = \begin{bmatrix} b_1 \\ \vdots \\ b_{k-1} \end{bmatrix}, \boldsymbol{c} = \begin{bmatrix} c_1 \\ \vdots \\ c_k \end{bmatrix}, \boldsymbol{d} = \begin{bmatrix} d_1 \\ \vdots \\ d_k \end{bmatrix} \qquad \textbf{(8)}$$

We calculate the vertices of the polyhedron using the double description method in [9], and uniformly sample knot-placements from within the polyhedron. Each knot placement yields a model, fit using the trimmed constrained spline approach described above.

5.3.5 Scoring

Once the ensemble is created, we score the resulting risk curves using two criteria: model fit (measured using the log-likelihood) and total variation (measured using the highest order derivative). These scores balance competing objectives of fit and generalizability. Once we have these scores, denoted as s_1 and s_2, we normalize them to the range [0,1]:

$$\boldsymbol{v_i} = \frac{s_i - \min(s_i)}{\max(s_i) - \min(s_i)} \qquad \textbf{(9)}$$

and apply a logistic transformation. The transformation is used to make the scoring meaningful even in the presence of spurious curves in a large ensemble. We then multiply the scores

$$\boldsymbol{w} = \boldsymbol{w}_1^{p_1} \odot \boldsymbol{w}_2^{p_2} \qquad \textbf{(10)}$$

to down-weight models that are low under either criterion (fit or total variation). The final weights are normalized to sum to 1.

5.3.6 New nonlinear ‘signal’ covariate

We fit a model of log-ICER on log-GDP per capita using a robust spine ensemble on log-GDP per capita with degree 2, two knots, and linear tails. This model also included the crosswalk covariates, when the analysis could be conducted, and we experimented with including as covariates log burden of disease, measured with either DALYs or prevalence and intervention keywords. We placed Gaussian priors with means $\hat{\alpha}_c$ and standard deviation $\widehat{SE}[\hat{\alpha}_c]$ on the crosswalk covariates’ coefficients. We used this model to generate a nonlinear log-GDP per capita response curve, which is encoded into a new nonlinear covariate called ‘signal’ and included in subsequent stages of the analysis. The shape of this transformation for each cause-group group is displayed in Section 7.

In particular, this allows us to fit a linear mixed effects model of the form:

$$\boldsymbol{y_{ij}} = \boldsymbol{\beta_0} + \mathbf{signal_{ij}} \times \boldsymbol{\beta_s} + \boldsymbol{x_{1ij}\beta_1} + \cdots + \boldsymbol{x_{kij}\beta_k} + \boldsymbol{u_i} + \boldsymbol{\epsilon_{ij}} \qquad \mathbf{(11)}$$

Where $\epsilon_{ij} \sim N(0, \sigma_{ij}^2)$ are known for each observation, and $u_i \sim N(0, \gamma)$ is a random study-specific intercept with unknown variance γ.

5.4. Covariate Selection

Additional covariates are selected using a Lasso stategy described below in the context of linear mixed effects models. [10,11] In considering potential covariates, we enforce that every categorical covariate has some variation; in particular every indicator covariate has at least two studies in each category.

• We iteratively decrease the weight on the Lasso regularizer and let coefficients of bias-covariates enter the model in the order derived from the Lasso solutions.

• As a group of coefficients enters the model, we test it for statistical significance.
  – If the coefficients are significant, we compute their posterior distribution and use this posterior as the prior for these coefficients for the next round.
  – If the coefficients are not significant, the process terminates, and we return the list of (significant) covariates obtained so far.

We included the signal and crosswalk covariates when estimated as pre-selected covariates without the Lasso regularizer in all models above. We added Gaussian priors to the coefficients of the crosswalk covariates with mean $\hat{\alpha}_c$ and standard deviation $\widehat{SE}\,[\hat{\alpha}_c]$, when estimated as in Section 5.1.

Covariates with low variance or that are highly correlated with others are unlikely to be selected by this process, since including them would likely inflate the variance of the resulting estimators by an amount that outweighs the reduction in bias. This is a limitation of the current data set, and future work to expand the data set by extracting sensitivity analyses for a wider number of covariates could allow for the stable estimation of additional parameters.

There is ongoing methodological work to improve variable selection in the presence of collinearity. Based on early work showing the advantages of bridge regression vs. lasso [12] in the presence of correlation, the elastic net penalty [13] has been used, and in principle able to find groups of correlated predictors. Practical use requires additional parameter selection. We are currently looking into methods based on nonconvex regularizes as well [14]. These innovations can further improve variable selection in future work, but now we test for collinearity using basic tests before the lasso procedure starts.

One of the difficult questions in any variable selection procedure is when to stop. The methodology in step 1 builds on the Lasso methodology, but has an automatic termination criteria, stopping as soon as sequentially selected variables (selected across a range of the Lasso parameter) cease to be statistically significant in a standard Gaussian analytical framework.

Bias covariates that pass the selection process are included in the next stage of the model fitting.

5.5. Gaussian prior cross-validation

In order to further safeguard against overfitting, we included a Gaussian prior on all covariates. We used 10-fold cross validation to determine the prior standard deviation, τ_{cv}, to apply to the coefficients of all covariates other than the four crosswalk covariates. We fit models of the same form as (4) with priors on coefficients β . For the seven crosswalk covariates, we used the priors calculated in Section 5.2. For all others, we used a common $N(0, \tau_{cv}^2)$ prior on their coefficients after standardizing the covariates to have mean 0 and unit variance. We used a grid-search to select the value of τ , that minimizes the MSE for predicting data in the hold-out set.

5.6. Meta-Regression Analysis

Once the signal covariate is obtained (Section 5.3), bias covariates are selected (Section 5.4), and priors are calculated for crosswalk (Section 5.2) and non-crosswalk (Section 5.5) covariates, we convert the priors on standardized covariates calculated in Section 5.5 to an unstandardized scale and fit a final model of the form

$$y_{ij} = \beta_0 + x_{1ij}\beta_1 + \cdots + x_{kij}\beta_k + u_i + \epsilon_{ij} \qquad (12)$$

where coefficients for crosswalk covariates have priors $\beta_c \sim N\left(\hat{\alpha}_c, \widehat{SE}[\hat{\alpha}_c]^2\right)$, as estimated in Section 5.1.

Coefficients for all other covariates selected in Section 3, including the signal covariate, have priors $\beta_l \sim N(0, \tau_{cv}^2)$, as selected in Section 5.4. $u_j \sim N(0, \gamma)$ is a study-specific random intercept and $\epsilon_{ij} \sim N(0, \sigma_\epsilon^2)$ are independent error terms. Parameter estimates for each cause-type group are reported in Section 6.
Parameters β and γ are estimated using maximum likelihood, as detailed in [3]. Standard errors of β are estimated by taking the standard deviation across 1000 samples from the posterior distribution of $\hat{\beta}$.

Section 5 References

1. Rosettie KL, Joffe JN, Sparks GW, Aravkin A, Chen S, Compton K, et al. Cost-effectiveness of HPV vaccination in 195 countries: A meta-regression analysis. PLOS ONE 2021;16:e0260808.

2. Janko MM, Joffe J, Michael D, et al. Cost-effectiveness of rotavirus vaccination in children under five years of age in 195 countries: A meta-regression analysis. Vaccine 2022; 40: 3903–17.

3. Zheng P, Barber R, Sorensen RJD, Murray CJL, Aravkin AY. Trimmed Constrained Mixed Effects Models: Formulations and Algorithms. J Comput Graph Stat 2021; 30: 544–56.

4. de Boor C. A Practical Guide to Spline. 1978 DOI:10.2307/2006241.

5. Friedman JH. Multivariate Adaptive Regression Splines. Ann Stat 1991; 19: 1–67.

6. Aravkin A, Davis D. Trimmed Statistical Estimation via Variance Reduction. Math Oper Res 2020; 45: 292–322.

7. Rousseeuw, P. J. Multivariate estimation with high breakdown point. Math Stat Appl 1985; 8: 283–97.

8. Murray CJL, Aravkin AY, Zheng P, et al. Global burden of 87 risk factors in 204 countries and territories, 1990–2019: a systematic analysis for the Global Burden of Disease Study 2019. The Lancet 2020; 396: 1223–49.

9. Motzkin TS, Raiffa H, Thompson GL, Thrall RM. 3. The Double Description Method. In: 3. The Double Description Method. Princeton University Press, 2016: 51–74.

10. Bondell HD, Krishna A, Ghosh SK. Joint Variable Selection for Fixed and Random Effects in Linear Mixed-Effects Models. Biometrics 2010; 66: 1069–77.

11. Müller S, Scealy JL, Welsh AH. Model Selection in Linear Mixed Models. Stat Sci 2013; 28: 135–67.

12. Fu WJ. Penalized Regressions: The Bridge versus the Lasso. J Comput Graph Stat 1998; 7: 397–416.

13. Zou H, Hastie T. Regularization and variable selection via the elastic net. J R Stat Soc Ser B Stat Methodol 2005; 67: 301–20.

14. Zheng P, Askham T, Brunton SL, Kutz JN, Aravkin AY. A Unified Framework for Sparse Relaxed Regularized Regression: SR3. IEEE Access 2019; 7: 1404–23.

Section 6. Implementation of the five-stage meta-regression analysis

6.1 Models

6.1.1 Grouping interventions by cause and type

In this study, we introduced two adaptations of the five-stage statistical model, because of the smaller samples of published ICERs for the interventions for HIV, malaria, syphilis, and tuberculosis (TB). 1) We grouped interventions by cause and type (prevention, diagnostics, treatment) with the same cost and efficacy variables to increase the sample sizes when possible, such as TB diagnostics. 2) We systematically compared estimates with all possible combinations of tools or alternate sets of models and selected the model with the best fit.

In our previous research, the HPV and rotavirus vaccines were data-rich interventions.[1,2] We had a large sample of ICERs to conduct the meta-regression analysis for a single intervention. The estimating equation was:

$$\log ICER_{ij} = \boldsymbol{\alpha_j} + \sum_k \beta_{jk} X_{ik} + \sum_l \gamma_{jl} Y_{il} + \sum_m \delta_{jm} Z_{im} + \mu_s + \varepsilon_i \qquad (1)$$

Where *i* refers to a ratio, *j* refers to intervention, and *s* refers to study for random effect. The subscript j denotes that coefficients are specific to intervention.

X_k= two variables explaining true variation: GDP per capita, DALYs per capita

Y_l= five methods variables explaining bias: cost discount rate, outcome discount rate, perspective (payer vs social), outcome (DALY vs QALY), time horizon

Z_m= Intervention characteristics, such as cost, coverage, vaccine type, target population, and standard of care.

For the analyses of interventions for HIV, malaria, syphilis and TB, the sample of ICERs for each intervention was smaller. We grouped data by cause and type for interventions with similar measures of cost and efficacy. The adapted the estimating equation was:

$$\log ICER_i = \sum_j \alpha_j I_{Gij} + \sum_k \beta_{Gk} X_{ik} + \sum_m \delta_{Gm} Z_{im} + \mu_s + \varepsilon_i \qquad (2)$$

Where I_{Gij} = Matrix of indicator variables for each intervention j in group G. The subscript G denotes that coefficients are specific to a group, and coefficient for α_j are specific to an intervention as in equation (1). The methods variables used in in previous research,[1,2] tended to fall in a single category (see Table 1 and Table S6.4), and were not used in the analysis.

6.1.2 Minimum of eight models estimated for each cause-type group

All cause-type groups were estimated in a minimum of eight models, which were all combinations of the following three options: 1) impose monotonicity constraint on the non-linear response covariate or "GDP spline, 2) include the interventions, termed "keywords" in the code, in the spline fit or not, which meant pre-selecting them for the final model, and 3) measure burden of disease by DALYs or prevalence. These eight models did not include stage 1 (See appendix S5). For three cause-type groups, these eight models were the only ones estimated, because the sample of sensitivity analyses did not meet the inclusion criteria: antenatal syphilis screening, TB prevention, and chemotherapy for DS-TB.

6.1.3 Crosswalk analysis for the HIV, malaria, and TB samples

The first tool was the cross-walk analysis described as stage 1 in Section 5.1. Unlike our samples for the HPV vaccine and rotavirus vaccines, the articles for some of the cause-type groups did not report many sensitivity analyses for variables for cost and efficacy, nor for methods variables. Inclusion criteria for the samples for the crosswalk analyses were the same as for the main analysis, which is two articles and three ICERs. Only four cause-

type groups met our inclusion criteria: ART, HIV prevention, malaria prevention, and TB diagnostics. (Sample sizes ranged from 78 to 824 for estimates of the crosswalk parameters for the HPV vaccine and from 12 to 426 for the rotavirus vaccine.) In addition, this tool was only available for a few variables within four cause-type groups, as shown in Table S6.1 below.

cause	intervention_type	covariate	beta	se_beta	n_article	sample_size
hiv/aids	Prep	log_per_year_cost	1.915	0.165	5	15
hiv/aids	treatment	efficacy	-0.005	0.003	10	85
hiv/aids	treatment	log_per_year_cost	0.656	0.055	14	151
malaria	prevention	log_per_prevention_year_cost	1.53	0.147	2	12
tuberculosis	diagnostic	log_per_prevention_year_cost	0.213	0.122	3	12
tuberculosis	diagnostic	sensitivity	-0.052	0.013	2	11
tuberculosis	diagnostic	specificity	-0.039	0.012	2	11

Table S6.1 Parameter estimates for crosswalk and number of articles and ICERs in the sample

6.1.4 Including sensitivity analyses in main analysis

The second tool was to include the sensitivity analysis in the main analysis. We did not apply the inclusion criteria to this tool, which meant that the eight models described in Section 6.3.1 were estimated with and without sensitivity analyses for a total of 16 models for all cause-type groups.

For cause-type groups with enough sensitivity analyses to conduct the crosswalk analyses a total of 32 models were estimated. Each tool multiplied the number of analyses, so the option of using the crosswalk analysis or not, and option of including the sensitivity analyses in the main analysis or not, created a total of four sets of eight models (Table S6.2):

		Crosswalk analysis	
		Yes	No
Include sensitivity analyses in main analysis	Yes	8 models	8 models
	No	8 models	8 models

Table S6.2 Additional model specifications

6.2 Inclusion criteria and exclusions from the analysis sample and final meta-regression models

6.2.1 Inclusion criteria and exclusions from the analysis sample

We included all ratios from the registries that met five inclusion criteria:

1. Categorized into an intervention with a minimum of two published articles and three published ICERs
2. Mapped to one of five GBD causes: HIV/AIDS, malaria, syphilis, drug-susceptible (DS) TB, or multi-drug resistant (MDR) TB
3. Mapped to a GBD country as opposed to a multi-country region or hypothetical location
4. Reported a currency year during or after 1990, the earliest year with GBD 2019 estimates
5. Comparator intervention was no intervention, standard or care, or placebo as opposed to “other” (see appendix S4)

After applying these inclusion criteria, we had 41 keywords in nine cause-type groups: HIV/AIDS prevention, antiretroviral therapy (ART), malaria prevention, malaria treatment, syphilis diagnostics, TB prevention, TB diagnostics, DS-TB treatment, and MDR-TB treatment.

We excluded 16 keywords and their associated ratios. We excluded seven keywords in the HIV/AIDS prevention group, because the interventions were not defined consistently, and cost or efficacy variables were not the same across ratios. For example, the three interventions with published ICERs for cotrimoxazole prophylaxis against opportunistic infections for people infected with HIV/AIDS are too dissimilar to group with the same keyword: employer-based delivery for adults,[33] home-based delivery for adults and children,[34] and facility-based delivery for children.[35] On closer inspection, we recategorized some ratios into different keywords, which meant that three keywords no longer met the first inclusion criterion. We also excluded keywords for a hypothetical intervention (HIV/AIDS vaccines), and one no longer in development (vaginal microbicide gel). Finally, we excluded four keywords, because estimates from the small samples were unstable: circumcision, post-exposure prophylaxis for HIV/AIDS, malaria treatment, and MDR-TB treatment. The majority of published results were cost-saving for post-exposure prophylaxis for men who have sex with men (MSM) with known exposure to HIV/AIDS infection and for multi-drug resistant MDR) TB treatment. This resulted in a reduction in the total cause-type groups from nine to seven: pre-exposure prophylaxis for HIV/AIDS, ART, malaria prevention, syphilis diagnostics, TB prevention, TB diagnostics, and drug sensitive (DS)-TB treatment.

6.2.2. Exclusions from the final meta-regression models

The sample for the selected meta-regression models was 612 ratios from 106 articles (Table S6.3). Four key exclusions are: 1) cost-saving results, 2) sensitivity analyses when the models with them were not selected, 3) sensitivity analyses when the cost or efficacy variable that was varied in the sensitivity analysis was not selected (i.e. there was no variable to explain why the ICERs differed between the two observations), and 4) 10 percent trimming of outliers, or 5% trimming of outliers for the PREP model. Descriptive statistics of this sample (Table S6.4) are similar to Table 1.

Table S6.3 Table of sample exclusions by cause-type group

	Total				HIV - ART				HIV - PREP				Malaria prevention			
	Running Total		Exclusions		Running Total		Exclusions		Running Total		Exclusions		Running Total		Exclusions	
	Article	Ratio	Article	Ratio	Article	Ratio	Article	Ratio	Article	Ratio	Article	Ratio	Article	Ratio	Article	Ratio
**Analysis sample (Table 1)**	**144**	**1273**			**74**	**612**			**10**	**86**			**15**	**148**		
Model with SA not selected			0	329	74	323		289	10	86			15	148		0
Cost saving	**135**	**857**	9	87	66	294	8	29	10	85		1	15	128		20
Missing values for covariates	**133**	**850**	2	7	65	293	1	1	10	85		0	15	128		
Keywords no longer meet criteria			0	15	65	293			10	85			15	126		2
10% outliers (5% for PREP)	**118**	**756**	15	79	61	264	4	29	9	80	1	5	12	113	3	13
Covariate with SA not selected	**118**	**662**	0	94	61	264			9	80	0	0	12	99		14
Missing values for covariates	**111**	**649**	7	13	57	258	4	6	9	80			12	99		
Keywords no longer meet criteria	**113**	**612**	5	37	57	258			9	80		0	10	74	2	25
**Final sample (Table 6.4)**	**106**	**612**	33	661	**57**	**258**			**9**	**80**			**10**	**74**		

	Syphilis diagnostics				DS - TB treatment				TB prevention				TB diagnostics			
	Running Total		Exclusions		Running Total		Exclusions		Running Total		Exclusions		Running Total		Exclusions	
	Article	Ratio	Article	Ratio	Article	Ratio	Article	Ratio	Article	Ratio	Article	Ratio	Article	Ratio	Article	Ratio
**Analysis sample (Table 1)**	**11**	**222**			**5**	**10**			**10**	**62**			**19**	**133**		
Model with SA not selected	11	222			5	10			10	55		7	19	100		33
Cost saving	11	221		1	5	8	0	2	10	25		30	18	96	1	4
Missing values for covariates	10	215	1	6	5	8	0	0	10	25			18	96		
Keywords no longer meet criteria	10	215			5	8			10	25			18	83	0	13
10% outliers	6	193	4	22	5	8		0	9	22	1	3	16	76	2	7
Covariate with SA not selected	6	113	0	80	5	8	0	0	9	22		0	16	76		
Missing values for covariates	6	113			5	8			8	20	1	2	14	71	2	5
Keywords no longer meet criteria	4	111	2	2	5	8			8	20			13	61	1	10
**Final sample**	**4**	**111**			**5**	**8**			**8**	**20**			**13**	**61**		

Legend: ART=Antiretroviral therapy, DS=drug susceptible, PREP=Pre-exposure prophylaxis, SA=sensitivity analysis, TB=Tuberculosis

6.3 Descriptive statistics on final sample for meta-regression estimates

Table S6.4: Descriptive statistics on final sample for meta-regression estimates: 612 ratios from 106 articles

	HIV/AIDS ART	HIV/AIDS PREP	Malaria prevention	Syphillis diagnostics	DS-TB treatment	TB prevention	TB diagnostics
Total sample size	258	80	74	111	8	20	61
Sensitivity analysis counted in total	0	15	2	42	2	0	0
Study characteristics, number (percentage)							
GBD Super-region							
Central Europe, Eastern Europe, & Central Asia	16 (6)	2 (3)	0	0	0	0	0
High-income	154 (60)	25 (31)	0	0	0	15 (75)	20 (33)
Latin America & Caribbean	4 (2)	6 (8)	0	11 (10)	0	1 (5)	7 (12)
North Africa and the Middle East	0	1 (1)	1 (1.0)	2 (2)	0	0	0
South Asia	6 (2)	0	0	2 (2)	0	0	11 (18)
Southeast Asia, East Asia & Oceania	16 (6)	0	0	8 (7)	4 (50)	0	0
Sub-Saharan Africa	62 (24)	46 (58)	73 (99)	86 (77)	4 (50)	4 (20)	23 (38)
Year published							
1990-1994	0	0	0	0	0	0	0
1995-1999	0	0	0	0	0	2 (10)	0
2000-2004	17 (7)	0	2 (3)	0	4 (50)	10 (50)	0
2005-2009	38 (15)	8 (10)	9 (12)	3 (3)	3 (38)	5 (25)	15 (25)
2010-2014	189 (73)	60 (75)	9 (12)	85 (77)	0	3 (15)	40 (66)
2015-2017	14 (5)	12 (15)	54 (73)	23 (21)	1 (12)	0	6 (10)
Methods, number (percentage)							
Health outcome measure							
QALYs	192 (74)	29 (36)	0	0	0	15 (75)	22 (36)
DALYs	66 (26)	51 (64)	74 (100)	111 (100)	8 (100)	5 (25)	39 (64)
Cost discount rate							

< 3%	24 (9)	1 (1)	0	107 (96)	0	2 (10)	1 (2)
3%	192 (74)	79 (99)	72 (97)	4 (4)	8 (100)	17 (85)	60 (98)
> 3%	42 (16)	0	2 (3)	0	0	1 (5)	0
Health outcome discount rate							
< 3%	21 (8)	1 (1)	2 (3)	0	0	2 (10)	1 (2)
3%	195 (76)	79 (99)	72 (97)	111 (100)	8 (100)	17 (85)	60 (98)
> 3%	42 (16)	0	0		0	1 (5)	0
Perspective							
Societal (1) or limited societal (3)	55 (21)	0	63 (85)	0	1 (12)	13 (65)	58 (95)
Health care payer (2) or sector (4)	199 (77)	78 (97)	11 (15)	111 (100)	7 (88)	7 (35)	3 (5)
Missing	4 (2)	2 (3)	0	0	0	0	0
Time Horizon							
Lifetime	88 (34)	5 (6)	14 (19)	23 (21)	2 (25)	13 (65)	42 (69)
Less than lifetime	170 (66)	75 (94)	60 (81)	85 (79)	6 (75)	7 (35)	19 (31)
Type of comparator							
Placebo (1)	5 (2)	0	0	0	0	0	0
Standard Care (2)	163 (63)	49 (61)	16 (22)	0	1 (12)	2 (10)	41 (67)
None (3)	90 (35)	31 (39)	58 (78)	111 (100)	7 (88)	18 (90)	20 (33)
Intervention characteristics, median (Interquartile range)							
Drug cost per person per year (HIV) and per episode (DS-TB) US$	6,208 (231, 17,746)				93 (93, 278)		
Drug cost per year of protection in $US		248 (186, 7,949)	5 (1, 6)			58 (5, 165)	
Cost per test in $US				1 (1, 1)			21 (14, 59)
Efficacy	88 (70, 90)	68 (58, 68)	91 (42, 91)		72 (57, 86)	70 (69, 75)	
Sensitivity				86 (75, 86)			79 (73, 83)
Specificity				99 (99, 99)			99 (98, 99)

Legend: ART = Antiretroviral therapy, DALY = disability-adjusted life-year, DS = drug susceptible, GBD = Global Burden of Disease, Injuries, and Risk Factor study, PREP = Pre-exposure prophylaxis, QALY = quality-adjusted life-year, TB = Tuberculosis, US$ = United States Dollars.

6.4 WHO guidelines, community costs, and efficacy of interventions with predicted ICERs

Table S6.5. WHO guidelines and commodity costs for interventions with predicted ICERs

Intervention and target population	WHO recom-mendation	Cost in $US	Description of cost	Source of prices	Notes
ART for prevention adults	4.6.1, Table 4.3[6]	58.2*	annual cost of drugs per person for adult	GFPPM RP: ARVs[7]	Reference cost per year for 1st line adult treatment Dolutgravir/Lamivudine/Tenofovir (DTG/3TC/TDF) 50/300/300mg tablets per WHO recommendation. *EXW price in US$
ART for prevention 0 to 9 years	4.6.1, Table 4.3[6]	133.5*	annual cost of drugs per person for a child 14-19.9 kg	GFPPM RP: ARVs[7]	Child treatment with Dolutgravir/Lamivudine/Abacavir (DTG/3TC/ABC) is preferred 1st line regimen. Global Fund price for DTG alone and 3TC/ABC + Lopinavir/Ritonavir (LPV/r) is (6.45). Assuming 3TC/ABC can be ordered separately, annual cost for DTG/3TC/ABC for 3-5.9 kg = $44.4, 6-9.9 kg = $80.1, 10-13.9 kg = $106.8, and 14-19.9 kg = $133.5, 20-24.9 kg = $160.2. *EXW price in US$
PREP for heterosexuals adults and MSM	3.2.1[6]	28.8*	annual cost of drugs per person	GFPPM RP: ARVs[7]	Reference cost per year for 30 tablets of Tenofovir (TDF) 300 mg is $2.40 x 12 months. NB: WHO recommendation is for TDF only. Global Fund price is listed as treatment for Hepatais B. *EXW price in US$
Antenatal syphilis screening 0 to 11 months	3[8]	0.60	US$18 per 30 rapid syphilis test kit divided to get cost per test	UNICEF[9]	WHO guideline for screening pregnant women strategy A in low prevalence settings is single rapid syphilis test. Strategy C in high prevalence setting is rapid syphilis test and if positive, rapid plasma reagin.
Bednets all ages	4.1.1.[10]	0.72	cost per protection year=2.16 per net/3 years	GFPPM RP: Long-lasting insecticide nets[11]	Standard reference price for 180*190*150 cm pyrethroid-only net. Net is 4th of 8 sizes. FOB and delivery price. Length of protection from WHO guideline.[10]

Table S6.5. WHO guidelines and commodity costs for interventions with predicted ICERs					
Intervention and target population	WHO recom-mendation	Cost in $US	Description of cost	Source of prices	Notes
Malaria vaccine ages 0 to 4 years	5.3[10]	40	cost per protection year=($10/dose*4 doses)/5 years	Assumptions from Malaria Vaccine Implementatio n Programme cost analysis.[12]	The RTS,S/AS01 malaria vaccine should be provided in a four-dose schedule in children from 5 months of age. As a new vaccine, its price is not included in 2020 WHO Global Vaccine Market Report. Duration based on 0 to 5 age group.
IPT for pregnant women and infants 0 to 11 months	4.2.1[10]	3.6	cost per protection year=(0.3/treatment* 3 treatments)/0.25 years	GFPPM RP: Antimalairal medicines[13]	Sulphadoxine-pyrimethamine (SYP 3 treatments of 1500/75 mg. Each treatment is 3 tablets of 500/25 mg. 1 month apart for pregnant women in endemic areas.
IPT for infants ages 0 to 11 months	4.2.2[10]	0.45	cost per protection year = (0.1/treatment * 3 treatments)/0.67 years	GFPPM RP: Antimalairal medicines[13]	ICERs and guidelines are for infants. Sulphadoxine-pyrimethamine (SP) 3 treatments of 500/25 mg. Each treatment is 1 tablets of 500/25 mg at 2nd & 3rd DTP-3 vaccine and 9-month measles vaccine. Given treatment intervals, duration of protection is 2 months for first treatment, and 3 for second and third treatment or 8 months total.
IRS all ages	4.1.1[10]	4.4	cost per protection year=8.8 per spray/2years	GFPPM RP: Antimalairal medicines[13]	Bendiocarb wettable power (WP), 80% WP in non-soluble bag, 100g sachet, 120 x sachets. WHO guidelines have no information about duration of protection of IRS. We can use 2 years, and based on a systematic review that explains that bendiocarb for which we have cost estimates is used biannually.[14]
Xpert TB test	Diagnosis 2.1[15]	12.1*	cost per test	Stop TB Partnership GDF Product Catalog[16]	GeneXpert IV, 4-modules with desktop computer @17,000, Xpert Mycobacterium TB complex/resistance to rifampin (MTB/RIF) kit of 50 tests @ 1x50 tests @499.00, Dust filter for GeneXpert system for GXIV-4 @350.00, GeneXpert new module for 6-color system @3,360.00. Calculations assumes 10,000 tests performed with equipment.

Table S6.5. WHO guidelines and commodity costs for interventions with predicted ICERs					
Intervention and target population	WHO recom-mendation	Cost in $US	Description of cost	Source of prices	Notes
BCG vaccine against TB 0 to 4 years-GAVI	Routine immunization - table 2[17]	0.01, 0.02	cost per protection year=$0.12 per dose/10 years of $0.22 per dose/10 years	2020 WHO Global Vaccine Market Report[18]	Universal BCG vaccination at birth is recommended in countries or settings with a high incidence of TB and/or high leprosy burden. A single dose of BCG vaccine should be given to all healthy neonates at birth, ideally together with Hepatitis B birth dose. 0.12 and 0.22 per dose of BCG vaccine for UNICEF purchases (Gavi eligible and middle-income countries), and PAHO revolving fund, respectively. Abubakar et al. (2013) report good evidence of efficacy for up to 10 years.[19]
Preventive therapy for TB adults	Prevention 17[20]	3.45	6-month cost of drugs per person	Stop TB partnership GDF Product Catalog[16]	3 options are: 1) 6 or 9 months of isoniazid, 2) 3 months weekly rifapentine plus isoniazid, or 3) 3 months daily isoniazid plus rifampin. We use the adult and adolescent formulation of isoniazid 300 mg for 6 months at ((11.86+13.52)/2)/672) per pill.
Chemotherapy for DS TB all ages	Treatment 1.1[21]	11.27	full treatment cost	Stop TB partnership GDF Product Catalog[16]	Adult & adolescent formulations of fixed-dose combination of isoniazid, rifampin, pyrazinamide, and either ethambutol or streptomycin for 2 months (HRZE 150/75/400/275) at US$30.93/336 per pill, and isoniazid and rifampin for 4 months (4HR 150/75) at US$15.65/336 per pill
Legend: * denotes parameter was selected in final model and estimate is used in predictions, adult = ages 10 or more years, ART = Antiretroviral therapy, ARV = antiretroviral drugs, BCG = bacille Calmette-Guerin, DS= drug susceptible, EWX=exworks, meaning buyers pay for transport, customs, and all other expenses, FOB=free on board means the price of goods at the border of exporting country; GAVI=GAVI The Vaccine Alliance, GDF=Global Drug Facility, GFPPM RP=Global Fund Pooled Procurement Mechanism Reference Pricing, IRS = Indoor residual spraying, IPT = Intermittent preventive treatment for malaria, MSM = Men who have sex with men, Option B+ = HIV screening for pregnant women and lifelong ART, PREP = Pre-exposure prophylaxis, TB = Tuberculosis, US$= United States dollar					

Table S6.6. Efficacy of interventions with predicted ICERs			
Intervention and target population	Efficacy rate	Description of efficacy rate	Notes
ART for prevention adults	93.5	virologic suppression	Estimate of efficacy of viral suppression among pregnant women is 92-95% for NRTI/PI (Zidovudine/Lamivudine/Lopinavir-Ritonovir (AZT/3TC/LPV-r)) and NRTI (Zidovudine/Lamivudine/Abacavir (AZT/3TC/ABC) regimens.[22] We used the mid-point of the range. Later comparison of NRTI/INSTI (Dolutegravir/Tenofovir/Lamivudine-Emtricitabine (DTG/TDF/3TD-FTC)) to NRTI/PI (Efavirenz/ Tenofovir/Lamivudine-Emtricitabine (EVF/TDF/3TC-FTC) regimens showed that DTG had significantly higher odds of suppression at all time points up to 50 weeks from initiation.[23]
ART for prevention 0 to 9 years	93.5	virologic suppression	Estimate of efficacy of viral suppression among pregnant women is 92-95% for NRTI/PI (Zidovudine/Lamivudine/Lopinavir-Ritonovir (AZT/3TC/LPV-r) and NRTI (Zidovudine/Lamivudine/Abacavir (AZT/3TC/ABC) regimens.[22] We used the mid-point of the range. Cochrane review of ART for children younger than 3 years reports NRTI/PI regimen with Lopinavir/Ritonavir (LPV/r) is more efficacious than NRTI/NNRTI regimen with Nevirapine (NPV), but doesn't provide efficacy relative to no treatment.[24] Later comparison of NRTI/INSTI regimen with Dolutegravir (DTG) to standard of care regimens with NNRTI or PI showed that DTG in children and adolescents, but did not compare efficicies. [25]
PREP for heterosexuals adults and MSM	67*	acquiring HIV (21-24 months)	Reduction in the risk of acquiring HIV infection from Tenofovir/Emtricitabine (TDF-FTC) vs placebo (RR 0.49; 95% CI 0.28 to 0.85), and TDF only versus placebo (RR 0.33; 95% CI 0.20 to 0.55).[26]
Antenatal syphilis screening 0 to 11 months	0.86,* 0.99	sensitivity, specificity	WHO guidelines refer tp Rogozińska et al (2017).[27]
Bednets all ages	44*	severe malaria	Insecticide treated nets reduce child mortality from all causes by 17% compared to no nets (rate ratio 0.83, 95% CI 0.77 to 0.89), incidence of uncomplicated episodes of P falciparum malaria by almost a half (rate ratio 0.55, 95% CI 0.48 to 0.64), prevalence of P falciparum malaria by 17% (RR 0.83, 95% CI 0.71 to 0.98), severe malaria episodes by 44% (rate ratio 0.56, 95% CI 0.38 to 0.82).[28]
Malaria vaccine ages 0 to 4 years	36*	clinical malaria in children	Results from month 0 for an average of 4 years, of 3 doses + booster at 20 months relative to the control group. In children (5-17 months) vaccine efficacy against malaria was 36·3% (95% CI 31·8-40·5) and severe malaria was 32.2% (95% CI 13·7 to 46·9). In young infants (6-12 weeks), vaccine efficacy against malaria was 25·9% (95% CI 19·9-31·5) and severe malaria was 17·3% (95% CI -9·4 to 37·5)).[29]

Table S6.6. Efficacy of interventions with predicted ICERs			
Intervention and target population	Efficacy rate	Description of efficacy rate	Notes
IPT for pregnant women, and infants 0 to 11 months	61*	maternal antenatal parasitaemia	For women in their first or second pregnancy, malaria chemoprevention with any drug regimen reduces the risk of antenatal parasitaemia by around 61% (RR 0.39, 95% CI 0.26 to 0.58).[30] In an analysis confined only to intermittent preventive therapy with Sulphadoxine-pyrimethamine (SP), the estimates of effect and the quality of the evidence were similar. For infants of women in their first and second pregnancies, malaria chemoprevention reduces placental parasitaemia by around 46% (RR 0.54, 95% CI 0.43 to 0.69).[30] DALYs are for pregnant women and infants 0-11 months. We used efficacy rate for pregnant women rather than infants in the predictions.
IPT for infants ages 0 to 11 months	30*	clinical malaria incidence	IPT reduced the incidence of clinical malaria by 30%, but the effect size varied over time and between drugs.[31]
IRS all ages	54	incidence of reinfection	IRS was shown to be effective in protecting children aged less than five years from reinfection with malaria parasites following radical cure: over an 11 month period, the efficacy against infection was 54%.[32] In the same setting, malaria case incidence assessed by passive surveillance was probably reduced in children aged one to five years: efficacy = 14%, but not in children older than five years: efficacy = -2%.[32] Adding IRS using a pyrethroid-like insecticide did not appear to markedly alter malaria incidence.[32]
Xpert TB test	64.6,* 99*	sensitivity, specificity	For sputum specimens in children, Xpert Mycobacterium TB complex/resistance to rifampin (MTB/RIF) pooled sensitivity verified by culture was 64.6% (55.3% to 72.9%) and specificity was 99.0% (98.1% to 99.5%).[33] Irrespective of signs and symptoms, in people living with HIV, Xpert MTB/RIF pooled sensitivity was 61.8% (53.6 to 69.9) and specificity was 98.8% (98.0 to 99.4); in non-hospitalized people in high-risk groups, sensitivity 69.4% (47.7 to 86.2) and specificity was 98.8% (97.2 to 99.5).[33]
BCG vaccine against TB 0 to 4 years-GAVI	80*	serious forms of TB in children	The US Centers for Disease Control and Prevention's summary of the evidence concluded that vaccine efficacy in children is "high (i.e. 80%).[34] A systematic review and meta-analysis of recent studies reported it was 71% effective against active TB.[35] Meta-analyses have consistently adjusted for latitude of the study site.

Table S6.6. Efficacy of interventions with predicted ICERs			
Intervention and target population	Efficacy rate	Description of efficacy rate	Notes
Preventive therapy for TB adults	64*	prediction based on TST positive	For TB preventive intervention, there are 3 recommended regimens. Cost estimates are for 6 months of Isoniazid. According to the WHO guidelines, the efficacy of 6 month of isoniazid was 33% for people living with HIV, and 64% for people with latent TB. "A systematic review of [randomized controlled trials in people living with HIV] showed isoniazid monotherapy reduces the overall risk for TB by 33% (RR 0.67; 95% CI 0.51; 0.87), and the that preventive efficacy reached 64% for people with a positive [tuberculin skin test] (RR 0.36; 95% CI 0.22; 0.61) (18).[20] The authors don't offer additional evidence for people without HIV, or suggest that it's lacking. US Center for Disease Control and Prevention guidelines do not add evidence from systematic reviews, but recommend alternatives to the 6-month isoniazid. Alternatives have higher cost and lower toxicity.[36] The 6-month isoniazid it the best estimate of efficacy relative to null, because later evidence is "non-inferior" to it.
Chemotherapy for DS TB all ages	93%*	culture negative at end of treatment	For TB treatment, the 2022 recommendation[21] is the same as 2010, and the sources are the same: Jindani et al. (2004) 6 vs 2 alternative 8-month duration,[37] and on Menzies et al. (2009) on shorter vs 6 months duration.[38] Jindani reports 85% culture negative at 2 months and 93% at end of treatment for isoniazid, rifampin, pyrazinamide, and either ethambutol or streptomycin for 2 months and isoniazid and rifampin for 4 months (2HRZE/4HR). Intervention was DOTS at health facility during intensive phase and DOTS under supervision of treatment monitor during continuation phase. Han et al. (2006) reported 87% success rate, but wasn't cited by WHO, "Treatment with 6-month daily regimens was effective for new TB patients with success rate (percentage of cure cases and treatment completed cases) of over 80%.[39]
Legend: * denotes parameter was selected in final model and estimate is used in predictions, adult = ages 10 or more years, ART = Antiretroviral therapy, BCG = Bacille Calmette-Guerin, DS= drug susceptible, DALYs=disability-adjusted life-years, IRS = Indoor residual spraying, DOTS=directly observed therapy, INSTI=integrase strand transfer inhibitors, IPT = Intermittent preventive treatment for malaria, MSM = Men who have sex with men, NRTI=nucleoside reverse transcriptase inhibitors, NNRTI=non-nucleoside reverse transcriptase inhibitors, Option B+ = HIV screening for pregnant women and lifelong ART, PI=protease inhibitors, PREP = Pre-exposure prophylaxis, RR=relative risk, TB = Tuberculosis,			

6.5 Logistic regression estimates

An ICER for a cost-saving intervention that decreases cost and improves health outcomes is not meaningful,[40] so we estimated the probability that an intervention is cost-saving. In this analysis, one meant that the cost-effectiveness result was cost saving with a decrease in cost and increase in DALYs or QALYs averted, and zero meant that the result was in the ICER quadrant with an increase in cost and increase in DALYs or QALYs averted. We used a mixed-effect logistic regression model to estimate the probability. The models used the same covariates as the meta-regression model when possible, and estimated a random intercept for each article. Estimates were performed for three interventions that had at least two cost-saving results and complete data for the covariates: ART for prevention, IPT for infants, and bed nets.

For those three interventions, the reported ICER was the product of the predicted ICERs and predicted probability that the result is in the ICER quadrant, i.e. one minus the predicted probability that it is cost-saving. We used the same probability for a country and intervention to calculate the adjusted ICER for the median, interquartile range, and 95% UI.

Section 6 References

1 Rosettie KL, Joffe JN, Sparks GW, *et al.* Cost-effectiveness of HPV vaccination in 195 countries: A meta-regression analysis. *PloS One* 2021; **16**: e0260808.

2 Janko MM, Joffe J, Michael D, *et al.* Cost-effectiveness of rotavirus vaccination in children under five years of age in 195 countries: A meta-regression analysis. *Vaccine* 2022; **40**: 3903–17.

3 Marseille E, Saba J, Muyingo S, Kahn JG. The costs and benefits of private sector provision of treatment to HIV-infected employees in Kampala, Uganda. *AIDS Lond Engl* 2006; **20**: 907–14.

4 Pitter C, Kahn JG, Marseille E, *et al.* Cost-effectiveness of cotrimoxazole prophylaxis among persons with HIV in Uganda. *J Acquir Immune Defic Syndr 1999* 2007; **44**: 336–43.

5 Ryan M, Griffin S, Chitah B, *et al.* The cost-effectiveness of cotrimoxazole prophylaxis in HIV-infected children in Zambia. *AIDS Lond Engl* 2008; **22**: 749–57.

6 World Health Organization. Consolidated guidelines on HIV prevention, testing, treatment, service delivery and monitoring: recommendations for a public health approach. 2021. https://www.who.int/publications-detail-redirect/9789240031593 (accessed Sept 12, 2022).

7 The Global Fund. Health Product Procurement: Antiretrovirals. https://www.theglobalfund.org/en/sourcing-management/health-products/antiretrovirals/ (accessed Feb 22, 2022).

8 World Health Organisation. WHO guideline on syphilis screening and treatment for pregnant women. https://www.who.int/publications-detail-redirect/9789241550093 (accessed May 10, 2023).

9 UNICEF. Supply Catalogue: Bioline Syphilis 3.0, kit/30. https://supply.unicef.org/s0004046.html (accessed May 10, 2023).

10 World Health Organization. WHO Guidelines for malaria. https://www.who.int/publications-detail-redirect/guidelines-for-malaria (accessed Sept 12, 2022).

11 The Global Fund. Health Product Procurement: Long-Lasting Insecticidal Nets. https://www.theglobalfund.org/en/sourcing-management/health-products/long-lasting-insecticidal-nets/ (accessed Oct 24, 2023).

12 Baral R, Levin A, Odero C, *et al.* Costs of continuing RTS,S/ASO1E malaria vaccination in the three malaria vaccine pilot implementation countries. *PLOS ONE* 2021; **16**: e0244995.

13 The Global Fund. Health Product Procurement: Antimalarial Medicines. https://www.theglobalfund.org/en/sourcing-management/health-products/antimalarial-medicines/ (accessed Oct 24, 2023).

14 Sherrard-Smith E, Griffin JT, Winskill P, *et al.* Systematic review of indoor residual spray efficacy and effectiveness against Plasmodium falciparum in Africa. *Nat Commun* 2018; **9**: 4982.

15 World Health Organization. WHO consolidated guidelines on tuberculosis: module 3: diagnosis: rapid diagnostics for tuberculosis detection, 2021 update. 2021. https://www.who.int/publications-detail-redirect/9789240029415 (accessed Sept 12, 2022).

16 Stop TB Partnership GDF. Diagnostics, Medical Devices & Other Health Products. 2022; published online Sept. https://www.stoptb.org/sites/default/files/gdfdiagnosticsmedicaldevotherhealthproductscatalog_0.pdf (accessed Oct 24, 2023).
17 World Health Organisation. WHO recommendations for routine immunization - summary tables. https://www.who.int/teams/immunization-vaccines-and-biologicals/policies/who-recommendations-for-routine-immunization---summary-tables (accessed Oct 24, 2023).
18 World Health Organisation. 2020 WHO Global Vaccine Market Report. https://www.who.int/publications/m/item/2020-who-global-vaccine-market-report (accessed Oct 24, 2023).
19 Abubakar I, Pimpin L, Ariti C, *et al.* Systematic review and meta-analysis of the current evidence on the duration of protection by bacillus Calmette-Guérin vaccination against tuberculosis. *Health Technol Assess Winch Engl* 2013; **17**: 1–372, v–vi.
20 World Health Organisation. WHO consolidated guidelines on tuberculosis: module 1: prevention: tuberculosis preventive treatment. 2020; published online Feb 25. https://www.who.int/publications-detail-redirect/9789240001503 (accessed Oct 24, 2023).
21 WHO consolidated guidelines on tuberculosis: module 4: treatment: drug-susceptible tuberculosis treatment. https://www.who.int/publications-detail-redirect/9789240048126 (accessed Oct 13, 2022).
22 Sturt AS, Dokubo EK, Sint TT. Antiretroviral therapy (ART) for treating HIV infection in ART-eligible pregnant women. *Cochrane Database Syst Rev* 2010. DOI:10.1002/14651858.CD008440.
23 Kanters S, Vitoria M, Zoratti M, *et al.* Comparative efficacy, tolerability and safety of dolutegravir and efavirenz 400mg among antiretroviral therapies for first-line HIV treatment: A systematic literature review and network meta-analysis. *EClinicalMedicine* 2020; **28**: 100573.
24 Penazzato M, Prendergast AJ, Muhe LM, Tindyebwa D, Abrams E. Optimisation of antiretroviral therapy in HIV-infected children under 3 years of age. *Cochrane Database Syst Rev* 2014. DOI:10.1002/14651858.CD004772.pub4.
25 Turkova A, White E, Mujuru HA, *et al.* Dolutegravir as First- or Second-Line Treatment for HIV-1 Infection in Children. *N Engl J Med* 2021; **385**: 2531–43.
26 Okwundu CI, Uthman OA, Okoromah CA. Antiretroviral pre-exposure prophylaxis (PrEP) for preventing HIV in high-risk individuals. *Cochrane Database Syst Rev* 2012. DOI:10.1002/14651858.CD007189.pub3.
27 Rogozińska E, Kara-Newton L, Zamora JR, Khan KS. On-site test to detect syphilis in pregnancy: a systematic review of test accuracy studies. *BJOG Int J Obstet Gynaecol* 2017; **124**: 734–41.
28 Pryce J, Richardson M, Lengeler C. Insecticide-treated nets for preventing malaria. *Cochrane Database Syst Rev* 2018. DOI:10.1002/14651858.CD000363.pub3.
29 RTS,S Clinical Trials Partnership. Efficacy and safety of RTS,S/AS01 malaria vaccine with or without a booster dose in infants and children in Africa: final results of a phase 3, individually randomised, controlled trial. *Lancet Lond Engl* 2015; **386**: 31–45.
30 Radeva-Petrova D, Kayentao K, Kuile FO ter, Sinclair D, Garner P. Drugs for preventing malaria in pregnant women in endemic areas: any drug regimen versus placebo or no treatment. *Cochrane Database Syst Rev* 2014. DOI:10.1002/14651858.CD000169.pub3.
31 Esu EB, Oringanje C, Meremikwu MM. Intermittent preventive treatment for malaria in infants. *Cochrane Database Syst Rev* 2021. DOI:10.1002/14651858.CD011525.pub3.
32 Pluess B, Tanser FC, Lengeler C, Sharp BL. Indoor residual spraying for preventing malaria. *Cochrane Database Syst Rev* 2010. DOI:10.1002/14651858.CD006657.pub2.
33 Shapiro AE, Ross JM, Yao M, *et al.* Xpert MTB/RIF and Xpert Ultra assays for screening for pulmonary tuberculosis and rifampicin resistance in adults, irrespective of signs or symptoms. *Cochrane Database Syst Rev* 2021. DOI:10.1002/14651858.CD013694.pub2.
34 US Centers for Disease Control and Prevention. The Role of BCG Vaccine in the Prevention and Control of Tuberculosis in the United States A Joint Statement by the Advisory Council for the Elimination of Tuberculosis and the Advisory Committee on Immunization Practices. MMWR Recomm Rep. 1996. https://www.cdc.gov/mmwr/preview/mmwrhtml/00041047.htm (accessed Oct 25, 2023).
35 Roy A, Eisenhut M, Harris RJ, *et al.* Effect of BCG vaccination against Mycobacterium tuberculosis infection in children: systematic review and meta-analysis. *BMJ* 2014; **349**: g4643.

36 Sterling TR. Guidelines for the Treatment of Latent Tuberculosis Infection: Recommendations from the National Tuberculosis Controllers Association and CDC, 2020. *MMWR Recomm Rep* 2020; **69**. DOI:10.15585/mmwr.rr6901a1.
37 Jindani A, Nunn A, Enarson D. Two 8-month regimens of chemotherapy for treatment of newly diagnosed pulmonary tuberculosis: international multicentre randomised trial. *The Lancet* 2004; **364**: 1244–51.
38 Menzies D, Benedetti A, Paydar A, *et al.* Effect of Duration and Intermittency of Rifampin on Tuberculosis Treatment Outcomes: A Systematic Review and Meta-Analysis. *PLOS Med* 2009; **6**: e1000146.
39 Han T. Effectiveness of standard short-course chemotherapy for treating tuberculosis and the impact of drug resistance on its outcome. *Int J Evid Based Healthc* 2006; **4**: 101–17.
40 Stinnett AA, Mullahy J. Net health benefits: a new framework for the analysis of uncertainty in cost-effectiveness analysis. *Med Decis Mak Int J Soc Med Decis Mak* 1998; **18**: S68-80.

7. Results

7.1 Introduction

In seven sections below, we present seven results for the each of the cause-type groups:

1. Correlation matrix with the associations of the covariates with the dependent variable (log ICER) and covariates
2. Three figures for the selected meta-regression model presenting the: a) relationship between the log ICER and non-linear signal variable, which we refer to as the "GDP spline," for the selected model, b) scatter plot of the log predicted ICER and log GDP per capita for 128 countries, and c) scatter plot of the log predicted ICER and log observed ICER for the meta-regression model sample.
3. Model fit statistics for the set of eight models that included the selected meta-regression model
4. Model fit statistics for alternative trimming strategies for the selected meta-regression model
5. Estimated meta-regression coefficients for the selected meta-regression model
6. Estimated logistic regression coefficients for the probability that an intervention would be cost-saving
7. Number of cost-saving ratios by interventions, termed "keywords" in the code, if applicable

Note that we report the correlation matrix rather than a forest plot. A forest plot for a meta-analysis lists each study on the vertical axis and reports the effect size and confidence intervals relative to the scale on the horizontal axis. The size of the entry may be proportional to the number of participants. The effect size would be the dependent variable in a meta-regression analysis.

In light of the differences between a meta-analysis of sample data and a meta-regression analysis of ICERs, Table S2 reports a summary of articles in the analysis sample, and we report correlation matrices below. Table S2 lists every study, and the range and number of ICERs reported in it. An ICER represents the result of a single simulation rather than sample data, so the size of each entry in the forest plot would be the same. Each reported ICER represents a different assumption about the input data, so each ICER reported in a study is different by design.

Our objective is to explain the differences in ICERs across simulations rather than identify a single effect size that summarizes all results. The correlation matrices in Section S7 for each cause-type group describe the association between the ICER and each of the potential covariates. For example, the correlation between the log ICER and log GDP per capita is 0.80 for ART, and 0.02 for malaria prevention. The correlation matrix summarizes the associations, in lieu of reporting a forest plot for each potential covariate.

We also provide brief commentary on the results, when appropriate.

The final section S7.9 presents a table that lists the 25 interventions that were included in the meta-analysis sample, (n=756 ICERs from 118 articles, see table S6.3), whether or not the covariate for that intervention was selected in the final model, and whether or not the intervention was recommended in recent WHO guidelines and for which we predicted ICERs.

7.2 HIV/AIDS - antiretroviral therapy (ART)

	Log ICER	Log GDP per capita (2019	Time Horizon Magnitude	Payer or Sector	Log DALYS per capita	Discount Rate	Efficacy	Log per year or full	Costs Discount Rate	Log prevalence per capita
Log ICER	1									
Log GDP per capita (2019 USD)	0.8	1								
Time Horizon Magnitude	0.3	0.3	1							
Payer or Sector	-0.2	-0.3	-0.3	1						
Log DALYS per capita	-0.7	-0.7	-0.03	0.1	1					
Discount Rate	0.02	0.05	-0.04	0.09	-0.1	1				
Efficacy	-0.2	-0.1	-0.3	-0.08	0.1	-0.05	1			
Log per year or full intervention cost (2019 USD)	0.8	0.8	0.2	-0.2	-0.7	0.04	-0.06	1		
Costs Discount Rate	0.03	0.08	-0.01	0.08	-0.1	1.0	-0.06	0.04	1	
Log prevalence per capita	-0.5	-0.5	0.03	0.07	0.9	-0.1	0.06	-0.6	-0.08	1

Table S7.1. Correlation matrix of ICERs and covariates for the ART analyses sample (n=612)

For the ART, version 952 (Table S7.2) was the model with the best fit; keywords were preselected for the model and included in the estimates in step 2, in which outliers were identified and the spline was fit. In this version however, so many of the ICERs for two interventions were identified as outliers that they no longer met the inclusion criteria: 1) ART for HIV prevention, HIV testing, and 2) ART for HIV prevention. When an intervention no longer met the inclusion criteria, all of the ratios were dropped even if they weren't outliers. Notice that the sample sizes for the versions with the keywords included in the spline fit were 15 to 20 smaller than the other versions. ART for prevention is currently recommended by the Global Fund, and we needed a version that included it to predict their ICERs.

Among the models in which the keywords were not preselected, the spline was fit and outliers were identified without them in the estimates. Keywords were selected in step 3 with the other covariates according to the Lasso strategy. ART for HIV prevention, HIV testing was selected in these models, and Version 961 had the lowest upper to lower ratio of the predictions among all the versions, and across the version 961 outlier experiments.

Alternate model fit statistics without sensitivity analysis

Ver-sion	Burden measure	Monotonicity imposed	Keywords in spline fit	R2	RMSE	Predicted mean ICER			Ratio upper lower mean			Mean from data		Sample size
						Adult	Child	PMTCT	Adult	Child	PMTCT	ART	PMTCT	
952	DALYs	No	Yes	0.9	0.9	NA	NA	2050	NA	NA	45	NA	2206	238
953	DALYs	No	No	0.8	1.2	3722	4771	1794	84	83	98	18,227	2206	257
954	DALYs	Yes	Yes	0.8	0.9	NA	NA	2545	NA	NA	55	NA	2206	243
955	DALYs	Yes	No	0.8	1.2	7342	9358	3633	124	126	143	18,357	2206	257
960	Prev	No	Yes	0.9	0.9	NA	NA	2116	NA	NA	51	NA	2206	238
961*	Prev	No	No	0.8	1.2	2807	4273	1461	70	77	80	18,227	2206	258
962	Prev	Yes	Yes	0.9	0.9	NA	NA	2116	NA	NA	51	NA	2206	238
963	Prev	Yes	No	0.8	1.2	4634	6834	2782	101	112	115	18, 357	2206	258

Table S7.2. Model fit statistics for set of eight models with no crosswalks or sensitivity analyses. (*) denotes selected model

Outliers dropped	Burden measure	Monotonicity imposed	Keywords in spline fit	R2	RMSE	Predicted mean ICER			Ratio upper lower mean			Mean from data		Sample size
						Adult	Child	PMTCT	Adult	Child	PMTCT	ART	PMTCT	
10% *	Prev	No	No	0.8	1.2	2807	4273	1461	70	77	80	18,227	2206	258
5%	Prev	No	No	0.8	1.1	2189	3204	5357	92	91	137	18,357	2739	271
None	Prev	No	No	0.7	1.4	4784	6129	5239	445	432	831	158,126	2739	286

Table S7.3. Model fit statistics for alternative trimming strategies for the selected model: 10%, 5% and none

Figure S7.1 ART signal and log GDP per capita

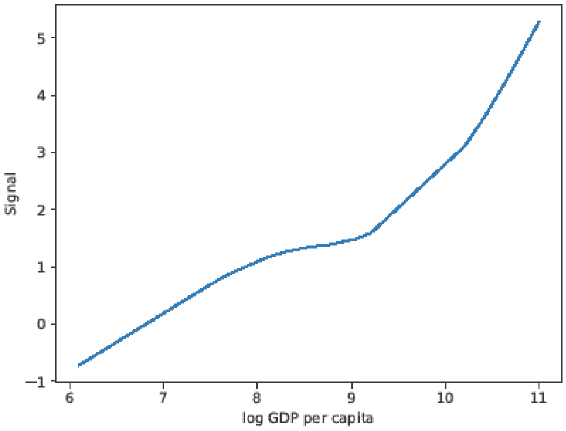

Figure S7.2 ART log predicted ICER and log GDP per capita

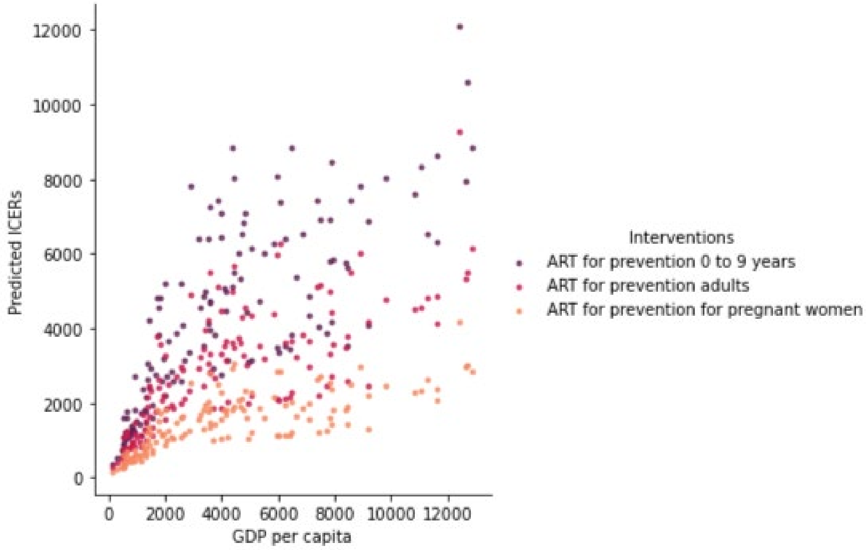

Figure S7.3 ART published and predicted log ICERs

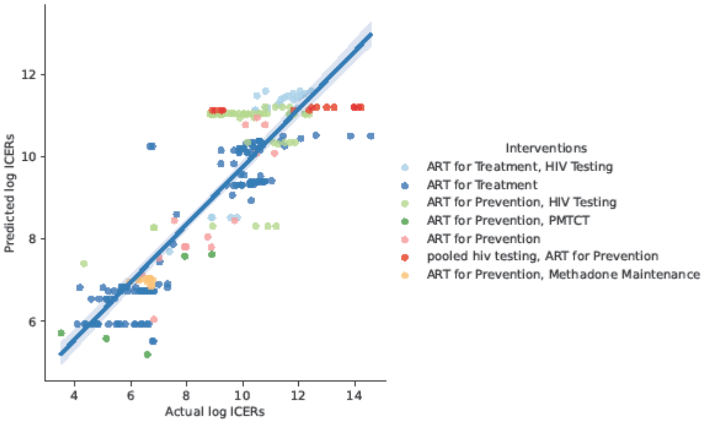

For the intervention “pooled hiv testing, ART for prevention” in Figure S7.3, the range of published log ICERs or training data on the horizontal axis is wider than the range of predicted log ICERs on the vertical axis. The training data were from two articles in two different high income countries, as one can see from the slight difference in the predicted ICERs. The training data for one of the articles reported ICERs from several simulations of different pooled hiv testing strategies and test costs. These covariates were not included in the model, because they were not reported in many of the other ART articles. Consequently, the model predicted only a single ICER for each country based on its GDP per capita, log prevalence per capita, and log cost per year of ART.

Covariate	$\hat{\beta}$	$\widehat{SE}[\hat{\beta}]$	$\hat{\gamma}$
Intercept - ART for HIV for prevention	5.5	0.5	1.1
New Spline Covariate	0.8	0.1	
ART for HIV, HIV testing	0.5	0.5	
ART for HIV for prevention, HIV testing	0.3	0.1	
ART for HIV	-0.4	0.3	
Pooled HIV testing, antiretroviral therapy for HIV for prevention	0.4	0.2	
Prevention of mother to child HIV transmission, ART for HIV for prevention	-0.4	0.3	
ART for prevention, Methadone Maintenance Therapy	-0.5	0.3	
Log per year or full intervention cost	0.05	0.03	
Log Prevalence per Capita	-0.1	0.05	
Second line	2.3	0.61	
	R^2	RMSE	Sample Size
Sample with Sensitivity Analyses			
Fixed and Random Effects	0.9	0.6	258
Fixed Effects Only	0.8	1.2	
Ratio of upper bound to lower bound of uncertainty interval for predicted ICERs			
ART for adults	70		
ART for under 10 years old	77		
Prevention of mother to child HIV transmission, ART for prevention	80		
Table S7.4. Parameter estimates and fit statistics for selected meta-regression model for HIV ART			
$\hat{\gamma}$ is the between study heterogeneity in the meta-regression analysis.			

Variable	Estimate	Standard error	Z value	P value
Intercept	-13.9	17.6	-0.8	0.4
Log GDP per capita	0.7	2.3	0.3	0.8
Log prevalence per capita	0.07	1.5	0.05	1.0
Log per year or full intervention cost	-0.6	1.6	-0.4	0.7
Second line	2.7	8.4	0.3	0.7
ART for HIV (treatment)*	-1.0	7.3	-0.1	0.9
ART for HIV for prevention, HIV testing*	-0.4	9.1	-0.04	1.0
Pooled HIV testing, ART for HIV for prevention*	0.5	12.3	0.4	1.0

Table S7.5. Logistic regression analysis of the probability that an intervention was cost-saving (n=240)**

*Cost saving adjustments are only applied to keywords with cost saving ratios.
** Sample size includes n=22 of 28 cost saving ratios. Cost data were missing for six ratios.

Intervention keyword	Cost saving ratios
ART for HIV	13
Pooled HIV testing, ART for hiv for prevention	4
ART for HIV for prevention	3
ART for HIV for prevention, HIV testing	2
Total cost saving ratios	22

Table S7.6: Numbers of cost saving ratios by intervention keyword

7.3 HIV/AIDS Pre-exposure prophylaxis (PREP)

	Log ICER	Log GDP per capita (2019	Time Horizon Magnitude	Log DALYS per capita	Discount Rate	Efficacy	Log per year or full intervention	Costs Discount Rate	Log prevalence per capita
Log ICER	1								
Log GDP per capita (2019 USD)	0.3	1							
Time Horizon Magnitude	0.3	0.4	1						
Log DALYS per capita	-0.4	-0.7	-0.3	1					
Discount Rate	0.03	0.2	0.02	0.3	1				
Efficacy	-0.5	-0.2	-0.4	-0.1	-0.2	1			
Log per year or full intervention cost (2019 USD)	0.5	0.9	0.4	-0.8	-0.2	-0.2	1		
Costs Discount Rate	-0.2	-0.03	0.005	0.05	0.2	-0.03	-0.04	1	
Log prevalence per capita	-0.4	-0.5	-0.15	0.9	0.3	-0.2	-0.6	0.05	1

Table S7.7. Correlation matrix of ICERs and covariates for the HIV PREP analyses sample (n=86)

Version	Burden measure	Monotonicity imposed	Keywords in spline fit**	R2	RMSE	Predicted mean ICER		Ratio upper lower mean		Mean from data	Sample size
						MSM	Hetero	MSM	Hetero		
1026	DALYs	No	NA	0.5	1.2	39,443	30,607	178	174	32,729	76
1027_o5*	DALYs	Yes	NA	0.5	1.2	23,406	18,842	98	98	31,683	80
1030	Prev	No	NA	0.5	1.2	65,338	46,225	228	208	33,070	77
1031	Prev	Yes	NA	0.5	1.1	86,223	60,076	266	279	33,070	76

Table S7.8. Model fit statistics for set of four models with sensitivity analyses, and no crosswalks.

* denotes selected model – model with outliers dropped of 5% as this was a better model than the original best version of 1027

** Experiments with including keyword in spline are not applicable to cause-type group with one keyword

Outliers dropped	Burden measure	Monotonicity imposed	Keywords in spline fit	R2	RMSE	Predicted mean ICER		Ratio upper lower mean		Mean from data	Sample size
						MSM	Hetero	MSM	Hetero		
10%	DALYs	No	NA	0.5	1.2	28,913	22,601	108	107	32,876	76
*5%	DALYs	No	NA	0.5	1.2	23,406	18,842	98	98	31,683	80
None	DALYs	No	NA	0.4	1.3	18,837	15,687	121	122	30,054	85

Table S7.9. Model fit statistics for alternative trimming strategies for the selected model: 10%, 5% and none

Figure S7.4 PREP signal and log GDP per capita

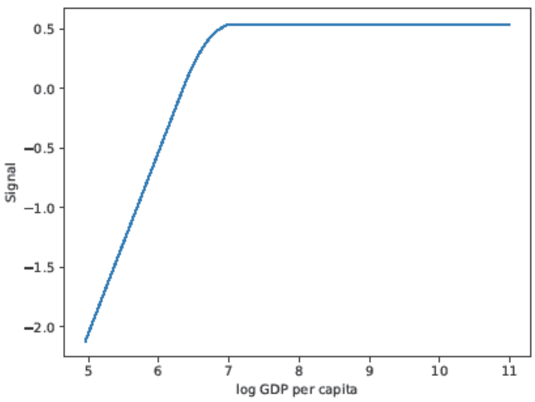

Figure S7.5 PREP log predicted ICER and log GDP per capita

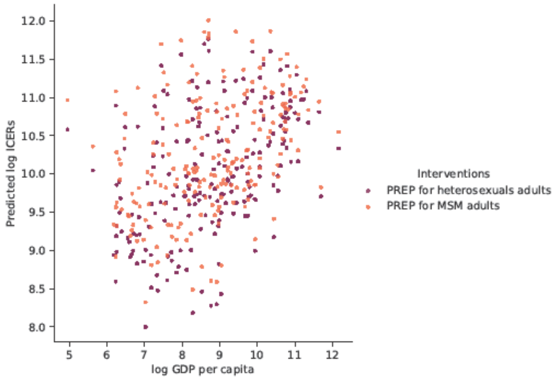

Figure S7.6 PREP published and predicted log ICERs

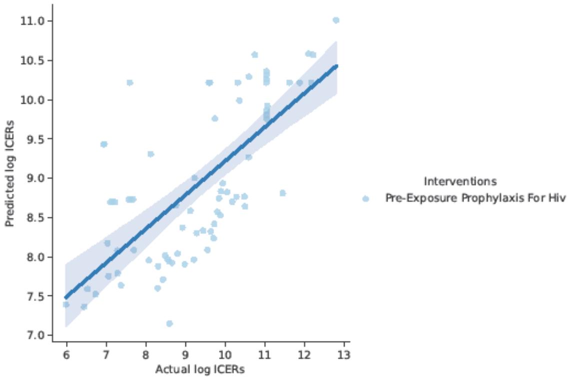

In contrast to Figure S7.3 for ART, Figure S7.6 does not show a broad range of training data for a single predicted ICER. For PREP when the articles reported ICERs for different risk HIV risk groups, we were able to extract data on two risk group (MSM adults or heterosexual adults) from every article. We included that covariate in the model and predicted ICERs for both risk groups.

Covariate	$\hat{\beta}$	$\widehat{SE}[\hat{\beta}]$	$\hat{\gamma}$
Intercept - Heterosexuals	8.7	0.5	1.2
New Spline Covariate	-0.5	0.07	
Log DALYs per Capita	-0.4	0.002	
Efficacy	-0.03	0.003	
MSM risk group	0.01	0.1	
Log per year or full intervention cost	0.09	0.03	
	R^2	RMSE	Sample size
Sample with Sensitivity Analyses			
Fixed and Random Effects	0.7	0.9	80
Fixed Effects Only	0.5	1.2	
Ratio of upper bound to lower bound of uncertainty interval for predicted ICERs			
Pre-exposure prophylaxis for heterosexual adults	98		
Pre-exposure prophylaxis for adults who have male to male sex	98		
Table S7.10. Parameter estimates and fit statistics for selected meta-regression model for HIV PREP			
$\hat{\gamma}$ is the between study heterogeneity in the meta-regression analysis.			

We did not estimate the probability that PREP would be cost-saving, because there was one cost saving ratio. It was based on a sensitivity analysis of cost.

7.4 Syphilis Diagnostics

	Log ICER	Log GDP per capita (2019 USD)	Time Horizon Magnitude	Payer or Sector	Log DALYS per capita	Efficacy	Log per year or full intervention cost (2019 USD)	Costs Discount Rate
Log ICER	1							
Log GDP per capita (2019 USD)	0.22	1						
Time Horizon Magnitude	0.5	0.4	1					
Payer or Sector	-0.2	0. 02	-0.4	1				
Log DALYS per capita	-0.5	-0.4	-0.5	-0.08	1			
Efficacy	0.4	0.3	1.0	-0.4	-0.4	1		
Log per year or full intervention cost (2019 USD)	0.06	-0.06	-0.3	-0.2	-0.2	-0.2	1	
Costs Discount Rate	0.3	-0.03	0.3	-0.3	0.1	0.2	-0.5	1
Table S7.11. Correlation matrix of ICERs and covariates for the ante-natal syphilis screening analyses sample (n=222)								

Ver-sion	Burden measure ***	Monotonicity imposed	Keywords in spline fit**	R2	RMSE	Predicted mean ICER	Ratio upper lower mean	Mean from data	Sample size
1026*	DALYs	Yes	NA	0.5	0.7	162	10	24	111
1028	DALYs	No	NA	0.4	0.7	32	17	23	150

Table S7.12 Model fit statistics for set of two models with sensitivity analyses, and no crosswalks

* denotes selected model
** Experiments with including keyword in spline are not applicable to cause-type group with one keyword
*** There are no prevalence data for neonatal syphilis

There is no prevalence data for syphilis

Outliers	Burden measure	Monotonicity imposed	Keywords in spline fit	R2	RMSE	Predicted mean ICER	Ratio upper lower mean	Mean from data	Sample size
10%	DALYs	Yes	NA	0.5	0.7	162	10	24	111
5%	DALYs	Yes	Yes	0.5	0.8	278	22	26	118
None	DALYs	Yes	Yes	0.1	1.0	164	109	27	215

Table S7.13 Model fit statistics for alternative trimming strategies for the selected model: 10%, 5% and none

Figure S7.7 Syphilis signal and log GDP per capita

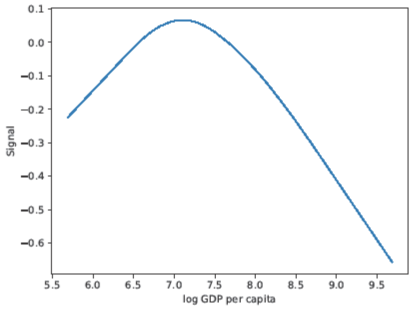

Figure S7.8 Syphilis log predicted ICER and log GDP per capita

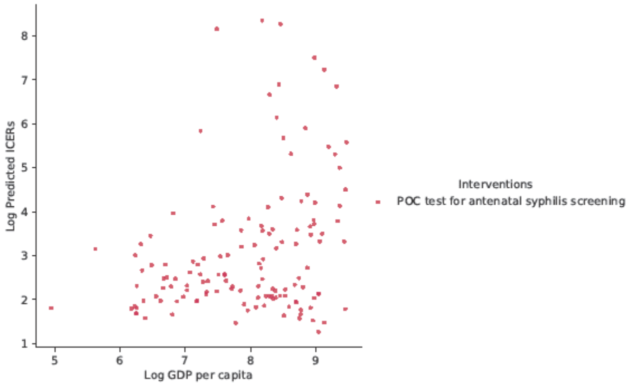

Figure S7.9 Syphilis published and predicted log ICERs

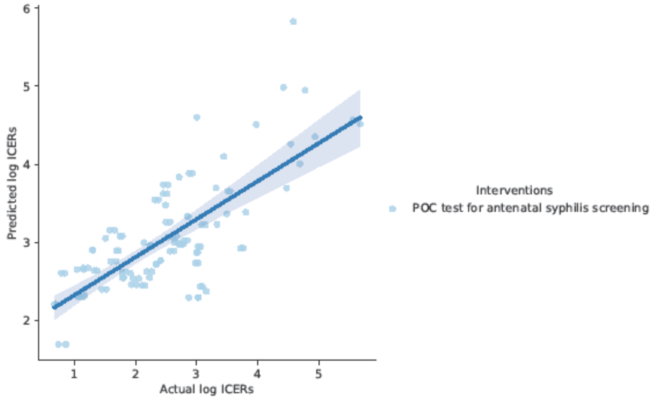

Covariate*	$\hat{\beta}$	$\widehat{SE}[\hat{\beta}]$	$\hat{\gamma}$
Intercept	0.9	0.5	0.3
New Spline Covariate	0.8	0.2	
Log DALYs per capita	-0.5	0.02	
Sensitivity	0.0	0.005	
	R^2	RMSE	Sample Size
Sample with Sensitivity Analyses			
Fixed and Random Effects	0.6	0.7	111
Fixed Effects Only	0.5	0.7	
Ratio of upper bound to lower bound of uncertainty interval for predicted ICERs			
Antenatal screening and antibiotics			10
Table S7.14. Parameter estimates and fit statistics for selected meta-regression model for syphilis diagnostics			
$\hat{\gamma}$ is the between study heterogeneity in the meta-regression analysis.			

We did not estimate the probability that antenatal syphilis screening would be cost-saving, because the two cost saving ratios were missing cost data and were dropped from the analysis.

7.5 Malaria Prevention

	Log ICER	Log GDP per capita (2019 USD)	Time Horizon Magnitude	Payer or Sector	Log DALYS per capita	Discount Rate	Efficacy	Log per year or full intervention cost	Costs Discount Rate	Log prevalence per capita
Log ICER	1									
Log GDP per capita (2019 USD)	0.02	1								
Time Horizon Magnitude	-0.3	-0.2	1							
Payer or Sector	-0.06	-0.03	0.1	1						
Log DALYS per capita	-0.4	-0.3	0.3	-0.1	1					
Discount Rate	0.1	-0.009	0.1	-0.2	0.2	1				
Efficacy	0.4	0.2	-0.5	-0.008	-0.3	-0.01	1			
Log per year or full intervention cost (2019 USD)	0.3	0.05	-0.005	0.2	0.07	-0.07	0.3	1		
Costs Discount Rate	-0.02	-0.1	0.05	0.06	0.4	0.4	-0.2	-0.06	1	
Log prevalence per capita	-0.4	-0.4	0.3	0.07	0.2	0.2	-0.3	-0.03	0.20	1

Table S7.15. Correlation matrix of ICERs and covariates for malaria prevention analyses sample (n=148)

Version	Burden measure	Monotonicity imposed	Keywords in spline fit	R2	RMSE	Predicted mean ICER					Ratio upper lower mean					Mean from data					Sample size
						Bed nets	Vacc	IRS	IPT infants	IPT preg	Bed nets	Vacc	IRS	IPT infants	IPT preg	Bed nets	Vacc	IRS	IPT infants	IPT preg	
704	DALYs	Yes	Yes	0.8	0.9	346	6820	NA	388	NA	73	83	NA	74	NA	36	446	NA	19	NA	56
705	DALYs	Yes	No	0.7	0.9	280	3284	5006	206	NA	109	127	116	95	NA	36	459	443	19	NA	81
706	DALYs	No	Yes	0.8	0.9	529	7194	NA	466	NA	94	122	NA	102	NA	36	446	NA	19	NA	56
707	DALYs	No	No	0.5	1.0	567	2425	5113	365	NA	72	84	100	78	NA	36	463	443	68	NA	82
1029 *	Prev	Yes	Yes	0.8	0.9	5937	3058	NA	2445	1209	9	9	NA	10	9	36	434	NA	17	11	74
1030	Prev	Yes	No	0.7	1.1	3097	2366	5925	1371	596	48	51	61	57	57	36	459	440	19	12	96
1031	Prev	No	Yes	0.8	0.9	7527	4608	NA	5084	2310	9	9	NA	10	10	36	439	NA	17	11	73
1032	Prev	No	No	0.7	1.1	2697	1782	2768	1183	548	38	40	45	42	53	36	456	440	17	11	97

Table S7.16. Model fit statistics for set of eight models with no crosswalks or sensitivity analyses. (*) denotes selected model

Outliers dropped	Burden measure	Monotonicity imposed	Keywords in spline fit	R2	RMSE	Predicted mean ICER					Ratio upper lower mean					Mean from data					Sample size
						Bed nets	Vacc	IRS	IPT infants	IPT preg	Bed nets	Vacc	IRS	IPT infants	IPT preg	Bed nets	Vacc	IRS	IPT infants	IPT preg	
10%	Prev	Yes	Yes	0.8	0.9	5937	3058	NA	2445	1209	9	9	NA	10	9	36	434	NA	17	11	74
5%	Prev	Yes	Yes	0.7	1.0	2184	1153	2579	653	316	28	32	34	36	37	36	419	443	17	11	103
None	Prev	Yes	Yes	0.6	1.2	521	528	2399	177	57	36	41	50	46	42	36	729	443	54	11	110

Table S7.17. Model fit statistics for alternative trimming strategies for the selected model: 10%, 5% and none

Figure S7.10 Malaria signal and log GDP per capita

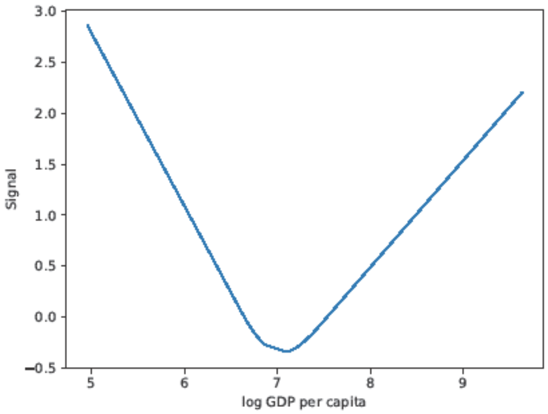

Figure S7.11 Malaria log predicted ICER and log GDP per capita

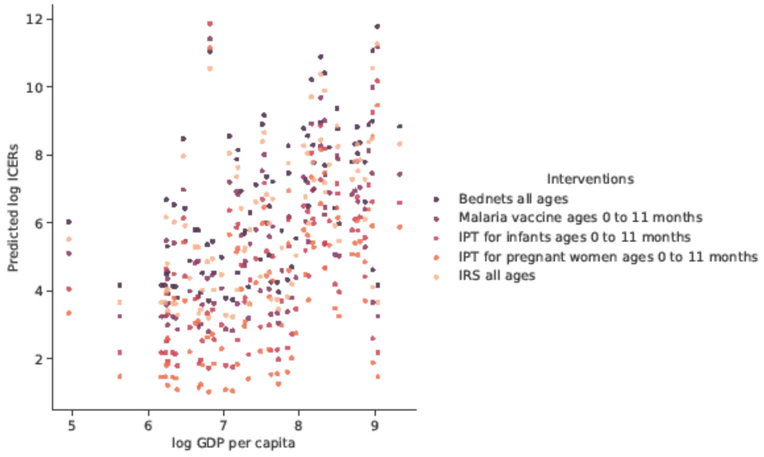

Figure S7.12 Malaria published and predicted log ICERs

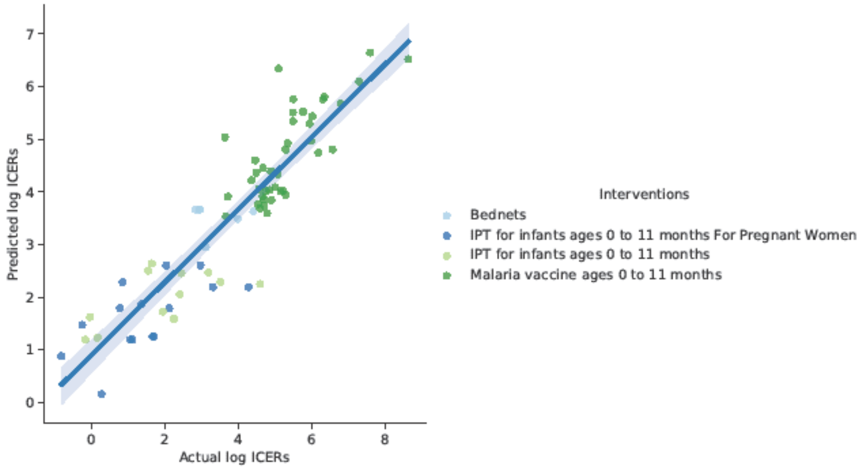

Covariate*	$\hat{\beta}$	$\widehat{SE}[\hat{\beta}]$	$\hat{\gamma}$
Intercept - malaria vaccines	1.3	0.4	0.2
New Spline Covariate	0.2	0.05	
Bed Nets	0.7	0.4	
Malaria Intermittent Preventive Treatment for pregnant women	-2.5	0.4	
Malaria intermittent preventive treatment	-1.2	0.4	
Efficacy	0.02	0.004	
Log Prevalence per Capita	-0.8	0.03	
	R^2	RMSE	Sample Size
Sample with Sensitivity Analyses			
Fixed and Random Effects	0.9	0.8	74
Fixed Effects Only	0.8	0.9	
Ratio of upper bound to lower bound of uncertainty interval for predicted ICERs			
Bed nets			9
Malaria vaccines			9
Intermittent preventive treatment (IPT) for infants			10
IPT for pregnant women			10
Table S7.18. Parameter estimates and fit statistics for selected meta-regression model for malaria prevention			
$\hat{\gamma}$ is the between study heterogeneity in the meta-regression analysis.			

Variable	Estimate	Standard error	Z value	P value
Intercept (Bed nets)*	8.5	11.2	0.8	0.4
Log prevalence per capita	0.06	1.7	0.04	1.0
Log GDP USD per capita	-1.4	1.7	-0.9	0.4
Intermittent preventive treatment (IPT) for infants*	-2.5	3.8	-0.7	0.5
Table S7.19. Logistic regression analysis of the probability that an intervention was cost-saving (n=11) and total sample of 29 (15 bed nets and 14 IPT).				
*Cost saving adjustments are only applied to keywords with cost saving ratios.<br>** Sample size includes n=11 of 20 cost saving ratios. One cost saving ratio for IPT for pregnant women was dropped. Four cost-saving ratios for malaria vaccines were dropped, because they were sensitivity analyses for the cost variable, which was not selected in the meta-regression analysis. Four other cost saving ratios were dropped as they were for the indoor residual spraying intervention, of which there was no ratios for in our final dataset used for the model fit.				

Intervention keyword	Cost saving ratios
Bed nets	9
IPT for infants	2
Total cost saving ratios in logistic regression	11
Table S7.20. Numbers of cost saving ratios by intervention keyword	

7.6 Tuberculosis Prevention

	Log ICER	Log GDP per capita (2019 USD)	Time Horizon Magnitude	Payer or Sector	Log DALYS per capita	Discount Rate	Efficacy	Log per year or full intervention cost (2019 USD)	Costs Discount Rate	Log prevalence per capita
Log ICER	1									
Log GDP per capita (2019 USD)	0.7	1								
Time Horizon Magnitude	0.04	0.2	1							
Payer or Sector	-0.3	-0.3	-0.8	1						
Log DALYS per capita	-0.8	-0.9	-0.1	0.3	1					
Discount Rate	-0.04	-0.04	0.1	-0.9	0.2	1				
Efficacy	0.3	0.2	0.2	-0.5	-0.3	-0.1	1			
Log per year or full intervention cost (2019 USD)	0.4	0.2	0.6	-0.6	-0.2	-0.09	0.3	1		
Costs Discount Rate	-0.1	-0.06	0.2	-0.2	0.2	1.0	-0.1	0.2	1	
Log prevalence per capita	-0.04	0.5	0.1	-0.3	-0.3	0.1	0.3	-0.2	0.2	1

Table S7.21. Correlation matrix of ICERs and covariates for the tuberculoisis prevention analyses sample (n=62)

Model statistics

Version	Burden measure	Monotonicity imposed	Keywords in spline fit	R2	RMSE	Predicted mean ICER		Ratio upper lower mean		Mean from data		Sample size
						Prophylaxis for people without active TB	BCG vaccine	Prophylaxis for people without active TB	BCG vaccine	Prophylaxis for people without active TB	BCG vaccine	
600	DALYs	Yes	Yes	0.6	1.2	NA	1935	NA	531	NA	3589	18
601	DALYs	Yes	No	0.5	1.5	NA	36,391	NA	23258	NA	3589	19
602*	DALYs	No	Yes	0.8	0.8	3074	2382	35	49	75,173	3589	20
603	DALYs	No	No	0.8	0.9	2566	1559	32	41	75,173	3589	20
604	Prev	Yes	Yes	0.7	1.0	NA	262	NA	73	NA	3589	18
605	Prev	Yes	No	0.6	1.3	1575	641	880	1517	55,907	4756	22
606	Prev	No	Yes	0.6	1.0	NA	419	NA	165	NA	3589	18
607	Prev	No	No	0.6	1.3	1679	693	952	1656	55,907	4756	22

Table S7.22**.** Model fit statistics for set of eight models with sensitivity analyses and no crosswalks. (*) denotes selected model

Outliers	Burden measure	Monotonicity imposed	Keywords in spline fit	R2	RMSE	Predicted mean ICER		Ratio upper lower mean		Mean from data		Sample size
						Prophylaxis for people without active TB	BCG vaccine	Prophylaxis for people without active TB	BCG vaccine	Prophylaxis for people without active TB	BCG vaccine	
10%	DALYs	No	Yes	0.8	0.8	3074	2382	35	49	75,173	3589	20
5%	DALYs	No	Yes	0.8	0.9	3054	2210	27	37	75,173	3589	21
None	DALYs	No	Yes	0.6	1.3	2436	1268	566	1268	75,173	3589	23

Table S7.23**.** Model fit statistics for alternative trimming strategies for the selected model: 10%, 5% and none

Figure S7.13 Tuberculosis Prevention signal and log GDP per capita

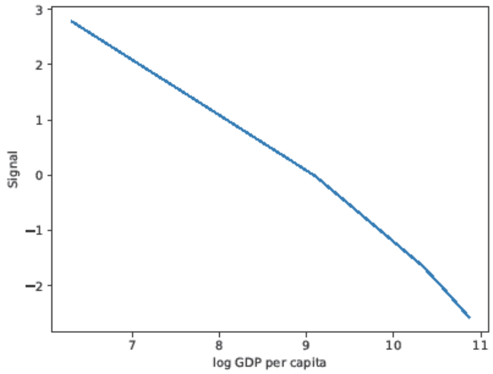

Figure S7.14 Tuberculosis prevention log predicted ICER and log GDP per capita

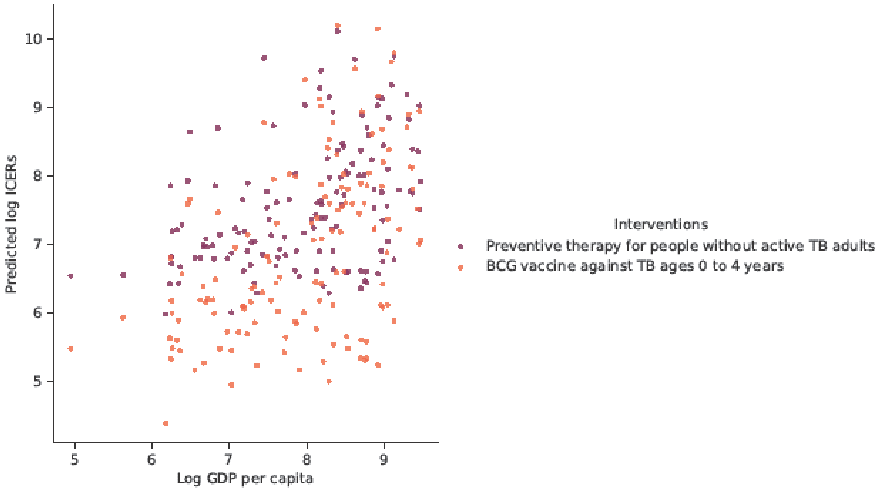

Figure S7.15 Tuberculosis prevention published and predicted log ICERs

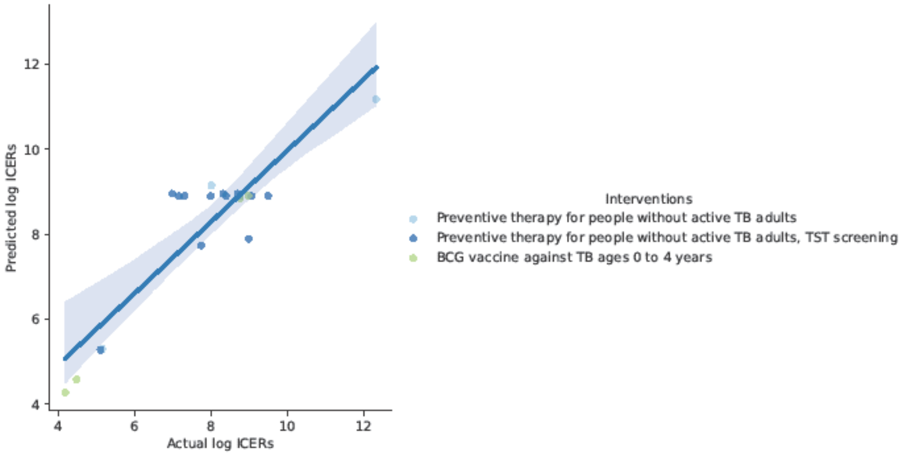

Similar to Figure S7.3 for ART, Figure S7.15 shows a broad range of training data for a single predicted ICER. For Preventive therapy for people without active TB Adults, TST screening, one article reported reported ICERs for

several different immigrant groups. Covariates for immigrant groups weren't included in the model, because they weren't relevant to other articles for that intervention nor to other TB prevention interventions.

Covariate - Prophylasis for people without active TB, TST	$\hat{\beta}$	$\widehat{SE}[\hat{\beta}]$	$\hat{\gamma}$
Intercept	2.7	0.983	0.4
New Spline Covariate	0.1	0.270	
Prophylaxis for people without active TB	0.2	0.221	
Tuberculosis vaccines	-1.4	0.611	
Efficacy	0.01	0.007	
Log DALYs per capita	-0.7	0.11	
	R^2	RMSE	Sample Size
Sample with Sensitivity Analyses			
Fixed and Random Effects	0.9	0.6	20
Fixed Effects Only	0.8	0.8	
Ratio of upper bound to lower bound of uncertainty interval for predicted ICERs			
Prophylaxis for people without active tuberculosis (TB)			35
TB vaccines			49
Table S7.24. Parameter estimates and fit statistics for selected meta-regression model for tuberculosis prevention			
^ is the between study heterogeneity in the meta-regression analysis. TST=screening with tuberculin skin test			

There were no cost saving ratios for the interventions prophylaxis for people without active TB and tuberculosis vaccines, so no logistics regression was run.

7.7 Tuberculosis Diagnostics

	Log ICER	Log GDP per capita (2019 USD)	Time Horizon Magnitude	Payer or Sector	Log DALYS per capita	Discount Rate	Efficacy	Log per year or full intervention	Costs Discount Rate	Log prevalence per capita
Log ICER	1									
Log GDP per capita (2019 USD)	0.8	1								
Time Horizon Magnitude	0.3	0.3	1							
Payer or Sector	-0.07	0.03	-0.05	1						
Log DALYS per capita	-0.8	-0.9	-0.6	-0.06	1					
Discount Rate	0.1	0.08	0.1	-0.03	-0.06	1				
Efficacy	-0.1	-0.1	0.09	0.04	0.09	-0.01	1			
Log per year or full intervention cost (2019 USD)	0.1	0.2	0.1	0.03	-0.2	0.4	-0.07	1		
Costs Discount Rate	0.1	0.06	0.2	0.2	-0.09	0.7	0.02	0.3	1	
Log prevalence per capita	-0.03	-0.09	-0.3	-0.08	0.3	-0.03	0.04	-0.3	-0.05	1

Table S7.25. Correlation matrix of ICERs and covariates for the tuberculosis diagnostics sample (n=612) (n=133)

Version	Burden measure	Monotonicity imposed	Keywords in spline fit	R2	RMSE	Predicted mean ICER	Ratio upper lower mean	Ratio upper lower median	Mean from data	Sam-ple size
624	DALYs	Yes	Yes	0.9	1.2	885	43	42	550	60
625	DALYs	Yes	No	0.9	1.1	730	42	40	453	60
626	DALYs	No	Yes	0.9	1.1	701	52	51	420	70
627	DALYs	No	No	0.9	1.1	719	42	41	444	60
628*	Prev	Yes	Yes	0.9	1.0	1035	24	24	733	61
629	Prev	Yes	No	0.9	1.0	965	26	25	678	60
630	Prev	No	Yes	0.8	1.7	1903	704	687	327	70
631	Prev	No	No	0.9	1.0	967	27	26	362	60

Table S7.26. Model fit statistics for set of eight models with crosswalks and no sensitivity analyses. (*) denotes selected model

Version	Burden measure	Monotonicity imposed	Keywords in spline fit	R2	RMSE	Predicted mean ICER	Ratio upper lower mean	Ratio upper lower median	Mean from data	Sample size
10%*	Prev	Yes	Yes	0.92	0.98	1035	24	24	733	61
5% outliers	Prev	No	Yes	0.68	2.06	1680	1674	1666	362	74
zero	Prev	No	No	0.65	2.05	1129	6070	6020	343	78

Table S7.27. Model fit statistics for alternative trimming strategies for the selected model: 10%, 5% and none

Figure S7.16 Tuberculosis diagnostics signal and log GDP per capita

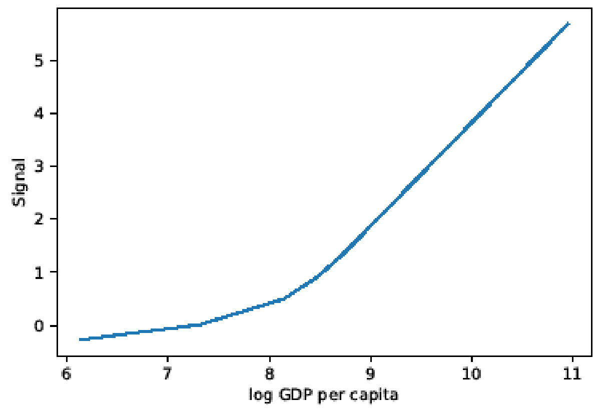

Figure S7.17 Tuberculosis diagnostics log predicted ICER and log GDP per capita

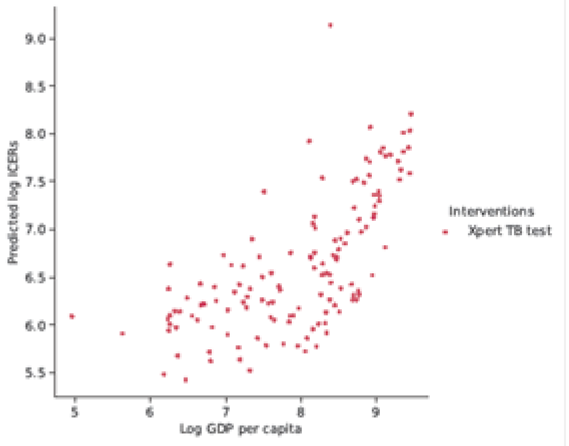

Figure S7.18 Tuberculosis diagnostics published and predicted log ICERs

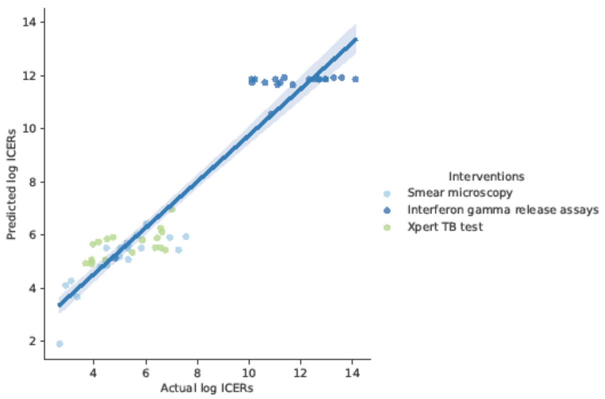

Covariate	$\hat{\beta}$	$\widehat{SE}[\hat{\beta}]$	$\hat{\gamma}$
Intercept - Xpert TB test	6.5	1.1	0.6
New Spline Covariate	0.6	0.07	
Specificity	-0.03	0.01	
Sensitivity	-0.009	0.003	
TB Screening with Smear Microscopy	-0.7	0.2	
TB Screening with Interferon Gamma Release Assays	1.0	0.6	
Log prevalence per Capita	-1.1	0.2	
Log per year or full intervention cost	0.6	0.07	
	R^2	RMSE	Sample Size
Sample with Sensitivity Analyses			
Fixed and Random Effects	1.0	0.7	61
Fixed Effects Only	0.9	1.0	
Ratio of upper bound to lower bound of uncertainty interval for predicted ICERs			
Xpert diagnostic test			24
Table S7.28. Parameter estimates and fit statistics for selected meta-regression model for tuberculosis diagnostics			
$\hat{\gamma}$ is the between study heterogeneity in the meta-regression analysis.			

We did not estimate the probability that TB diagnostics would be cost-saving, because there was only one Xpert diagnostic test ratio that was cost saving, and this did not meet our criteria for running the logistic regression.

7.8 Tuberculosis Treatment

	Log ICER	Log GDP per capita (2019 USD)	Time Horizon Magnitude	Payer or Sector	Log DALYS per capita	Efficacy	Log per year or full intervention cost (2019 USD)	Log prevalence per capita
Log ICER	1							
Log GDP per capita (2019 USD)	0.2	1						
Time Horizon Magnitude	-0.2	-0.4	1					
Payer or Sector	0.4	-0.7	0.8	1				
Log DALYS per capita	0.8	-0.4	-0.2	0.06	1			
Efficacy	-0.8	-0.6	0.7	0.9	-0.06	1		
Log per year or full intervention cost (2019 USD)	0.6	0.05	-0.2	0.3	0.6	0.3	1	
Log prevalence per capita	-0.4	0.6	-0.2	-0.2	0.3	0.006	0.6	1

Table S7.29. Correlation matrix of ICERs and covariates for the tuberculosis treatment analyses sample (n=612) (n=10)

Version	Burden measure ***	Monotonicity imposed	Keywords in spline fit*	R2	RMSE	Predicted mean ICER	Ratio upper lower mean	Mean from data	Sample size
1091*	NA	Yes	NA	0.6	1.4	122	195	180	8

Table S7.30. Model fit statistics for model with sensitivity analyses and no crosswalks. (*) denotes selected model

* Denotes selected model
** Experiments with including keyword in spline are not applicable to cause-type group with one keyword
*** Sample size supported only one independent variable (efficacy), so the burden variable was not included

Given the small sample size we set the percent trimmed to zero and did not conduct experiments with this parameter.

Figure S7.19 Tuberculosis Treatment Signal and log GDP per capita

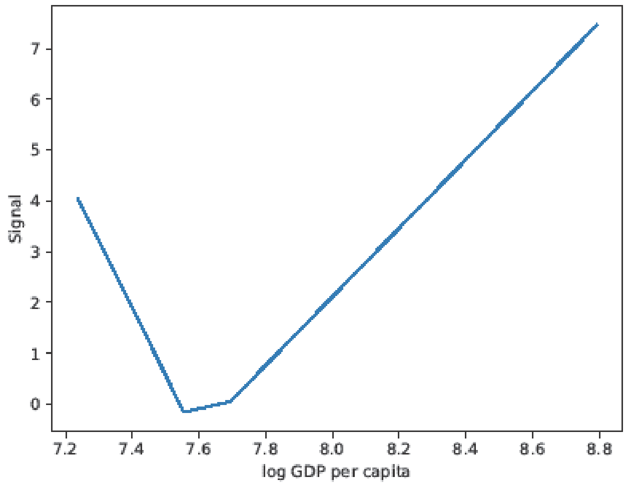

Figure S7.20 Tuberculosis treatment log predicted ICER and log GDP per capita

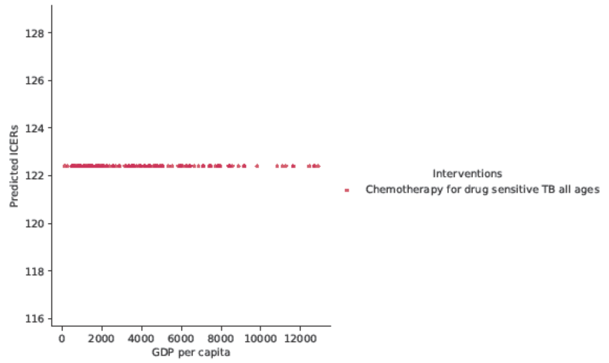

Figure S7.21 Tuberculosis treatment published and predicted log ICERs

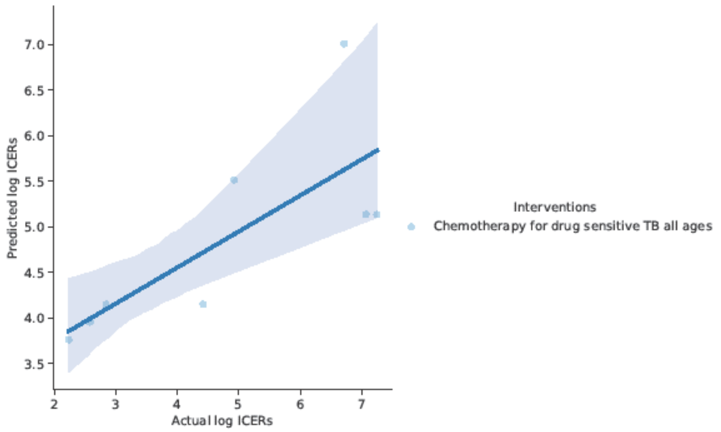

Covariate	$\hat{\beta}$	$\widehat{SE}[\hat{\beta}]$	$\hat{\gamma}$
Intercept	7.5	1.0	1.3
Efficacy	0.004	0.01	
	R^2	RMSE	Sample Size
Sample with Sensitivity Analyses			
Fixed and Random Effects	1.00	0.08	8
Fixed Effects Only	0.6	1.4	
Ratio of upper bound to lower bound of uncertainty interval for predicted ICERs			
Chemotherapy for TB			195
Table S7.31. Parameter estimates and fit statistics for selected meta-regression model for tuberculosis treatment			

We did not estimate the probability that TB treatment would be cost-saving, because the two cost saving ratios were from same article. While there are three ratios total from that article, the model does not converge. The model suffers from issues of singularity, which cannot be managed given the small dataset and inclusion of only one covariate.

7.9 List of 25 interventions included the meta-regression analysis sample and 14 with predictions

Count	intervention_keywords_final	Covariate selected	ICER predicted
	ART		
1	antiretroviral therapy for hiv	yes	no
2	antiretroviral therapy for hiv for prevention	yes	yes, 2 age groups
3	antiretroviral therapy for hiv, hiv testing	yes	no
4	antiretroviral therapy for hiv for prevention, hiv testing	yes	no
5	pooled hiv testing, antiretroviral therapy for hiv for prevention	yes	no
6	prevention of mother to child hiv transmission, antiretroviral therapy for hiv	yes	yes
7	antiretroviral therapy for hiv, methadone maintenance therapy	yes	no
	PREP		yes
8	Heterosexuals	yes	
9	MSM	yes	yes
	Syphilis diagnostics		
10	antibiotics for syphilis, syphilis testing	yes	yes
11	antibiotics for syphilis, syphilis testing, hiv testing	no	n/a
	Malaria prevention		
12	bed nets	yes	yes
13	malaria vaccines	yes	yes
14	malaria intermittent preventive treatment for pregnant women	yes	yes
15	malaria intermittent preventive treatment	yes	yes
16	indoor residual spraying	no	n/a
17	indoor residual spraying, malaria treatment, bed nets	no	n/a
	TB prevention		
18	tuberculosis vaccines	yes	yes
19	prophylaxis for people without active tb	yes	yes
20	prophylaxis for people without active tb, tuberculosis screening with tuberculin skin test	yes	no
	TB diagnostics		
21	tuberculosis screening with interferon gamma release assays	yes	no
22	tuberculosis screening with smear microscopy	yes	no
23	tuberculosis testing	no	n/a
24	xpert rapid tuberculosis test	yes	yes
	TB treatment		
25	antibiotics for tuberculosis	yes	yes

Table S7.32 List of 25 interventions included in the meta-regression analysis sample and 14 with predictions

Section 8. League tables

In this section, we report country-specific league tables with 14 interventions for 128 countries.

Legend: Causes are grouped by color where red is HIV/AIDS, yellow is malaria, green is syphilis, and blue is TB.

The league tables also show the potential role of thresholds in selecting interventions to support. The green vertical line is the country-specific threshold and the dashed line is GDP per capita.

Interventions in bold font are eligible for Global Fund support and to purchase commodities as subsidized prices when relevant. The ICERs may be lower-bound estimates when a country is not eligible for support for that cause. The final samples for the meta-regression analyses were 258 ratios from 57 articles for HIV/AIDS-ART, 74 ratios from 9 articles for HIV/AIDS-PREP, 74 ratios from 10 articles for malaria prevention, 111 ratios from 4 articles for syphilis diagnostics, 8 ratios from 5 articles for DS-TB treatment, 20 ratios from 8 articles for TB prevention, and 61 ratios from 13 articles for TB diagnostics. The samples sizes for the logistic regression analyses were 240 ratios for HIV/AIDS – ART, and 29 for malaria prevention.

Abbreviations: adults=ages 10 or more years, ART=Antiretroviral therapy, BCG=Bacille Calmette−Guerin, DS=drug susceptible, ICER=incremental cost-effectiveness ratio, IRS=Indoor residual spraying, IPT=Intermittent preventive treatment for malaria, m=months, MSM=Men who have sex with men, Option B+=HIV/AIDS screening for pregnant women and lifelong ART, PREP=Pre−exposure prophylaxis, TB=Tuberculosis, y=years

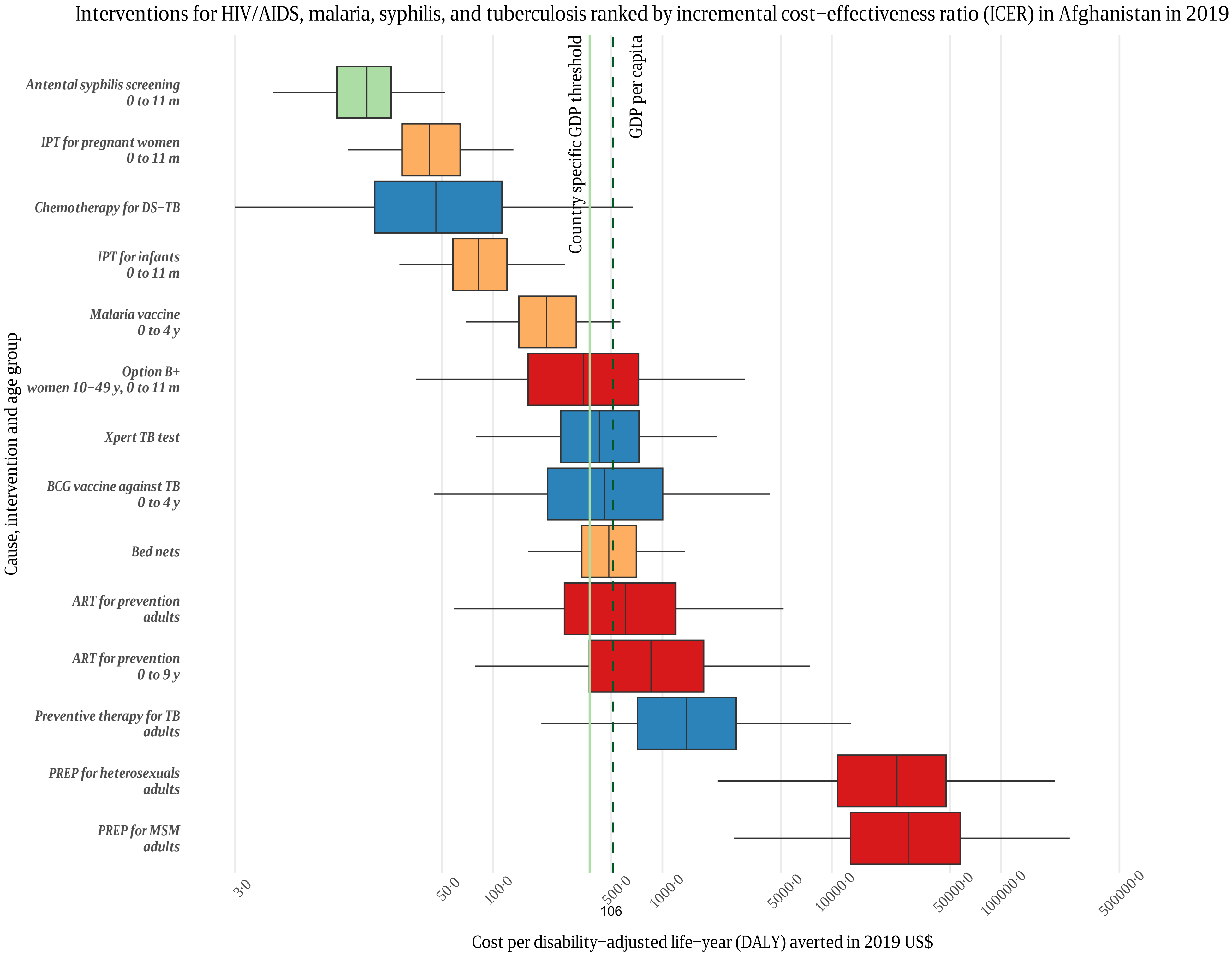

Interventions for HIV/AIDS, malaria, syphilis, and tuberculosis ranked by incremental cost−effectiveness ratio (ICER) in Afghanistan in 2019
Country specific GDP threshold
GDP per capita
Antental syphilis screening 0 to 11 m
IPT for pregnant women 0 to 11 m
Chemotherapy for DS−TB
IPT for infants 0 to 11 m
Malaria vaccine 0 to 4 y
Option B+ women 10−49 y, 0 to 11 m
Xpert TB test
BCG vaccine against TB 0 to 4 y
Bed nets
ART for prevention adults
ART for prevention 0 to 9 y
Preventive therapy for TB adults
PREP for heterosexuals adults
PREP for MSM adults
Cause, intervention and age group
3·0
50·0
100·0
500·0
106
1000·0
5000·0
10000·0
50000·0
100000·0
500000·0
Cost per disability−adjusted life−year (DALY) averted in 2019 US$

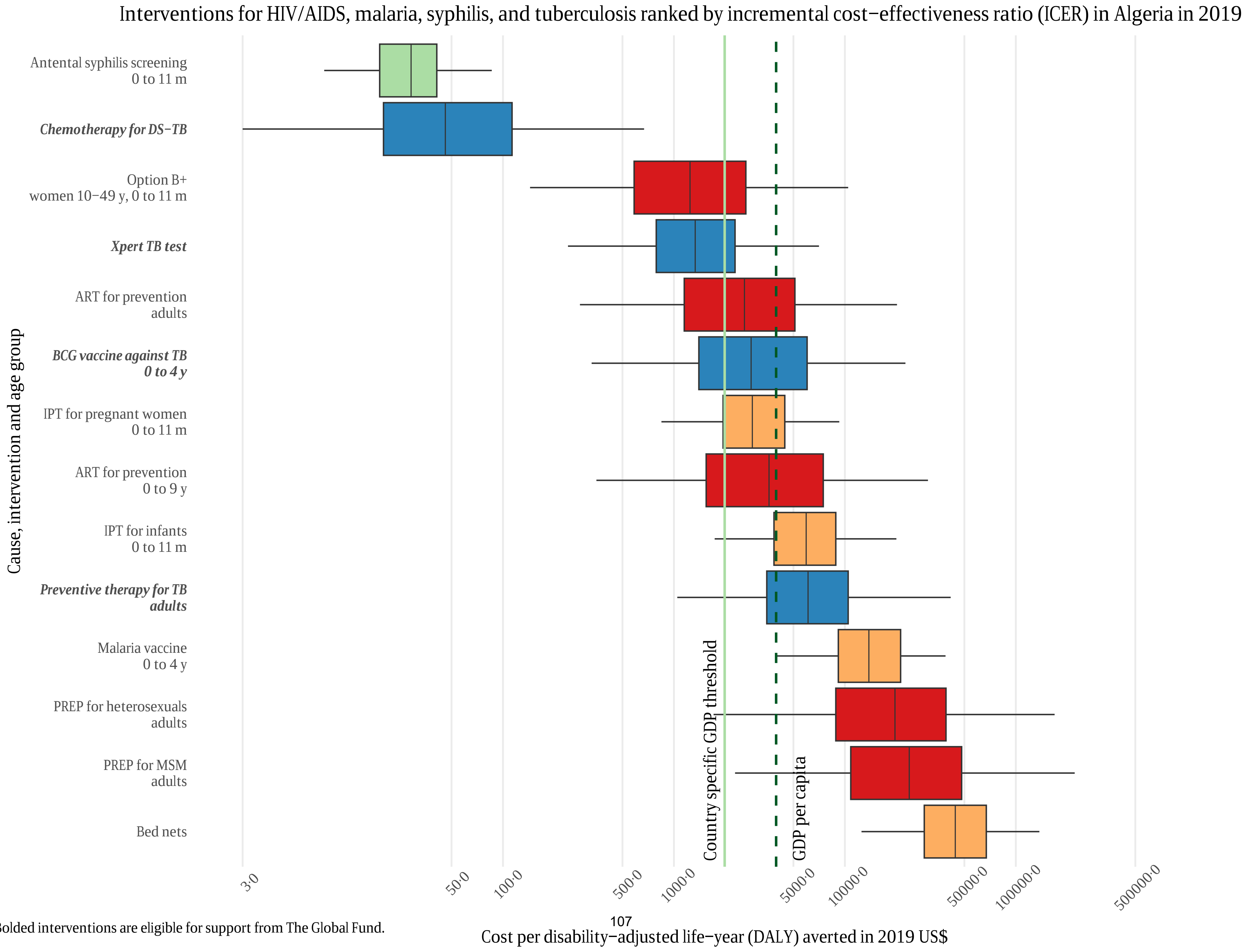

Bolded interventions are eligible for support from The Global Fund.

Interventions for HIV/AIDS, malaria, syphilis, and tuberculosis ranked by incremental cost−effectiveness ratio (ICER) in Angola in 2019

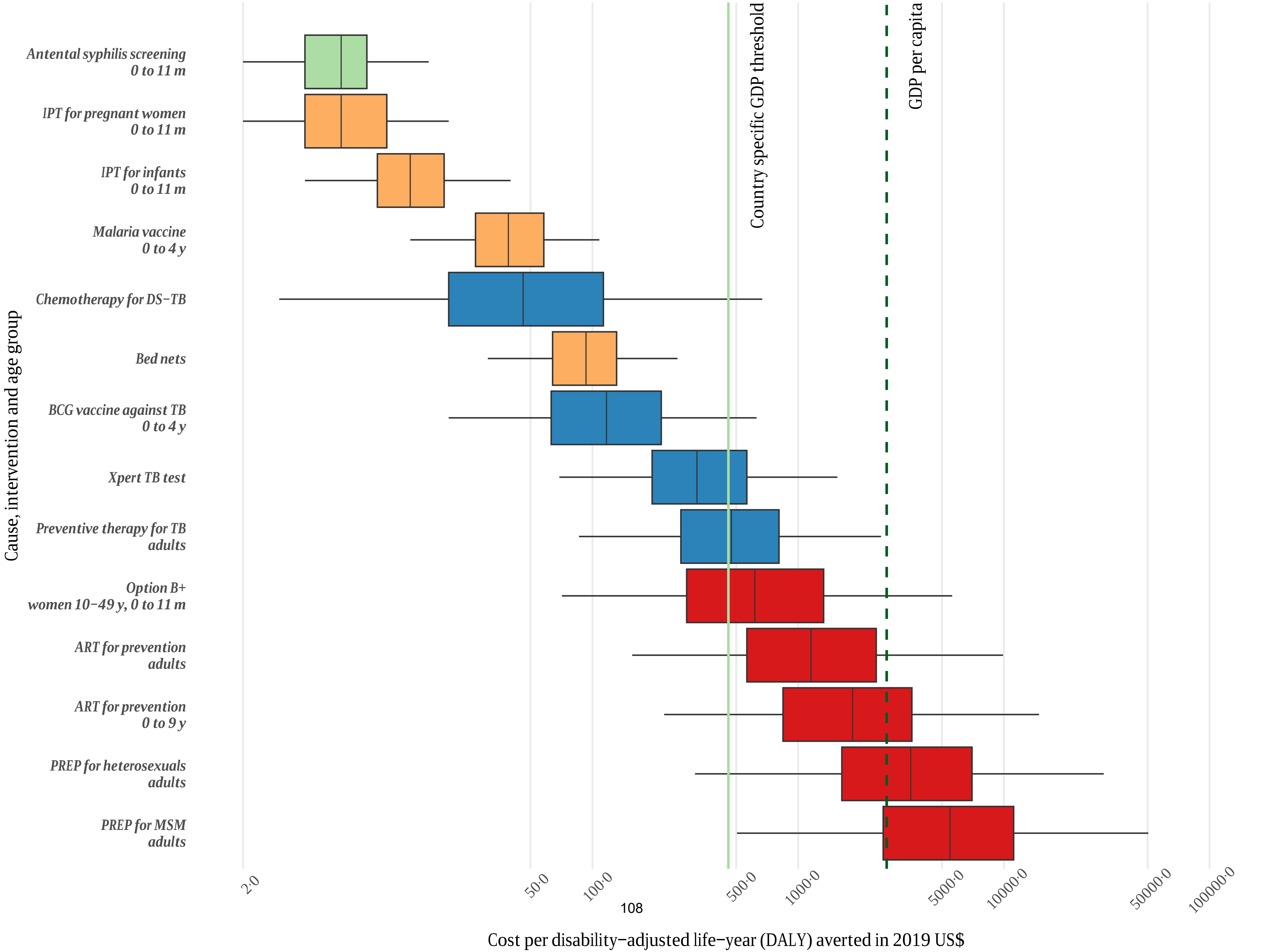

Interventions for HIV/AIDS, malaria, syphilis, and tuberculosis ranked by incremental cost−effectiveness ratio (ICER) in Armenia in 2019

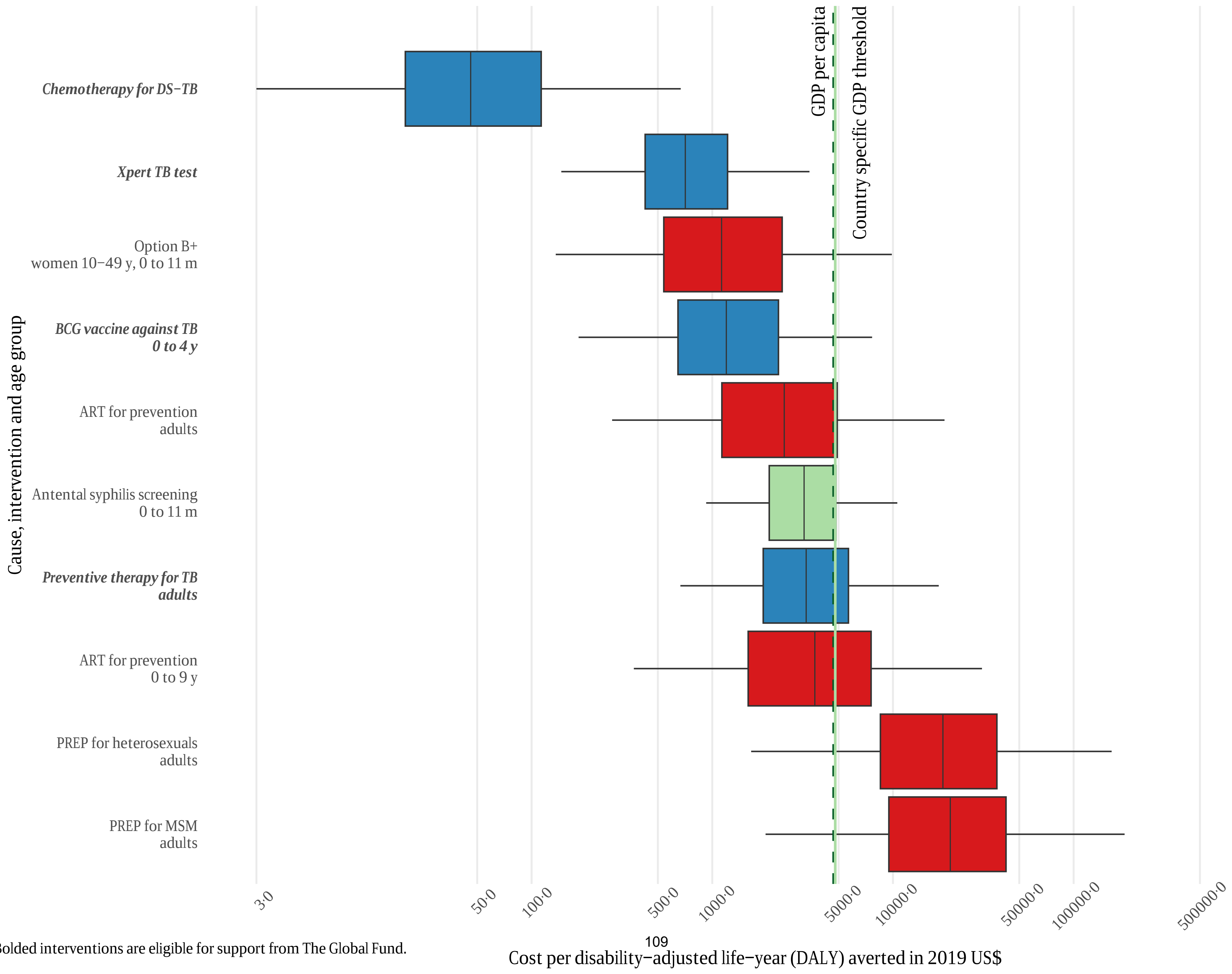

Bolded interventions are eligible for support from The Global Fund.

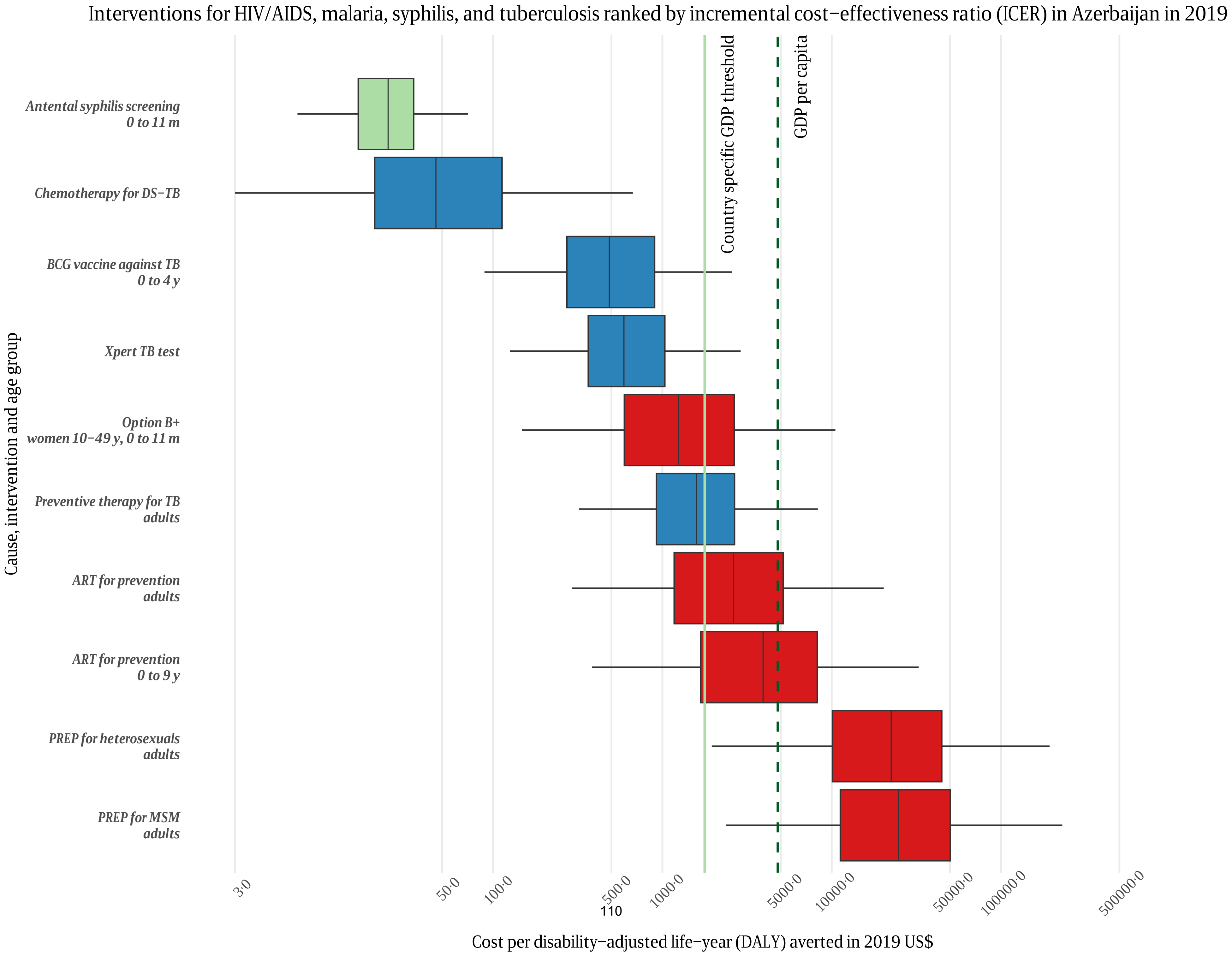
Interventions for HIV/AIDS, malaria, syphilis, and tuberculosis ranked by incremental cost−effectiveness ratio (ICER) in Azerbaijan in 2019
Country specific GDP threshold
GDP per capita
Antental syphilis screening
0 to 11 m
Chemotherapy for DS−TB
BCG vaccine against TB
0 to 4 y
Xpert TB test
Option B+
women 10−49 y, 0 to 11 m
Preventive therapy for TB
adults
ART for prevention
adults
ART for prevention
0 to 9 y
PREP for heterosexuals
adults
PREP for MSM
adults
Cause, intervention and age group
3·0
50·0
100·0
500·0
1000·0
5000·0
10000·0
50000·0
100000·0
500000·0
Cost per disability−adjusted life−year (DALY) averted in 2019 US$

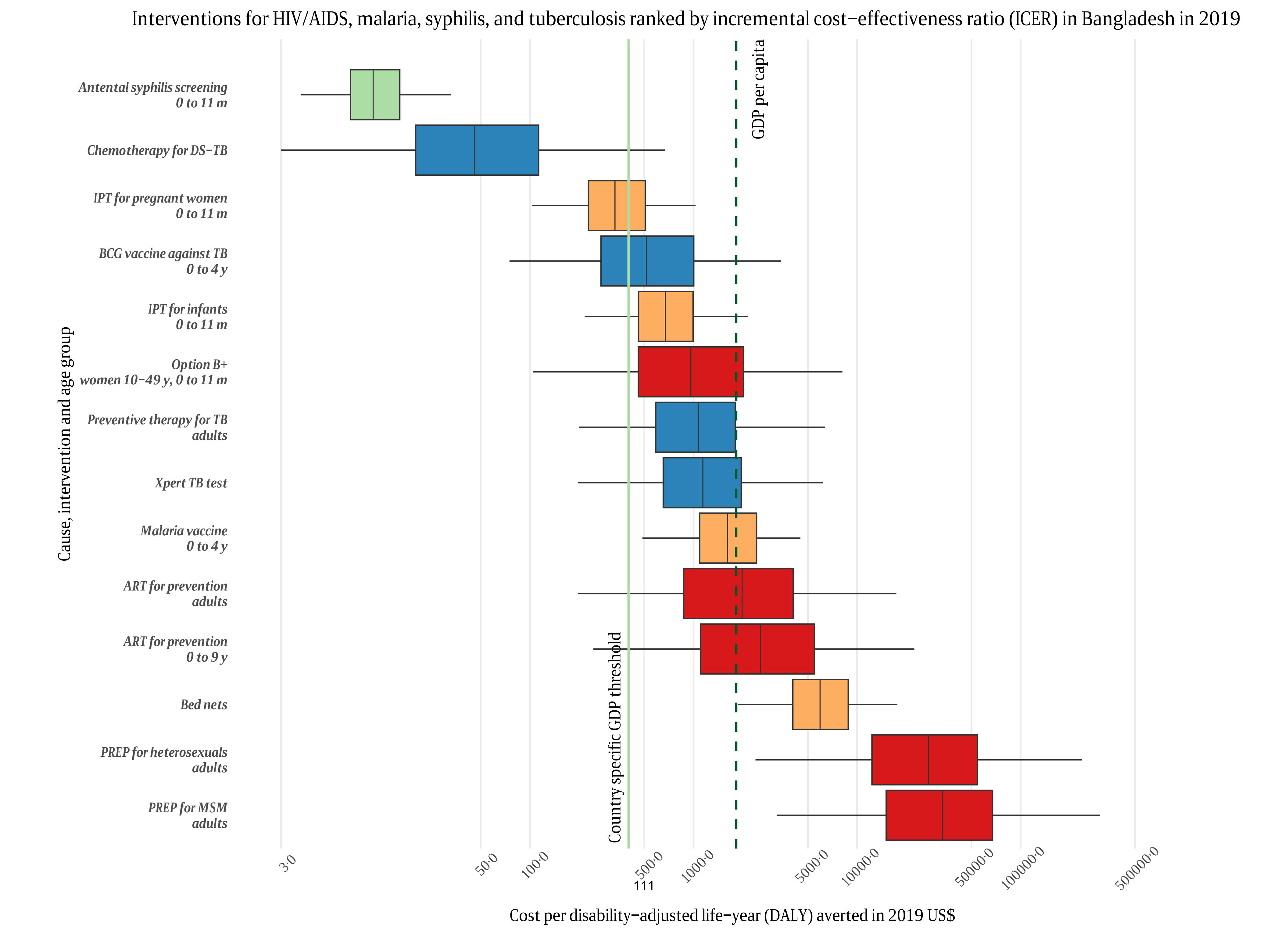

Interventions for HIV/AIDS, malaria, syphilis, and tuberculosis ranked by incremental cost−effectiveness ratio (ICER) in Bangladesh in 2019
Cause, intervention and age group
Antental syphilis screening 0 to 11 m
Chemotherapy for DS−TB
IPT for pregnant women 0 to 11 m
BCG vaccine against TB 0 to 4 y
IPT for infants 0 to 11 m
Option B+ women 10−49 y, 0 to 11 m
Preventive therapy for TB adults
Xpert TB test
Malaria vaccine 0 to 4 y
ART for prevention adults
ART for prevention 0 to 9 y
Bed nets
PREP for heterosexuals adults
PREP for MSM adults
GDP per capita
Country specific GDP threshold
3·0
50·0
100·0
500·0
1000·0
5000·0
10000·0
50000·0
100000·0
500000·0
111
Cost per disability−adjusted life−year (DALY) averted in 2019 US$

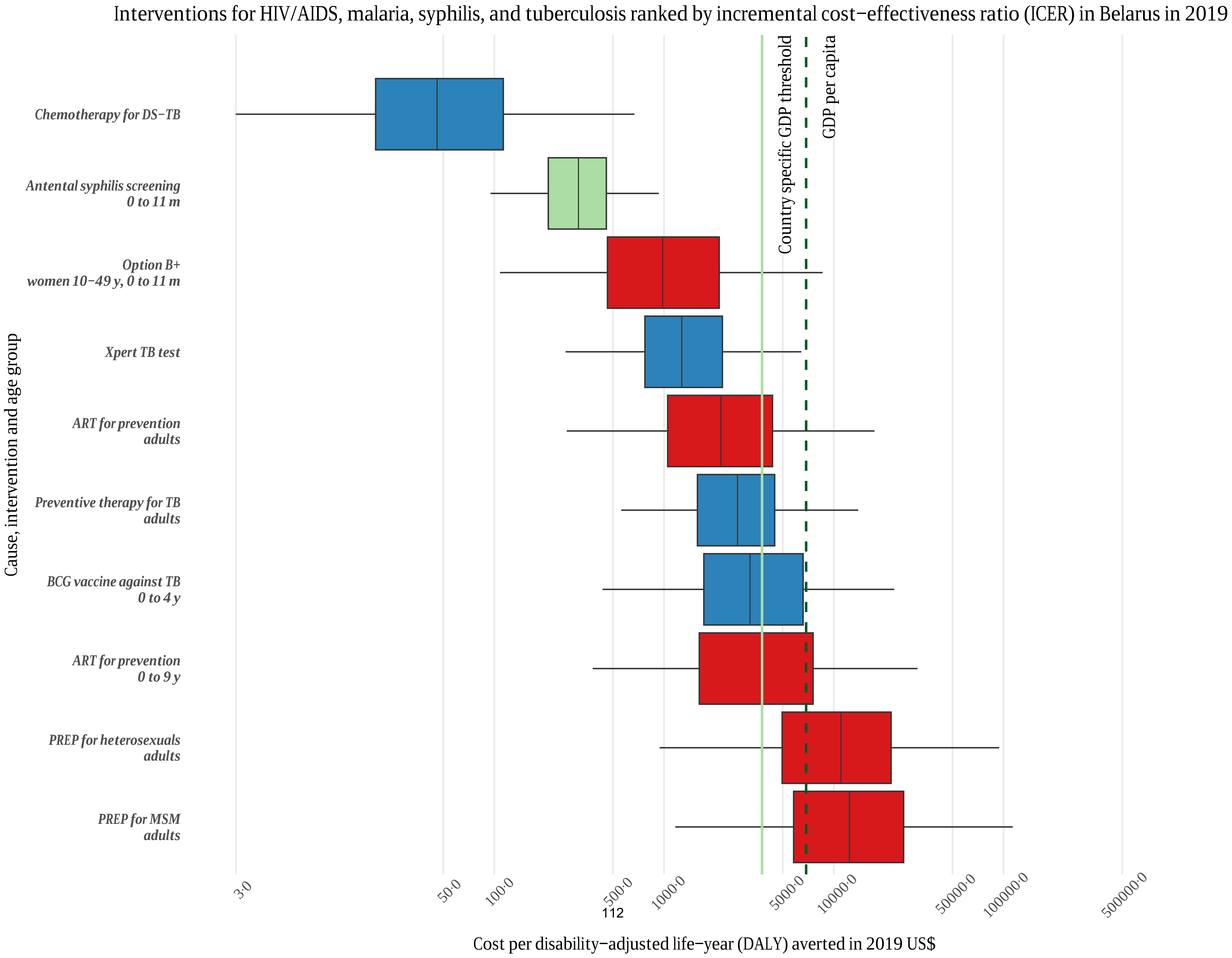

Interventions for HIV/AIDS, malaria, syphilis, and tuberculosis ranked by incremental cost−effectiveness ratio (ICER) in Belarus in 2019
Country specific GDP threshold
GDP per capita
Chemotherapy for DS−TB
Antental syphilis screening 0 to 11 m
Option B+ women 10−49 y, 0 to 11 m
Xpert TB test
ART for prevention adults
Preventive therapy for TB adults
BCG vaccine against TB 0 to 4 y
ART for prevention 0 to 9 y
PREP for heterosexuals adults
PREP for MSM adults
Cause, intervention and age group
3·0
50·0
100·0
500·0
1000·0
5000·0
10000·0
50000·0
100000·0
500000·0
Cost per disability−adjusted life−year (DALY) averted in 2019 US$

Interventions for HIV/AIDS, malaria, syphilis, and tuberculosis ranked by incremental cost−effectiveness ratio (ICER) in Belize in 2019

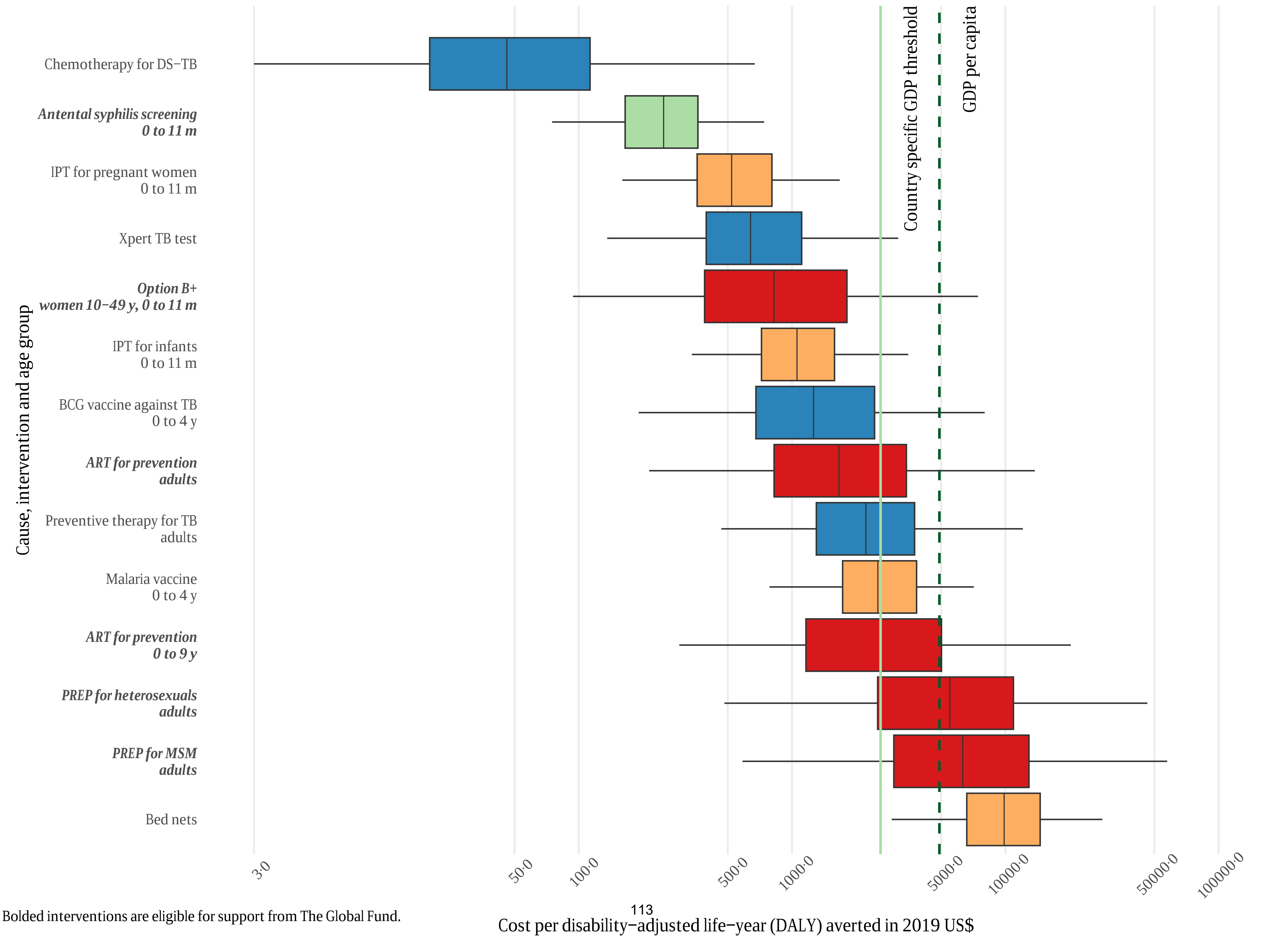

Bolded interventions are eligible for support from The Global Fund.

Interventions for HIV/AIDS, malaria, syphilis, and tuberculosis ranked by incremental cost−effectiveness ratio (ICER) in Benin in 2019

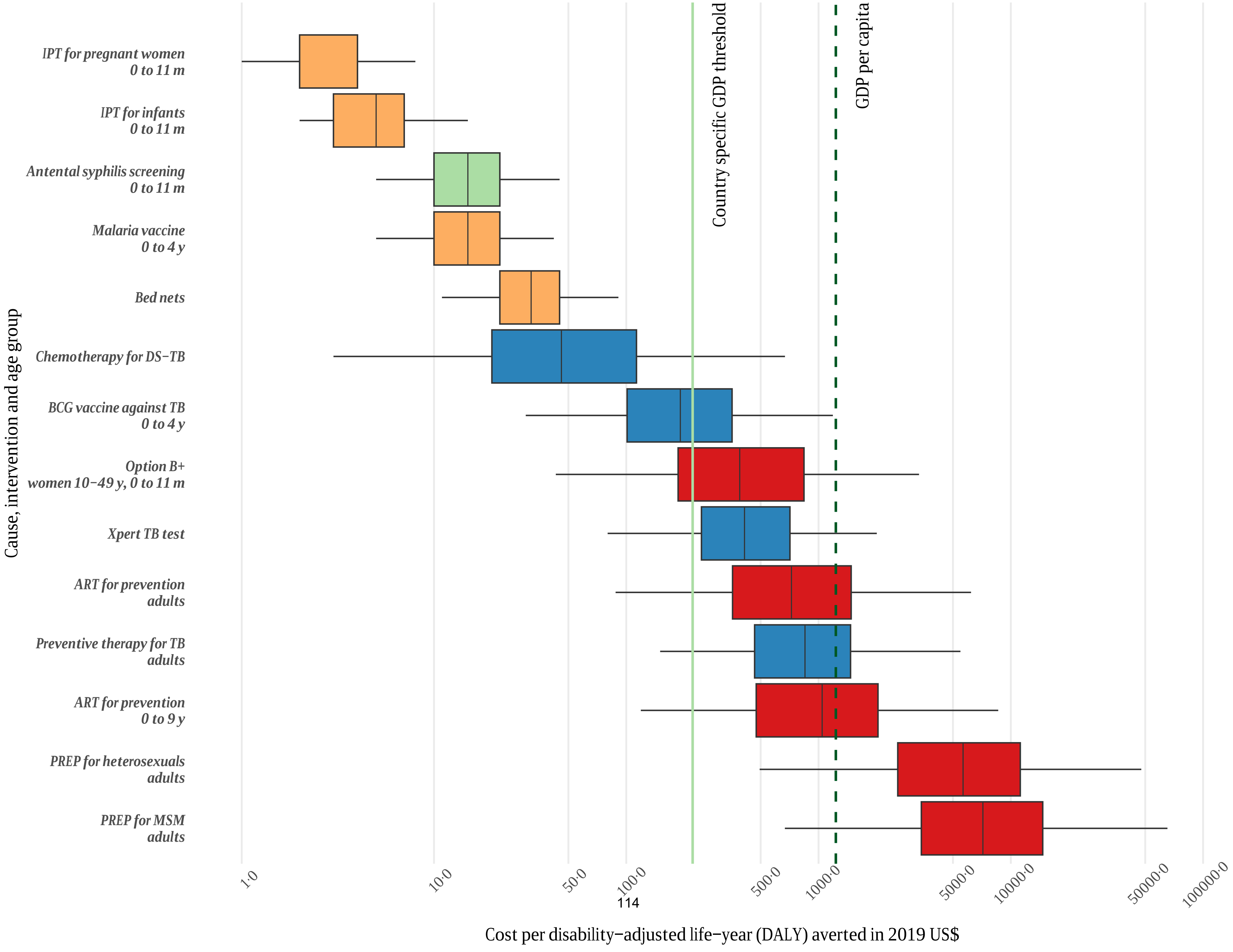

Interventions for HIV/AIDS, malaria, syphilis, and tuberculosis ranked by incremental cost−effectiveness ratio (ICER) in Bhutan in 2019

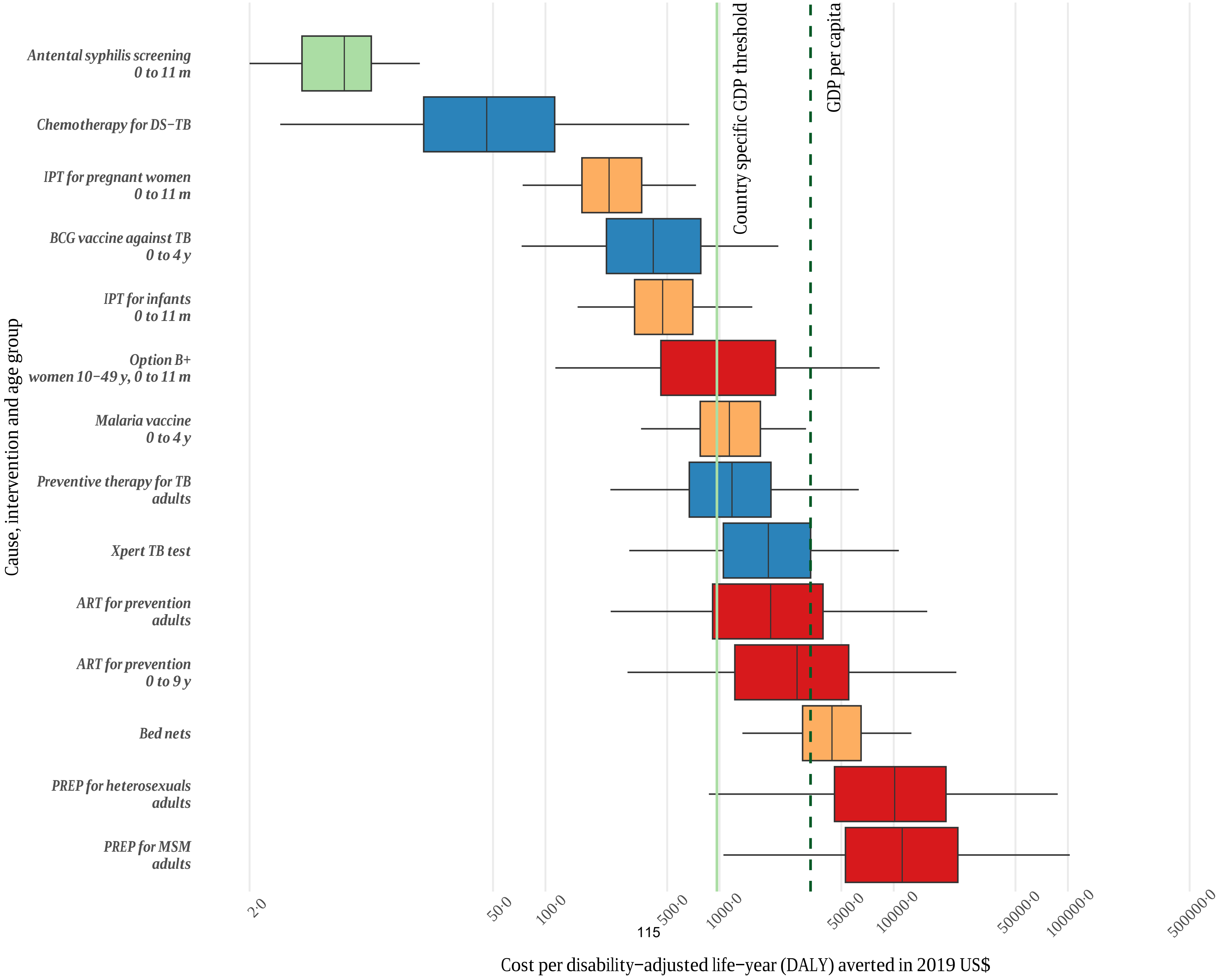

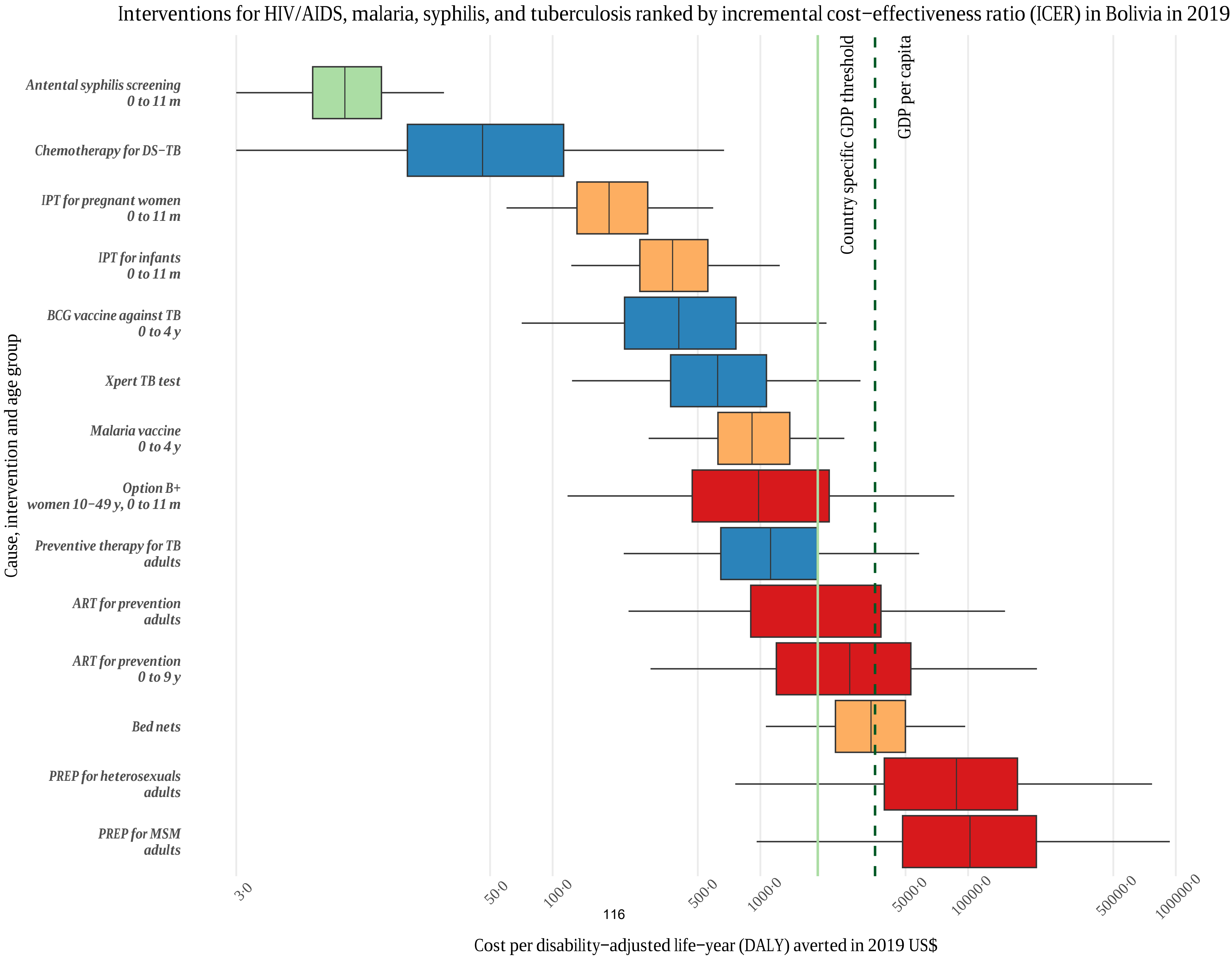

Interventions for HIV/AIDS, malaria, syphilis, and tuberculosis ranked by incremental cost−effectiveness ratio (ICER) in Bolivia in 2019
Cause, intervention and age group
Antental syphilis screening
0 to 11 m
Chemotherapy for DS−TB
IPT for pregnant women
0 to 11 m
IPT for infants
0 to 11 m
BCG vaccine against TB
0 to 4 y
Xpert TB test
Malaria vaccine
0 to 4 y
Option B+
women 10−49 y, 0 to 11 m
Preventive therapy for TB
adults
ART for prevention
adults
ART for prevention
0 to 9 y
Bed nets
PREP for heterosexuals
adults
PREP for MSM
adults
Country specific GDP threshold
GDP per capita
3·0
50·0
100·0
500·0
1000·0
5000·0
10000·0
50000·0
100000·0
Cost per disability−adjusted life−year (DALY) averted in 2019 US$

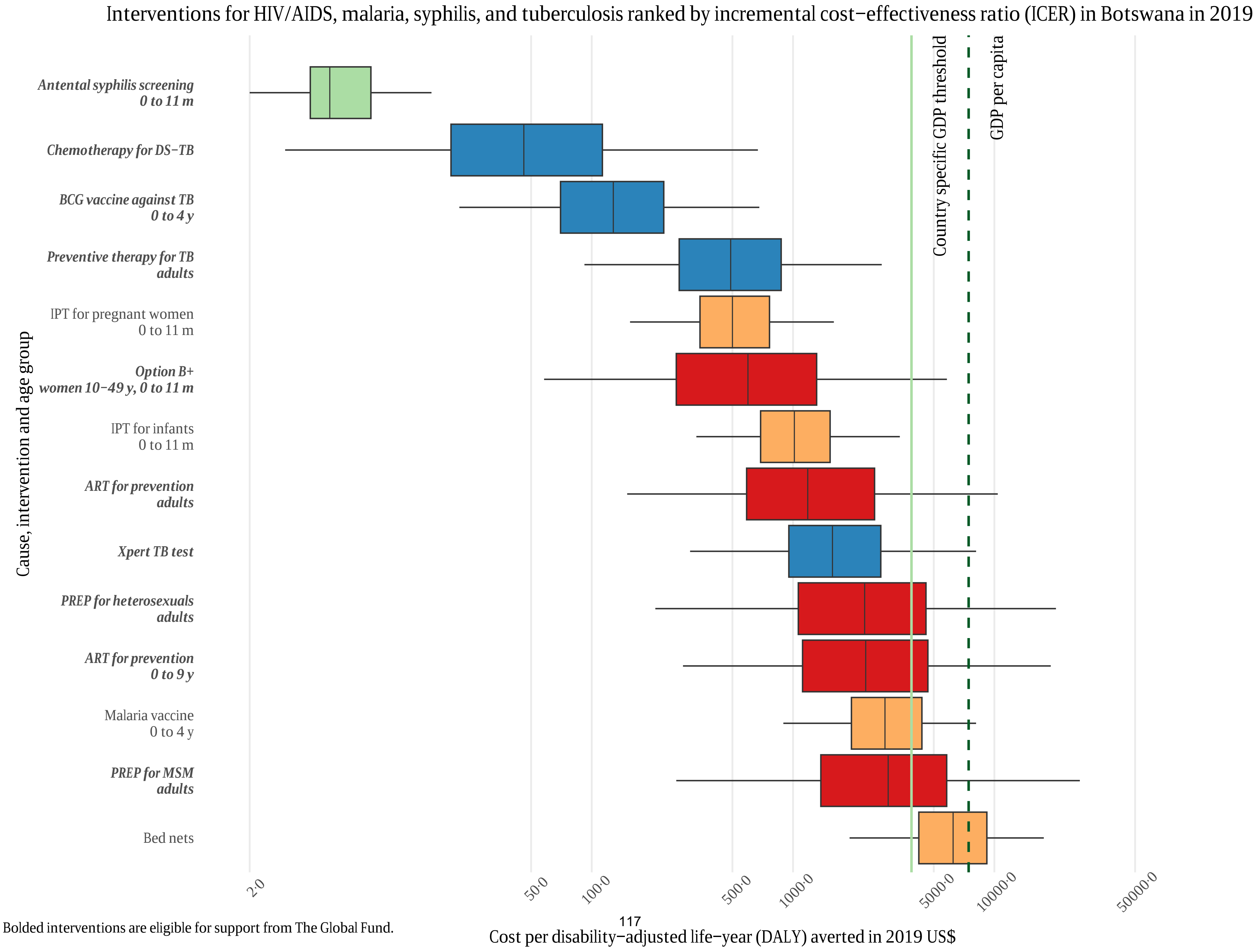

Interventions for HIV/AIDS, malaria, syphilis, and tuberculosis ranked by incremental cost−effectiveness ratio (ICER) in Botswana in 2019
Antental syphilis screening
0 to 11 m
Chemotherapy for DS−TB
BCG vaccine against TB
0 to 4 y
Preventive therapy for TB
adults
IPT for pregnant women
0 to 11 m
Option B+
women 10−49 y, 0 to 11 m
IPT for infants
0 to 11 m
ART for prevention
adults
Xpert TB test
PREP for heterosexuals
adults
ART for prevention
0 to 9 y
Malaria vaccine
0 to 4 y
PREP for MSM
adults
Bed nets
Cause, intervention and age group
Country specific GDP threshold
GDP per capita
2·0
50·0
100·0
500·0
1000·0
5000·0
10000·0
50000·0
Cost per disability−adjusted life−year (DALY) averted in 2019 US$
Bolded interventions are eligible for support from The Global Fund.

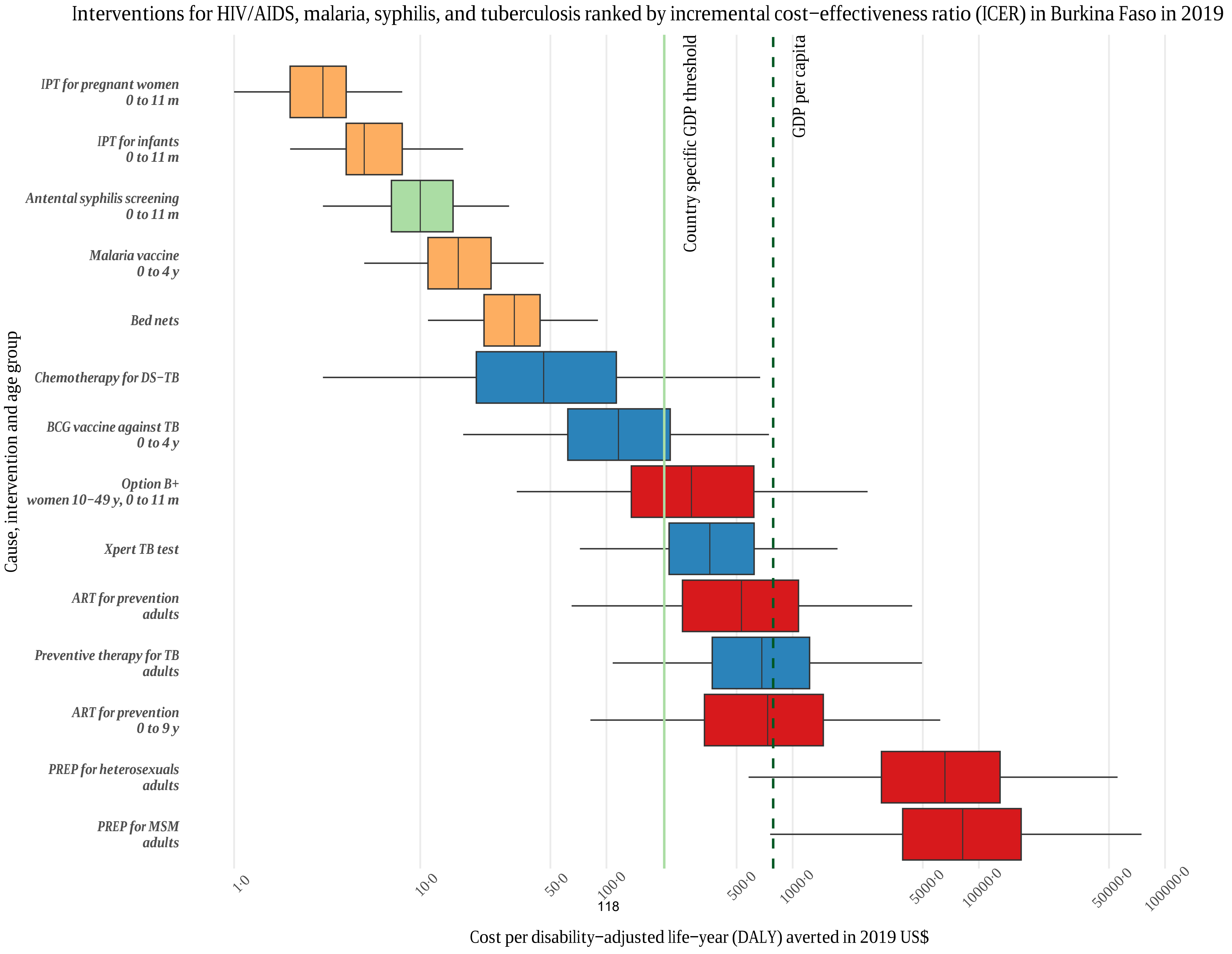

Interventions for HIV/AIDS, malaria, syphilis, and tuberculosis ranked by incremental cost−effectiveness ratio (ICER) in Burkina Faso in 2019
Country specific GDP threshold
GDP per capita
IPT for pregnant women 0 to 11 m
IPT for infants 0 to 11 m
Antental syphilis screening 0 to 11 m
Malaria vaccine 0 to 4 y
Bed nets
Chemotherapy for DS−TB
BCG vaccine against TB 0 to 4 y
Option B+ women 10−49 y, 0 to 11 m
Xpert TB test
ART for prevention adults
Preventive therapy for TB adults
ART for prevention 0 to 9 y
PREP for heterosexuals adults
PREP for MSM adults
Cause, intervention and age group
1·0
10·0
50·0
100·0
118
500·0
1000·0
5000·0
10000·0
50000·0
100000·0
Cost per disability−adjusted life−year (DALY) averted in 2019 US$

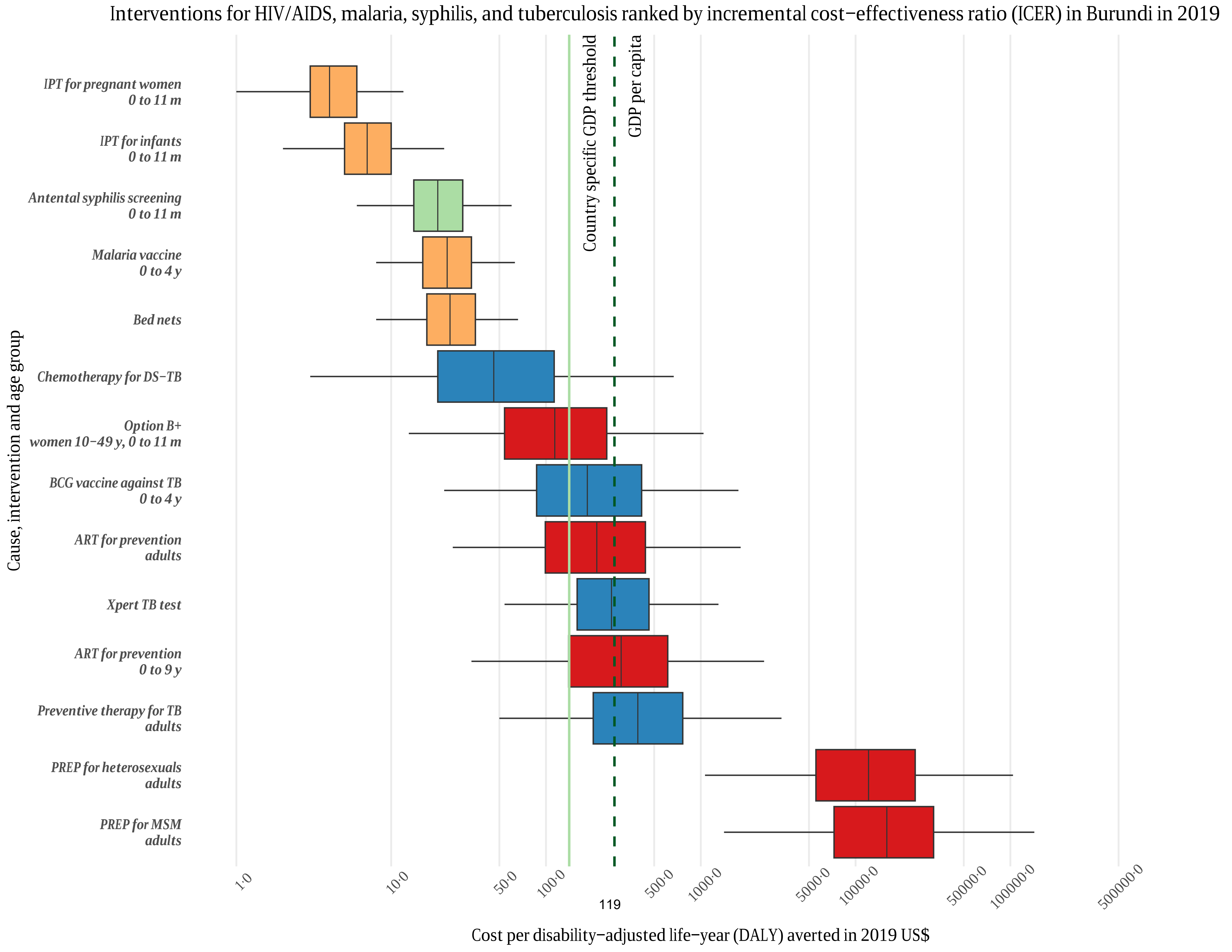
Interventions for HIV/AIDS, malaria, syphilis, and tuberculosis ranked by incremental cost−effectiveness ratio (ICER) in Burundi in 2019
Country specific GDP threshold
GDP per capita
IPT for pregnant women
0 to 11 m
IPT for infants
0 to 11 m
Antental syphilis screening
0 to 11 m
Malaria vaccine
0 to 4 y
Bed nets
Chemotherapy for DS−TB
Option B+
women 10−49 y, 0 to 11 m
BCG vaccine against TB
0 to 4 y
ART for prevention
adults
Xpert TB test
ART for prevention
0 to 9 y
Preventive therapy for TB
adults
PREP for heterosexuals
adults
PREP for MSM
adults
Cause, intervention and age group
1·0
10·0
50·0
100·0
500·0
1000·0
5000·0
10000·0
50000·0
100000·0
500000·0
119
Cost per disability−adjusted life−year (DALY) averted in 2019 US$

Interventions for HIV/AIDS, malaria, syphilis, and tuberculosis ranked by incremental cost−effectiveness ratio (ICER) in Cape Verde in 2019

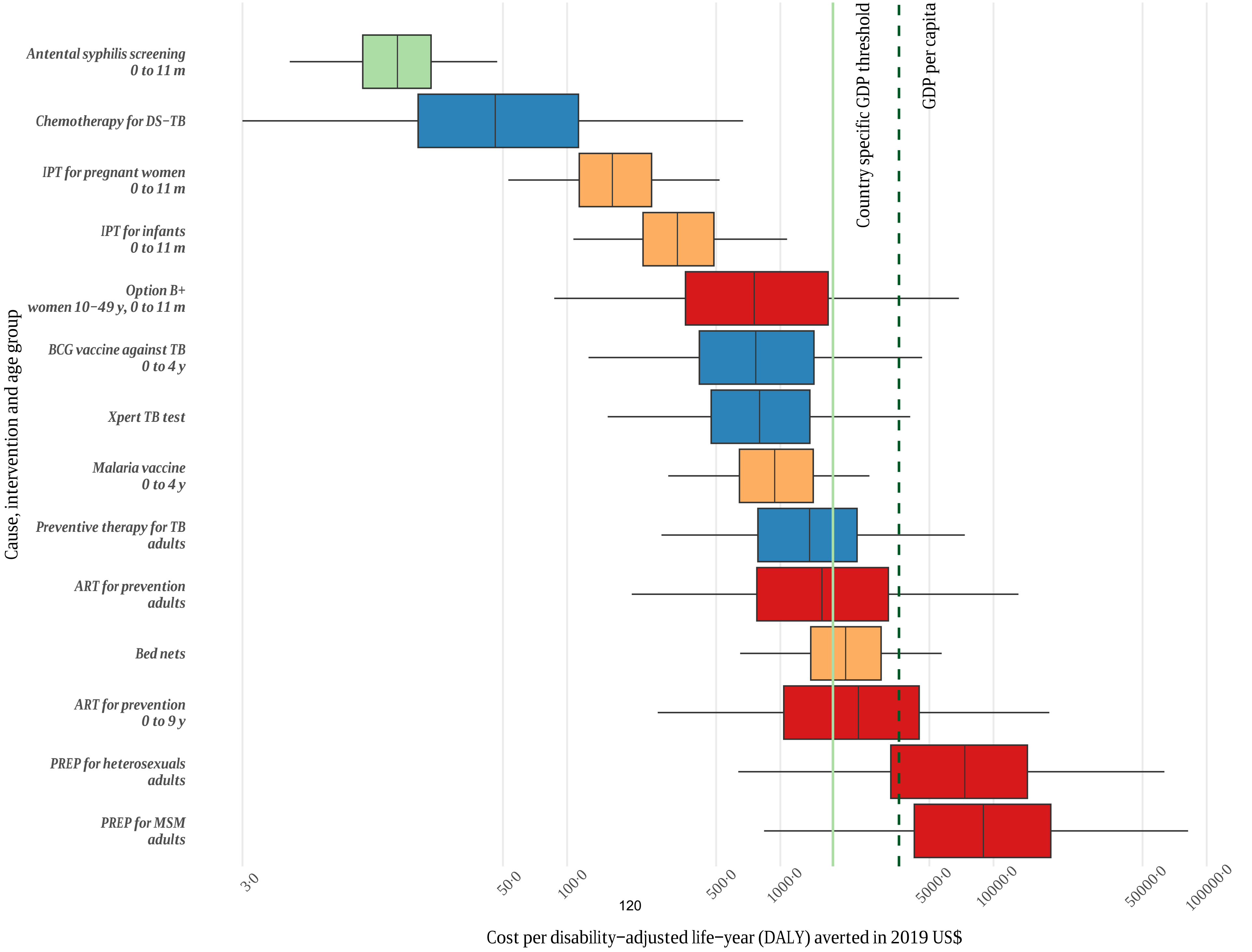

Interventions for HIV/AIDS, malaria, syphilis, and tuberculosis ranked by incremental cost−effectiveness ratio (ICER) in Cambodia in 2019

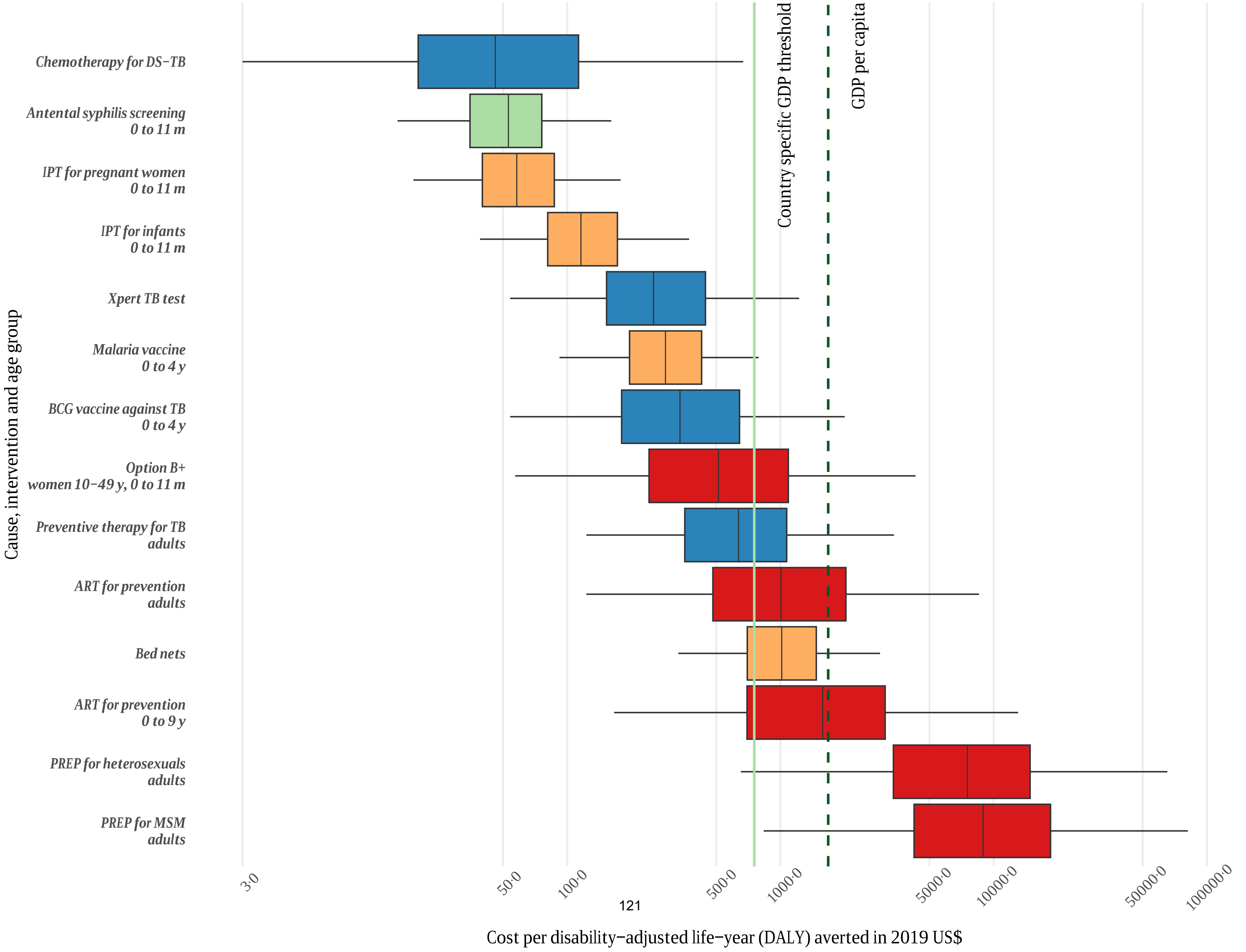

Interventions for HIV/AIDS, malaria, syphilis, and tuberculosis ranked by incremental cost−effectiveness ratio (ICER) in Cameroon in 2019

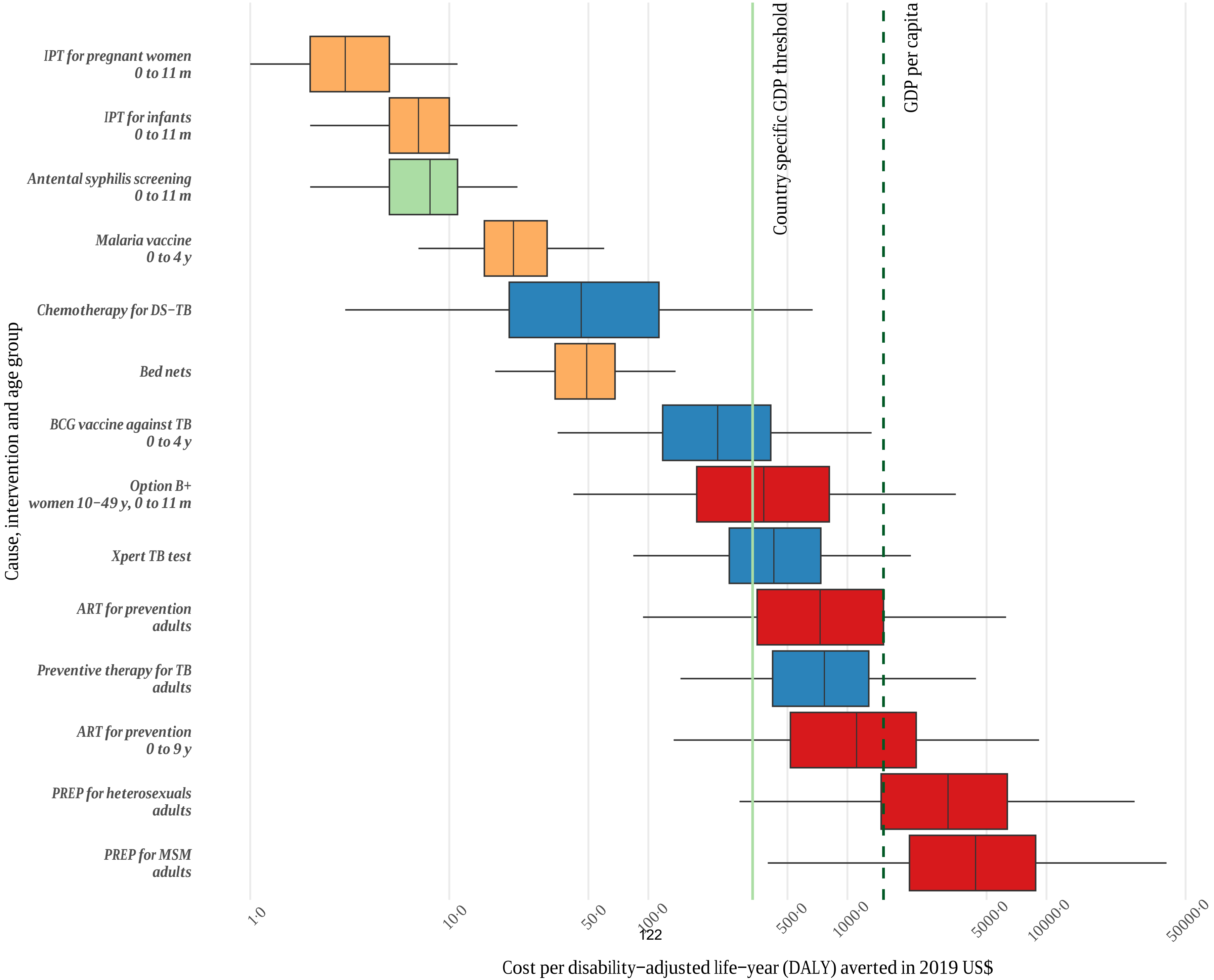

Interventions for HIV/AIDS, malaria, syphilis, and tuberculosis ranked by incremental cost−effectiveness ratio (ICER) in Central African Republic in 2019

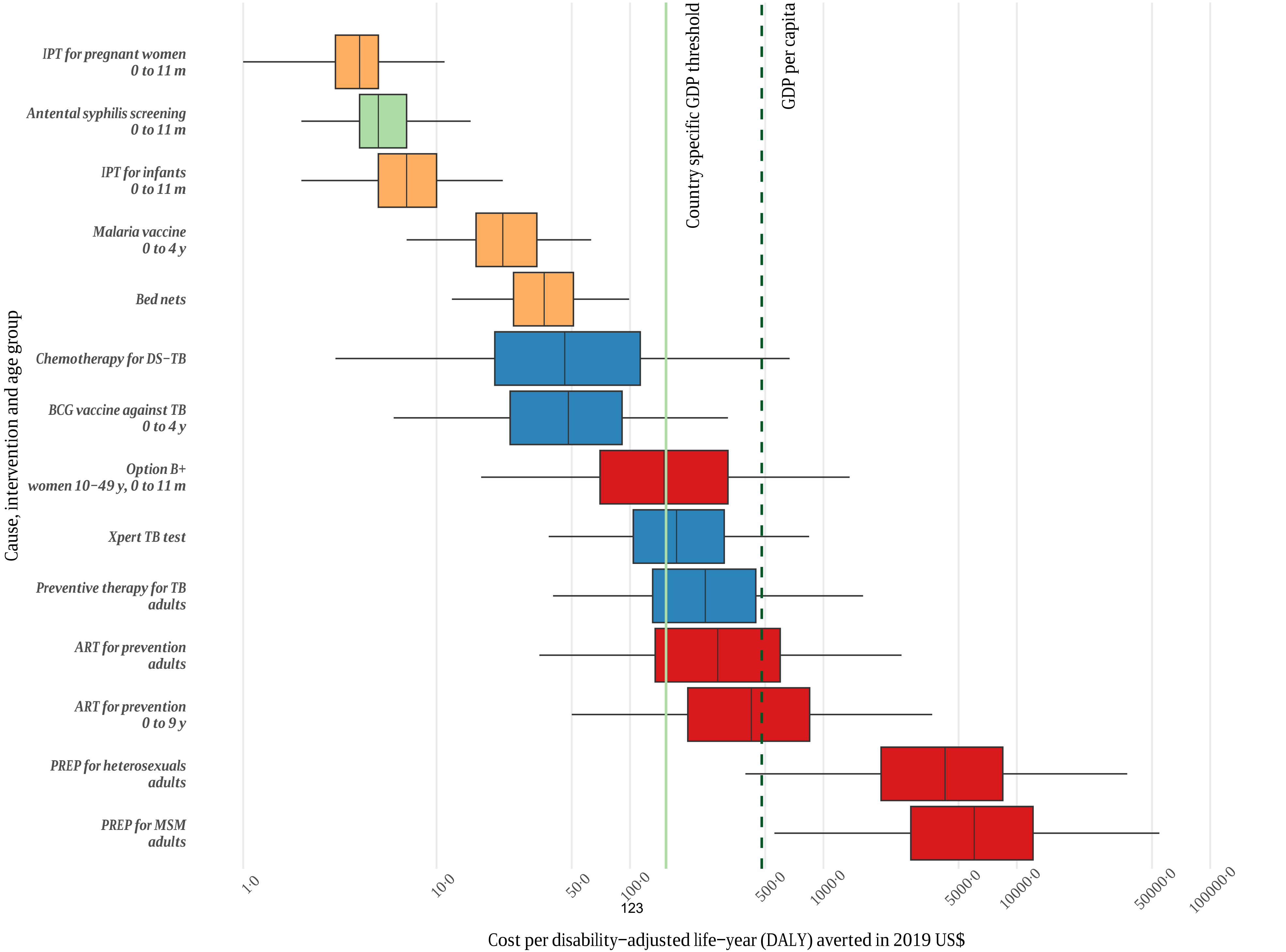

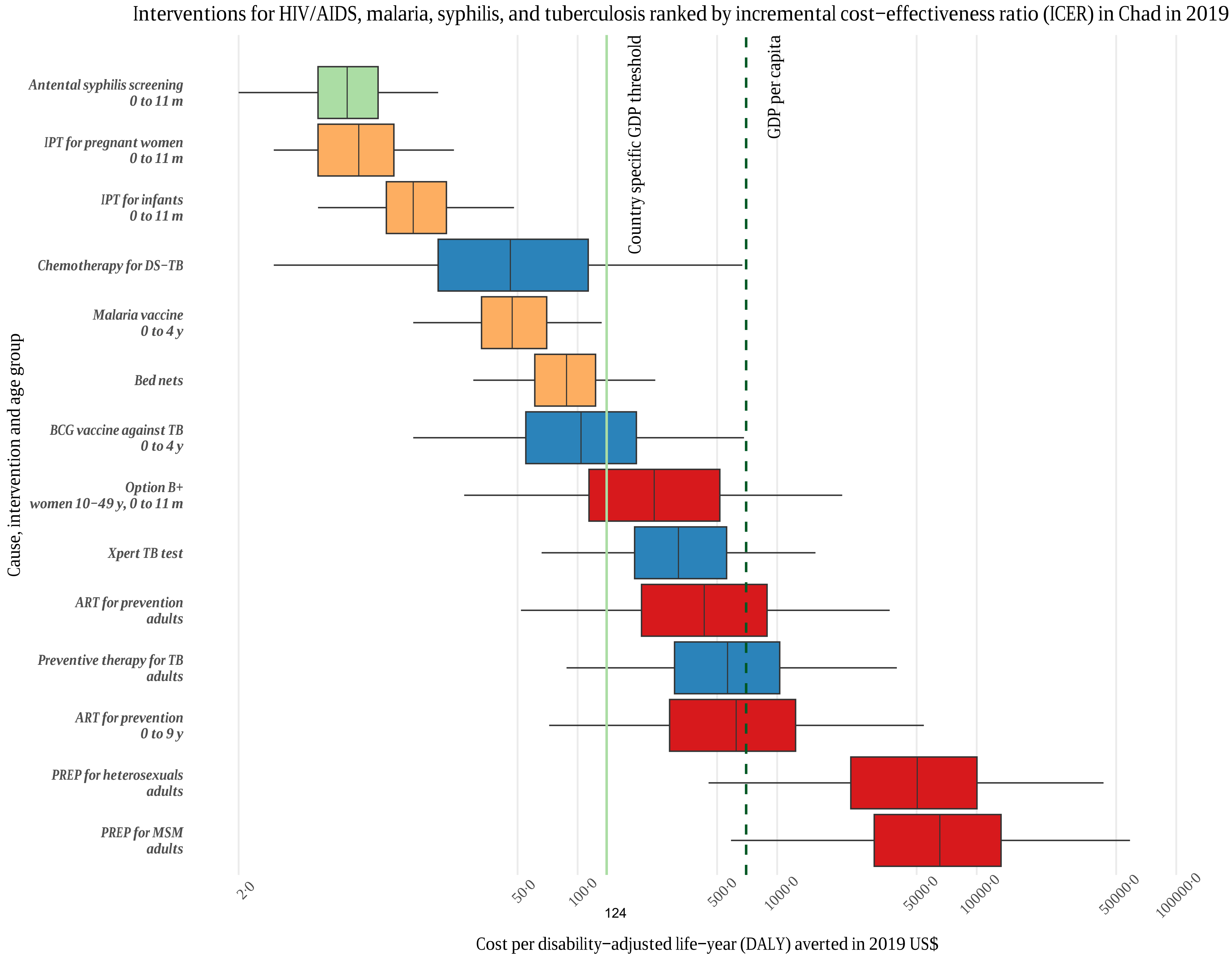

Interventions for HIV/AIDS, malaria, syphilis, and tuberculosis ranked by incremental cost−effectiveness ratio (ICER) in Chad in 2019
Country specific GDP threshold
GDP per capita
Antental syphilis screening
0 to 11 m
IPT for pregnant women
0 to 11 m
IPT for infants
0 to 11 m
Chemotherapy for DS−TB
Malaria vaccine
0 to 4 y
Bed nets
BCG vaccine against TB
0 to 4 y
Option B+
women 10−49 y, 0 to 11 m
Xpert TB test
ART for prevention
adults
Preventive therapy for TB
adults
ART for prevention
0 to 9 y
PREP for heterosexuals
adults
PREP for MSM
adults
Cause, intervention and age group
2·0
50·0
100·0
124
500·0
1000·0
5000·0
10000·0
50000·0
100000·0
Cost per disability−adjusted life−year (DALY) averted in 2019 US$

Interventions for HIV/AIDS, malaria, syphilis, and tuberculosis ranked by incremental cost−effectiveness ratio (ICER) in Colombia in 2019

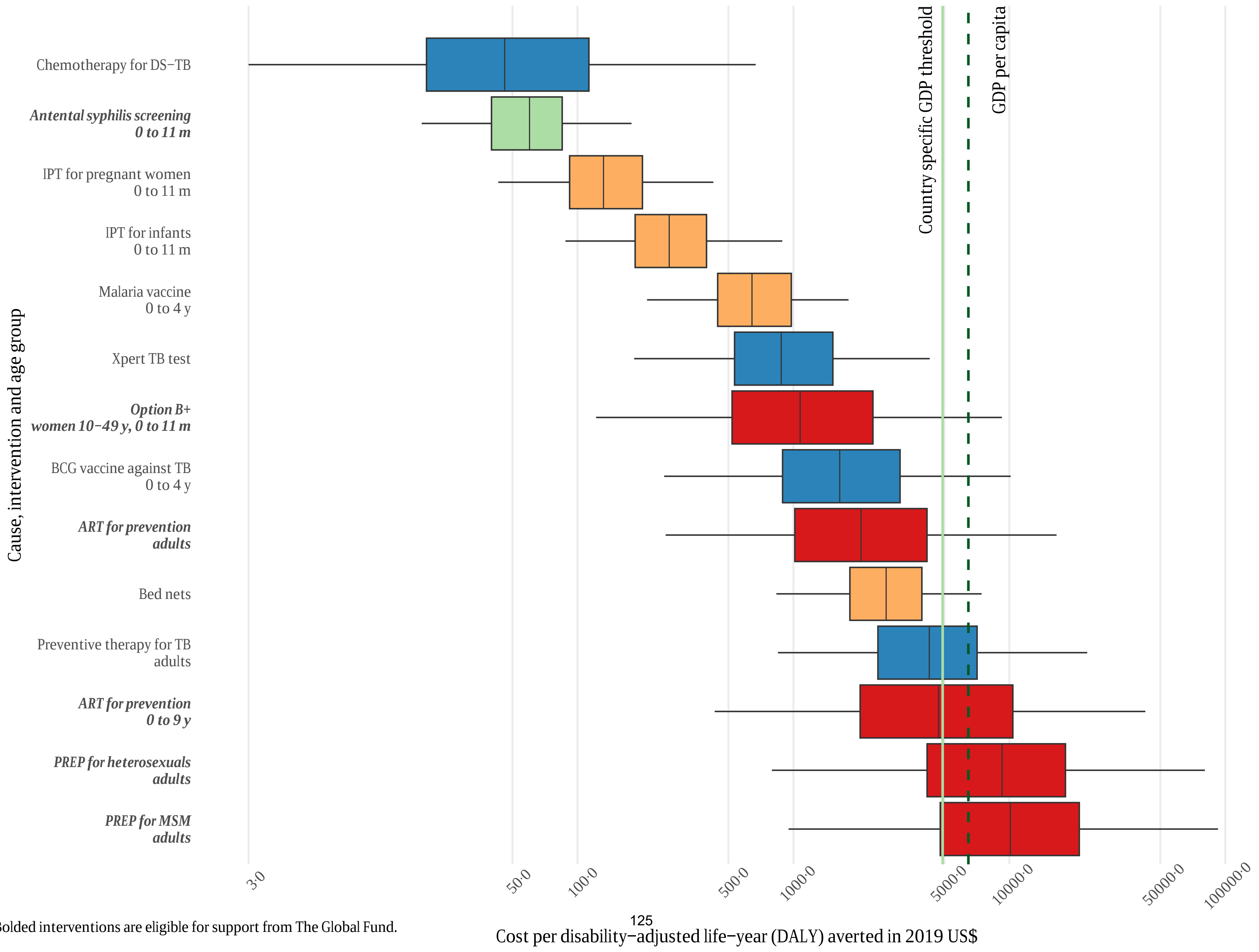

Bolded interventions are eligible for support from The Global Fund.

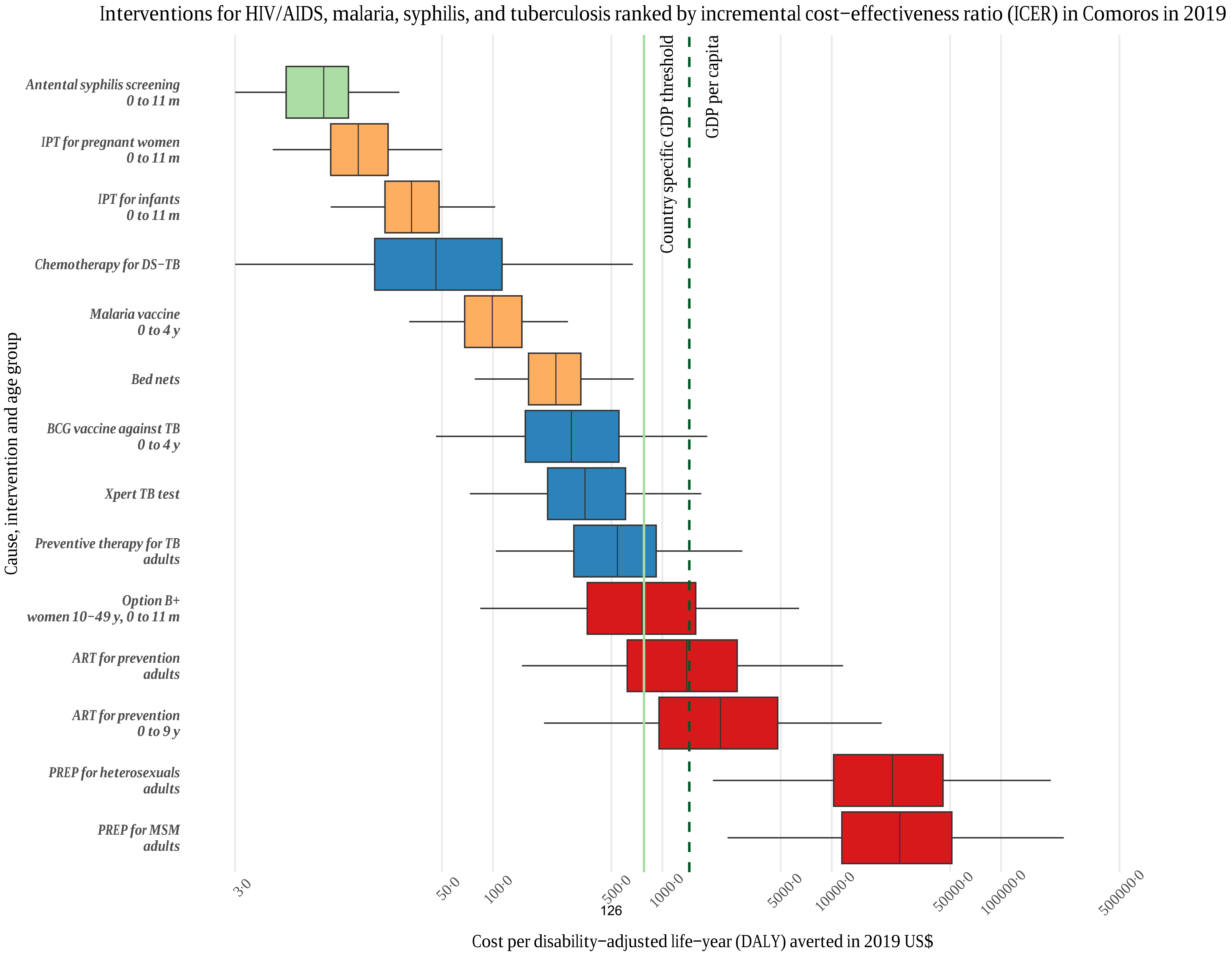
Interventions for HIV/AIDS, malaria, syphilis, and tuberculosis ranked by incremental cost−effectiveness ratio (ICER) in Comoros in 2019
Country specific GDP threshold
GDP per capita
Cause, intervention and age group
Antental syphilis screening
0 to 11 m
IPT for pregnant women
0 to 11 m
IPT for infants
0 to 11 m
Chemotherapy for DS−TB
Malaria vaccine
0 to 4 y
Bed nets
BCG vaccine against TB
0 to 4 y
Xpert TB test
Preventive therapy for TB
adults
Option B+
women 10−49 y, 0 to 11 m
ART for prevention
adults
ART for prevention
0 to 9 y
PREP for heterosexuals
adults
PREP for MSM
adults
3·0
50·0
100·0
500·0
1000·0
5000·0
10000·0
50000·0
100000·0
500000·0
Cost per disability−adjusted life−year (DALY) averted in 2019 US$

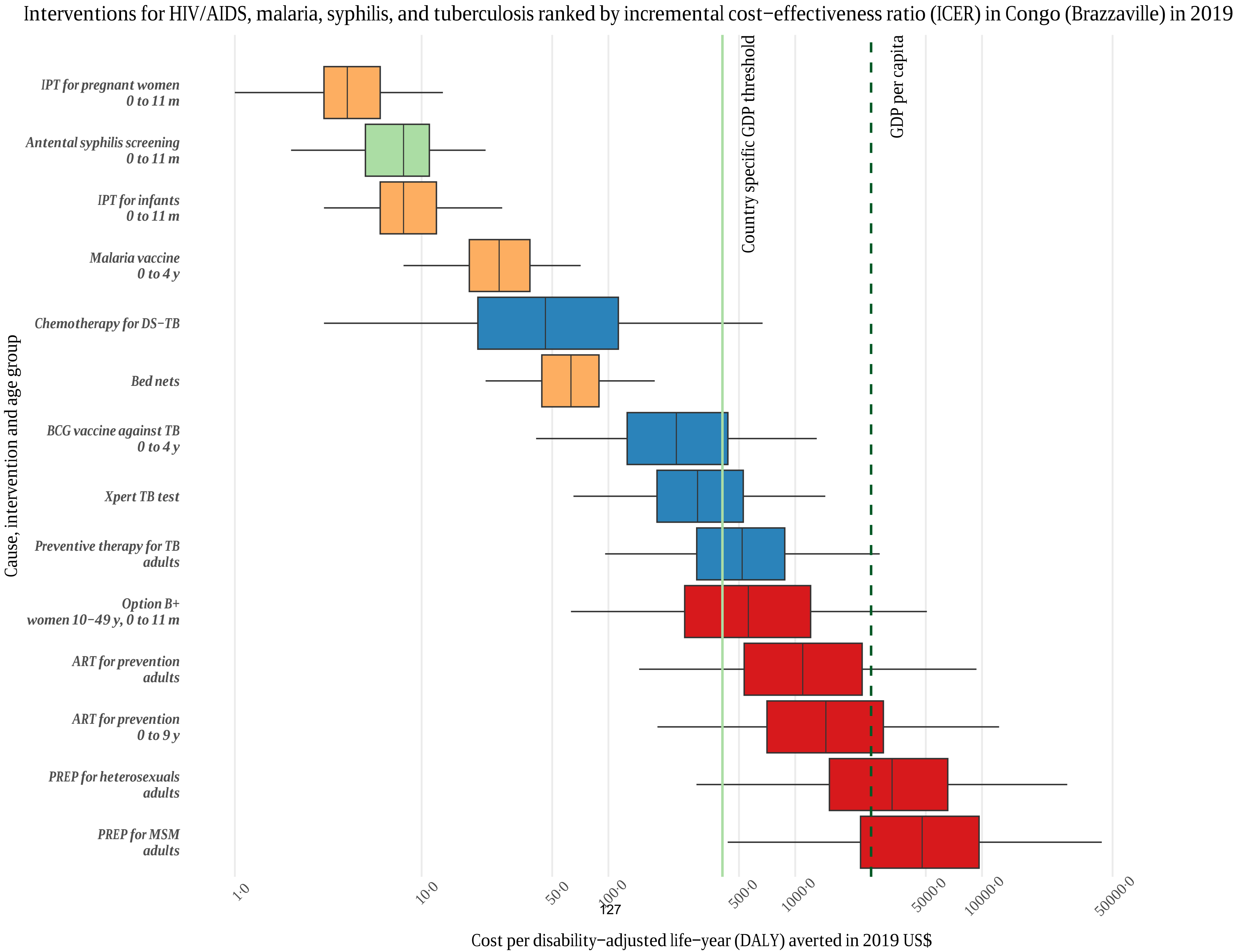

Interventions for HIV/AIDS, malaria, syphilis, and tuberculosis ranked by incremental cost−effectiveness ratio (ICER) in Congo (Brazzaville) in 2019
Country specific GDP threshold
GDP per capita
IPT for pregnant women
0 to 11 m
Antental syphilis screening
0 to 11 m
IPT for infants
0 to 11 m
Malaria vaccine
0 to 4 y
Chemotherapy for DS−TB
Bed nets
BCG vaccine against TB
0 to 4 y
Xpert TB test
Preventive therapy for TB
adults
Option B+
women 10−49 y, 0 to 11 m
ART for prevention
adults
ART for prevention
0 to 9 y
PREP for heterosexuals
adults
PREP for MSM
adults
Cause, intervention and age group
1·0
10·0
50·0
100·0
500·0
1000·0
5000·0
10000·0
50000·0
Cost per disability−adjusted life−year (DALY) averted in 2019 US$

Interventions for HIV/AIDS, malaria, syphilis, and tuberculosis ranked by incremental cost−effectiveness ratio (ICER) in Costa Rica in 2019

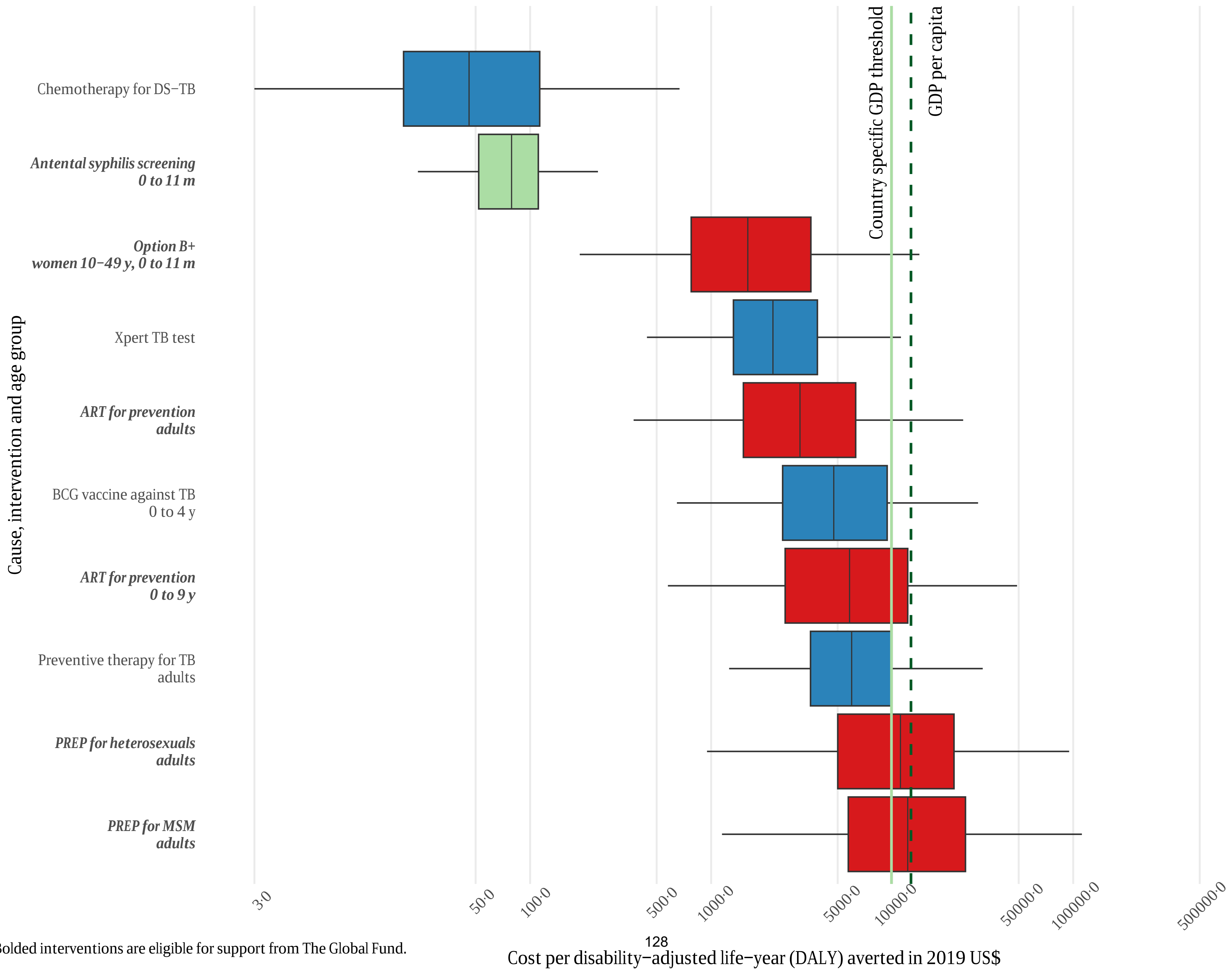

Bolded interventions are eligible for support from The Global Fund.

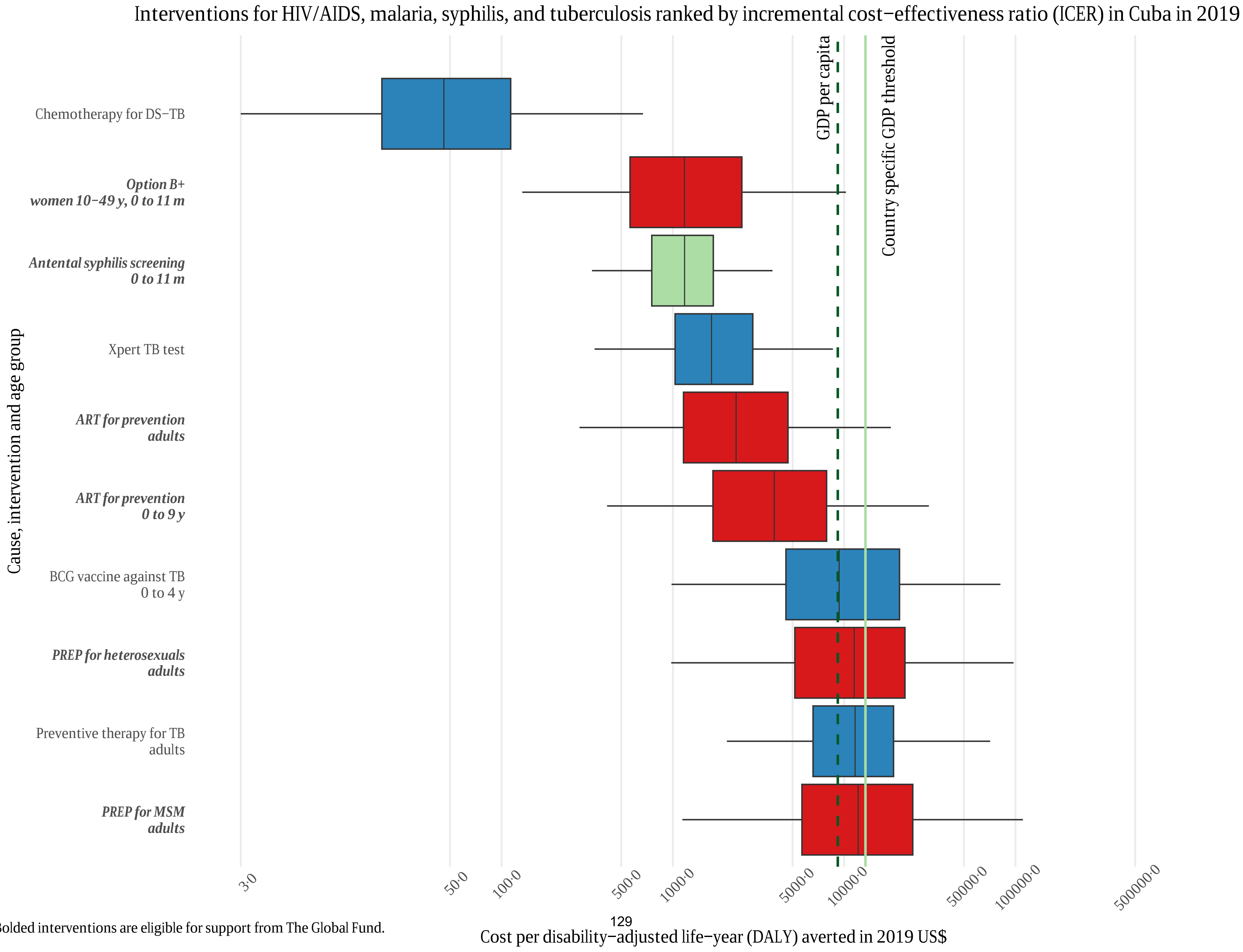

Bolded interventions are eligible for support from The Global Fund.

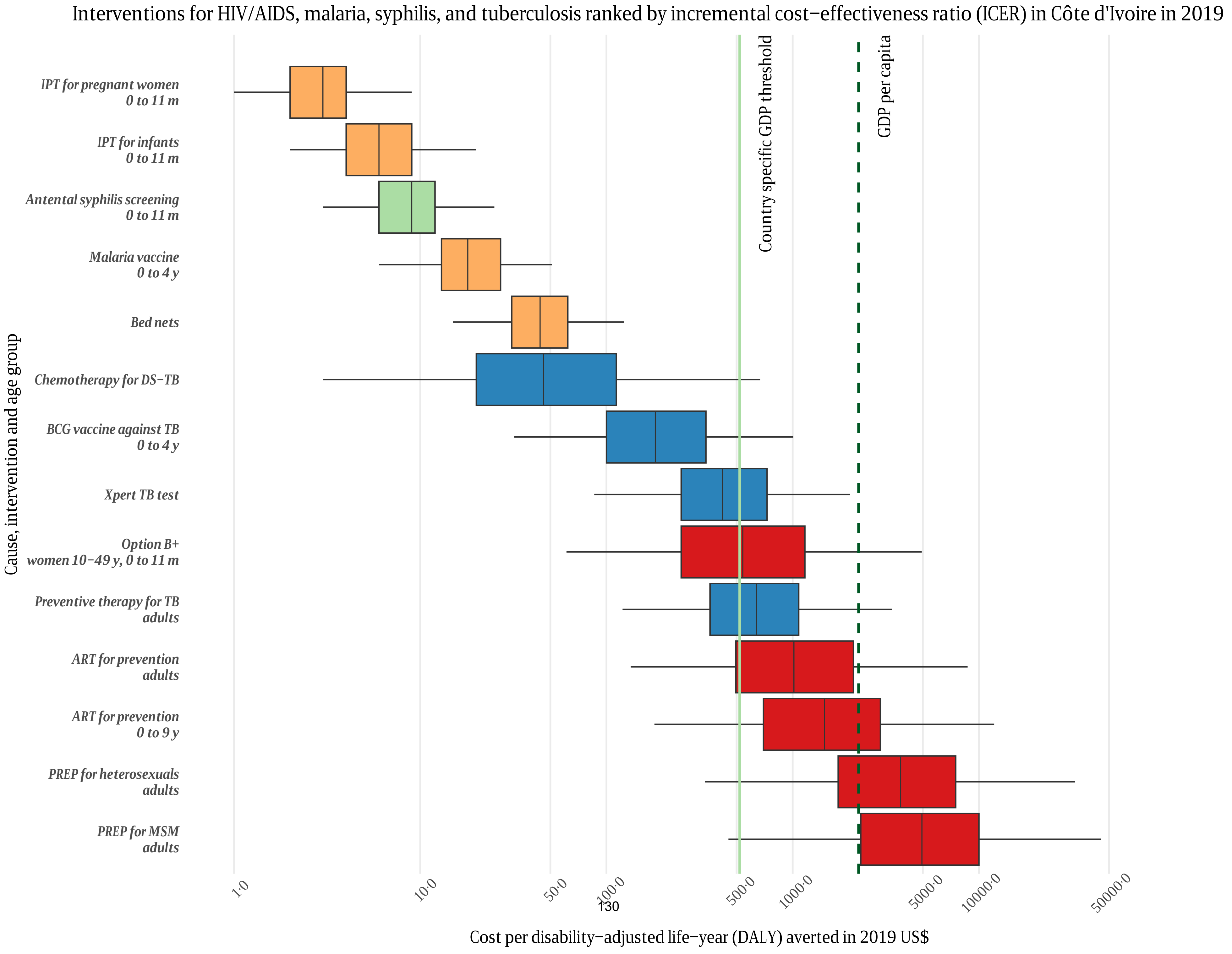
Interventions for HIV/AIDS, malaria, syphilis, and tuberculosis ranked by incremental cost−effectiveness ratio (ICER) in Côte d'Ivoire in 2019
Country specific GDP threshold
GDP per capita
IPT for pregnant women
0 to 11 m
IPT for infants
0 to 11 m
Antental syphilis screening
0 to 11 m
Malaria vaccine
0 to 4 y
Bed nets
Chemotherapy for DS−TB
BCG vaccine against TB
0 to 4 y
Xpert TB test
Option B+
women 10−49 y, 0 to 11 m
Preventive therapy for TB
adults
ART for prevention
adults
ART for prevention
0 to 9 y
PREP for heterosexuals
adults
PREP for MSM
adults
Cause, intervention and age group
1.0
10.0
50.0
100.0
500.0
1000.0
5000.0
10000.0
50000.0
Cost per disability−adjusted life−year (DALY) averted in 2019 US$

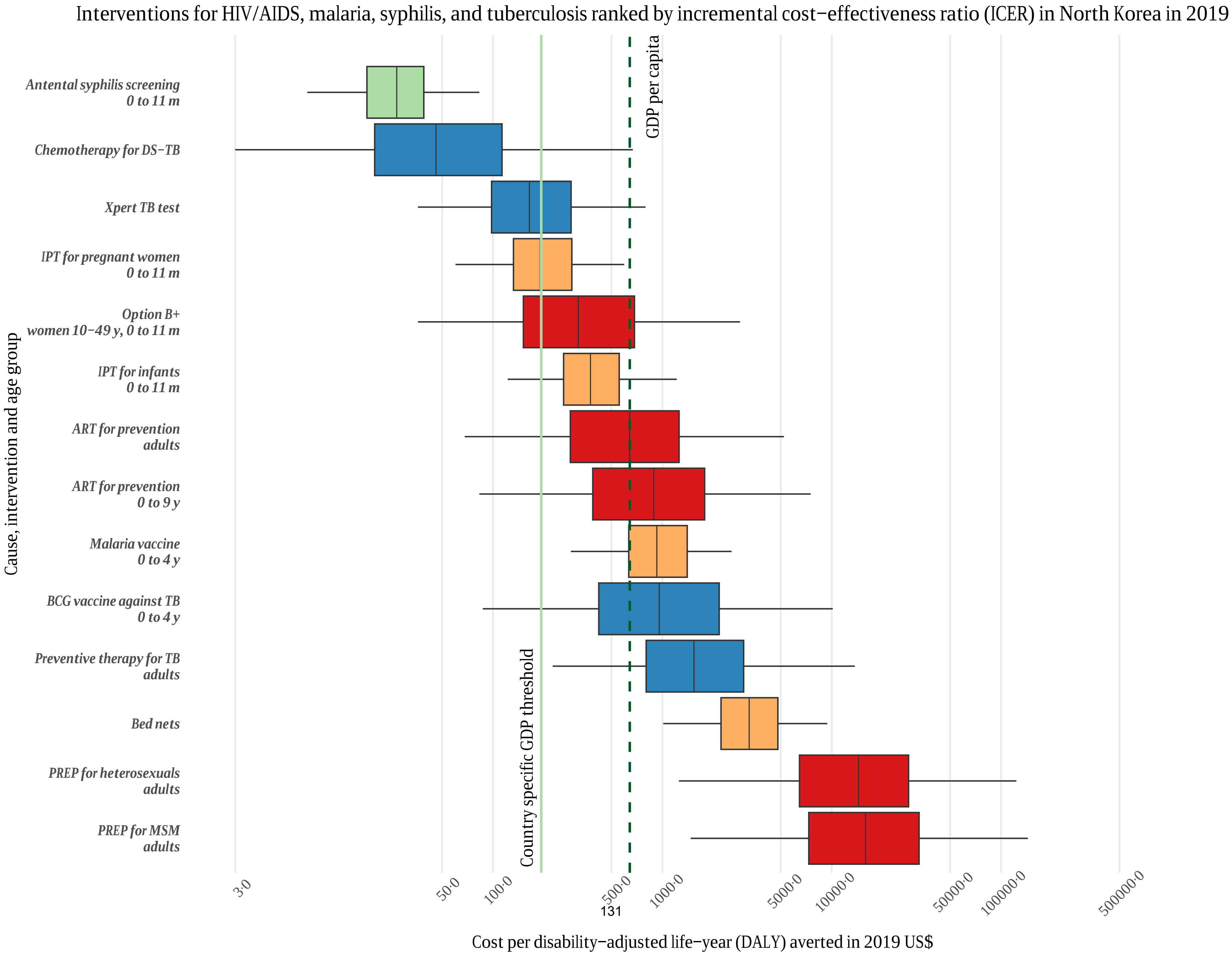
Interventions for HIV/AIDS, malaria, syphilis, and tuberculosis ranked by incremental cost−effectiveness ratio (ICER) in North Korea in 2019
Antental syphilis screening 0 to 11 m
Chemotherapy for DS−TB
Xpert TB test
IPT for pregnant women 0 to 11 m
Option B+ women 10−49 y, 0 to 11 m
IPT for infants 0 to 11 m
ART for prevention adults
ART for prevention 0 to 9 y
Malaria vaccine 0 to 4 y
BCG vaccine against TB 0 to 4 y
Preventive therapy for TB adults
Bed nets
PREP for heterosexuals adults
PREP for MSM adults
Cause, intervention and age group
GDP per capita
Country specific GDP threshold
3·0
50·0
100·0
500·0
131
1000·0
5000·0
10000·0
50000·0
100000·0
500000·0
Cost per disability−adjusted life−year (DALY) averted in 2019 US$

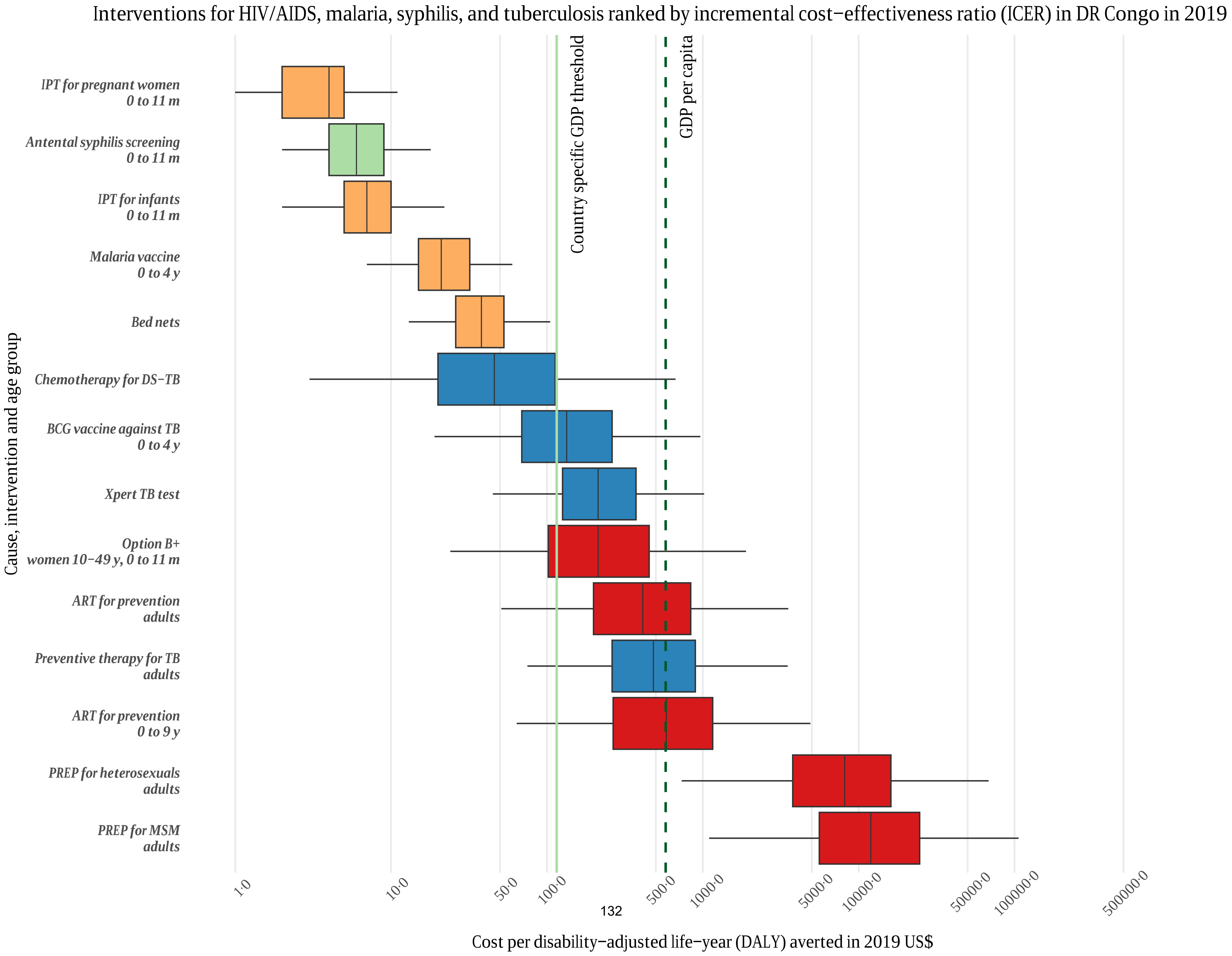
Interventions for HIV/AIDS, malaria, syphilis, and tuberculosis ranked by incremental cost−effectiveness ratio (ICER) in DR Congo in 2019
Country specific GDP threshold
GDP per capita
IPT for pregnant women 0 to 11 m
Antental syphilis screening 0 to 11 m
IPT for infants 0 to 11 m
Malaria vaccine 0 to 4 y
Bed nets
Chemotherapy for DS−TB
BCG vaccine against TB 0 to 4 y
Xpert TB test
Option B+ women 10−49 y, 0 to 11 m
ART for prevention adults
Preventive therapy for TB adults
ART for prevention 0 to 9 y
PREP for heterosexuals adults
PREP for MSM adults
Cause, intervention and age group
1·0
10·0
50·0
100·0
500·0
1000·0
5000·0
10000·0
50000·0
100000·0
500000·0
Cost per disability−adjusted life−year (DALY) averted in 2019 US$

Interventions for HIV/AIDS, malaria, syphilis, and tuberculosis ranked by incremental cost−effectiveness ratio (ICER) in Djibouti in 2019

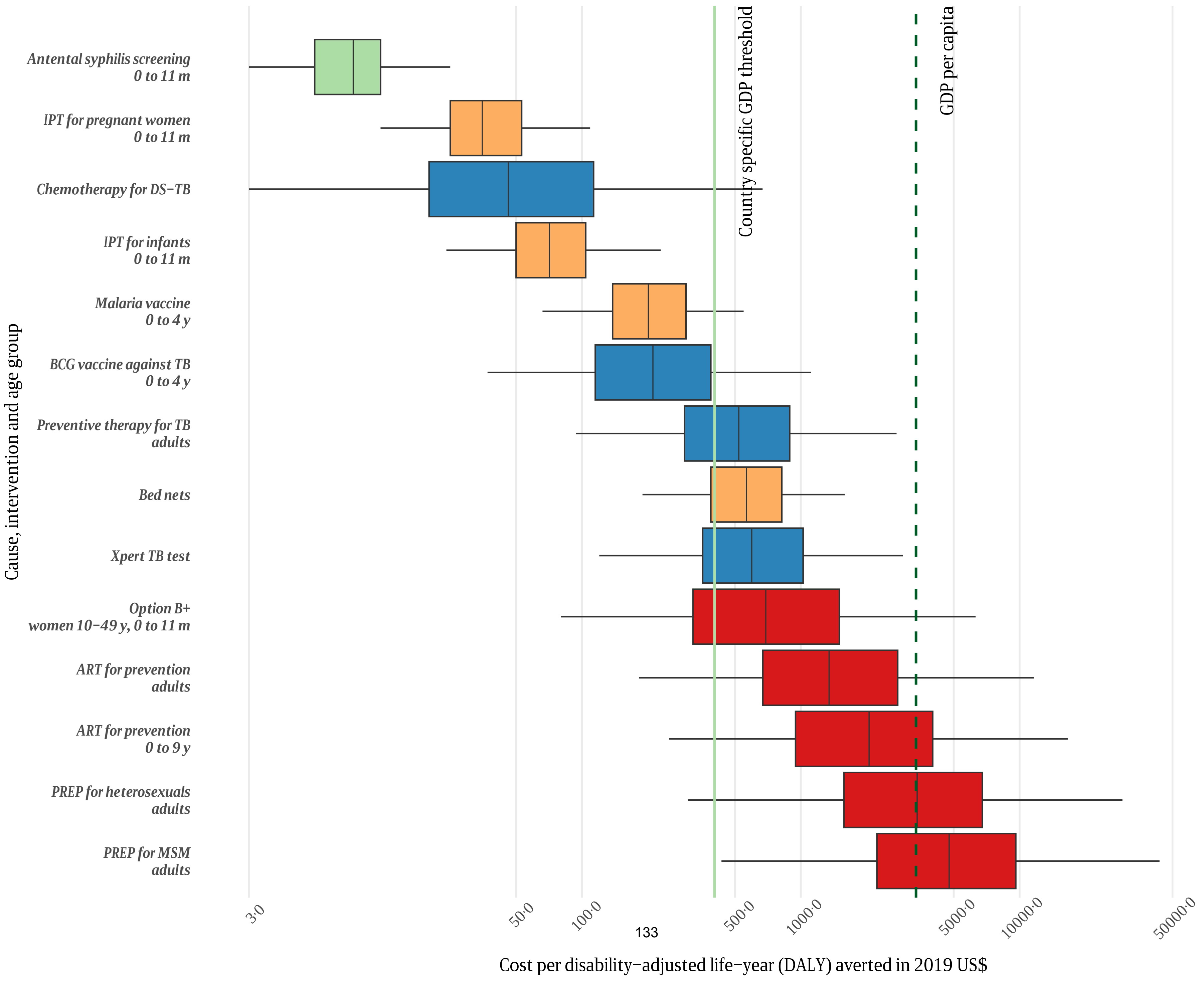

Interventions for HIV/AIDS, malaria, syphilis, and tuberculosis ranked by incremental cost−effectiveness ratio (ICER) in Dominica in 2019

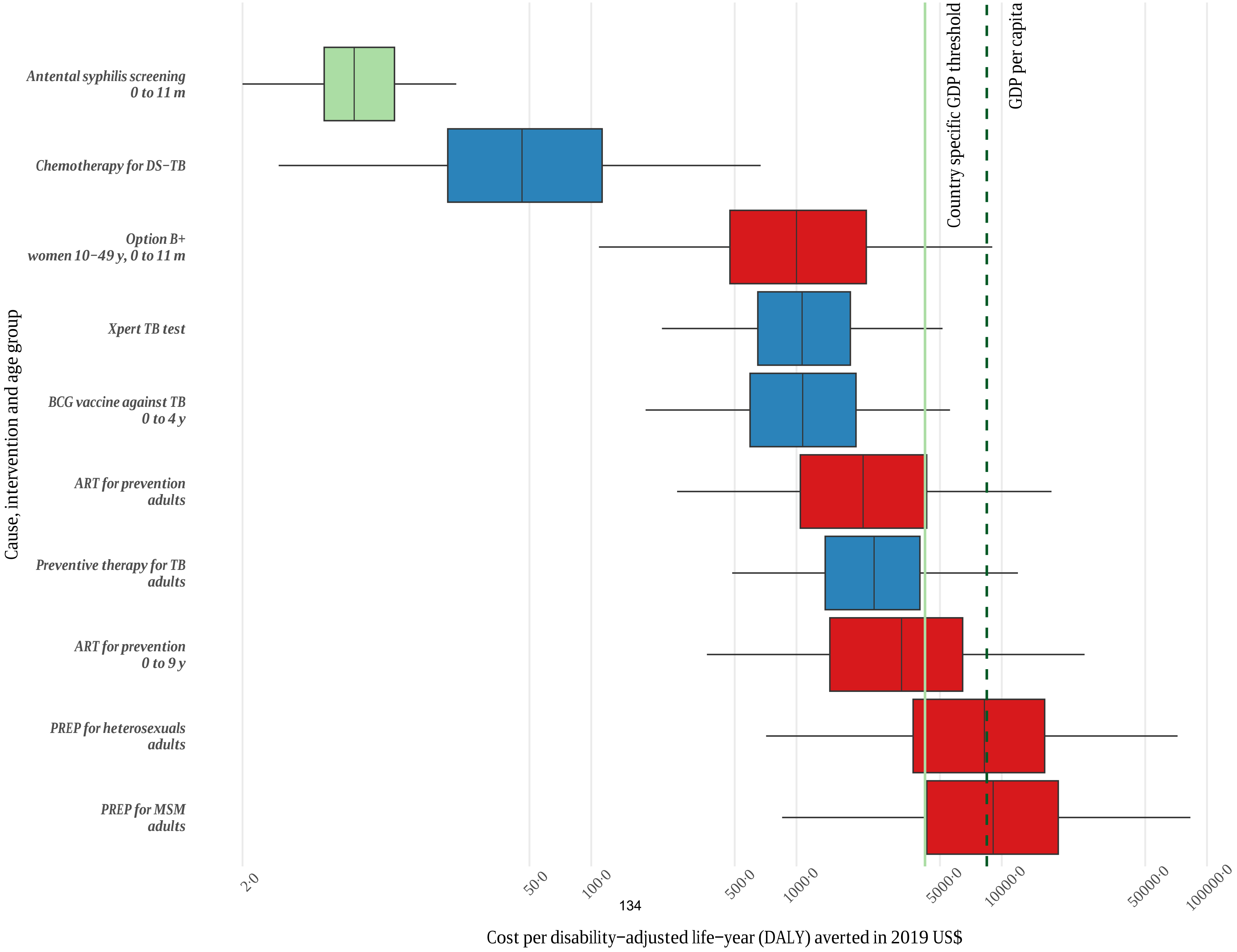

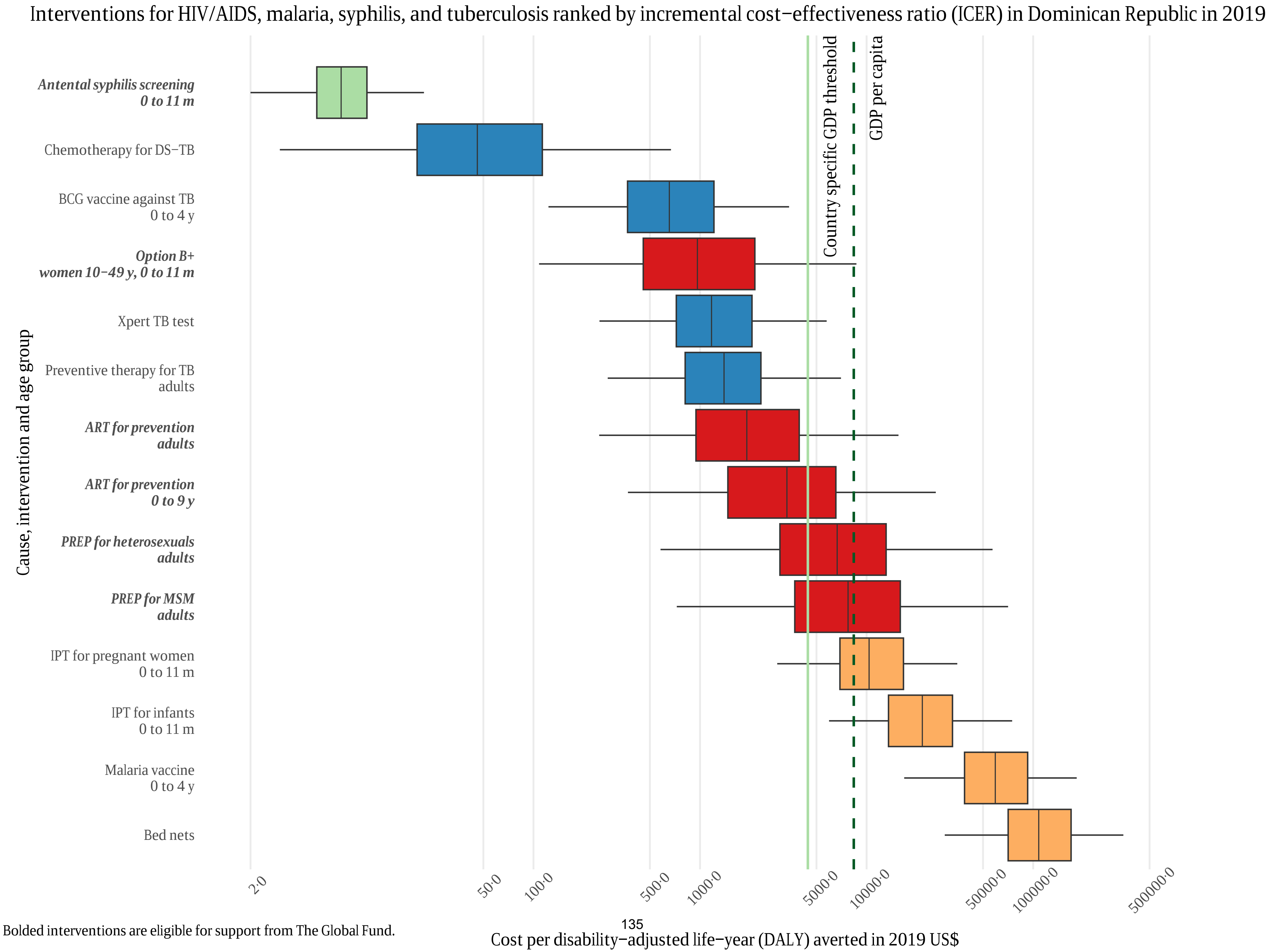

Bolded interventions are eligible for support from The Global Fund.

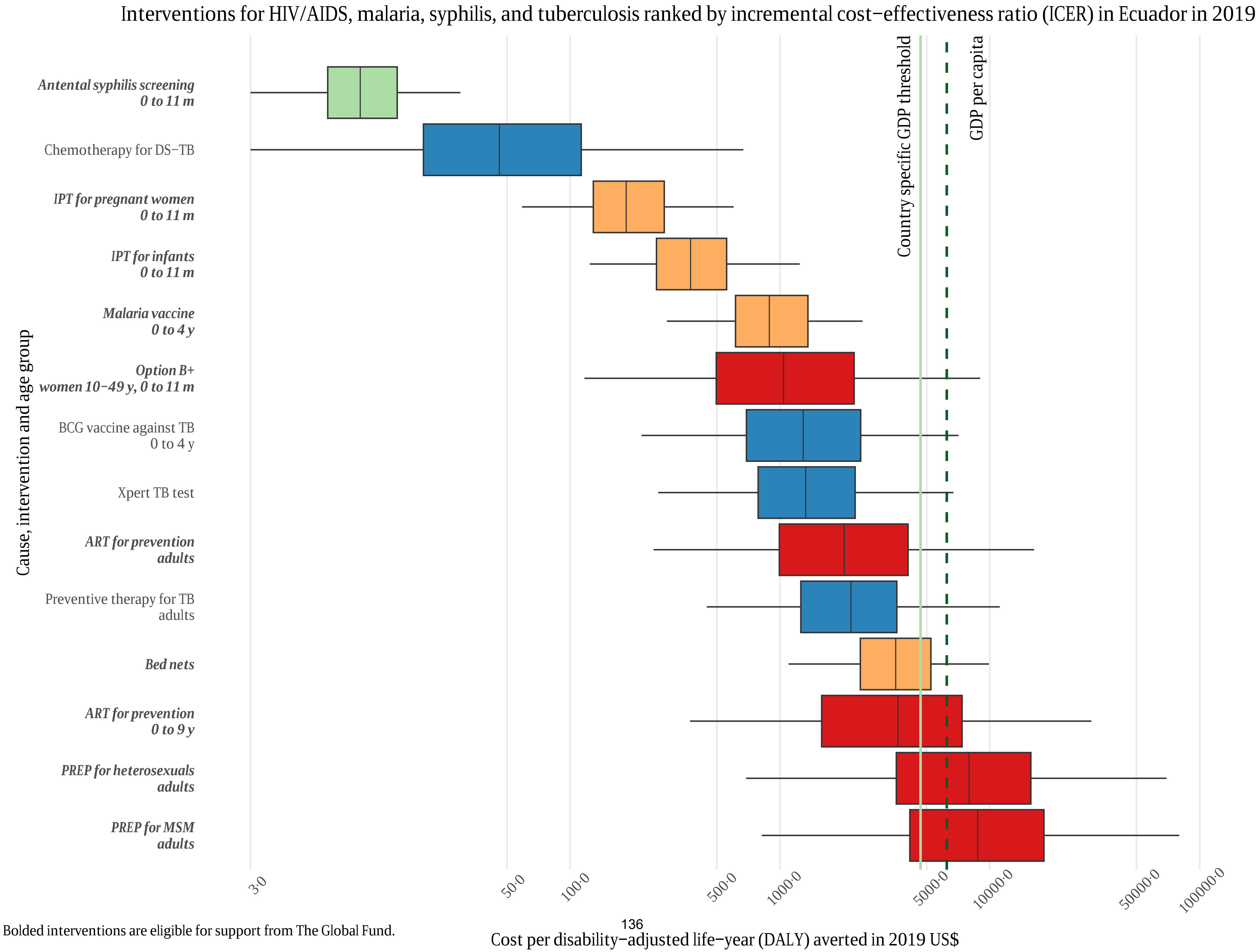

Interventions for HIV/AIDS, malaria, syphilis, and tuberculosis ranked by incremental cost−effectiveness ratio (ICER) in Ecuador in 2019
Cause, intervention and age group
Antental syphilis screening
0 to 11 m
Chemotherapy for DS−TB
IPT for pregnant women
0 to 11 m
IPT for infants
0 to 11 m
Malaria vaccine
0 to 4 y
Option B+
women 10−49 y, 0 to 11 m
BCG vaccine against TB
0 to 4 y
Xpert TB test
ART for prevention
adults
Preventive therapy for TB
adults
Bed nets
ART for prevention
0 to 9 y
PREP for heterosexuals
adults
PREP for MSM
adults
Country specific GDP threshold
GDP per capita
3·0
50·0
100·0
500·0
1000·0
5000·0
10000·0
50000·0
100000·0
Bolded interventions are eligible for support from The Global Fund.
Cost per disability−adjusted life−year (DALY) averted in 2019 US$

Interventions for HIV/AIDS, malaria, syphilis, and tuberculosis ranked by incremental cost−effectiveness ratio (ICER) in Egypt in 2019

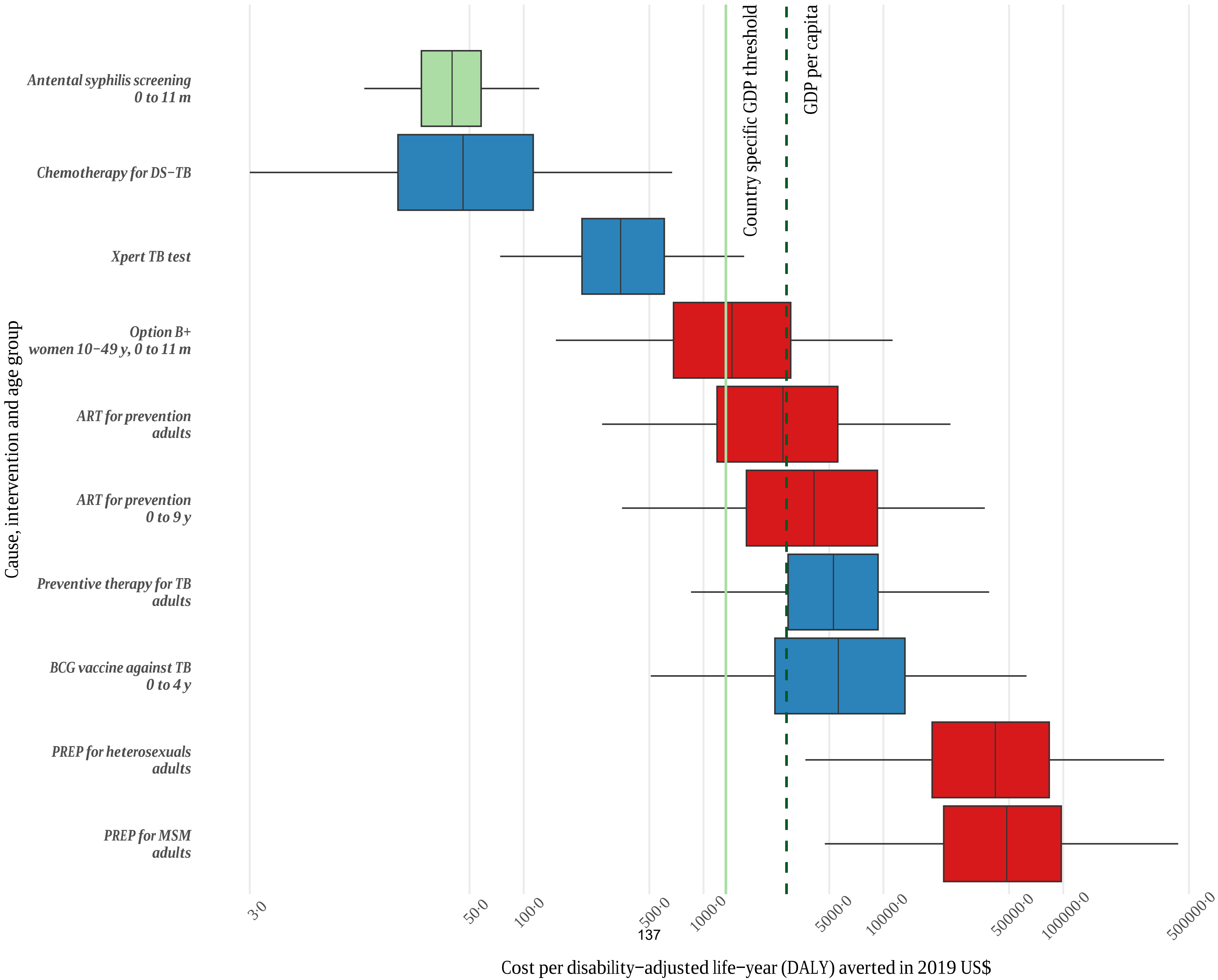

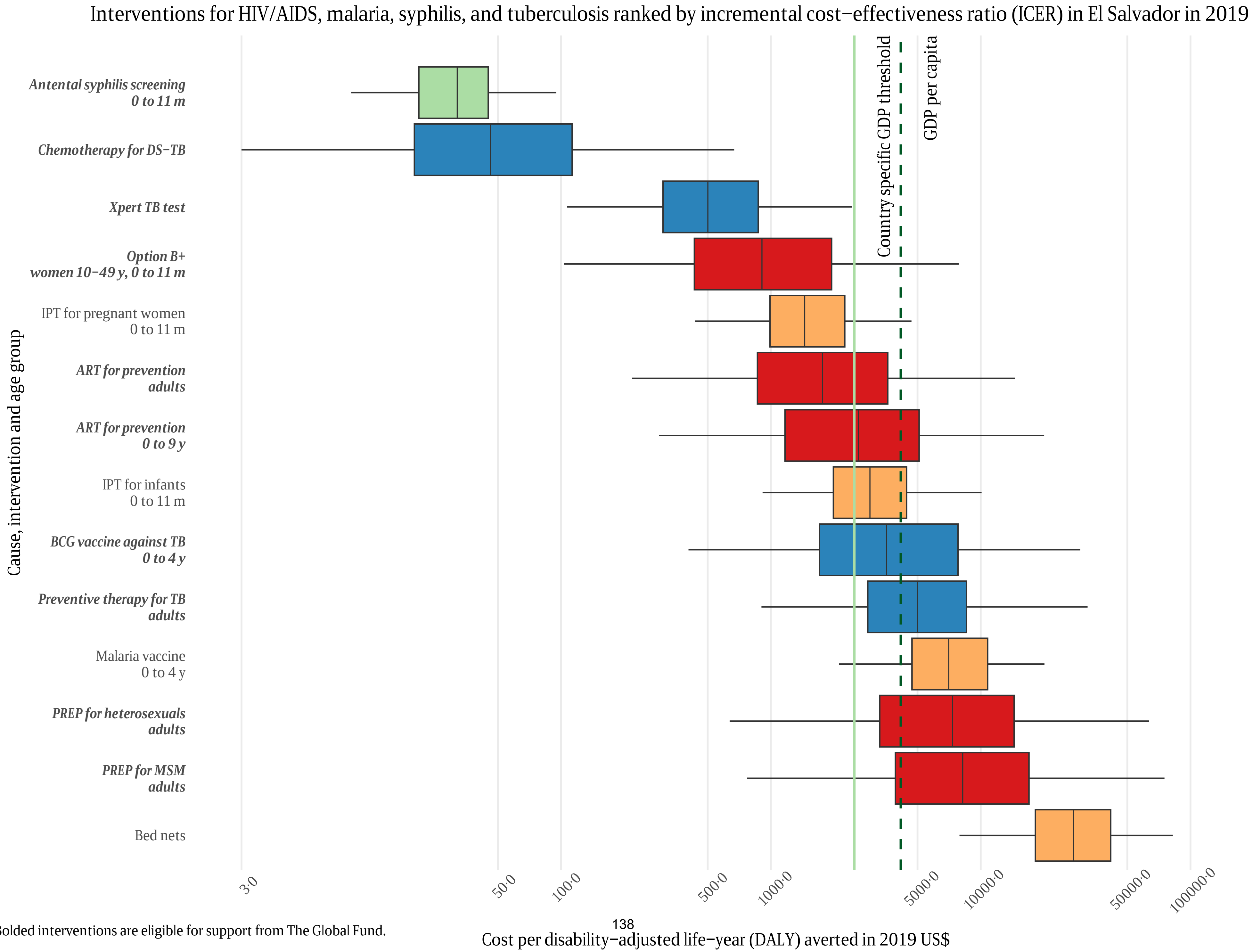

Bolded interventions are eligible for support from The Global Fund.

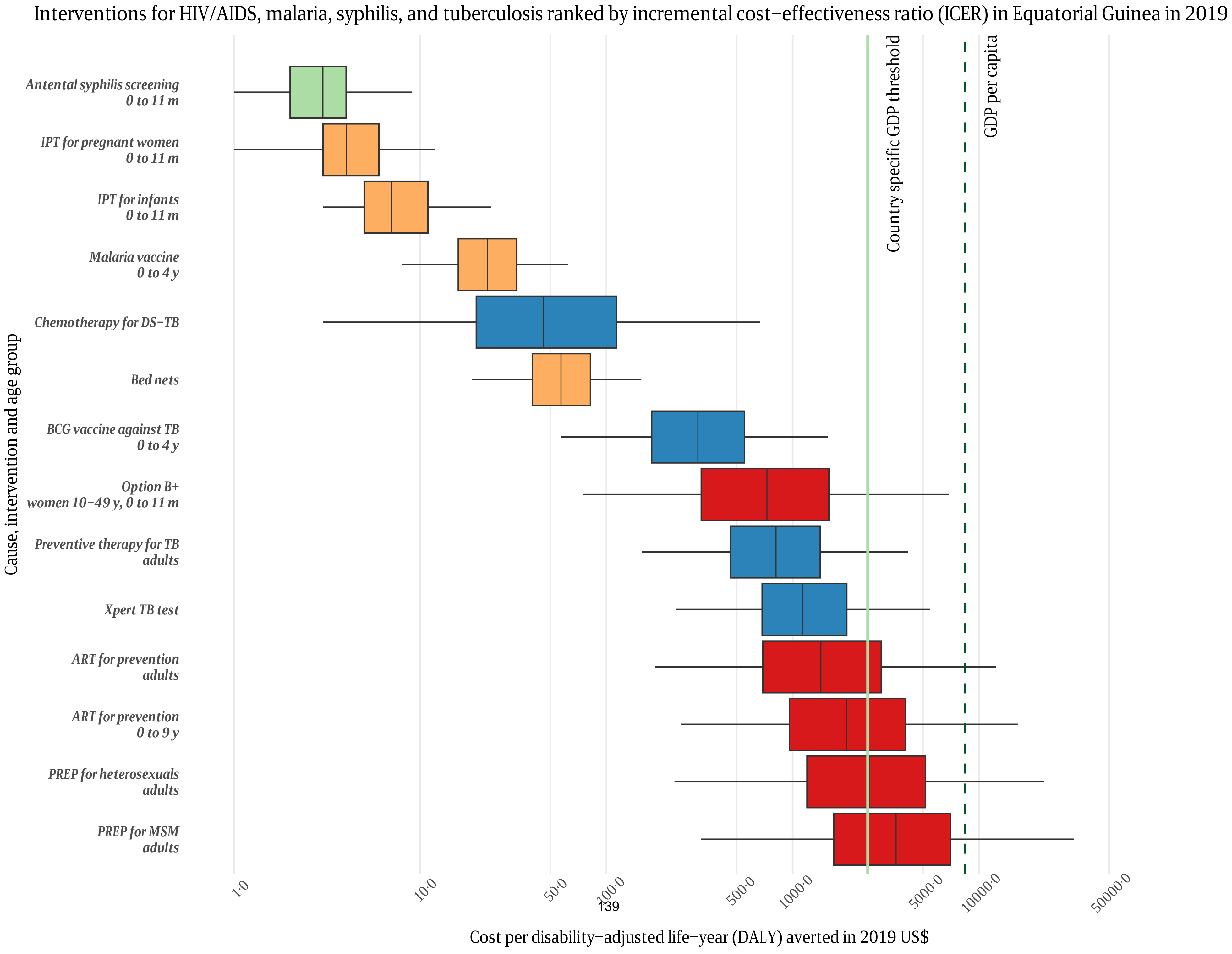
Interventions for HIV/AIDS, malaria, syphilis, and tuberculosis ranked by incremental cost−effectiveness ratio (ICER) in Equatorial Guinea in 2019
Country specific GDP threshold
GDP per capita
Cause, intervention and age group
Antental syphilis screening
0 to 11 m
IPT for pregnant women
0 to 11 m
IPT for infants
0 to 11 m
Malaria vaccine
0 to 4 y
Chemotherapy for DS−TB
Bed nets
BCG vaccine against TB
0 to 4 y
Option B+
women 10−49 y, 0 to 11 m
Preventive therapy for TB
adults
Xpert TB test
ART for prevention
adults
ART for prevention
0 to 9 y
PREP for heterosexuals
adults
PREP for MSM
adults
1.0
10.0
50.0
100.0
500.0
1000.0
5000.0
10000.0
50000.0
Cost per disability−adjusted life−year (DALY) averted in 2019 US$

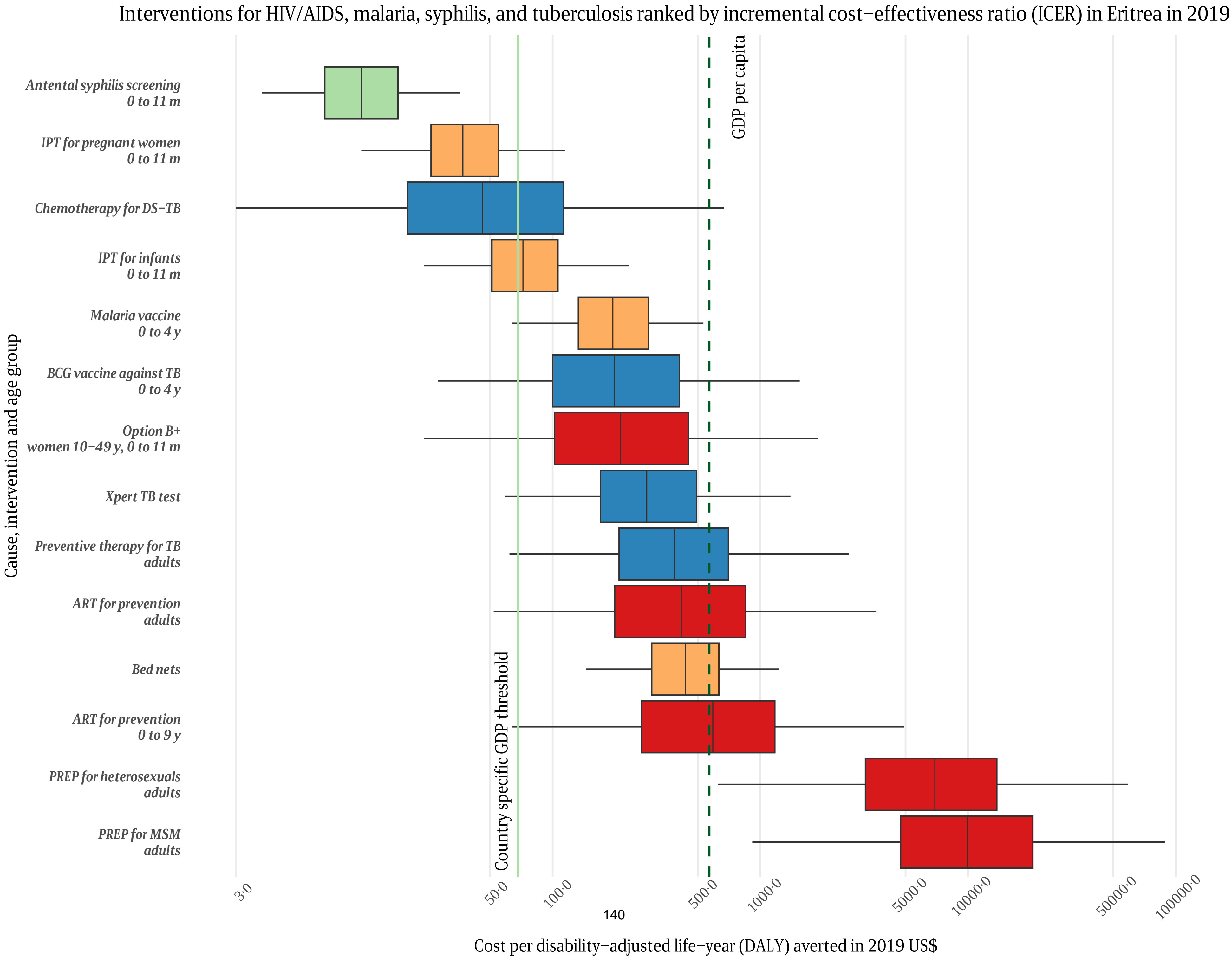

Interventions for HIV/AIDS, malaria, syphilis, and tuberculosis ranked by incremental cost−effectiveness ratio (ICER) in Eritrea in 2019
Antental syphilis screening 0 to 11 m
IPT for pregnant women 0 to 11 m
Chemotherapy for DS−TB
IPT for infants 0 to 11 m
Malaria vaccine 0 to 4 y
BCG vaccine against TB 0 to 4 y
Option B+ women 10−49 y, 0 to 11 m
Xpert TB test
Preventive therapy for TB adults
ART for prevention adults
Bed nets
ART for prevention 0 to 9 y
PREP for heterosexuals adults
PREP for MSM adults
Cause, intervention and age group
GDP per capita
Country specific GDP threshold
3·0
50·0
100·0
500·0
1000·0
5000·0
10000·0
50000·0
100000·0
140
Cost per disability−adjusted life−year (DALY) averted in 2019 US$

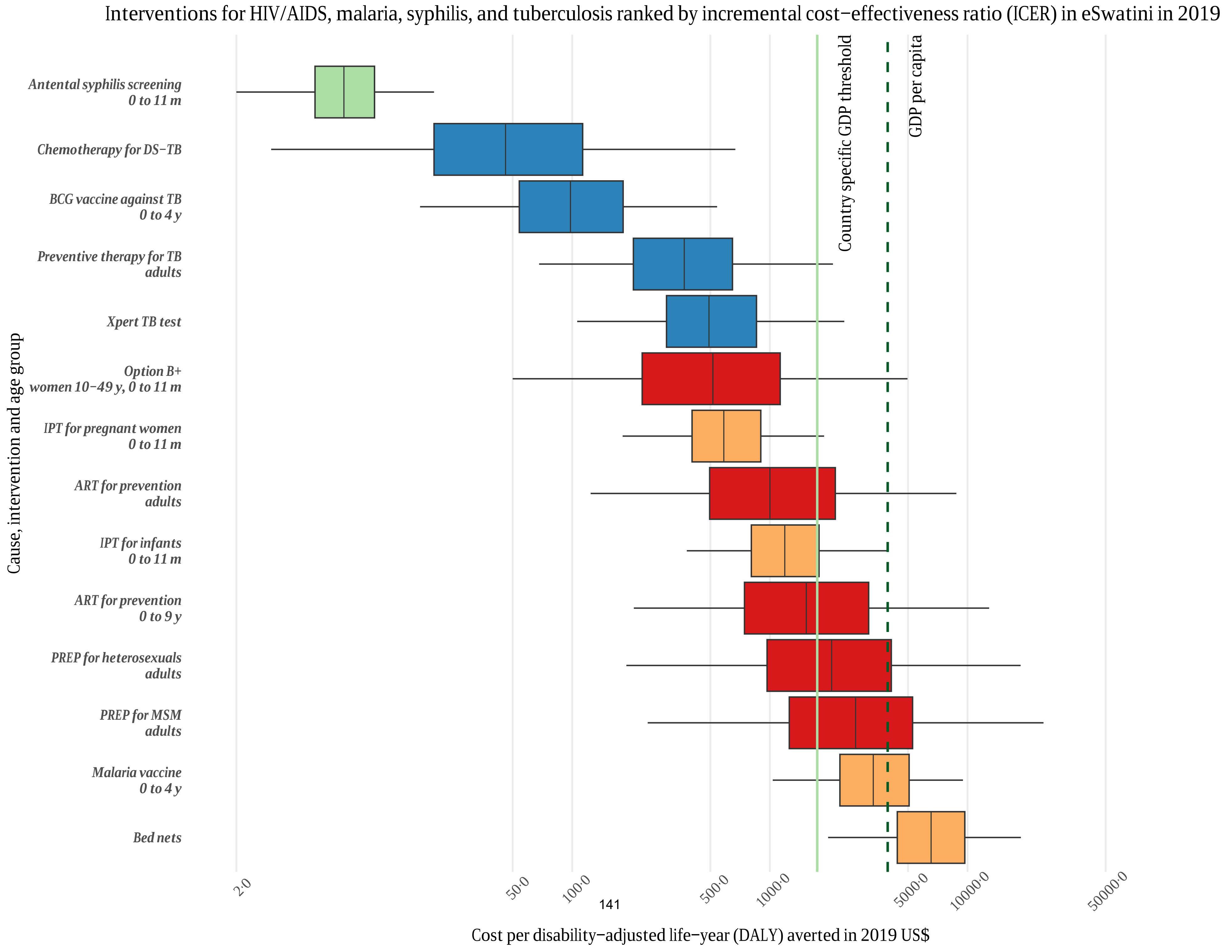

Interventions for HIV/AIDS, malaria, syphilis, and tuberculosis ranked by incremental cost−effectiveness ratio (ICER) in eSwatini in 2019
Antental syphilis screening
0 to 11 m
Chemotherapy for DS−TB
BCG vaccine against TB
0 to 4 y
Preventive therapy for TB
adults
Xpert TB test
Option B+
women 10−49 y, 0 to 11 m
IPT for pregnant women
0 to 11 m
ART for prevention
adults
IPT for infants
0 to 11 m
ART for prevention
0 to 9 y
PREP for heterosexuals
adults
PREP for MSM
adults
Malaria vaccine
0 to 4 y
Bed nets
Country specific GDP threshold
GDP per capita
Cause, intervention and age group
2·0
50·0
100·0
500·0
1000·0
5000·0
10000·0
50000·0
Cost per disability−adjusted life−year (DALY) averted in 2019 US$

Interventions for HIV/AIDS, malaria, syphilis, and tuberculosis ranked by incremental cost−effectiveness ratio (ICER) in Ethiopia in 2019

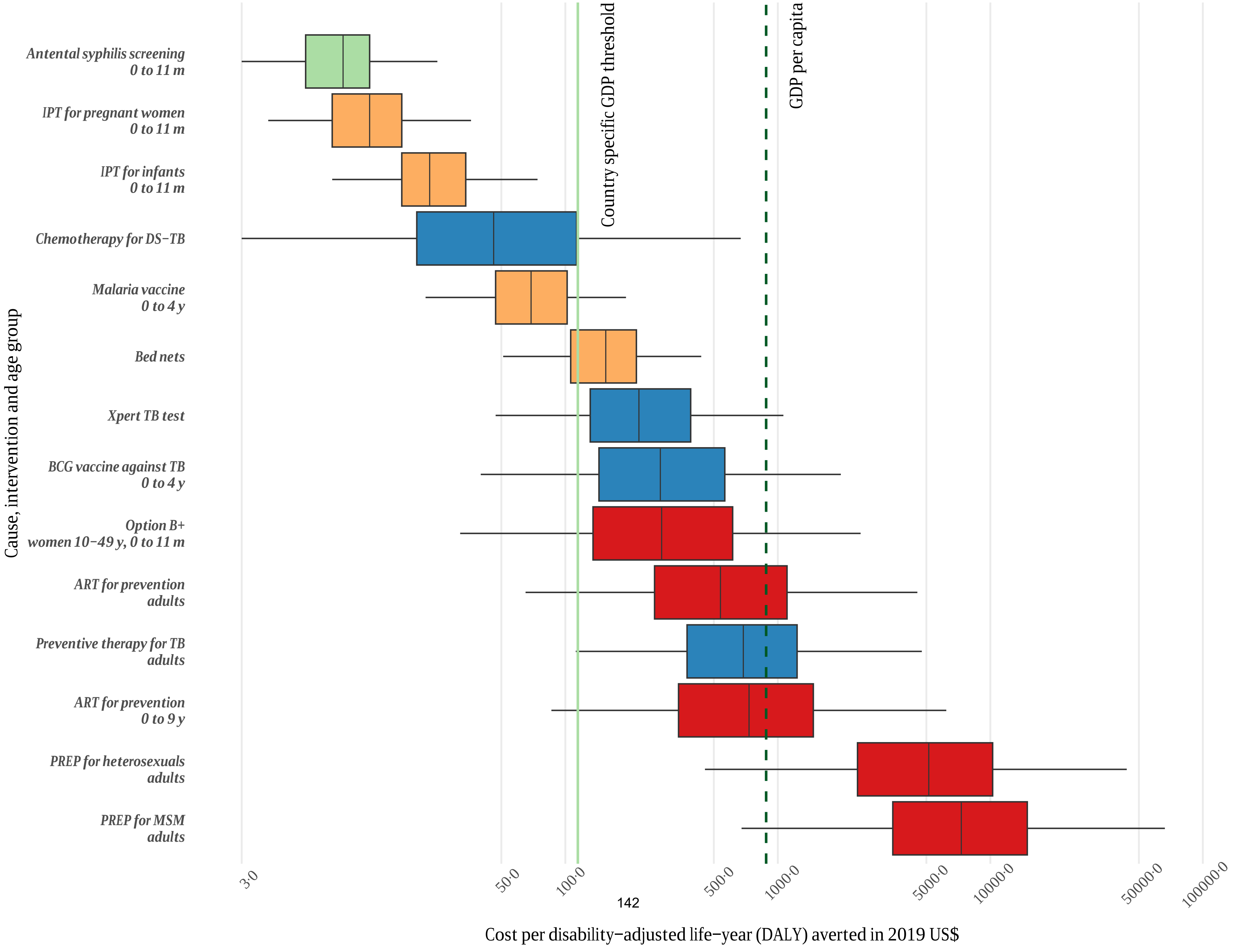

Interventions for HIV/AIDS, malaria, syphilis, and tuberculosis ranked by incremental cost−effectiveness ratio (ICER) in Fiji in 2019

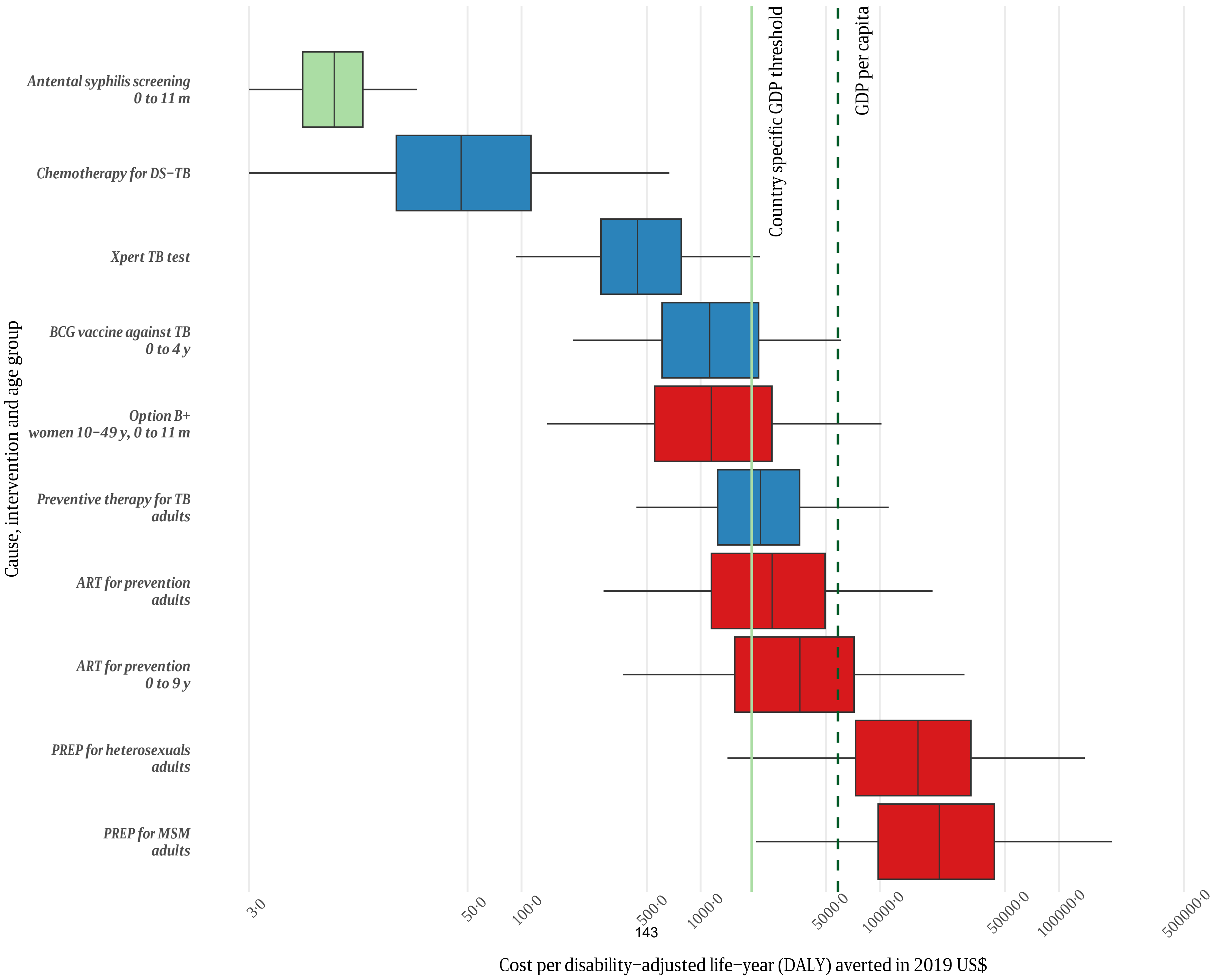

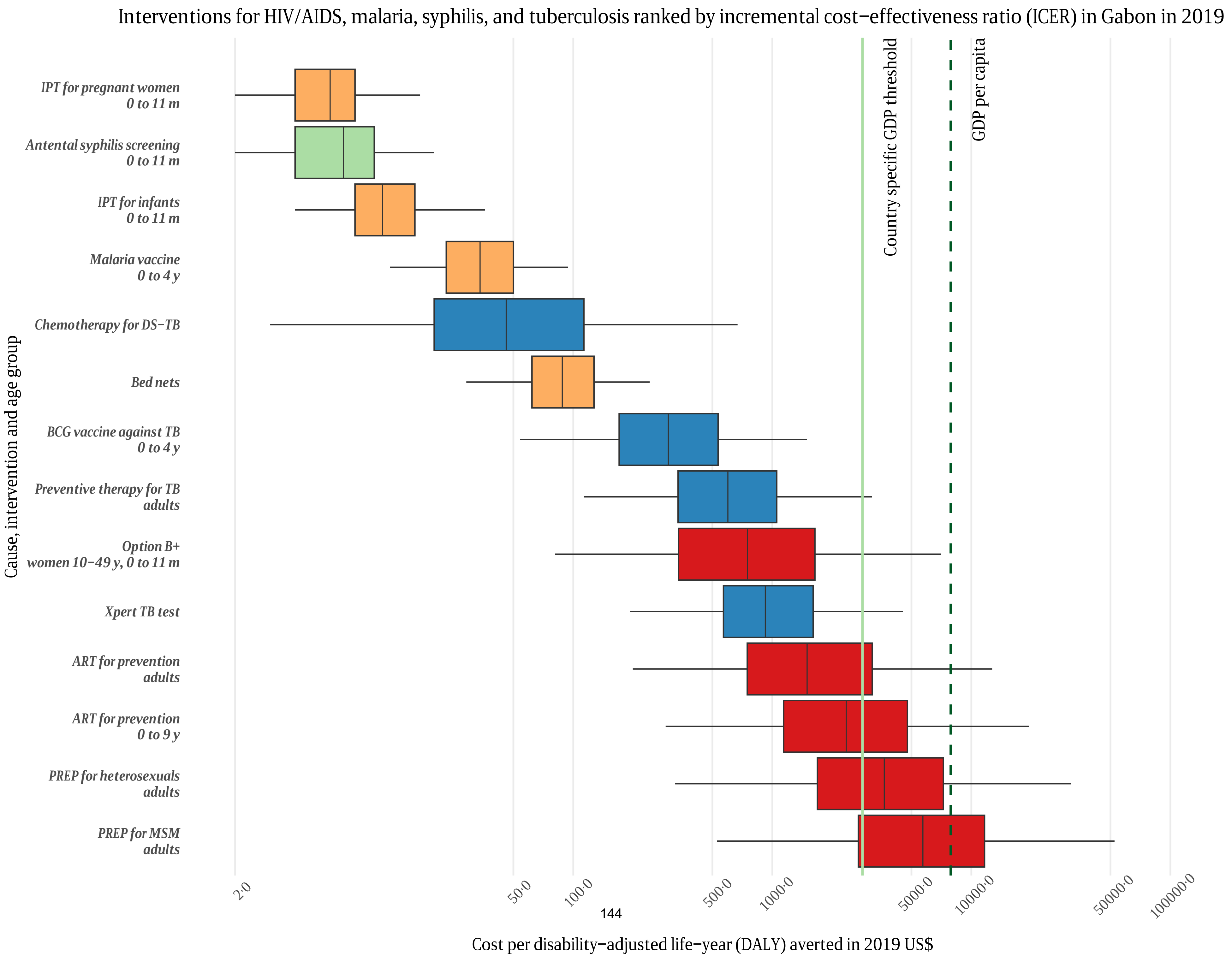
Interventions for HIV/AIDS, malaria, syphilis, and tuberculosis ranked by incremental cost−effectiveness ratio (ICER) in Gabon in 2019
IPT for pregnant women
0 to 11 m
Antental syphilis screening
0 to 11 m
IPT for infants
0 to 11 m
Malaria vaccine
0 to 4 y
Chemotherapy for DS−TB
Bed nets
BCG vaccine against TB
0 to 4 y
Preventive therapy for TB
adults
Option B+
women 10−49 y, 0 to 11 m
Xpert TB test
ART for prevention
adults
ART for prevention
0 to 9 y
PREP for heterosexuals
adults
PREP for MSM
adults
Cause, intervention and age group
Country specific GDP threshold
GDP per capita
2·0
50·0
100·0
500·0
1000·0
5000·0
10000·0
50000·0
100000·0
Cost per disability−adjusted life−year (DALY) averted in 2019 US$

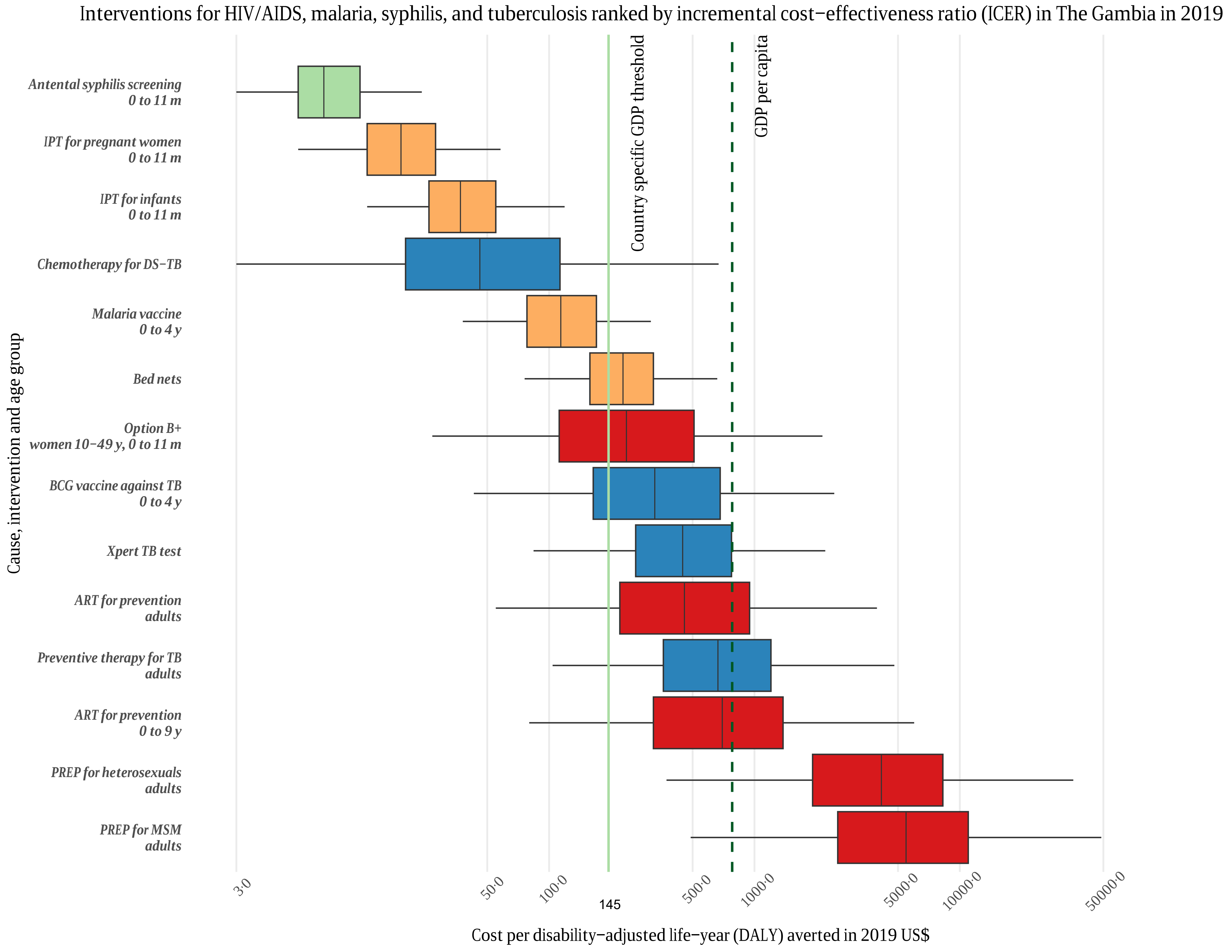
Interventions for HIV/AIDS, malaria, syphilis, and tuberculosis ranked by incremental cost−effectiveness ratio (ICER) in The Gambia in 2019
Country specific GDP threshold
GDP per capita
Antental syphilis screening
0 to 11 m
IPT for pregnant women
0 to 11 m
IPT for infants
0 to 11 m
Chemotherapy for DS−TB
Malaria vaccine
0 to 4 y
Bed nets
Option B+
women 10−49 y, 0 to 11 m
BCG vaccine against TB
0 to 4 y
Xpert TB test
ART for prevention
adults
Preventive therapy for TB
adults
ART for prevention
0 to 9 y
PREP for heterosexuals
adults
PREP for MSM
adults
Cause, intervention and age group
3·0
50·0
100·0
145
500·0
1000·0
5000·0
10000·0
50000·0
Cost per disability−adjusted life−year (DALY) averted in 2019 US$

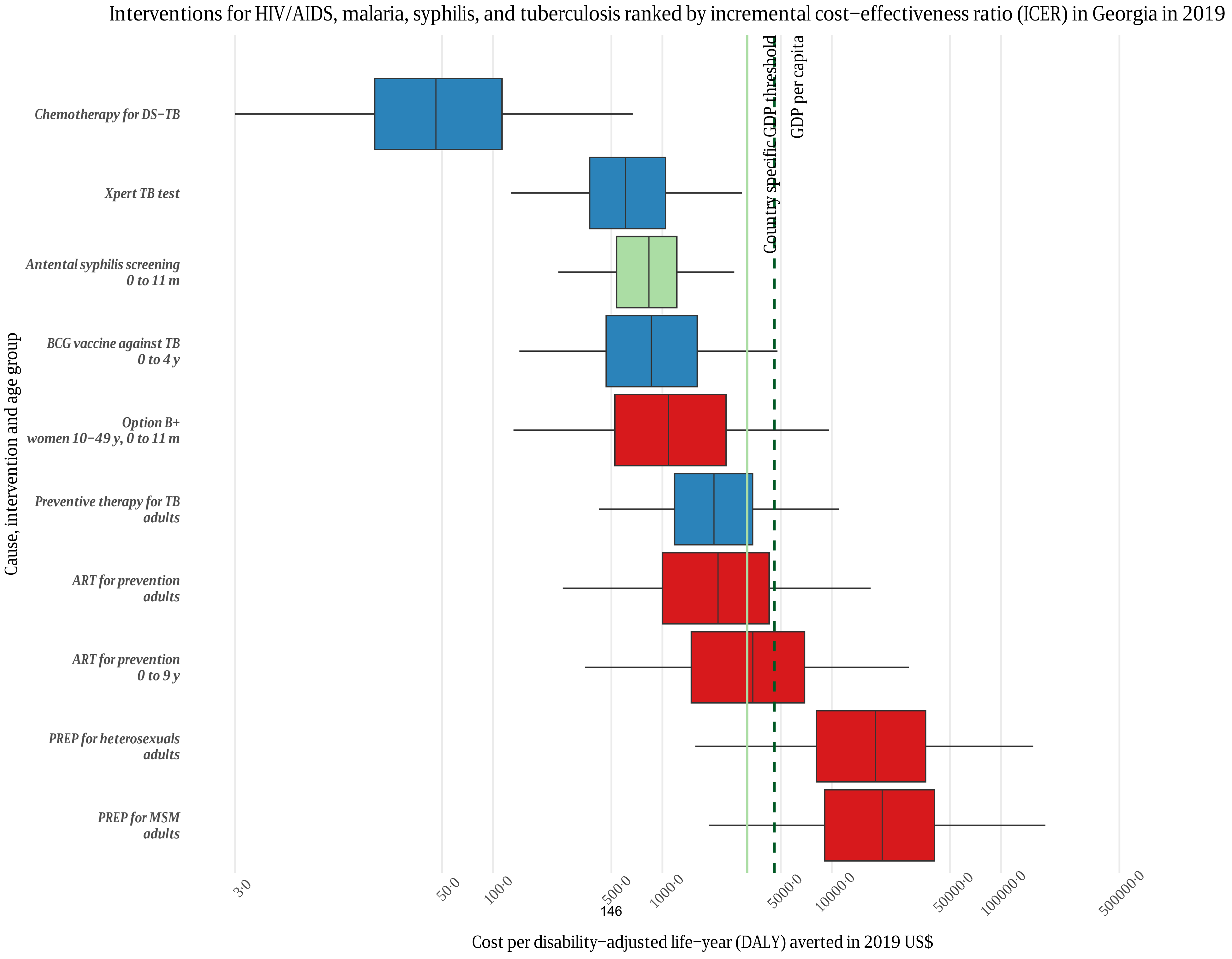

Interventions for HIV/AIDS, malaria, syphilis, and tuberculosis ranked by incremental cost−effectiveness ratio (ICER) in Georgia in 2019
Country specific GDP threshold
GDP per capita
Chemotherapy for DS−TB
Xpert TB test
Antental syphilis screening 0 to 11 m
BCG vaccine against TB 0 to 4 y
Option B+ women 10−49 y, 0 to 11 m
Preventive therapy for TB adults
ART for prevention adults
ART for prevention 0 to 9 y
PREP for heterosexuals adults
PREP for MSM adults
Cause, intervention and age group
3·0
50·0
100·0
500·0
1000·0
5000·0
10000·0
50000·0
100000·0
500000·0
Cost per disability−adjusted life−year (DALY) averted in 2019 US$

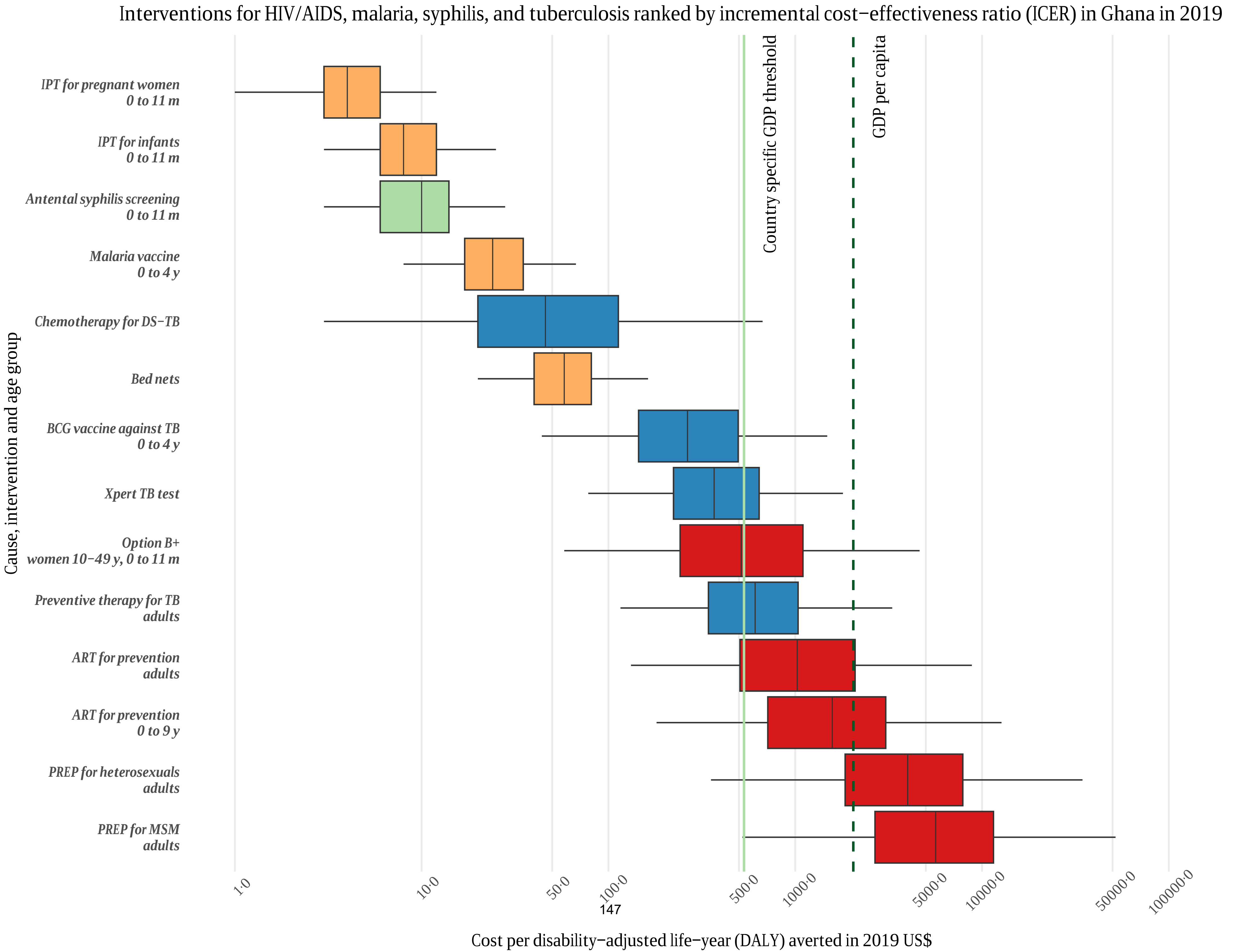
Interventions for HIV/AIDS, malaria, syphilis, and tuberculosis ranked by incremental cost−effectiveness ratio (ICER) in Ghana in 2019
Country specific GDP threshold
GDP per capita
IPT for pregnant women
0 to 11 m
IPT for infants
0 to 11 m
Antental syphilis screening
0 to 11 m
Malaria vaccine
0 to 4 y
Chemotherapy for DS−TB
Bed nets
BCG vaccine against TB
0 to 4 y
Xpert TB test
Option B+
women 10−49 y, 0 to 11 m
Preventive therapy for TB
adults
ART for prevention
adults
ART for prevention
0 to 9 y
PREP for heterosexuals
adults
PREP for MSM
adults
Cause, intervention and age group
1.0
10.0
50.0
100.0
500.0
1000.0
5000.0
10000.0
50000.0
100000.0
Cost per disability−adjusted life−year (DALY) averted in 2019 US$

Interventions for HIV/AIDS, malaria, syphilis, and tuberculosis ranked by incremental cost−effectiveness ratio (ICER) in Grenada in 2019

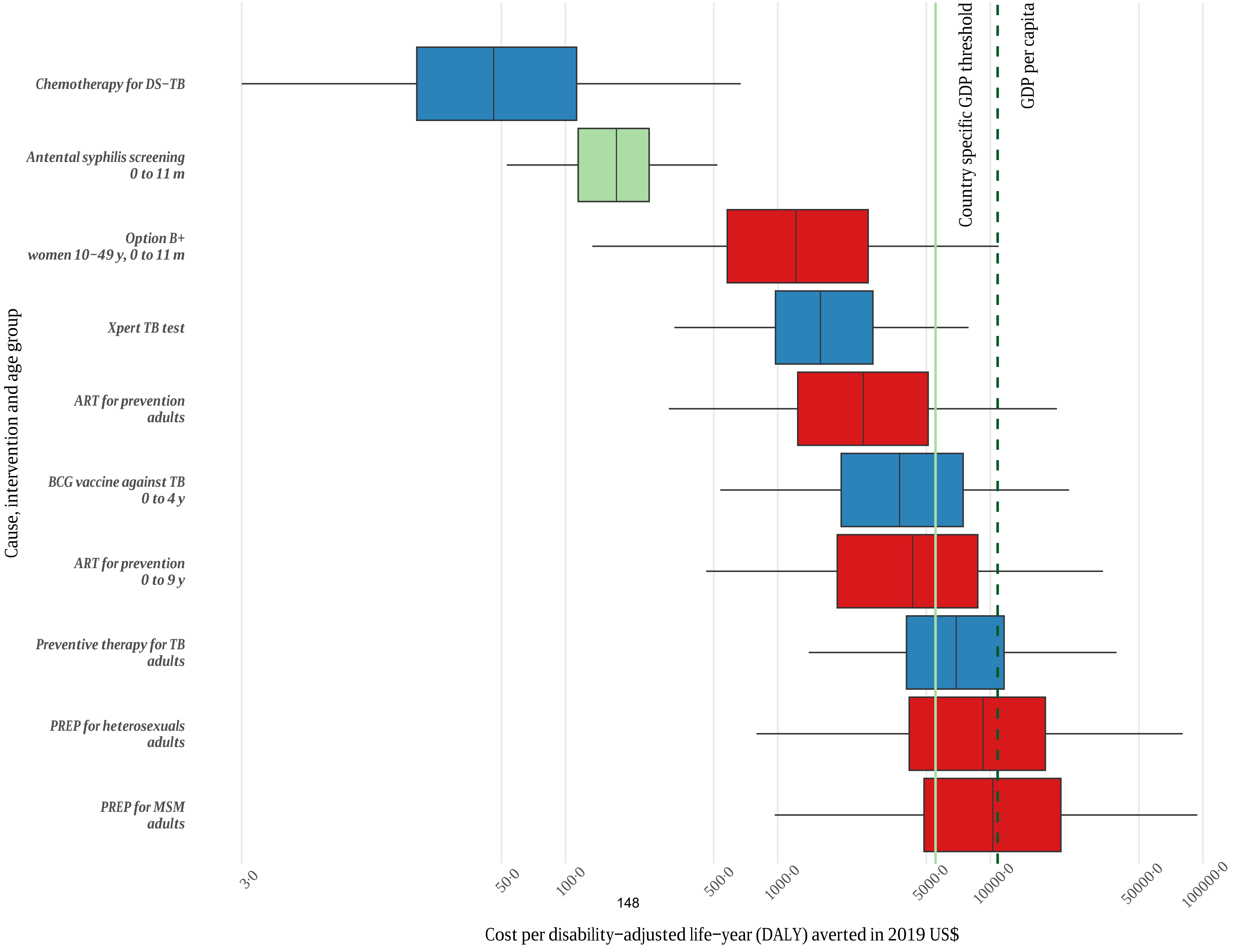

Interventions for HIV/AIDS, malaria, syphilis, and tuberculosis ranked by incremental cost−effectiveness ratio (ICER) in Guatemala in 2019

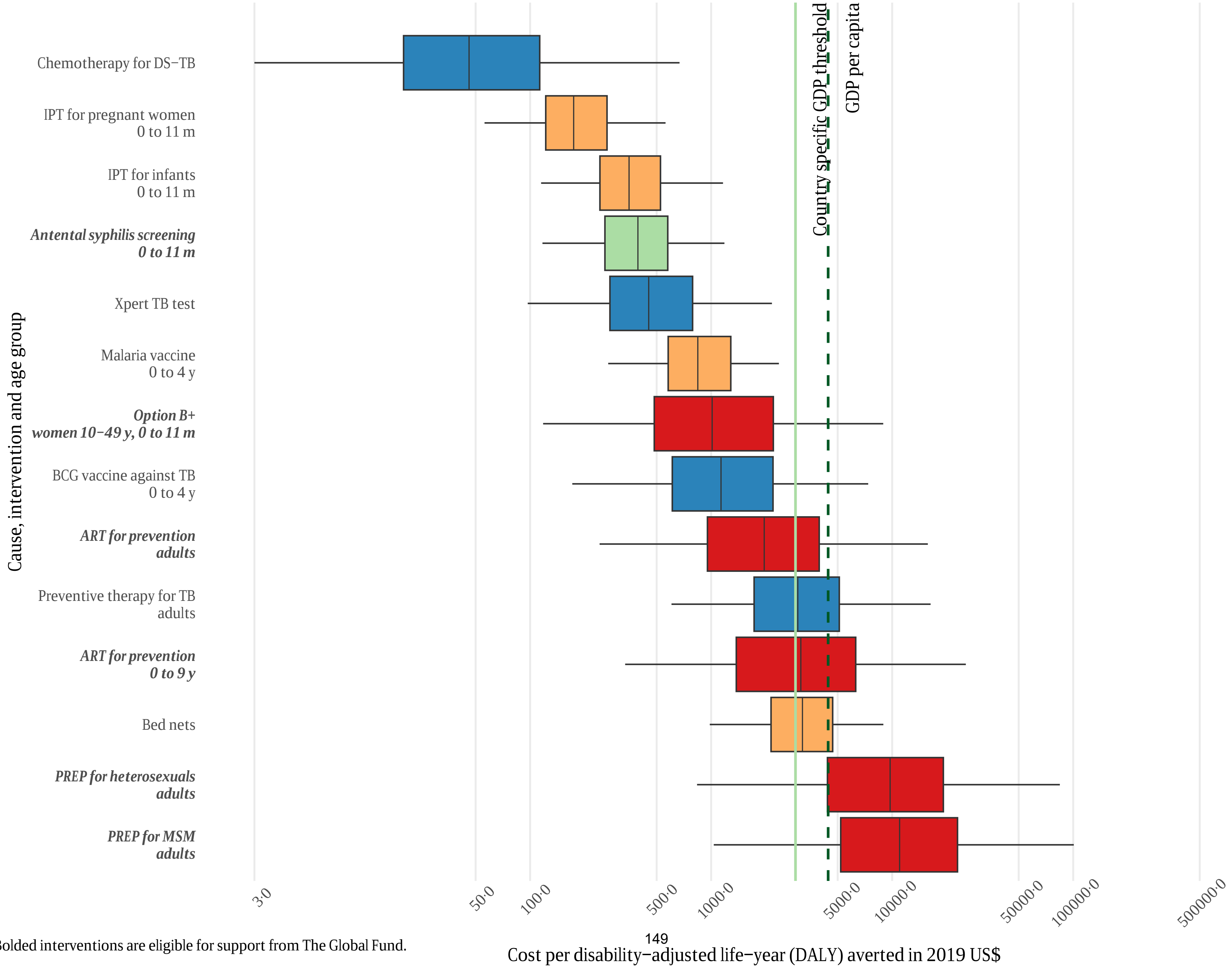

Bolded interventions are eligible for support from The Global Fund.

Interventions for HIV/AIDS, malaria, syphilis, and tuberculosis ranked by incremental cost−effectiveness ratio (ICER) in Guinea in 2019

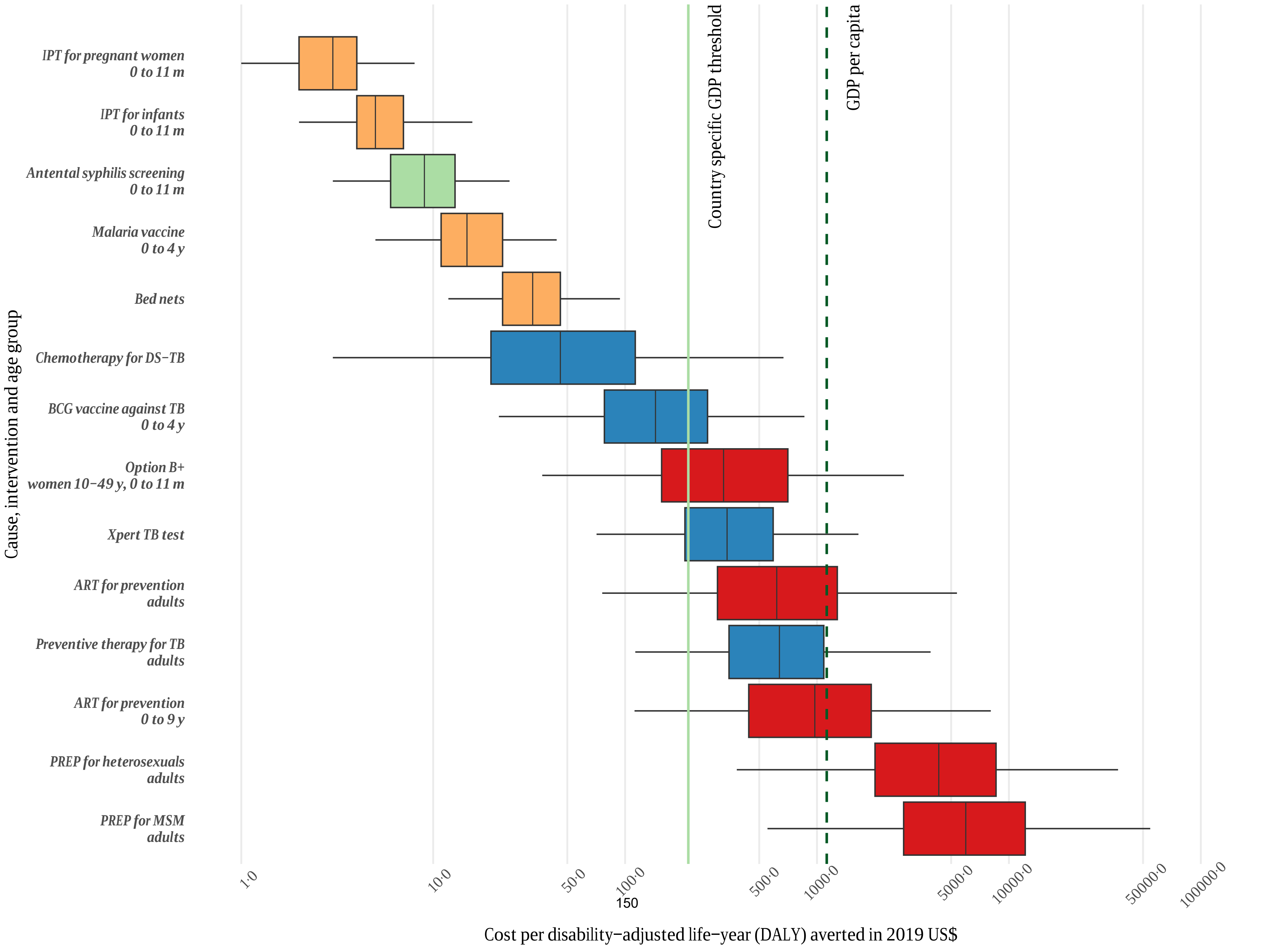

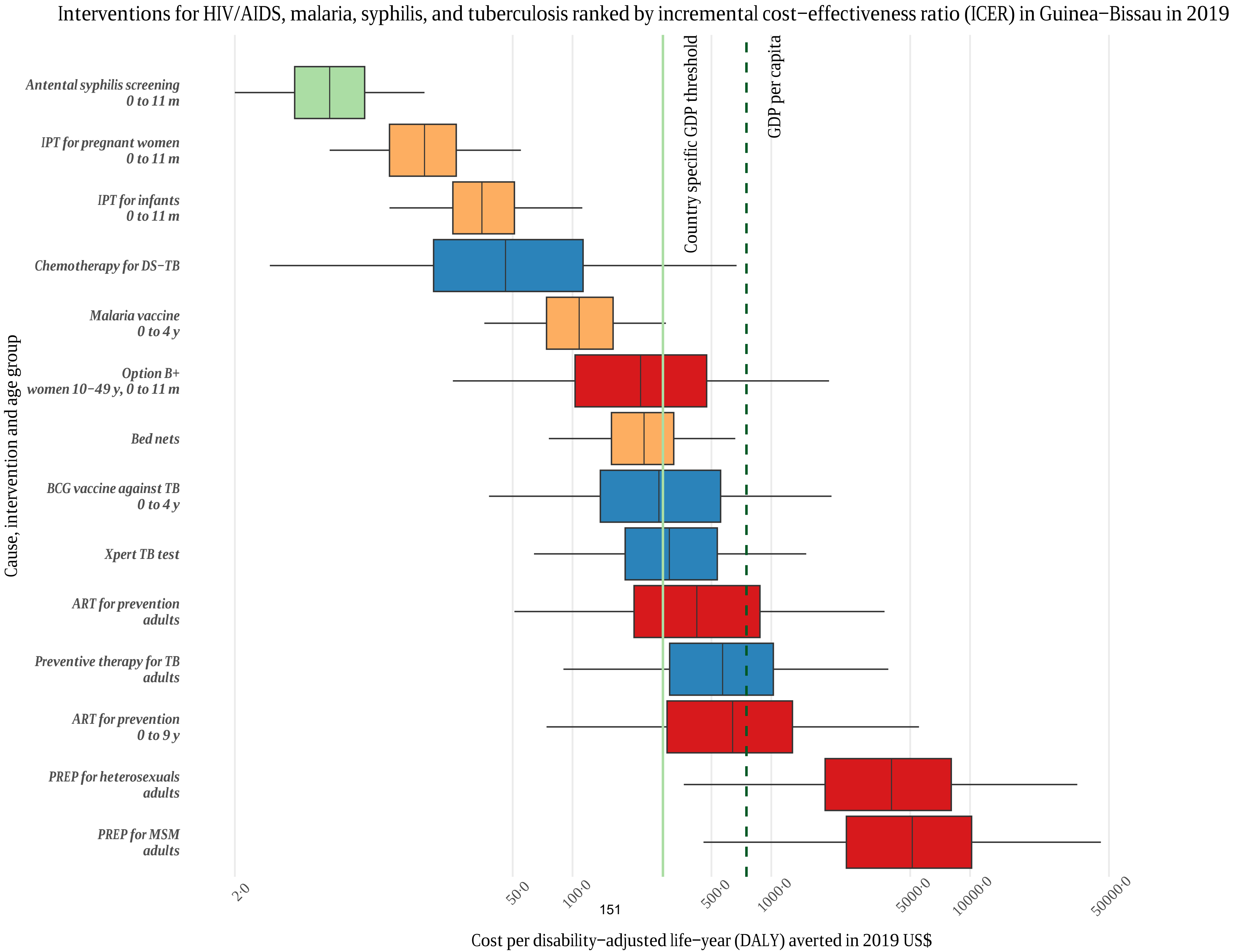
Interventions for HIV/AIDS, malaria, syphilis, and tuberculosis ranked by incremental cost−effectiveness ratio (ICER) in Guinea−Bissau in 2019
Country specific GDP threshold
GDP per capita
Antental syphilis screening
0 to 11 m
IPT for pregnant women
0 to 11 m
IPT for infants
0 to 11 m
Chemotherapy for DS−TB
Malaria vaccine
0 to 4 y
Option B+
women 10−49 y, 0 to 11 m
Bed nets
BCG vaccine against TB
0 to 4 y
Xpert TB test
ART for prevention
adults
Preventive therapy for TB
adults
ART for prevention
0 to 9 y
PREP for heterosexuals
adults
PREP for MSM
adults
Cause, intervention and age group
2·0
50·0
100·0
500·0
1000·0
5000·0
10000·0
50000·0
Cost per disability−adjusted life−year (DALY) averted in 2019 US$

Interventions for HIV/AIDS, malaria, syphilis, and tuberculosis ranked by incremental cost−effectiveness ratio (ICER) in Guyana in 2019

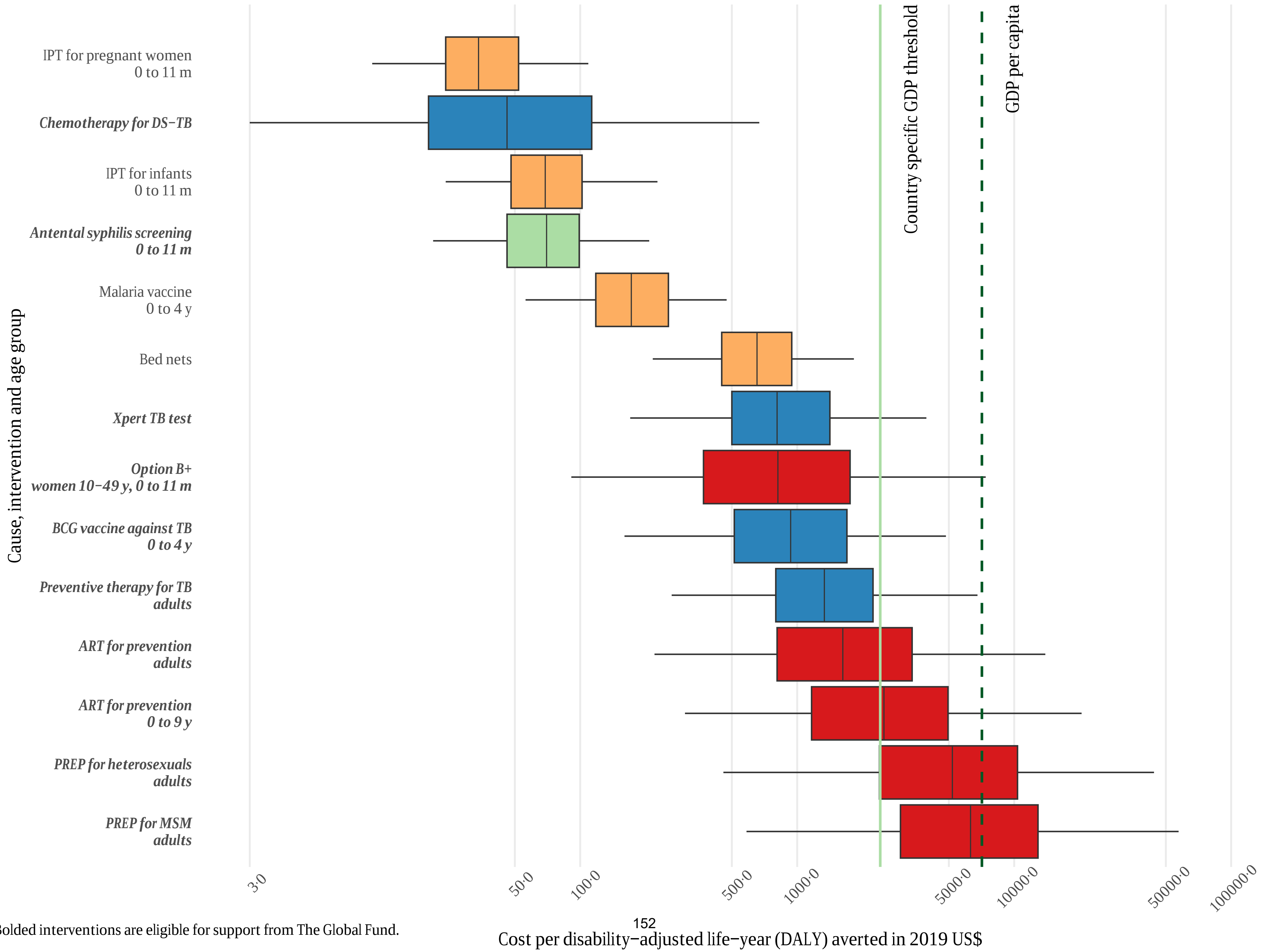

Bolded interventions are eligible for support from The Global Fund.

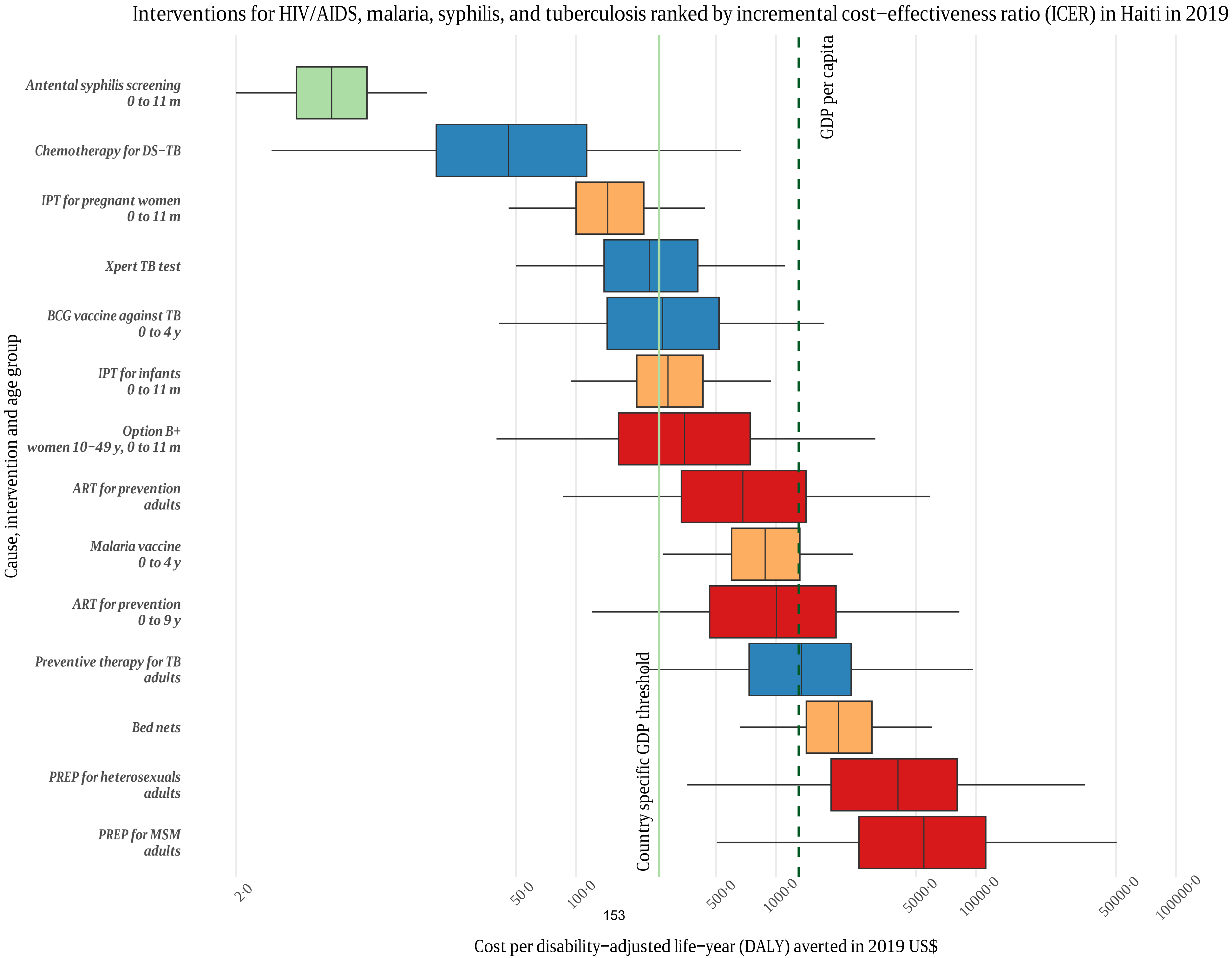

Interventions for HIV/AIDS, malaria, syphilis, and tuberculosis ranked by incremental cost−effectiveness ratio (ICER) in Haiti in 2019
Antental syphilis screening 0 to 11 m
Chemotherapy for DS−TB
IPT for pregnant women 0 to 11 m
Xpert TB test
BCG vaccine against TB 0 to 4 y
IPT for infants 0 to 11 m
Option B+ women 10−49 y, 0 to 11 m
ART for prevention adults
Malaria vaccine 0 to 4 y
ART for prevention 0 to 9 y
Preventive therapy for TB adults
Bed nets
PREP for heterosexuals adults
PREP for MSM adults
Cause, intervention and age group
GDP per capita
Country specific GDP threshold
2·0
50·0
100·0
500·0
1000·0
5000·0
10000·0
50000·0
100000·0
Cost per disability−adjusted life−year (DALY) averted in 2019 US$

Interventions for HIV/AIDS, malaria, syphilis, and tuberculosis ranked by incremental cost−effectiveness ratio (ICER) in Honduras in 2019

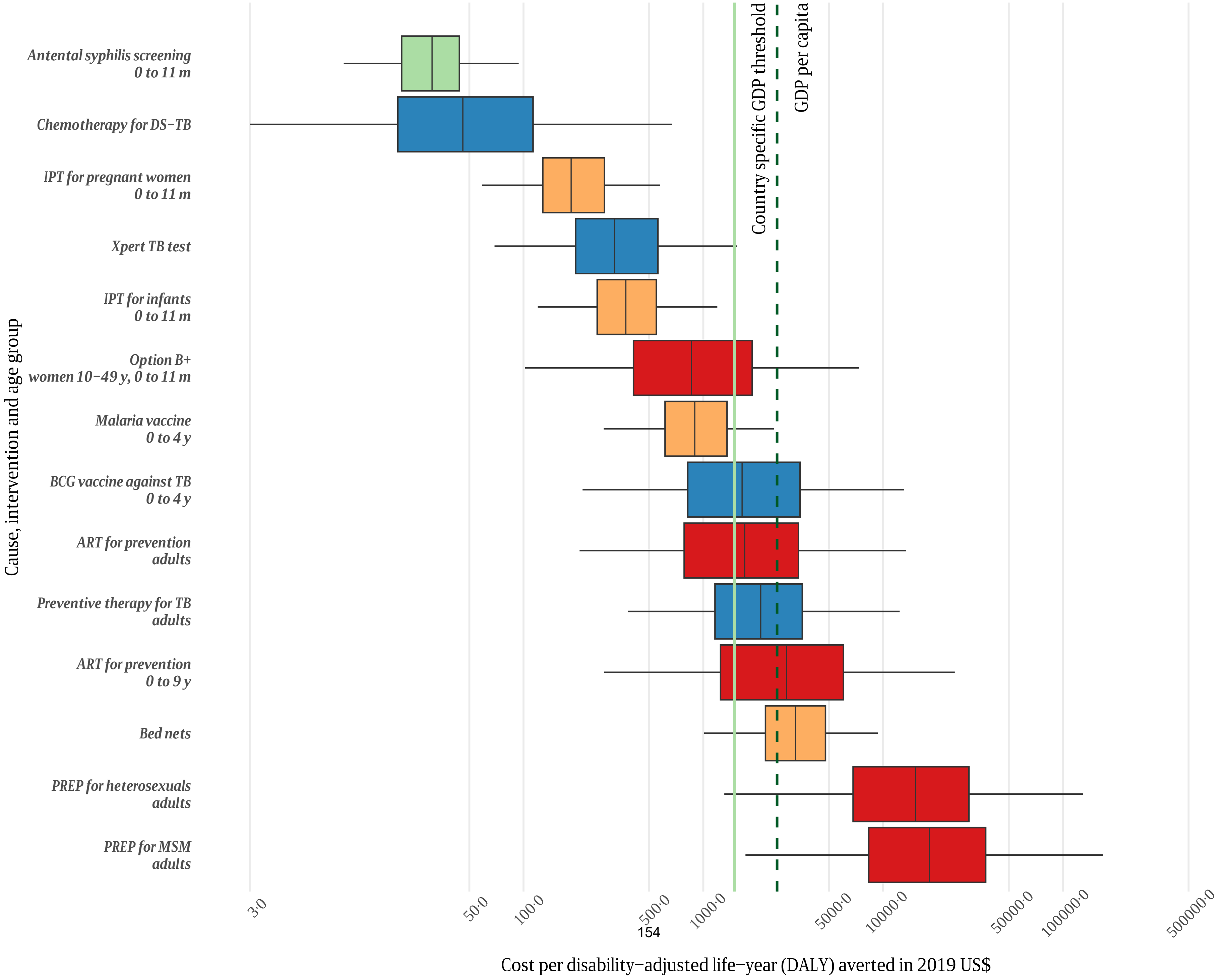

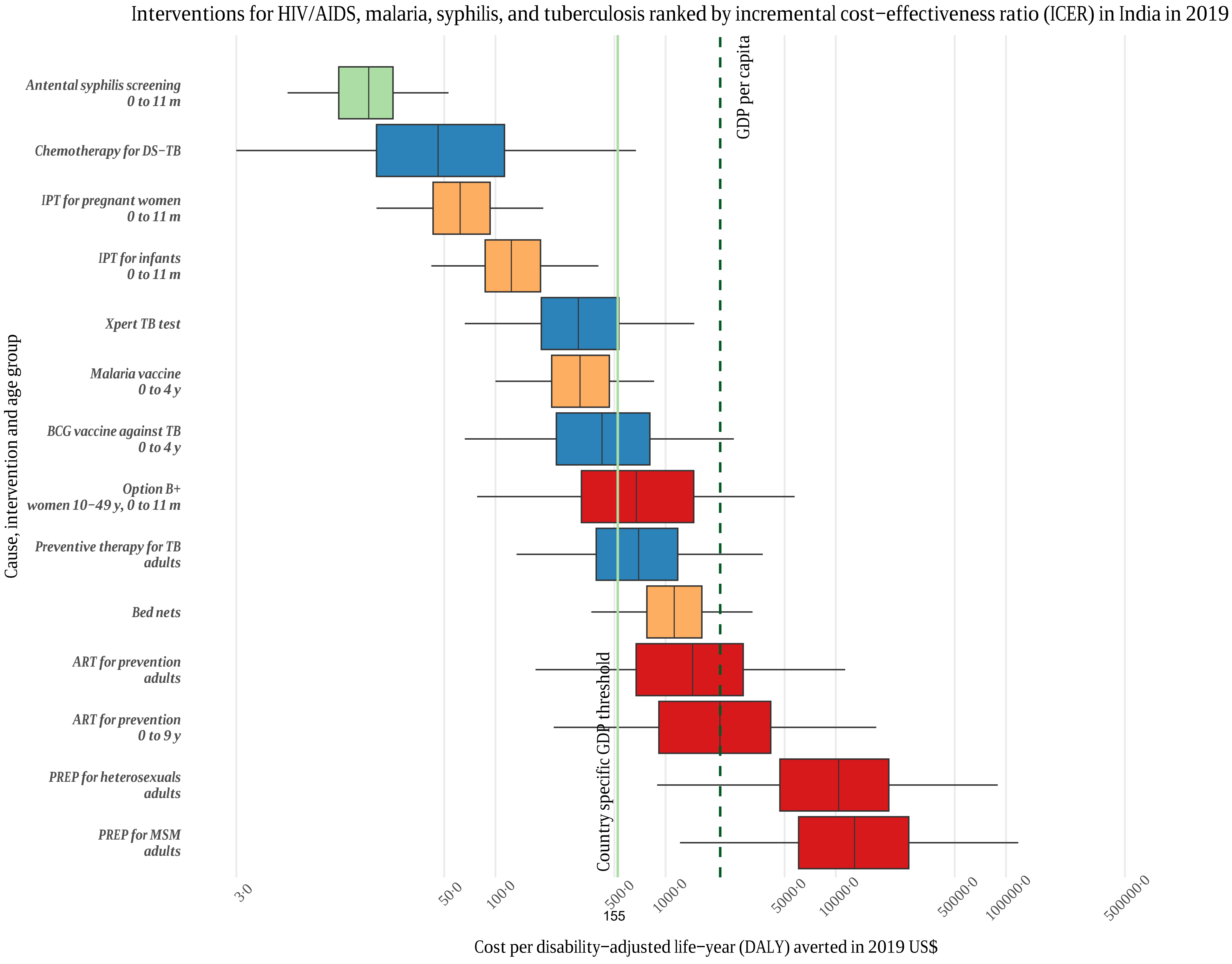

Interventions for HIV/AIDS, malaria, syphilis, and tuberculosis ranked by incremental cost−effectiveness ratio (ICER) in India in 2019
Antental syphilis screening
0 to 11 m
Chemotherapy for DS−TB
IPT for pregnant women
0 to 11 m
IPT for infants
0 to 11 m
Xpert TB test
Malaria vaccine
0 to 4 y
BCG vaccine against TB
0 to 4 y
Option B+
women 10−49 y, 0 to 11 m
Preventive therapy for TB
adults
Bed nets
ART for prevention
adults
ART for prevention
0 to 9 y
PREP for heterosexuals
adults
PREP for MSM
adults
Cause, intervention and age group
GDP per capita
Country specific GDP threshold
3·0
50·0
100·0
500·0
155
1000·0
5000·0
10000·0
50000·0
100000·0
500000·0
Cost per disability−adjusted life−year (DALY) averted in 2019 US$

Interventions for HIV/AIDS, malaria, syphilis, and tuberculosis ranked by incremental cost−effectiveness ratio (ICER) in Indonesia in 2019

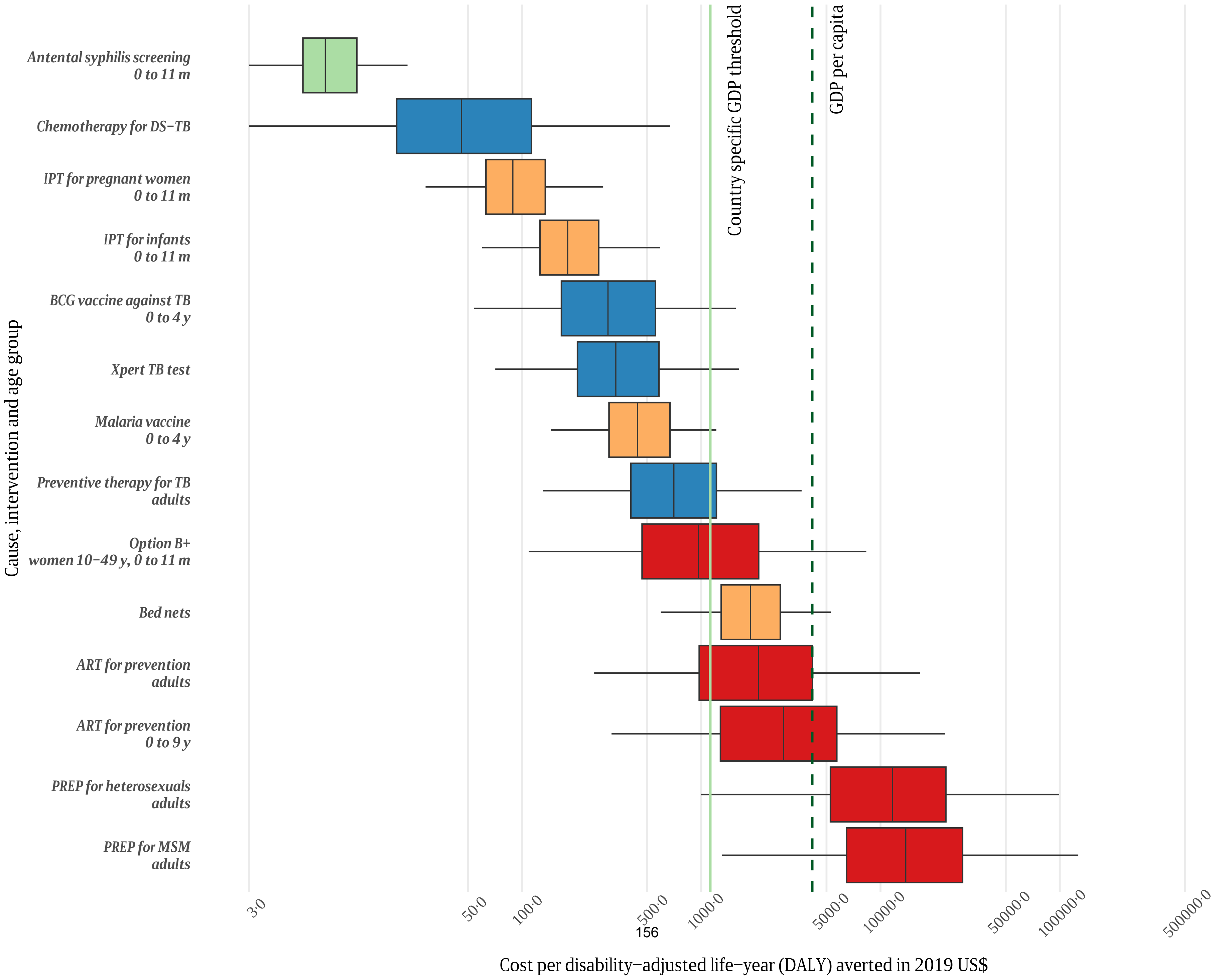

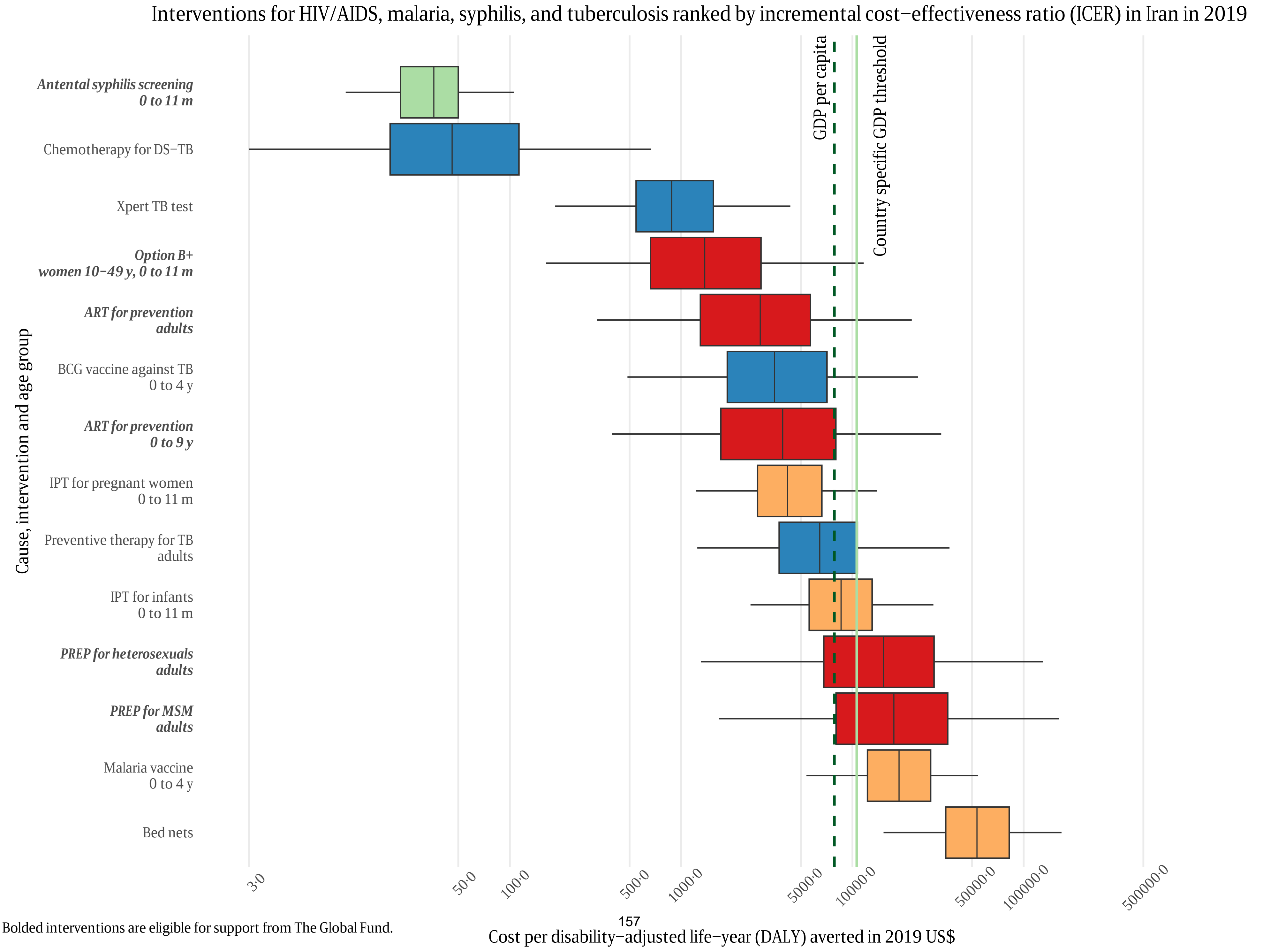

Bolded interventions are eligible for support from The Global Fund.

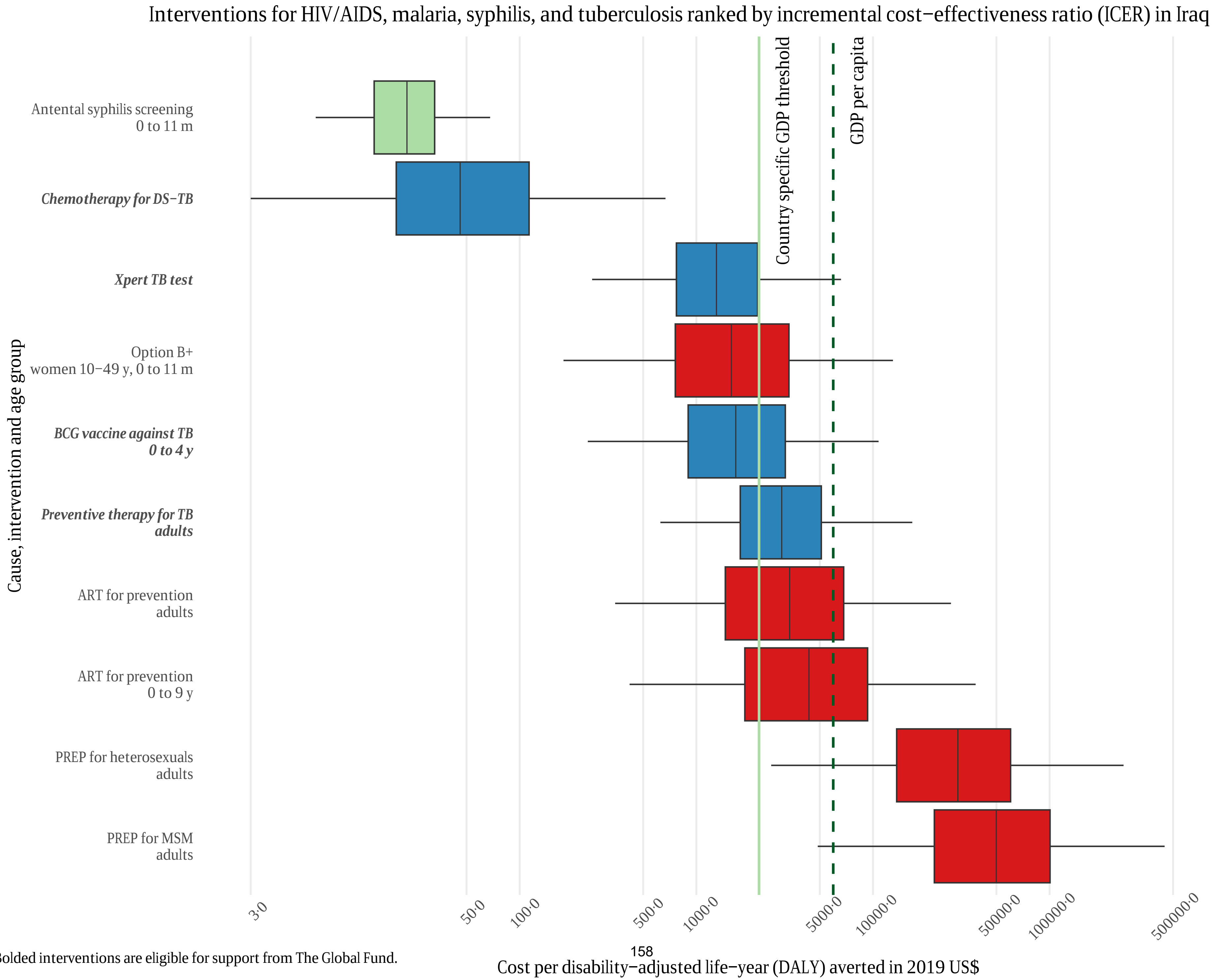

Bolded interventions are eligible for support from The Global Fund.

Interventions for HIV/AIDS, malaria, syphilis, and tuberculosis ranked by incremental cost−effectiveness ratio (ICER) in Jamaica in 2019

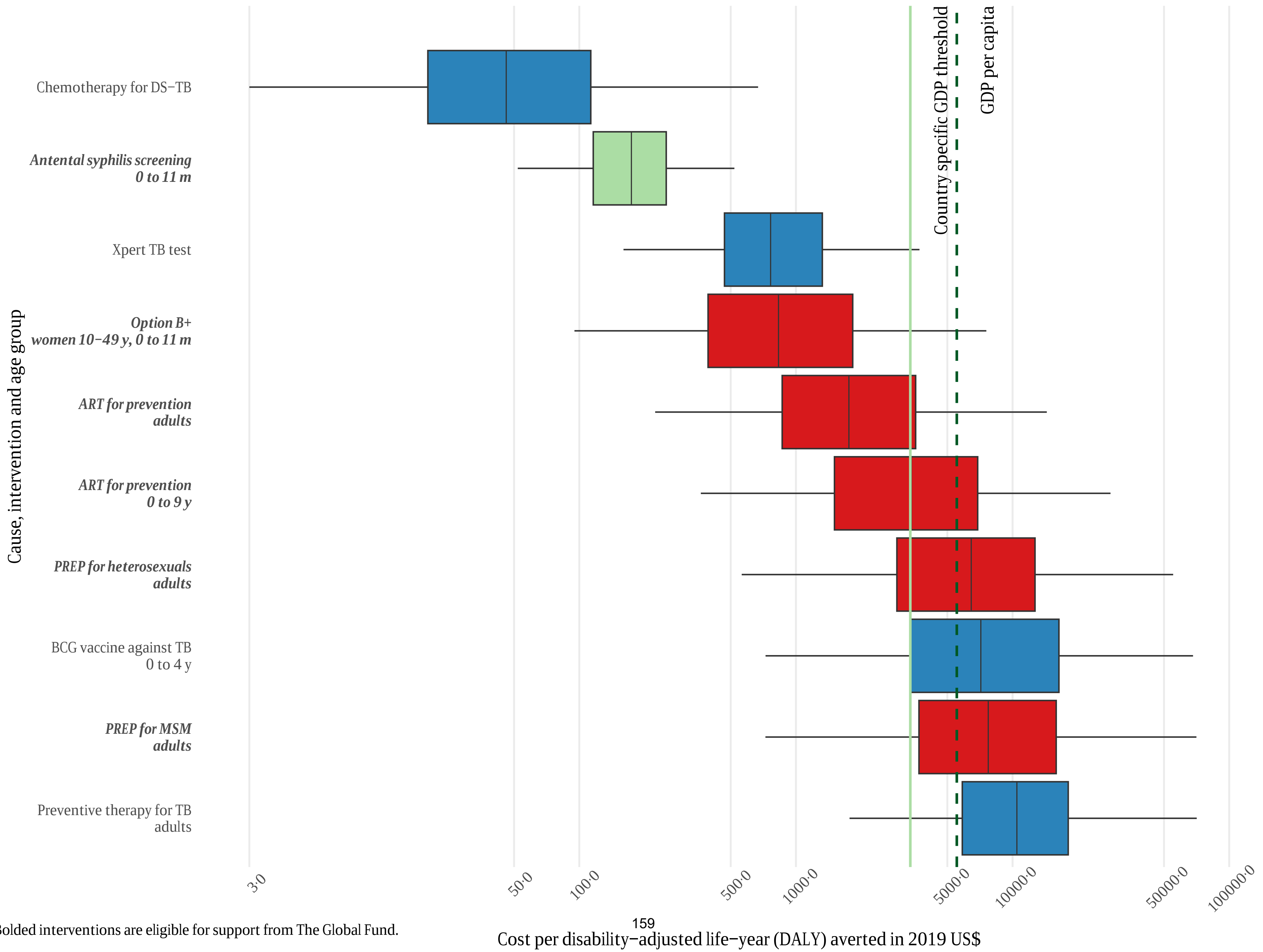

Bolded interventions are eligible for support from The Global Fund.

Interventions for HIV/AIDS, malaria, syphilis, and tuberculosis ranked by incremental cost−effectiveness ratio (ICER) in Jordan in 2019

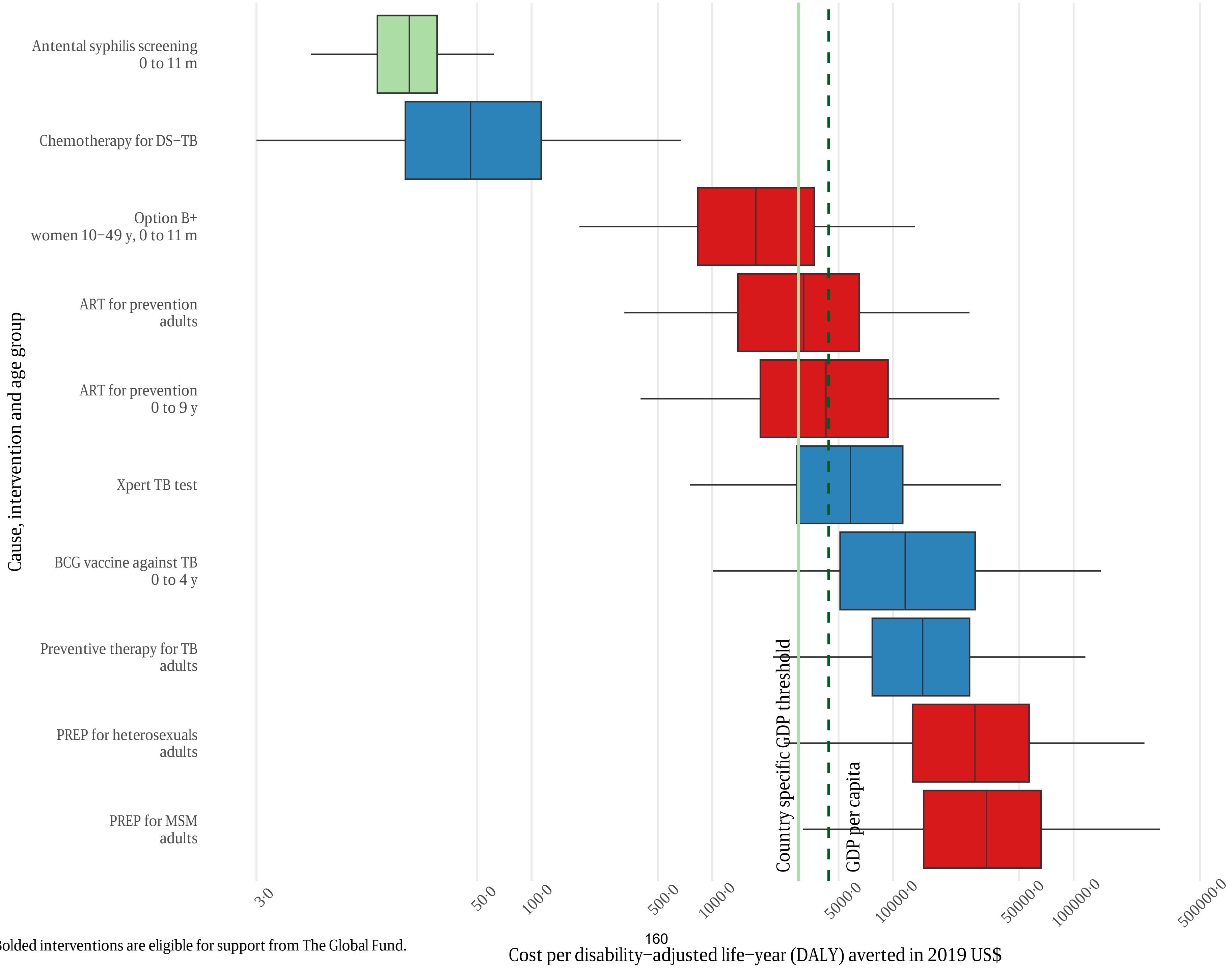

Bolded interventions are eligible for support from The Global Fund.

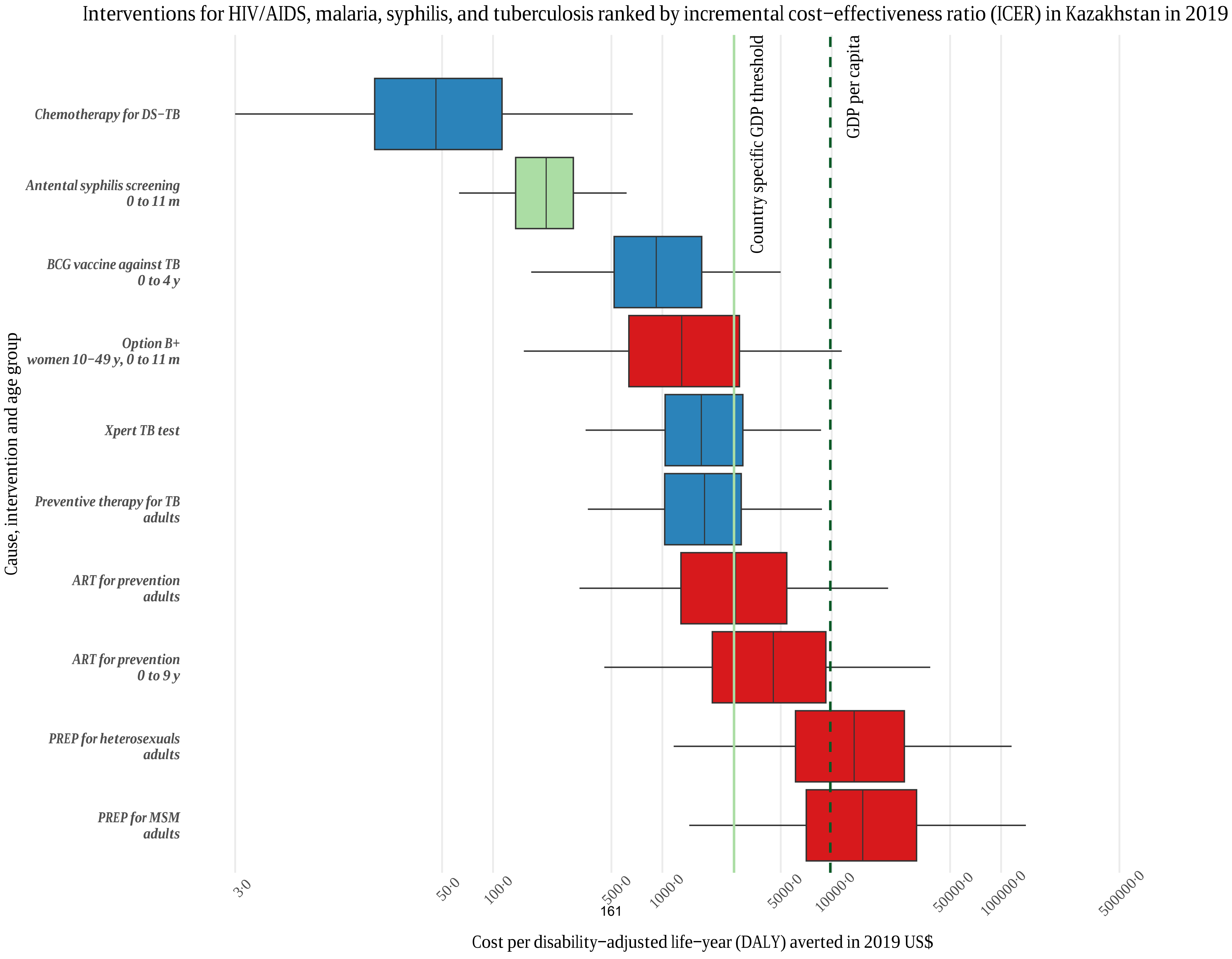

Interventions for HIV/AIDS, malaria, syphilis, and tuberculosis ranked by incremental cost−effectiveness ratio (ICER) in Kazakhstan in 2019
Country specific GDP threshold
GDP per capita
Chemotherapy for DS−TB
Antental syphilis screening
0 to 11 m
BCG vaccine against TB
0 to 4 y
Option B+
women 10−49 y, 0 to 11 m
Xpert TB test
Preventive therapy for TB
adults
ART for prevention
adults
ART for prevention
0 to 9 y
PREP for heterosexuals
adults
PREP for MSM
adults
Cause, intervention and age group
3·0
50·0
100·0
500·0
1000·0
5000·0
10000·0
50000·0
100000·0
500000·0
Cost per disability−adjusted life−year (DALY) averted in 2019 US$

Interventions for HIV/AIDS, malaria, syphilis, and tuberculosis ranked by incremental cost−effectiveness ratio (ICER) in Kenya in 2019

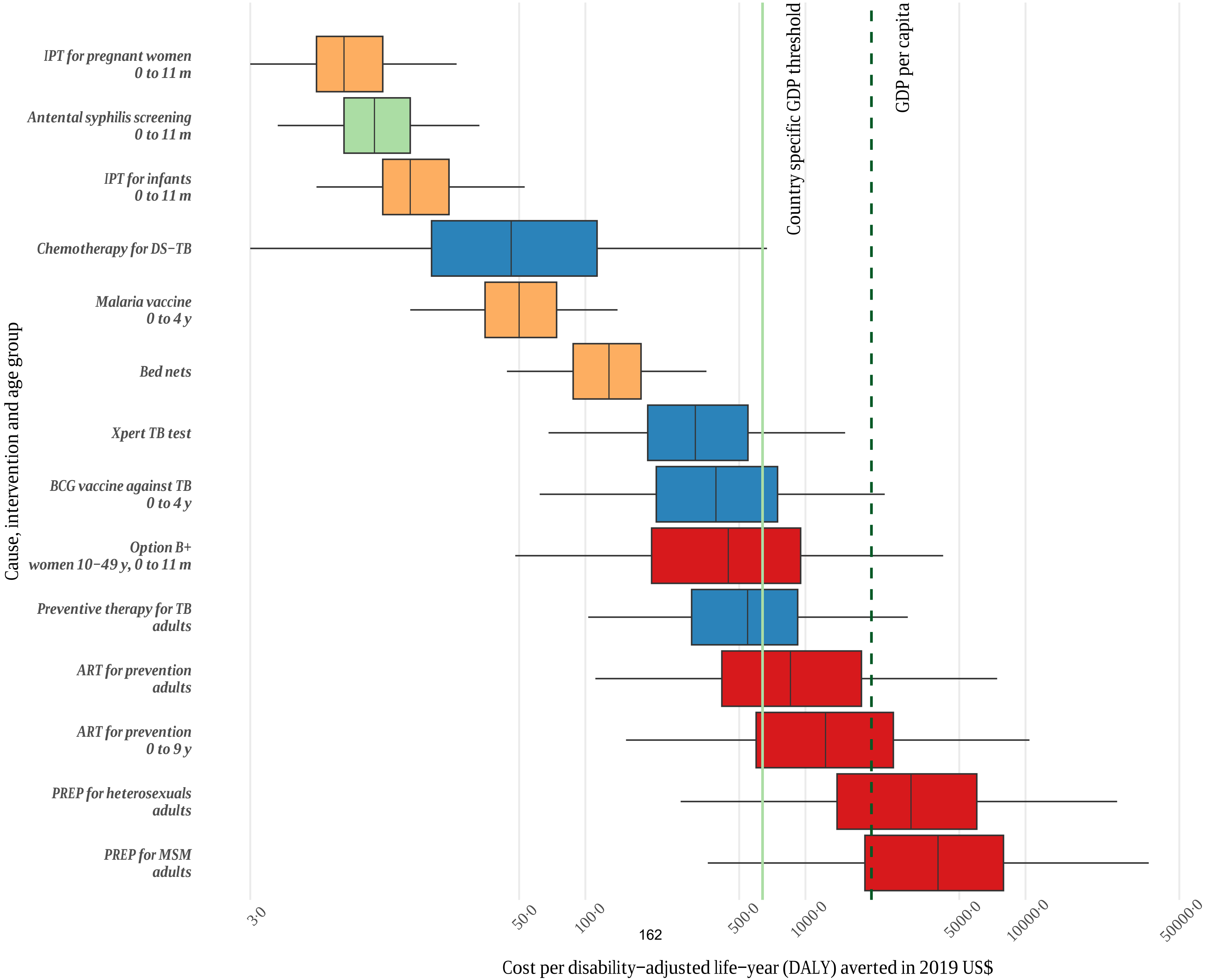

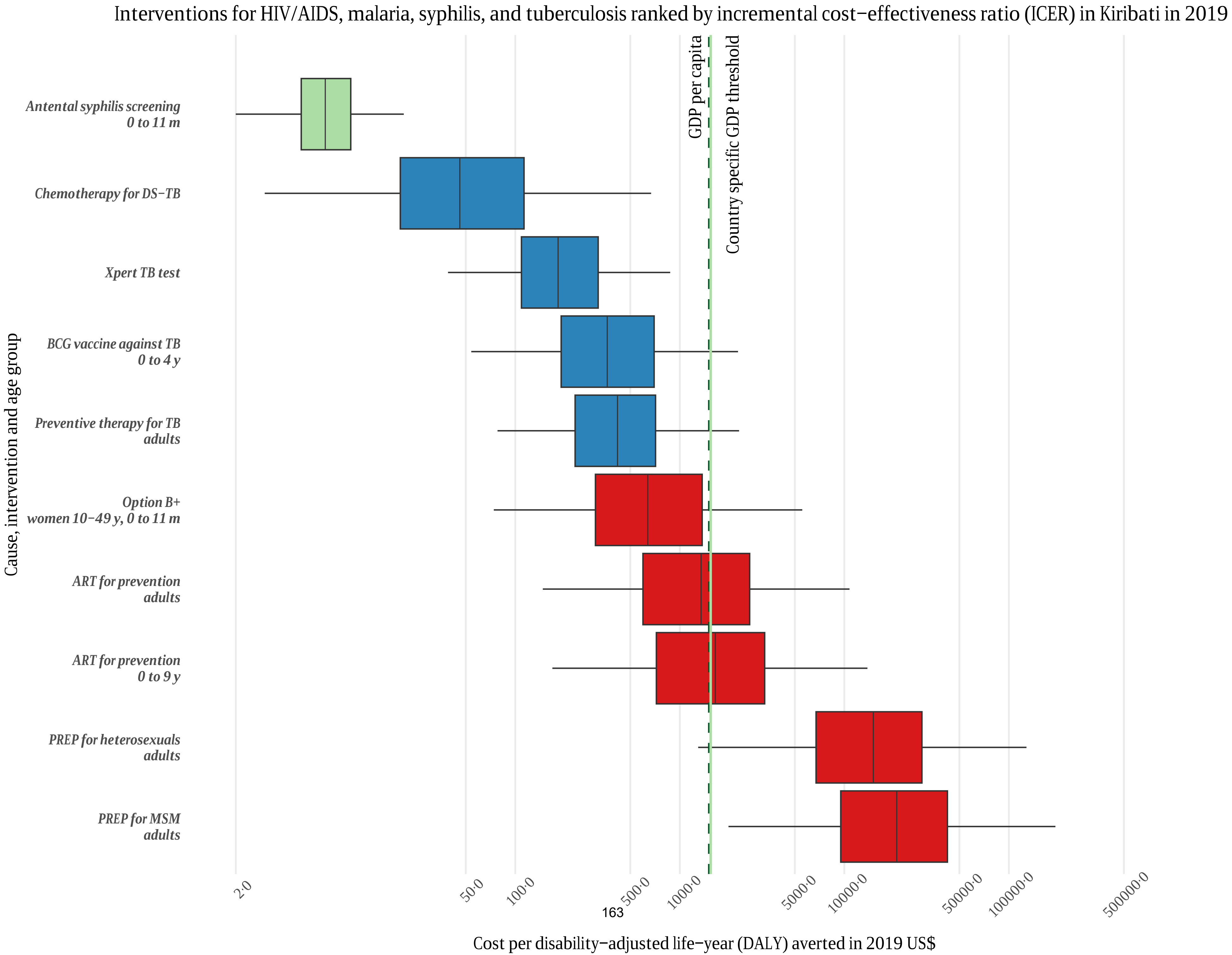
Interventions for HIV/AIDS, malaria, syphilis, and tuberculosis ranked by incremental cost−effectiveness ratio (ICER) in Kiribati in 2019
GDP per capita
Country specific GDP threshold
Cause, intervention and age group
Antental syphilis screening 0 to 11 m
Chemotherapy for DS−TB
Xpert TB test
BCG vaccine against TB 0 to 4 y
Preventive therapy for TB adults
Option B+ women 10−49 y, 0 to 11 m
ART for prevention adults
ART for prevention 0 to 9 y
PREP for heterosexuals adults
PREP for MSM adults
2·0
50·0
100·0
500·0
1000·0
5000·0
10000·0
50000·0
100000·0
500000·0
Cost per disability−adjusted life−year (DALY) averted in 2019 US$

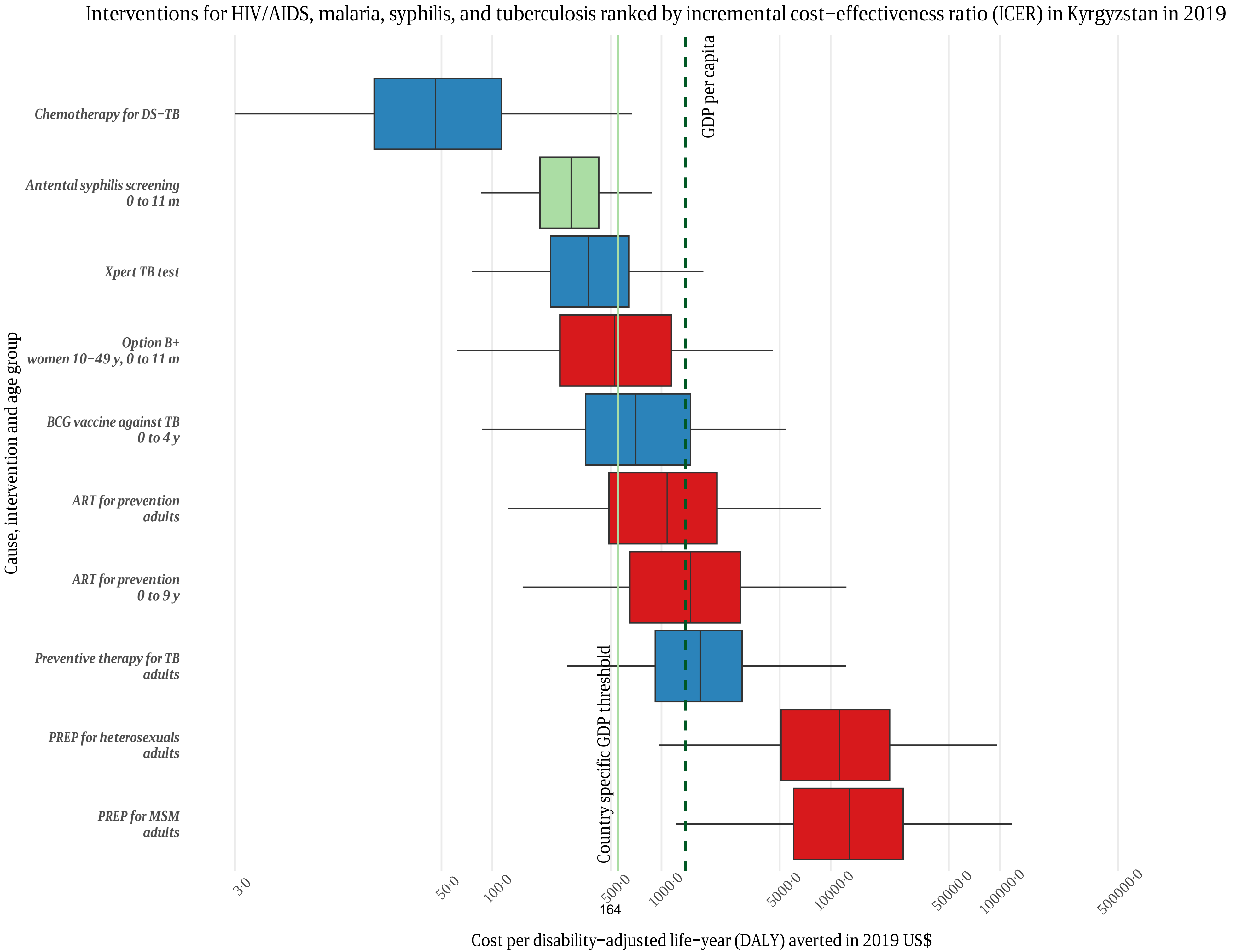

Interventions for HIV/AIDS, malaria, syphilis, and tuberculosis ranked by incremental cost−effectiveness ratio (ICER) in Kyrgyzstan in 2019
Cause, intervention and age group
Chemotherapy for DS−TB
Antental syphilis screening
0 to 11 m
Xpert TB test
Option B+
women 10−49 y, 0 to 11 m
BCG vaccine against TB
0 to 4 y
ART for prevention
adults
ART for prevention
0 to 9 y
Preventive therapy for TB
adults
PREP for heterosexuals
adults
PREP for MSM
adults
GDP per capita
Country specific GDP threshold
3·0
50·0
100·0
500·0
164
1000·0
5000·0
10000·0
50000·0
100000·0
500000·0
Cost per disability−adjusted life−year (DALY) averted in 2019 US$

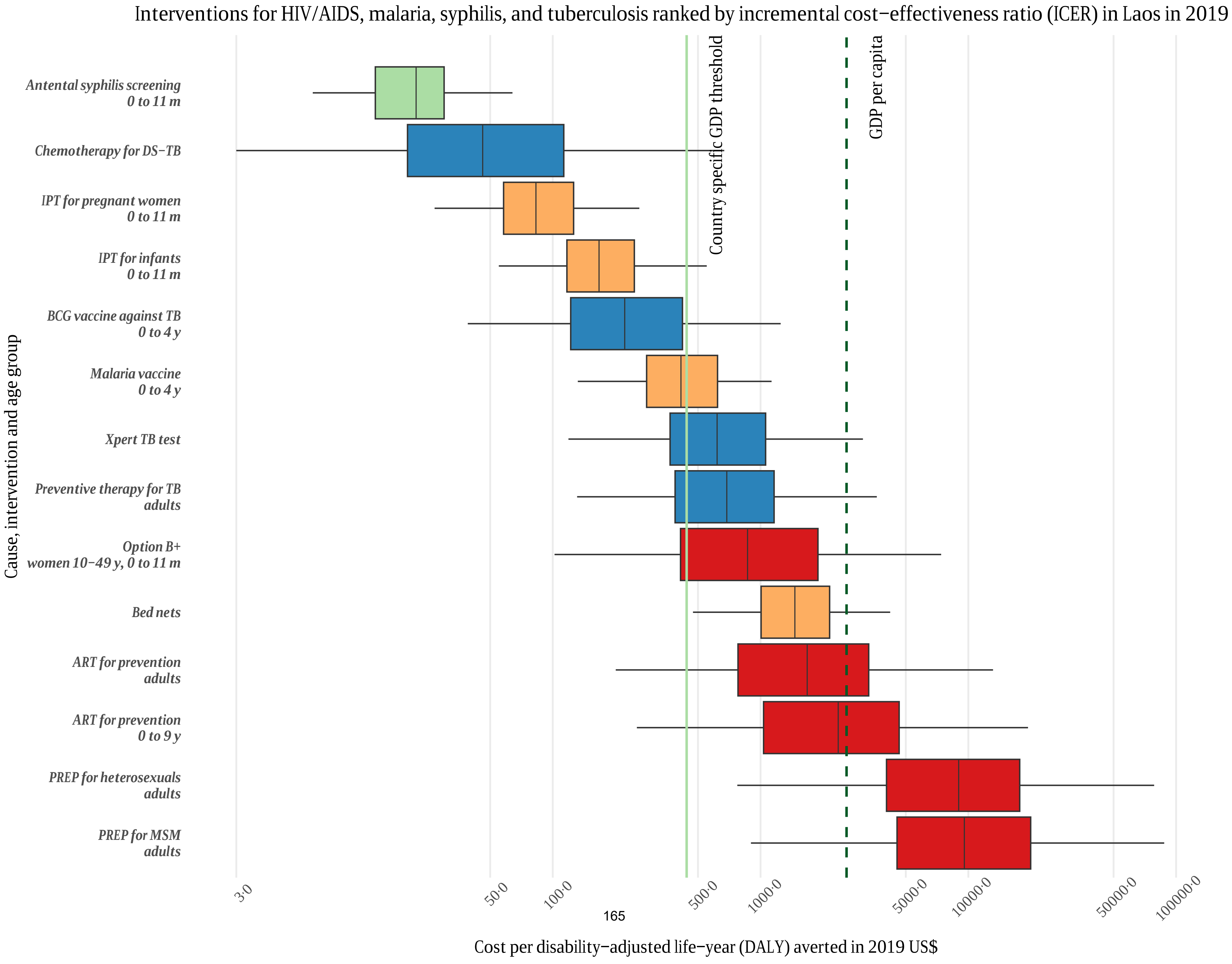
Interventions for HIV/AIDS, malaria, syphilis, and tuberculosis ranked by incremental cost−effectiveness ratio (ICER) in Laos in 2019
Country specific GDP threshold
GDP per capita
Antental syphilis screening
0 to 11 m
Chemotherapy for DS−TB
IPT for pregnant women
0 to 11 m
IPT for infants
0 to 11 m
BCG vaccine against TB
0 to 4 y
Malaria vaccine
0 to 4 y
Xpert TB test
Preventive therapy for TB
adults
Option B+
women 10−49 y, 0 to 11 m
Bed nets
ART for prevention
adults
ART for prevention
0 to 9 y
PREP for heterosexuals
adults
PREP for MSM
adults
Cause, intervention and age group
3·0
50·0
100·0
500·0
1000·0
5000·0
10000·0
50000·0
100000·0
Cost per disability−adjusted life−year (DALY) averted in 2019 US$

Interventions for HIV/AIDS, malaria, syphilis, and tuberculosis ranked by incremental cost−effectiveness ratio (ICER) in Lebanon in 2019

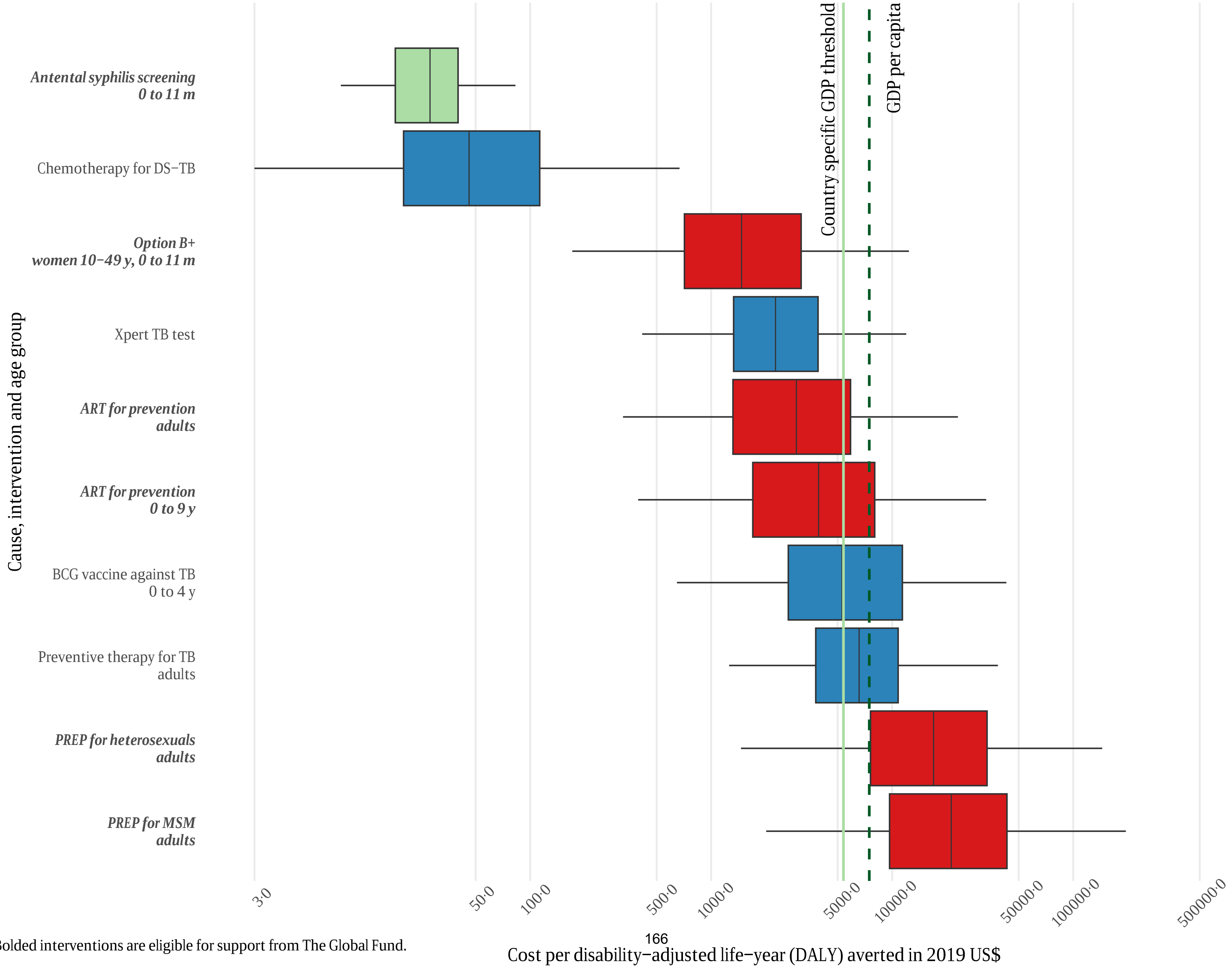

Bolded interventions are eligible for support from The Global Fund.

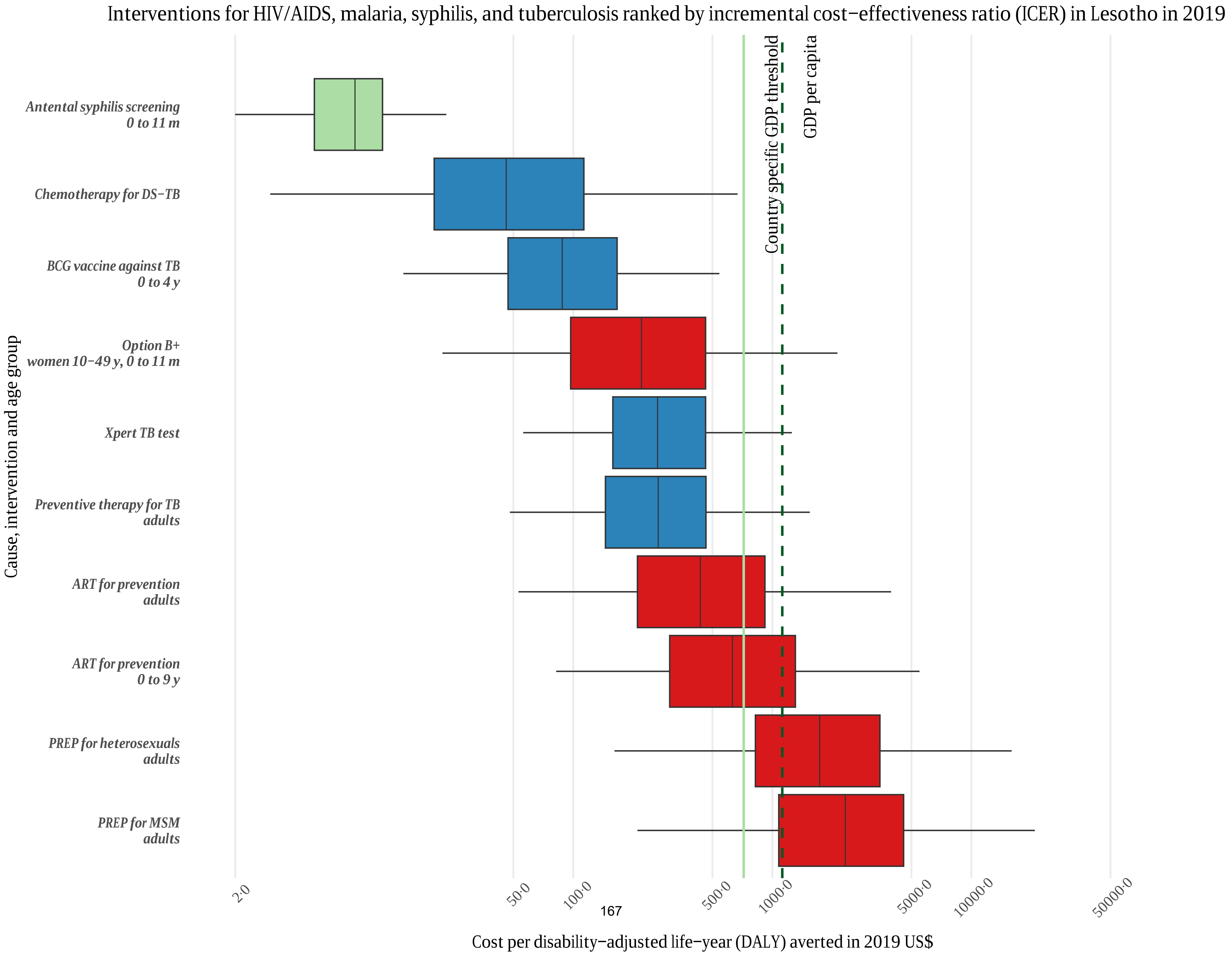

Interventions for HIV/AIDS, malaria, syphilis, and tuberculosis ranked by incremental cost−effectiveness ratio (ICER) in Lesotho in 2019
Country specific GDP threshold
GDP per capita
Cause, intervention and age group
Antental syphilis screening
0 to 11 m
Chemotherapy for DS−TB
BCG vaccine against TB
0 to 4 y
Option B+
women 10−49 y, 0 to 11 m
Xpert TB test
Preventive therapy for TB
adults
ART for prevention
adults
ART for prevention
0 to 9 y
PREP for heterosexuals
adults
PREP for MSM
adults
2·0
50·0
100·0
500·0
1000·0
5000·0
10000·0
50000·0
Cost per disability−adjusted life−year (DALY) averted in 2019 US$

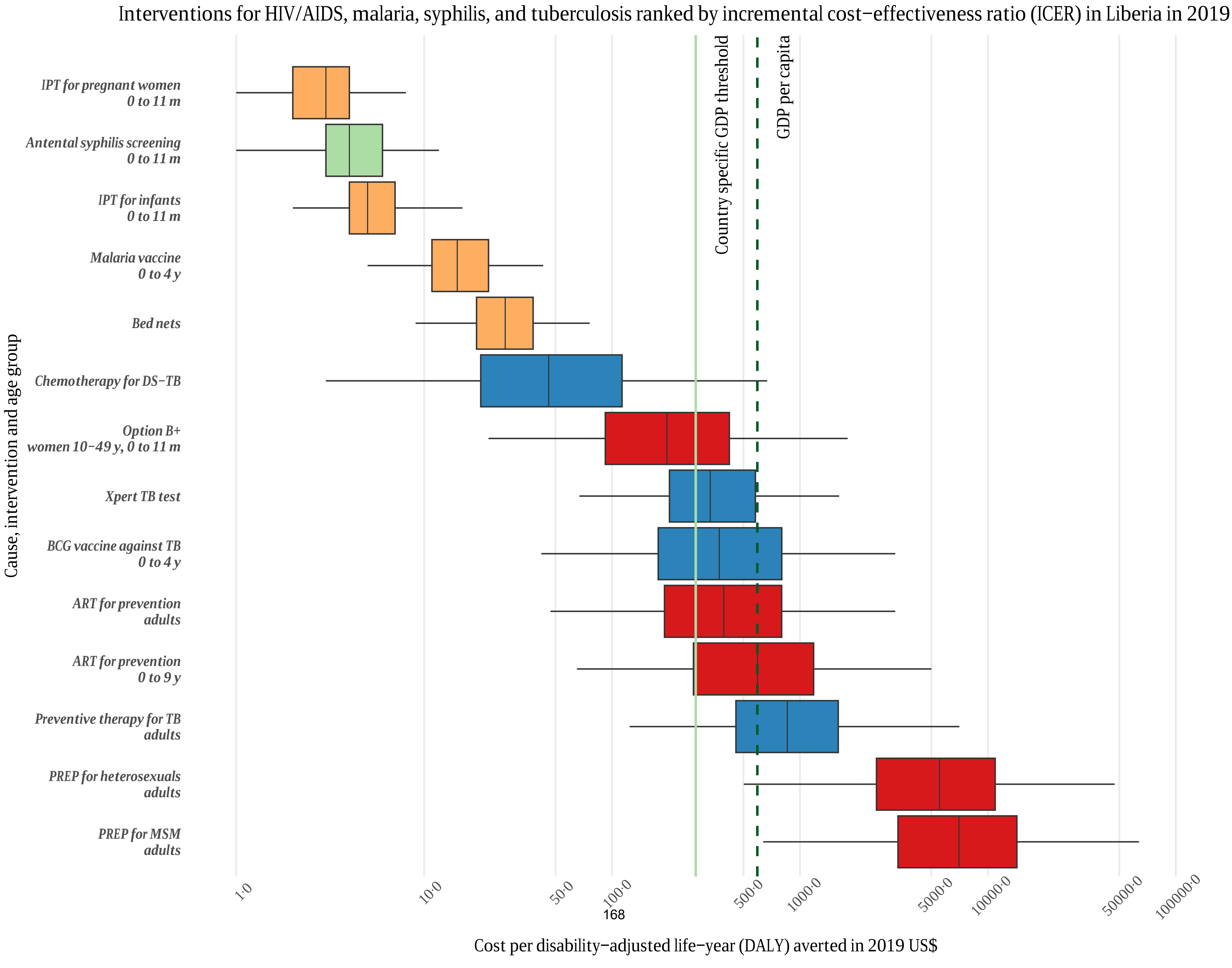
Interventions for HIV/AIDS, malaria, syphilis, and tuberculosis ranked by incremental cost−effectiveness ratio (ICER) in Liberia in 2019
Country specific GDP threshold
GDP per capita
IPT for pregnant women
0 to 11 m
Antental syphilis screening
0 to 11 m
IPT for infants
0 to 11 m
Malaria vaccine
0 to 4 y
Bed nets
Chemotherapy for DS−TB
Option B+
women 10−49 y, 0 to 11 m
Xpert TB test
BCG vaccine against TB
0 to 4 y
ART for prevention
adults
ART for prevention
0 to 9 y
Preventive therapy for TB
adults
PREP for heterosexuals
adults
PREP for MSM
adults
Cause, intervention and age group
1·0
10·0
50·0
100·0
500·0
1000·0
5000·0
10000·0
50000·0
100000·0
Cost per disability−adjusted life−year (DALY) averted in 2019 US$

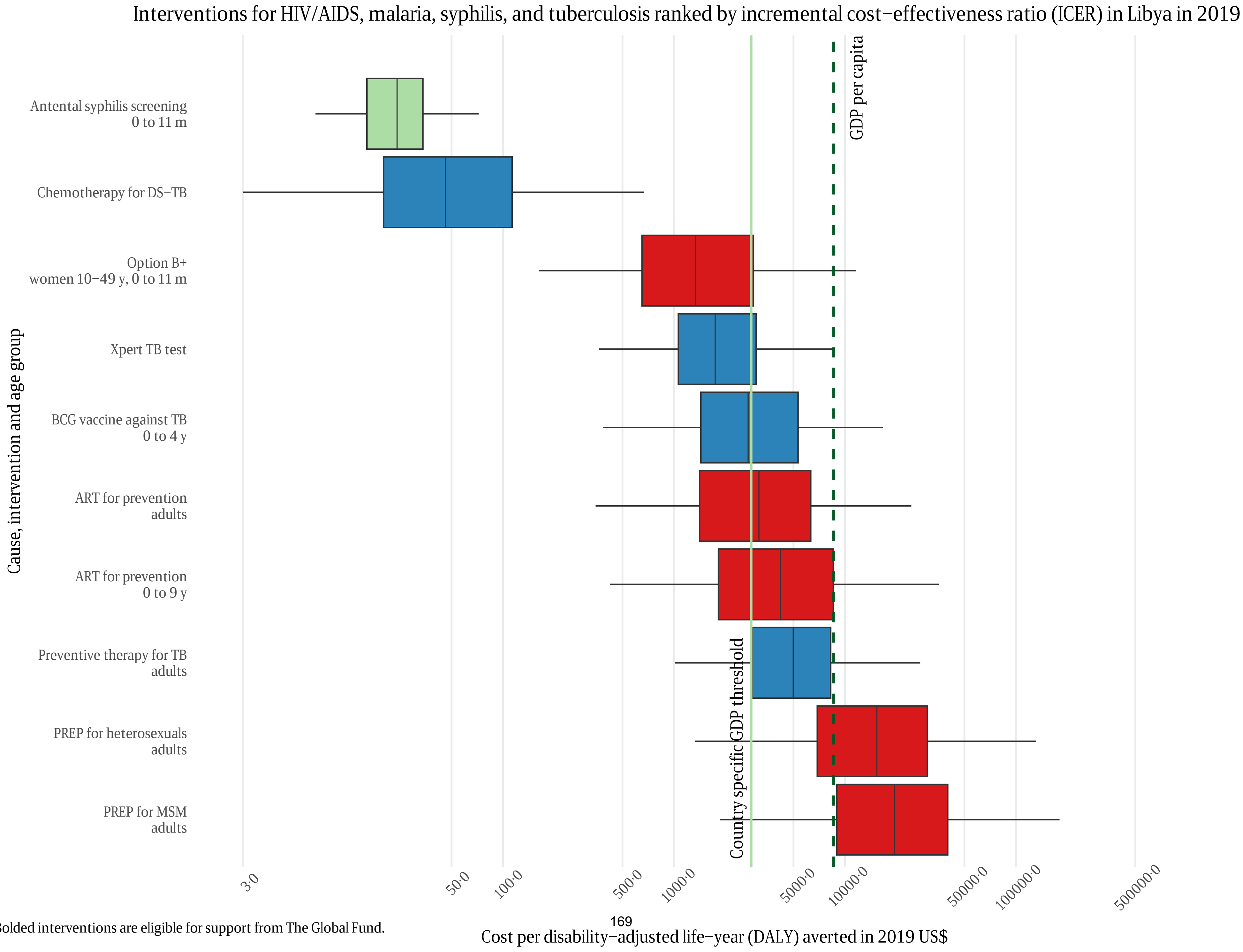

Bolded interventions are eligible for support from The Global Fund.

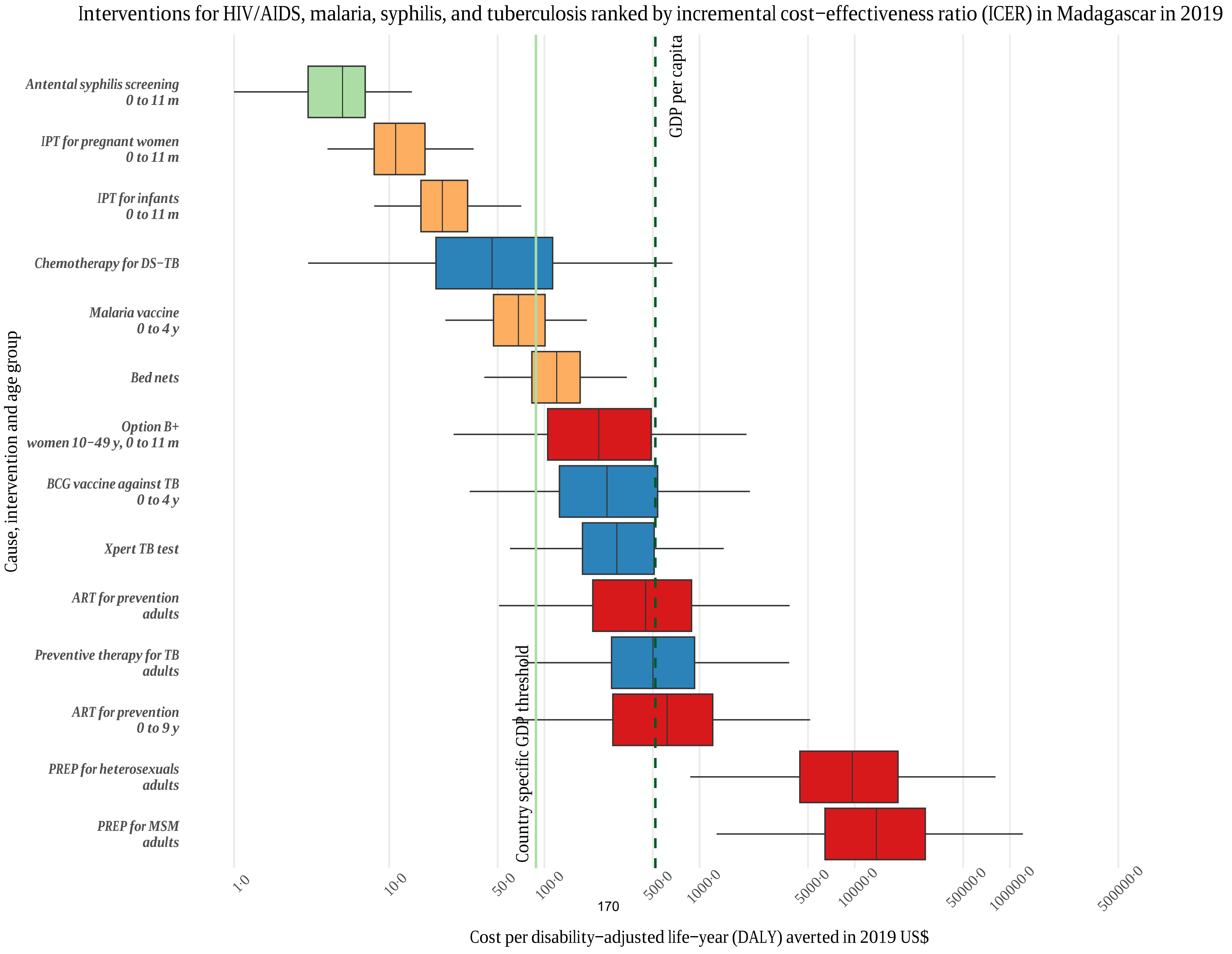
Interventions for HIV/AIDS, malaria, syphilis, and tuberculosis ranked by incremental cost−effectiveness ratio (ICER) in Madagascar in 2019
Antental syphilis screening 0 to 11 m
IPT for pregnant women 0 to 11 m
IPT for infants 0 to 11 m
Chemotherapy for DS−TB
Malaria vaccine 0 to 4 y
Bed nets
Option B+ women 10−49 y, 0 to 11 m
BCG vaccine against TB 0 to 4 y
Xpert TB test
ART for prevention adults
Preventive therapy for TB adults
ART for prevention 0 to 9 y
PREP for heterosexuals adults
PREP for MSM adults
Cause, intervention and age group
GDP per capita
Country specific GDP threshold
1·0
10·0
50·0
100·0
500·0
1000·0
5000·0
10000·0
50000·0
100000·0
500000·0
Cost per disability−adjusted life−year (DALY) averted in 2019 US$

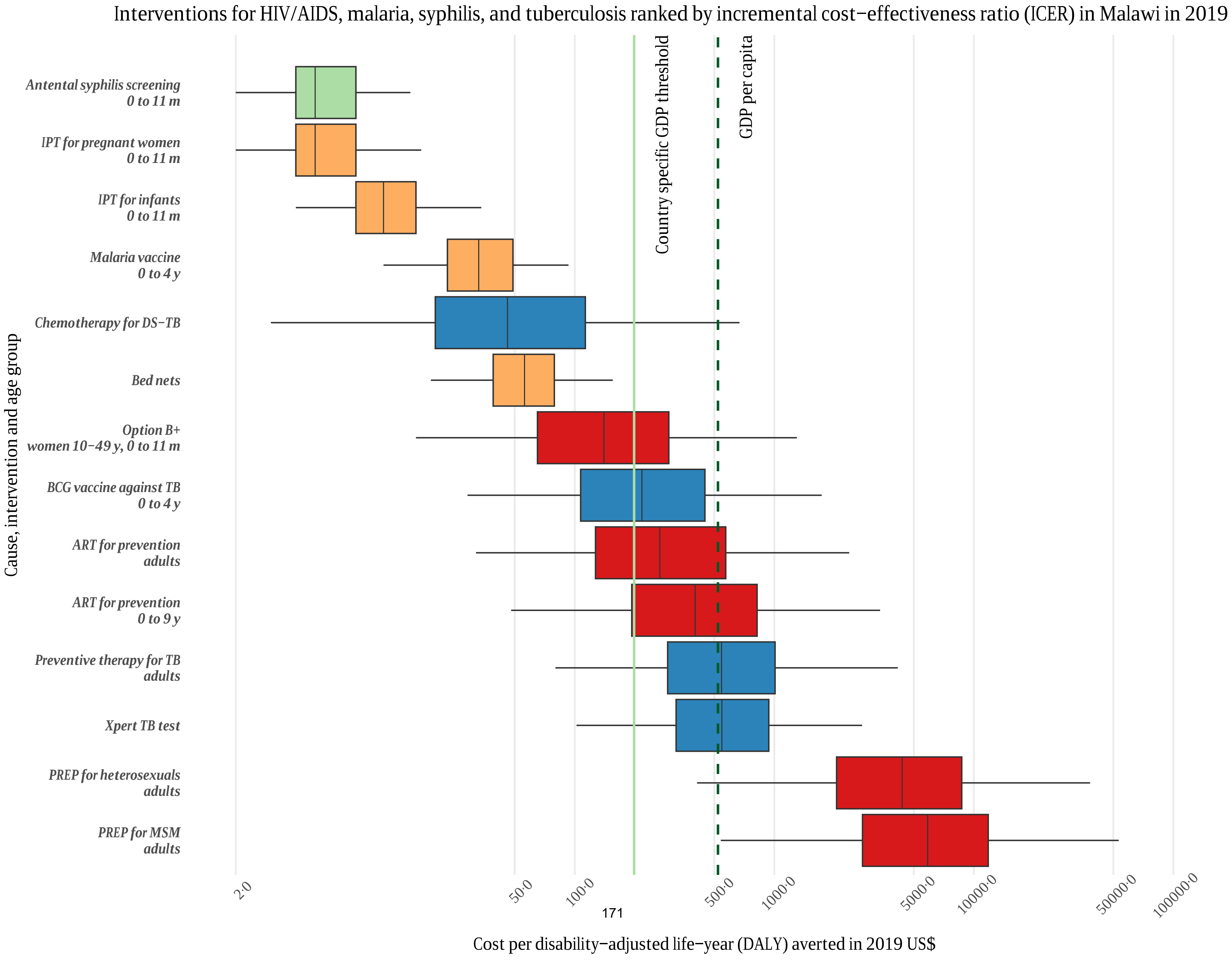

Interventions for HIV/AIDS, malaria, syphilis, and tuberculosis ranked by incremental cost−effectiveness ratio (ICER) in Malawi in 2019
Country specific GDP threshold
GDP per capita
Cause, intervention and age group
Antental syphilis screening
0 to 11 m
IPT for pregnant women
0 to 11 m
IPT for infants
0 to 11 m
Malaria vaccine
0 to 4 y
Chemotherapy for DS−TB
Bed nets
Option B+
women 10−49 y, 0 to 11 m
BCG vaccine against TB
0 to 4 y
ART for prevention
adults
ART for prevention
0 to 9 y
Preventive therapy for TB
adults
Xpert TB test
PREP for heterosexuals
adults
PREP for MSM
adults
2·0
50·0
100·0
171
500·0
1000·0
5000·0
10000·0
50000·0
100000·0
Cost per disability−adjusted life−year (DALY) averted in 2019 US$

Interventions for HIV/AIDS, malaria, syphilis, and tuberculosis ranked by incremental cost−effectiveness ratio (ICER) in Malaysia in 2019

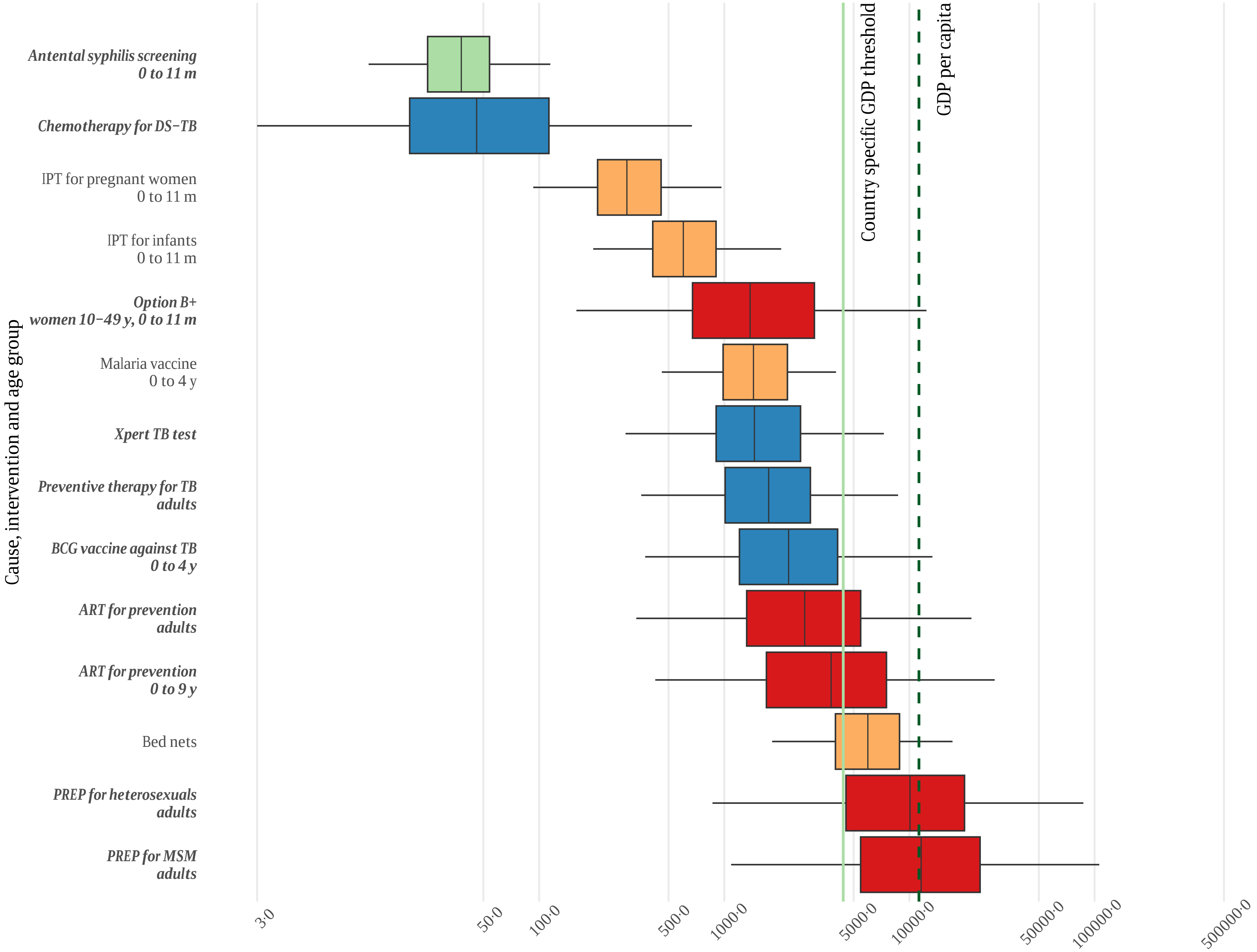

Bolded interventions are eligible for support from The Global Fund.

Interventions for HIV/AIDS, malaria, syphilis, and tuberculosis ranked by incremental cost−effectiveness ratio (ICER) in Maldives in 2019

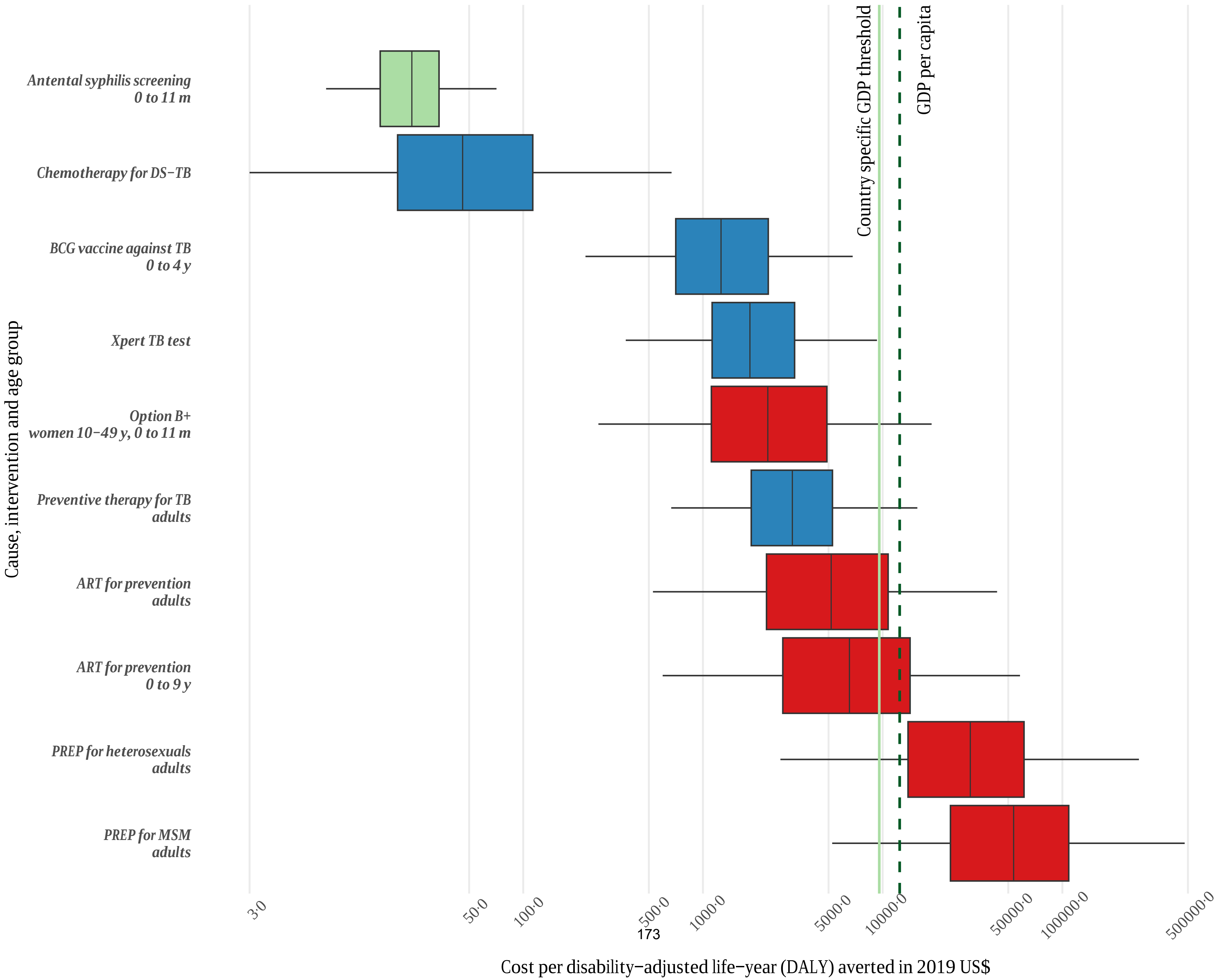

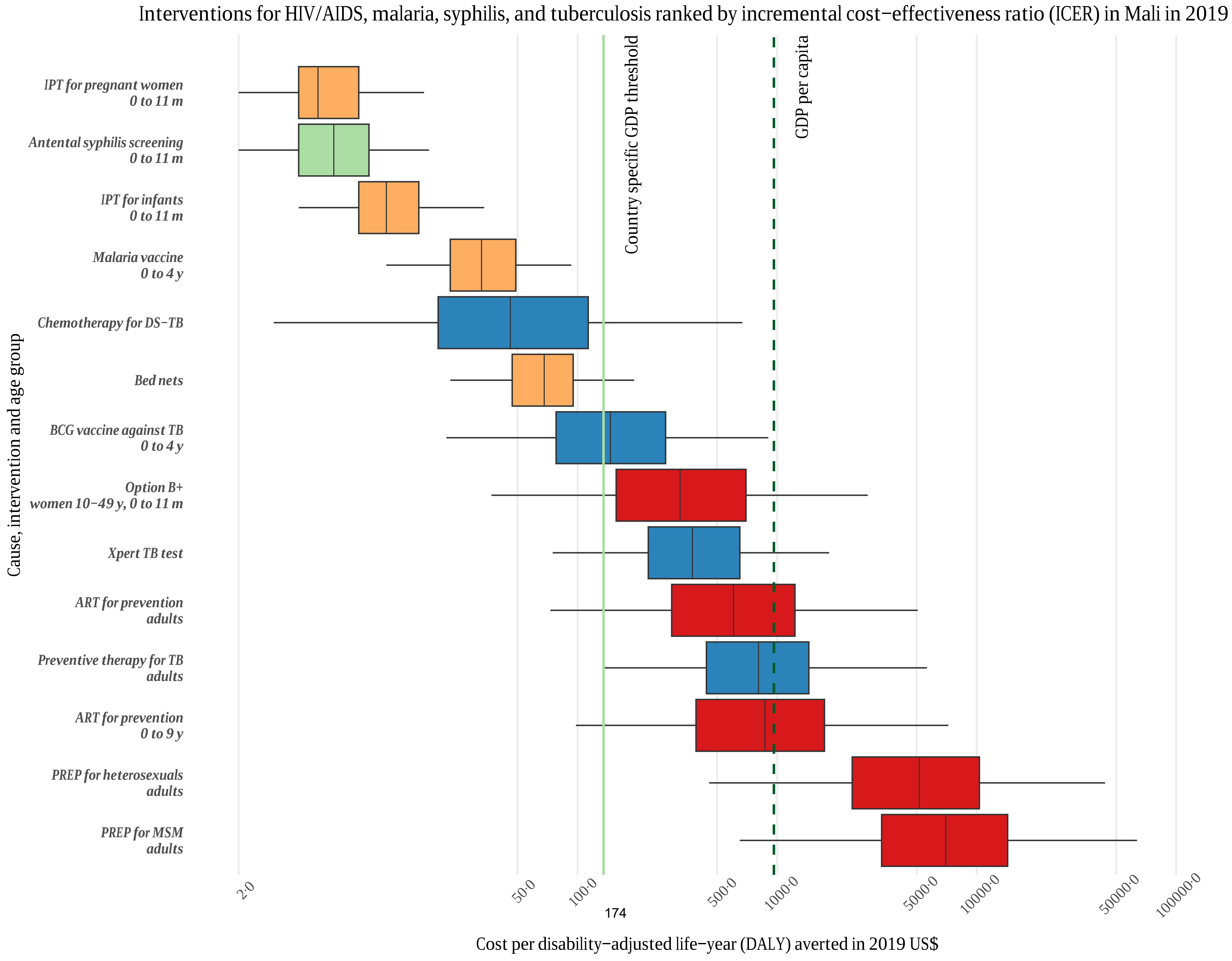
Interventions for HIV/AIDS, malaria, syphilis, and tuberculosis ranked by incremental cost−effectiveness ratio (ICER) in Mali in 2019
Country specific GDP threshold
GDP per capita
IPT for pregnant women
0 to 11 m
Antental syphilis screening
0 to 11 m
IPT for infants
0 to 11 m
Malaria vaccine
0 to 4 y
Chemotherapy for DS−TB
Bed nets
BCG vaccine against TB
0 to 4 y
Option B+
women 10−49 y, 0 to 11 m
Xpert TB test
ART for prevention
adults
Preventive therapy for TB
adults
ART for prevention
0 to 9 y
PREP for heterosexuals
adults
PREP for MSM
adults
Cause, intervention and age group
2·0
50·0
100·0
174
500·0
1000·0
5000·0
10000·0
50000·0
100000·0
Cost per disability−adjusted life−year (DALY) averted in 2019 US$

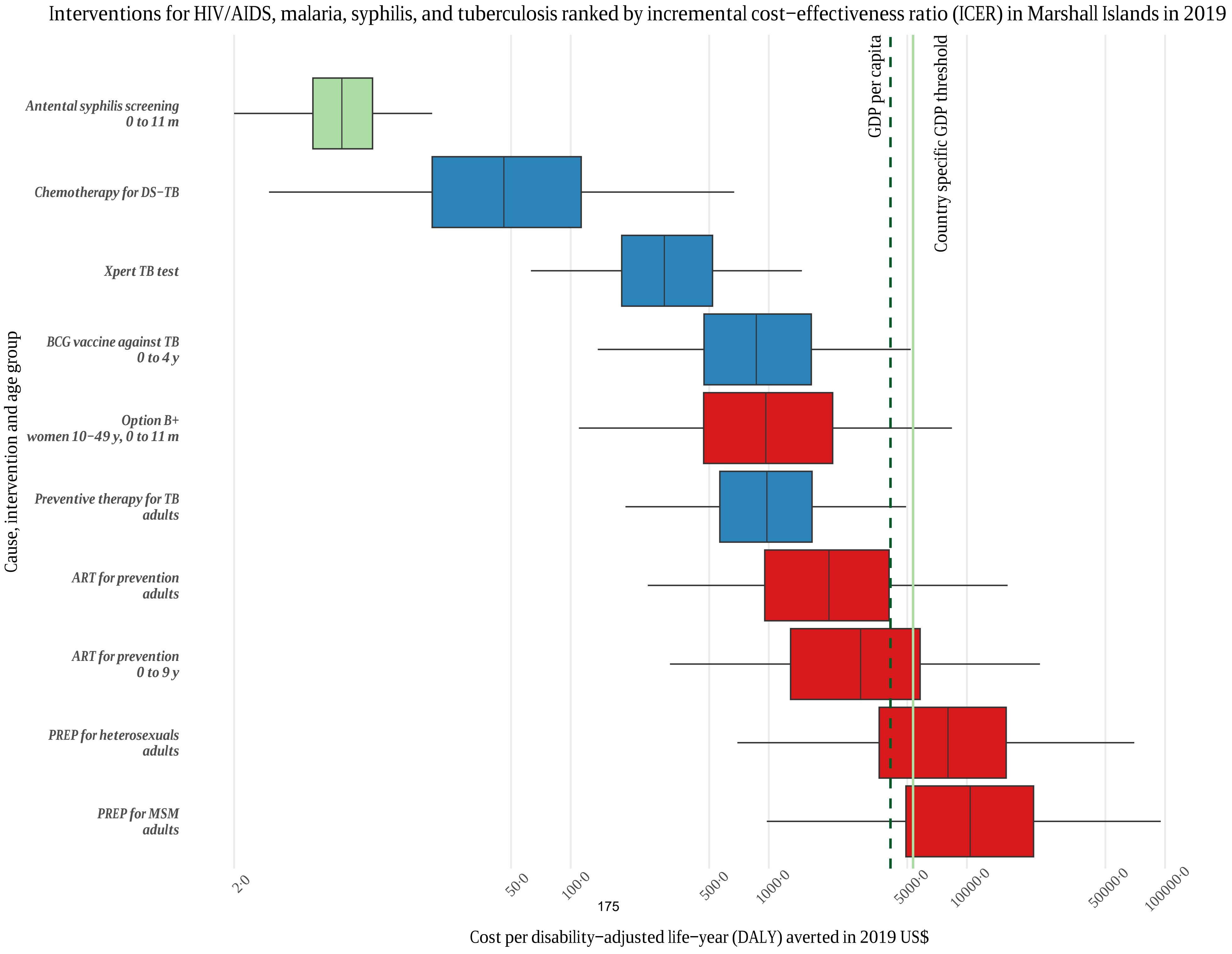
Interventions for HIV/AIDS, malaria, syphilis, and tuberculosis ranked by incremental cost−effectiveness ratio (ICER) in Marshall Islands in 2019
GDP per capita
Country specific GDP threshold
Cause, intervention and age group
Antental syphilis screening
0 to 11 m
Chemotherapy for DS−TB
Xpert TB test
BCG vaccine against TB
0 to 4 y
Option B+
women 10−49 y, 0 to 11 m
Preventive therapy for TB
adults
ART for prevention
adults
ART for prevention
0 to 9 y
PREP for heterosexuals
adults
PREP for MSM
adults
2·0
50·0
100·0
500·0
1000·0
5000·0
10000·0
50000·0
100000·0
Cost per disability−adjusted life−year (DALY) averted in 2019 US$

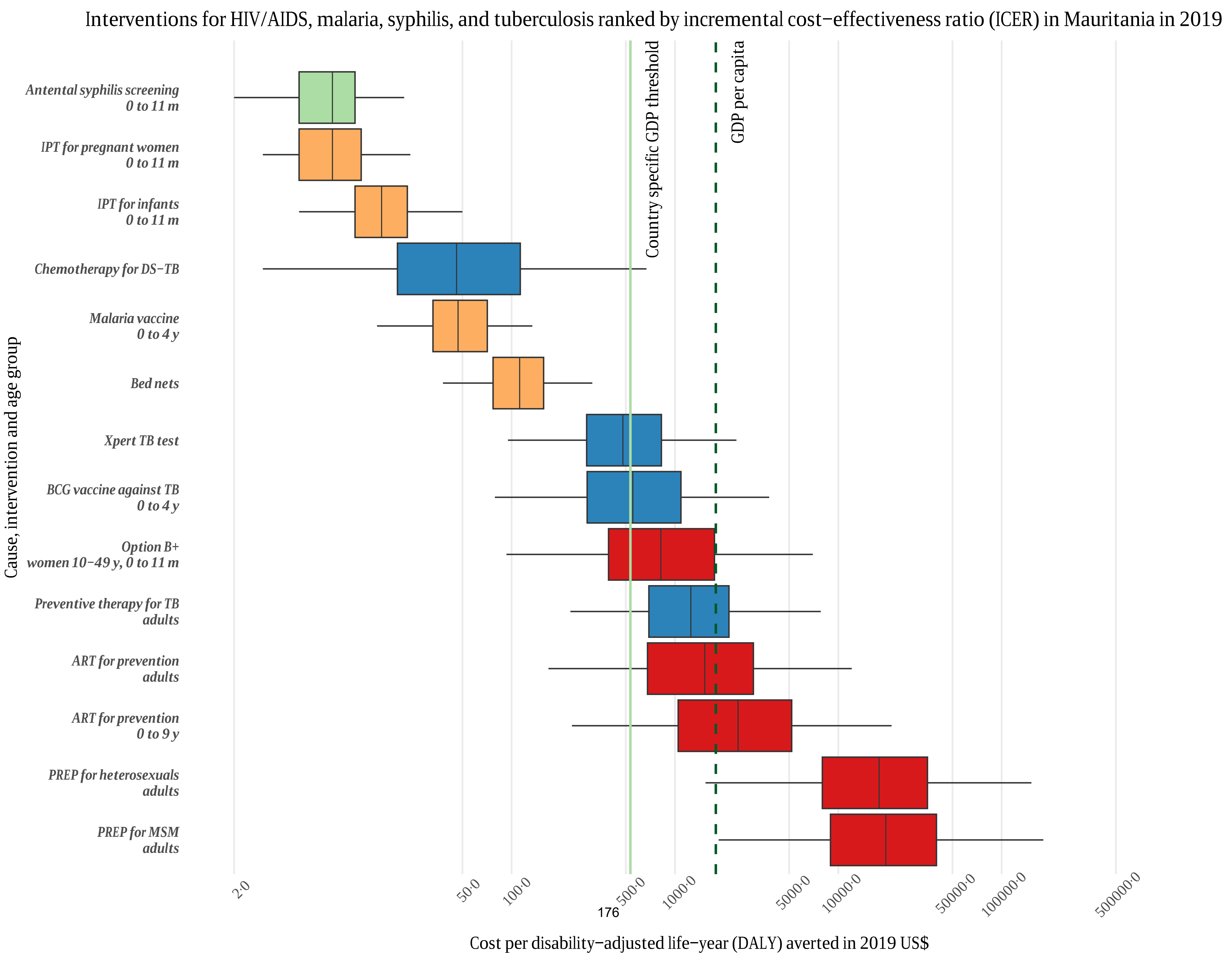
Interventions for HIV/AIDS, malaria, syphilis, and tuberculosis ranked by incremental cost−effectiveness ratio (ICER) in Mauritania in 2019
Country specific GDP threshold
GDP per capita
Antental syphilis screening 0 to 11 m
IPT for pregnant women 0 to 11 m
IPT for infants 0 to 11 m
Chemotherapy for DS−TB
Malaria vaccine 0 to 4 y
Bed nets
Xpert TB test
BCG vaccine against TB 0 to 4 y
Option B+ women 10−49 y, 0 to 11 m
Preventive therapy for TB adults
ART for prevention adults
ART for prevention 0 to 9 y
PREP for heterosexuals adults
PREP for MSM adults
Cause, intervention and age group
2·0
50·0
100·0
500·0
1000·0
5000·0
10000·0
50000·0
100000·0
500000·0
176
Cost per disability−adjusted life−year (DALY) averted in 2019 US$

Interventions for HIV/AIDS, malaria, syphilis, and tuberculosis ranked by incremental cost−effectiveness ratio (ICER) in Mauritius in 2019

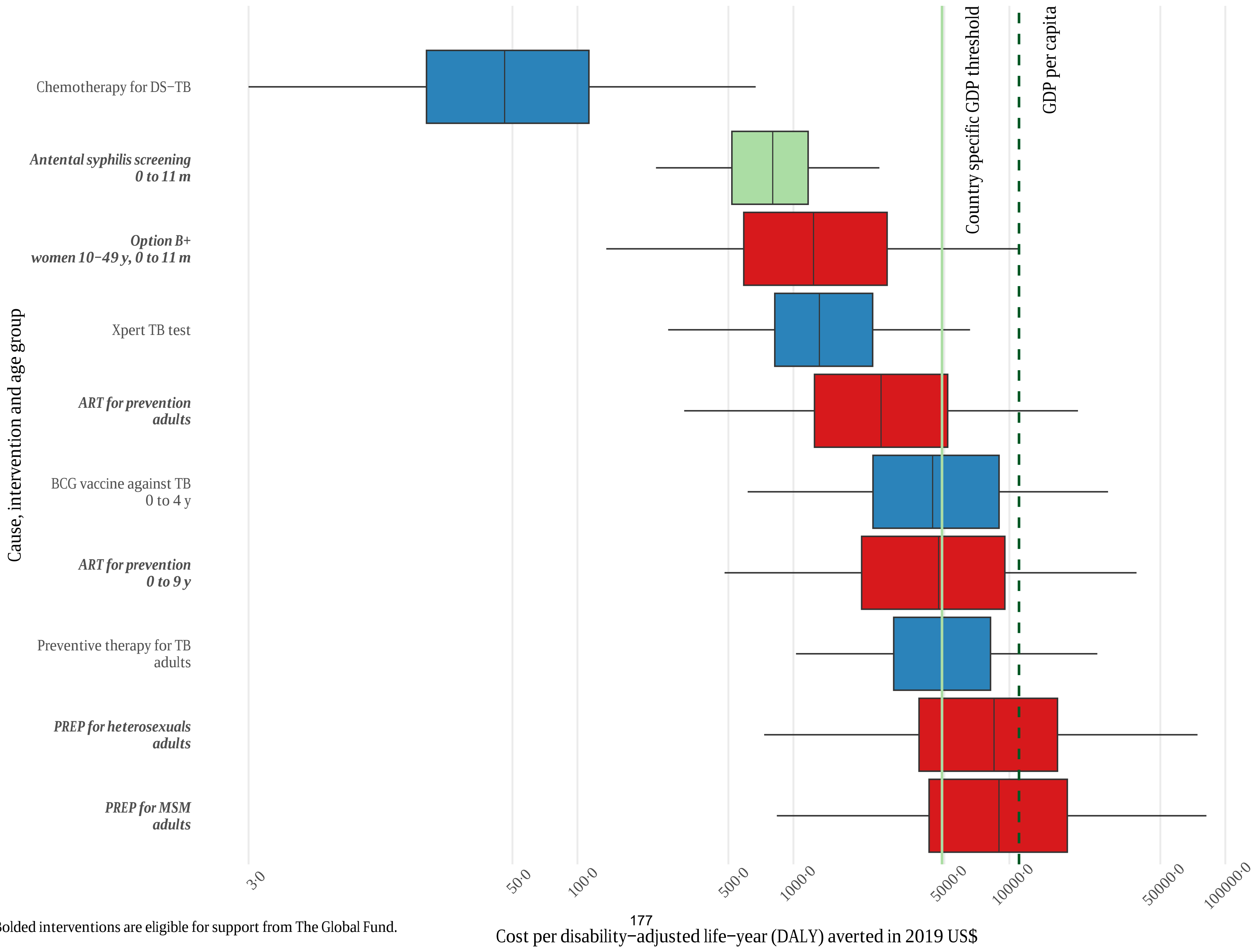

Bolded interventions are eligible for support from The Global Fund.

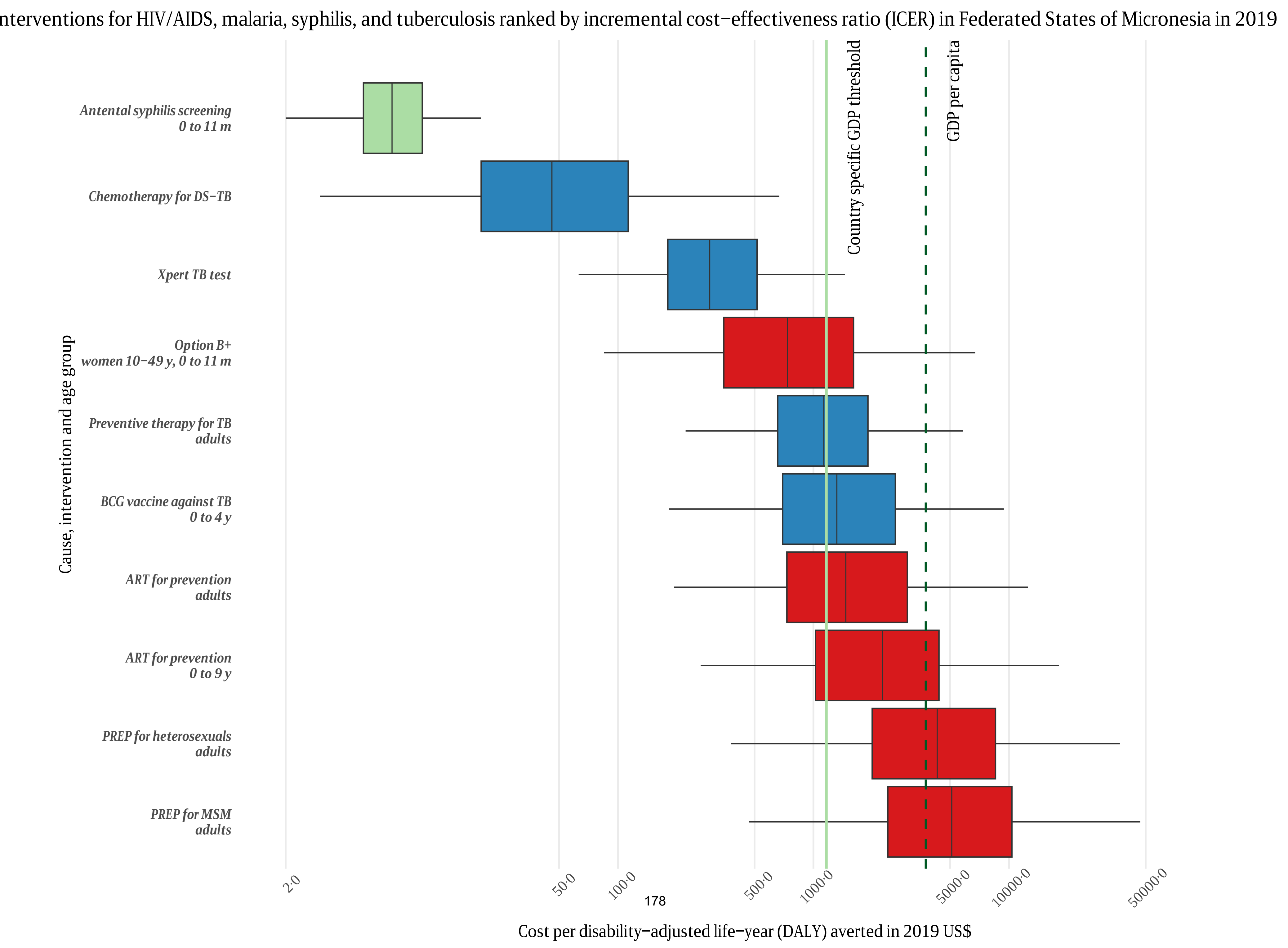

nterventions for HIV/AIDS, malaria, syphilis, and tuberculosis ranked by incremental cost−effectiveness ratio (ICER) in Federated States of Micronesia in 2019
Cause, intervention and age group
Antental syphilis screening
0 to 11 m
Chemotherapy for DS−TB
Xpert TB test
Option B+
women 10−49 y, 0 to 11 m
Preventive therapy for TB
adults
BCG vaccine against TB
0 to 4 y
ART for prevention
adults
ART for prevention
0 to 9 y
PREP for heterosexuals
adults
PREP for MSM
adults
Country specific GDP threshold
GDP per capita
2·0
50·0
100·0
500·0
1000·0
5000·0
10000·0
50000·0
Cost per disability−adjusted life−year (DALY) averted in 2019 US$

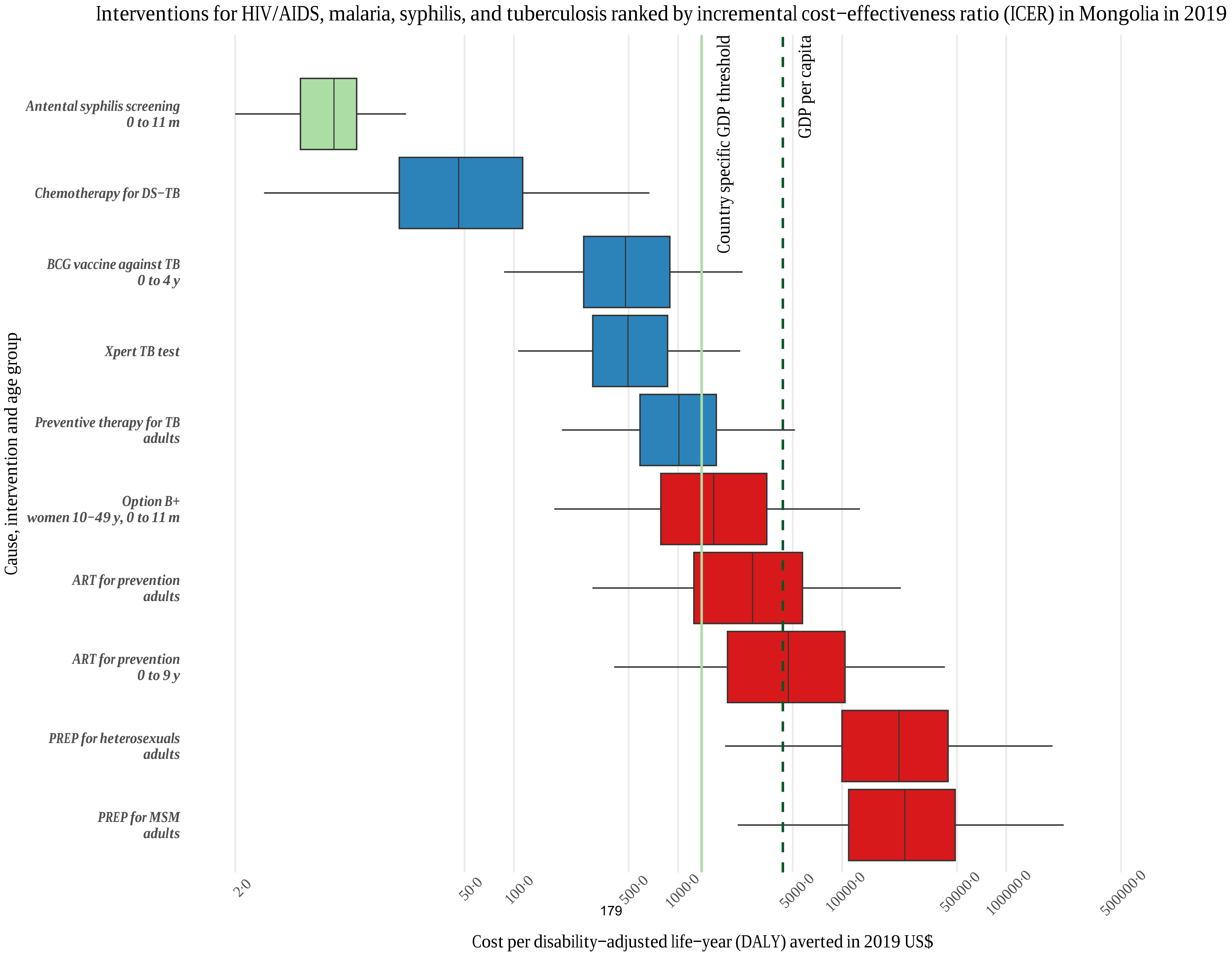
Interventions for HIV/AIDS, malaria, syphilis, and tuberculosis ranked by incremental cost−effectiveness ratio (ICER) in Mongolia in 2019
Country specific GDP threshold
GDP per capita
Antental syphilis screening 0 to 11 m
Chemotherapy for DS−TB
BCG vaccine against TB 0 to 4 y
Xpert TB test
Preventive therapy for TB adults
Option B+ women 10−49 y, 0 to 11 m
ART for prevention adults
ART for prevention 0 to 9 y
PREP for heterosexuals adults
PREP for MSM adults
Cause, intervention and age group
2·0
50·0
100·0
500·0
1000·0
5000·0
10000·0
50000·0
100000·0
500000·0
Cost per disability−adjusted life−year (DALY) averted in 2019 US$

Interventions for HIV/AIDS, malaria, syphilis, and tuberculosis ranked by incremental cost−effectiveness ratio (ICER) in Montenegro in 2019

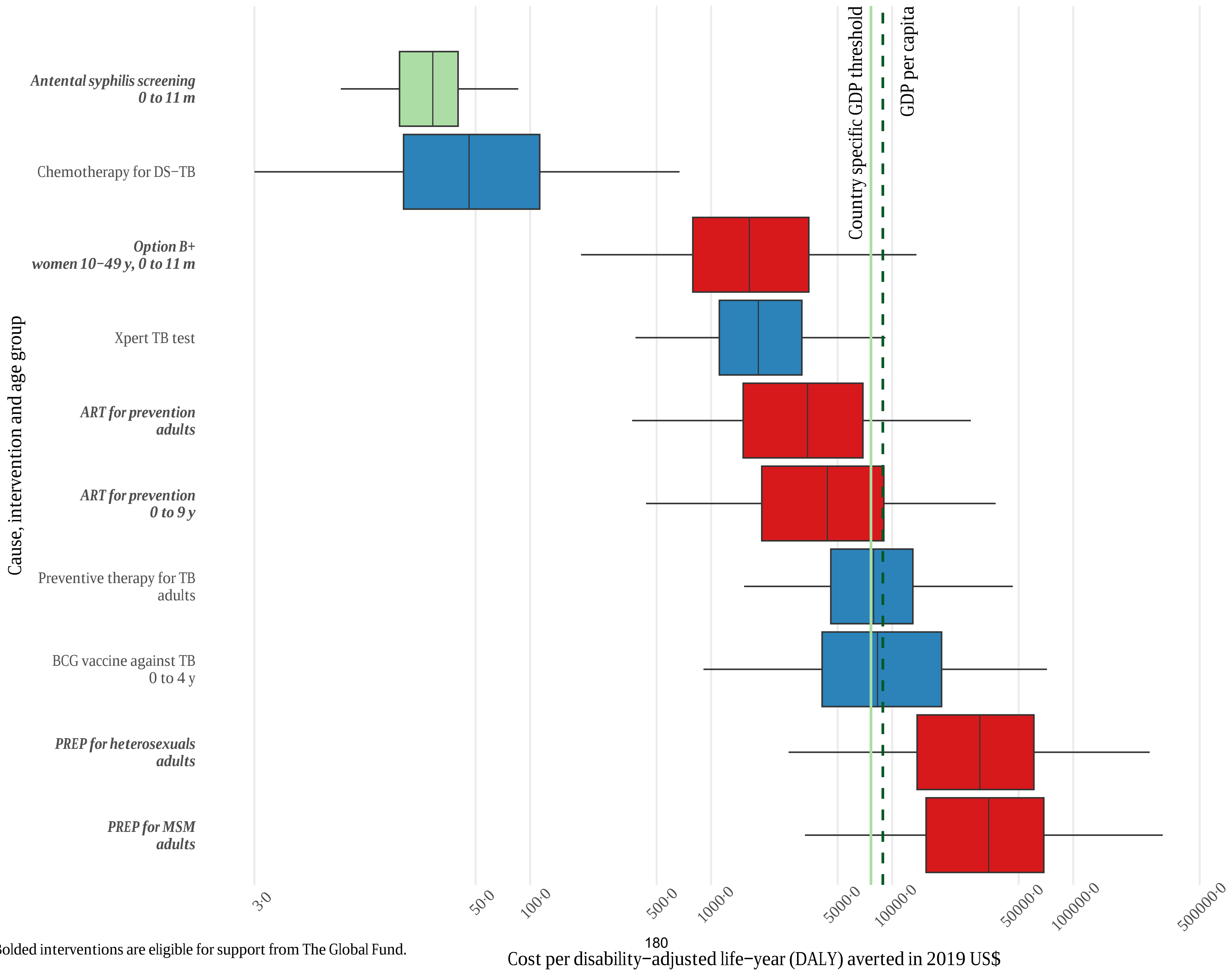

Bolded interventions are eligible for support from The Global Fund.

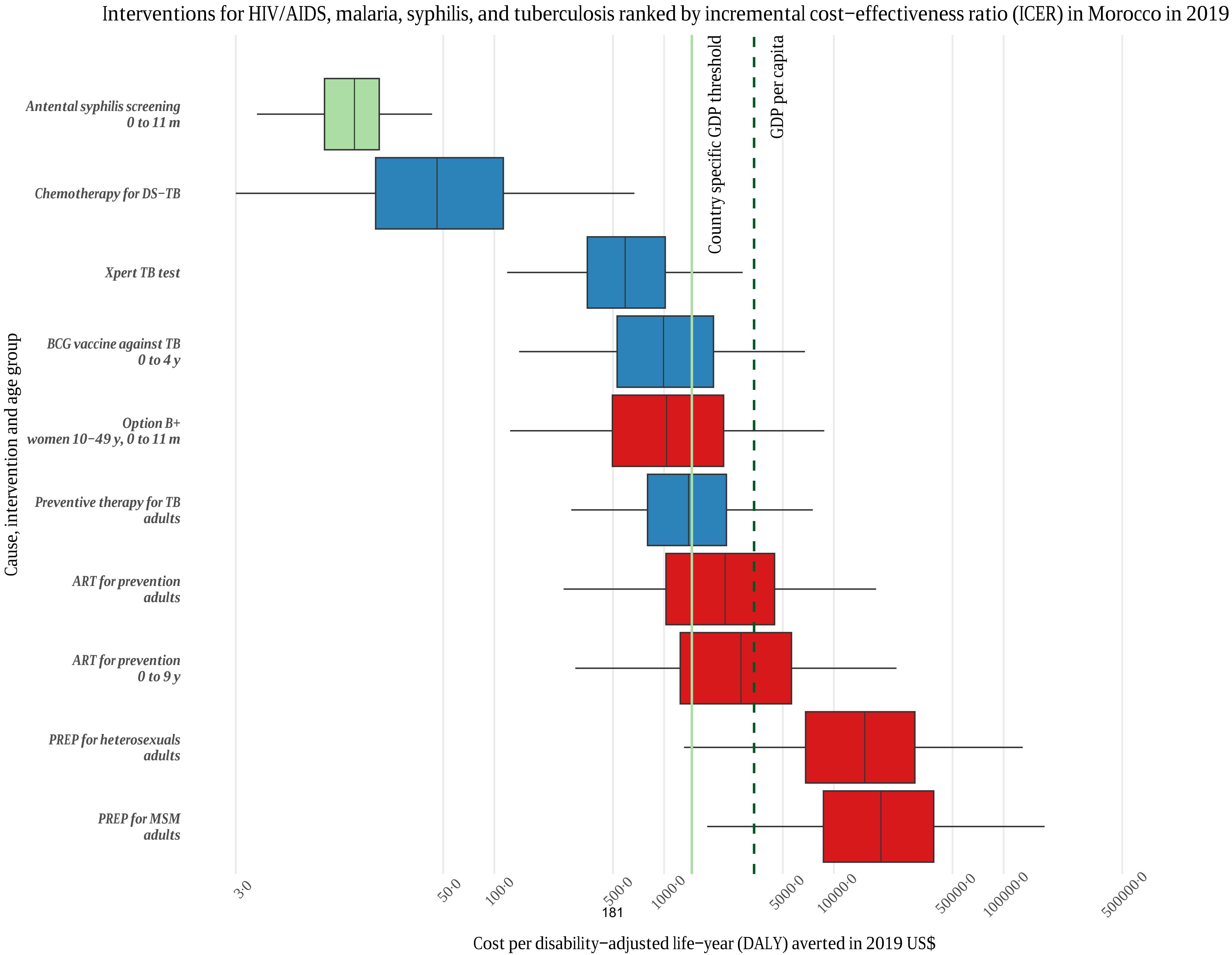

Interventions for HIV/AIDS, malaria, syphilis, and tuberculosis ranked by incremental cost−effectiveness ratio (ICER) in Morocco in 2019
Antental syphilis screening
0 to 11 m
Chemotherapy for DS−TB
Xpert TB test
BCG vaccine against TB
0 to 4 y
Option B+
women 10−49 y, 0 to 11 m
Preventive therapy for TB
adults
ART for prevention
adults
ART for prevention
0 to 9 y
PREP for heterosexuals
adults
PREP for MSM
adults
Country specific GDP threshold
GDP per capita
Cause, intervention and age group
3·0
50·0
100·0
500·0
1000·0
5000·0
10000·0
50000·0
100000·0
500000·0
Cost per disability−adjusted life−year (DALY) averted in 2019 US$

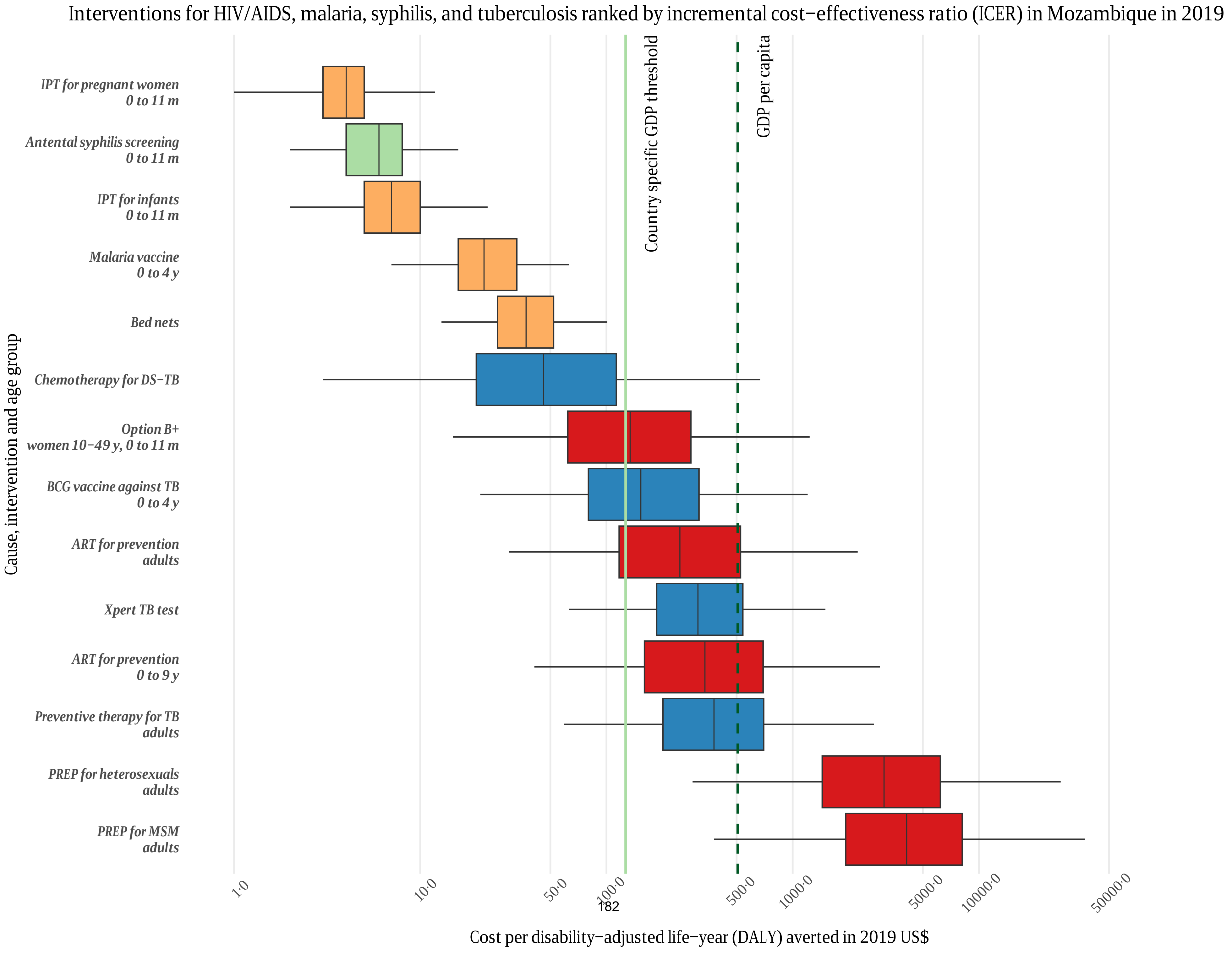

Interventions for HIV/AIDS, malaria, syphilis, and tuberculosis ranked by incremental cost−effectiveness ratio (ICER) in Mozambique in 2019
Country specific GDP threshold
GDP per capita
IPT for pregnant women 0 to 11 m
Antental syphilis screening 0 to 11 m
IPT for infants 0 to 11 m
Malaria vaccine 0 to 4 y
Bed nets
Chemotherapy for DS−TB
Option B+ women 10−49 y, 0 to 11 m
BCG vaccine against TB 0 to 4 y
ART for prevention adults
Xpert TB test
ART for prevention 0 to 9 y
Preventive therapy for TB adults
PREP for heterosexuals adults
PREP for MSM adults
Cause, intervention and age group
1.0
10.0
50.0
100.0
182
500.0
1000.0
5000.0
10000.0
50000.0
Cost per disability−adjusted life−year (DALY) averted in 2019 US$

Interventions for HIV/AIDS, malaria, syphilis, and tuberculosis ranked by incremental cost−effectiveness ratio (ICER) in Myanmar in 2019

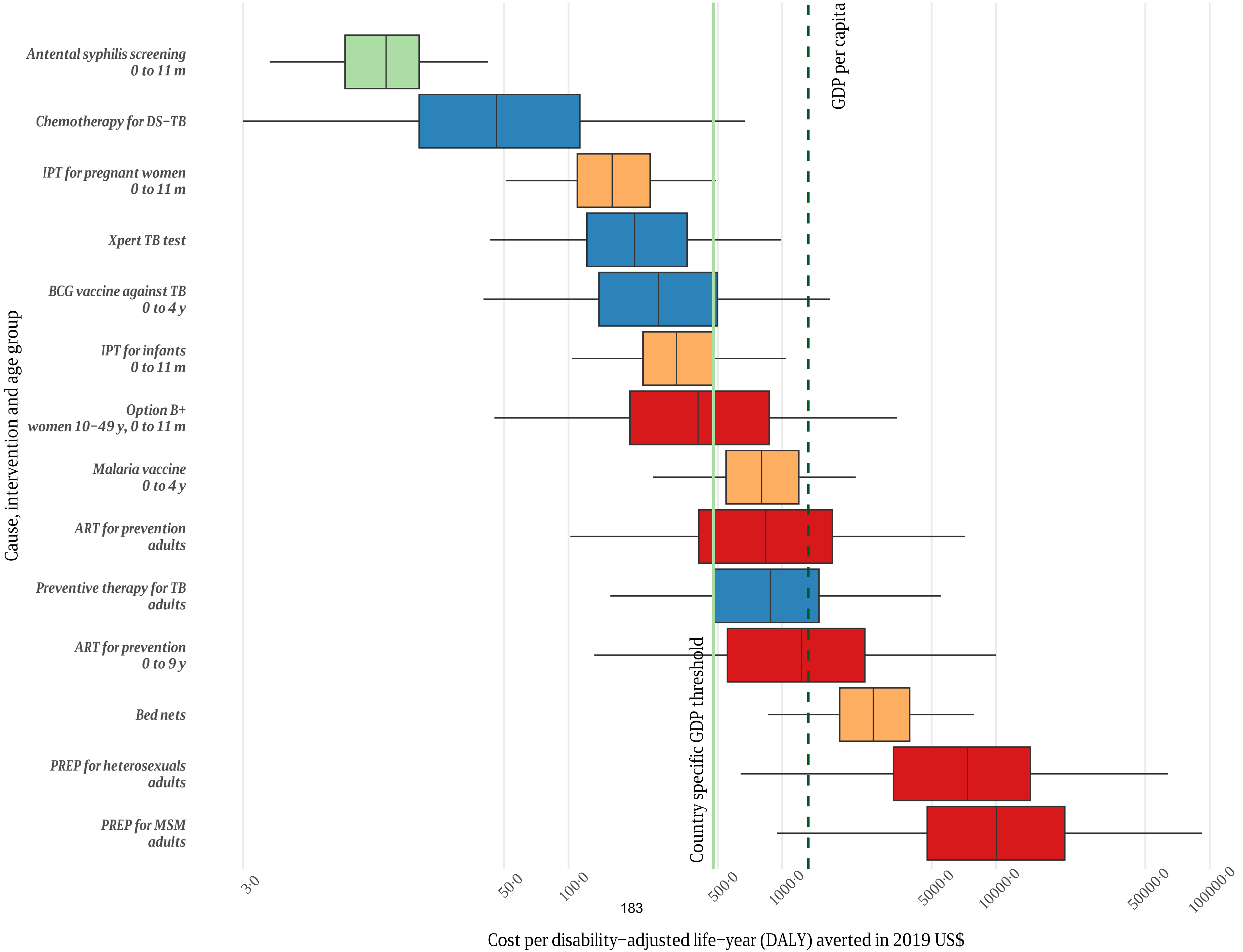

Interventions for HIV/AIDS, malaria, syphilis, and tuberculosis ranked by incremental cost−effectiveness ratio (ICER) in Namibia in 2019

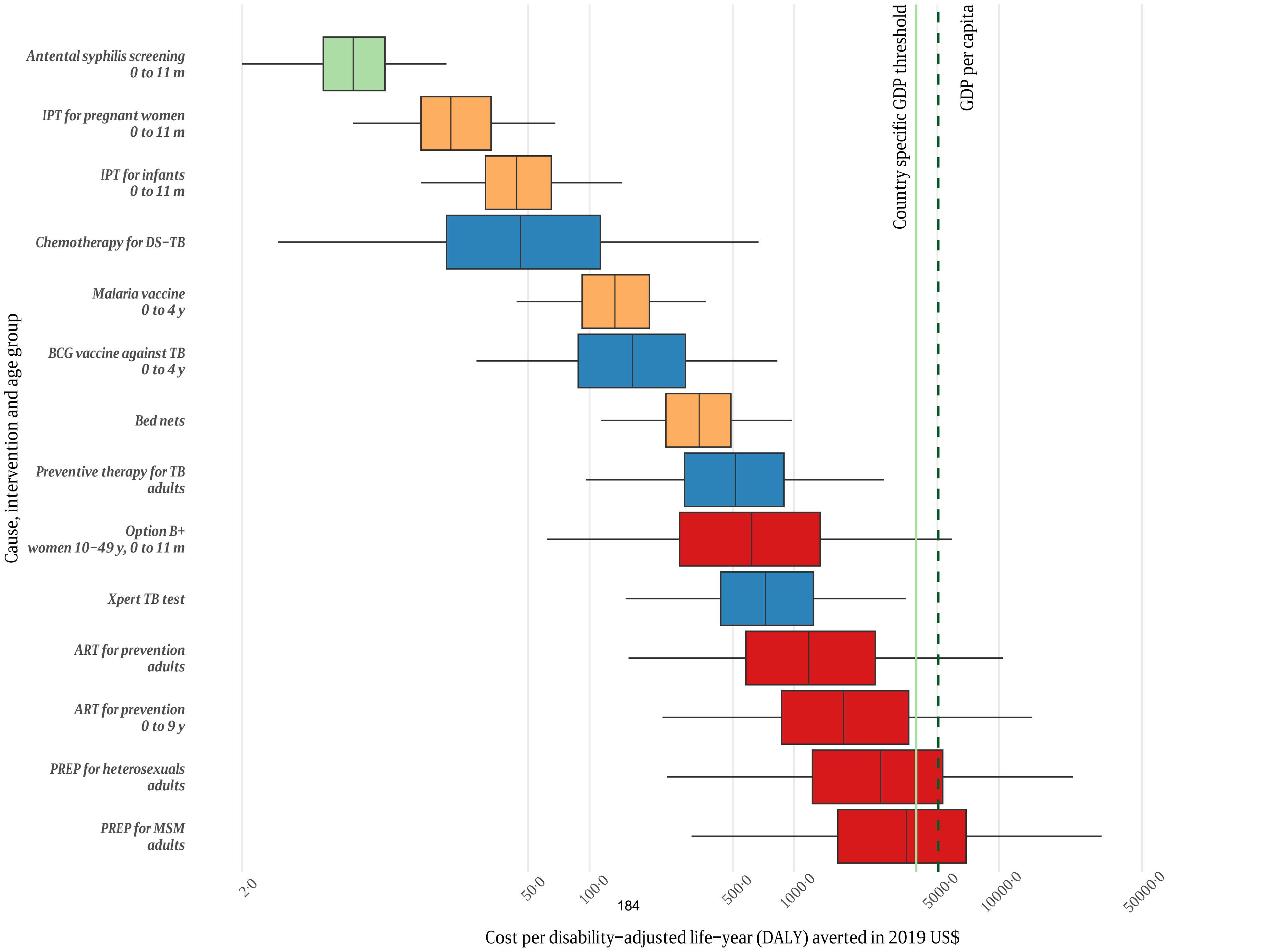

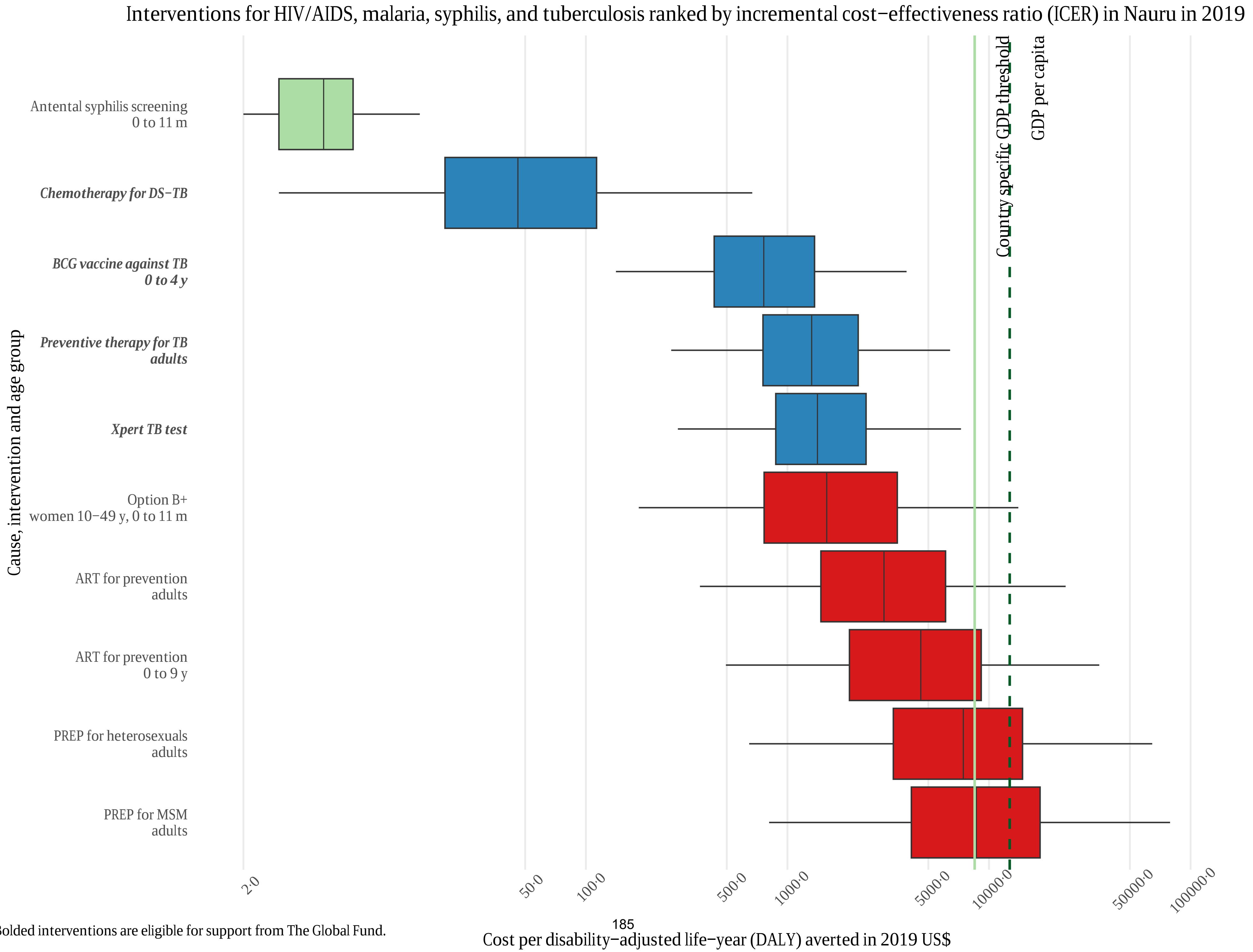

Bolded interventions are eligible for support from The Global Fund.

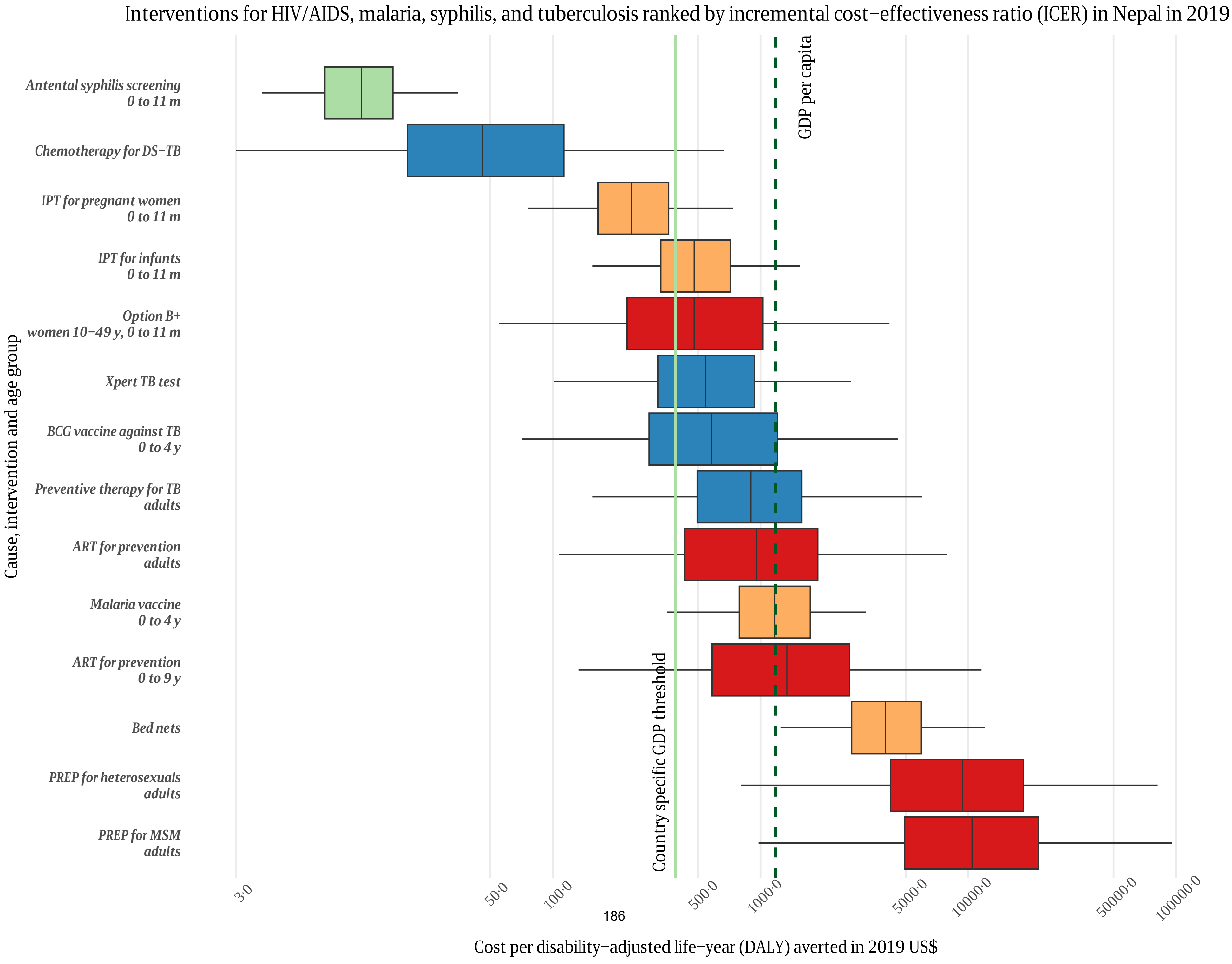

Interventions for HIV/AIDS, malaria, syphilis, and tuberculosis ranked by incremental cost−effectiveness ratio (ICER) in Nepal in 2019
Antental syphilis screening
0 to 11 m
Chemotherapy for DS−TB
IPT for pregnant women
0 to 11 m
IPT for infants
0 to 11 m
Option B+
women 10−49 y, 0 to 11 m
Xpert TB test
BCG vaccine against TB
0 to 4 y
Preventive therapy for TB
adults
ART for prevention
adults
Malaria vaccine
0 to 4 y
ART for prevention
0 to 9 y
Bed nets
PREP for heterosexuals
adults
PREP for MSM
adults
GDP per capita
Country specific GDP threshold
Cause, intervention and age group
3·0
50·0
100·0
186
500·0
1000·0
5000·0
10000·0
50000·0
100000·0
Cost per disability−adjusted life−year (DALY) averted in 2019 US$

Interventions for HIV/AIDS, malaria, syphilis, and tuberculosis ranked by incremental cost−effectiveness ratio (ICER) in Nicaragua in 2019

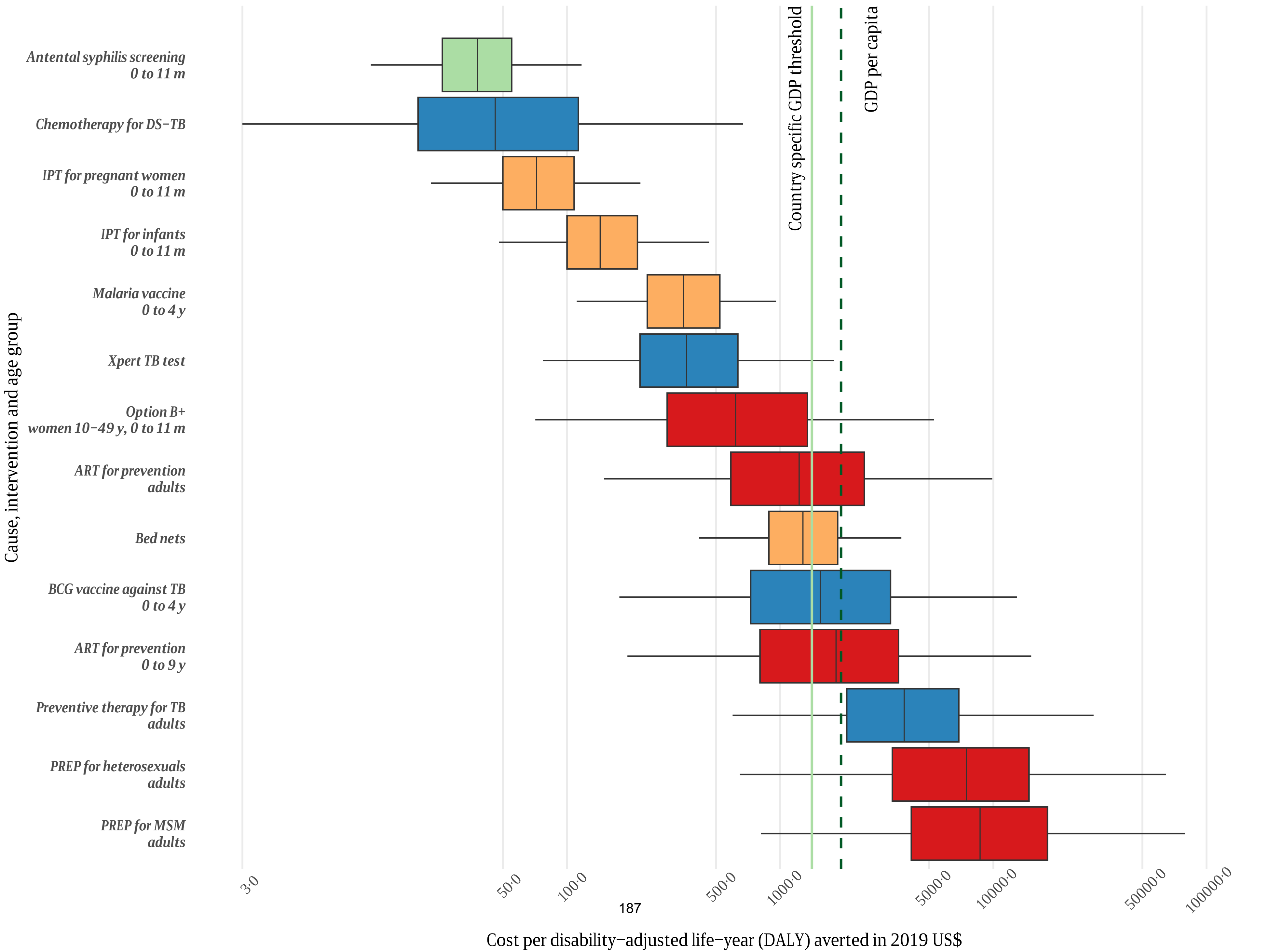

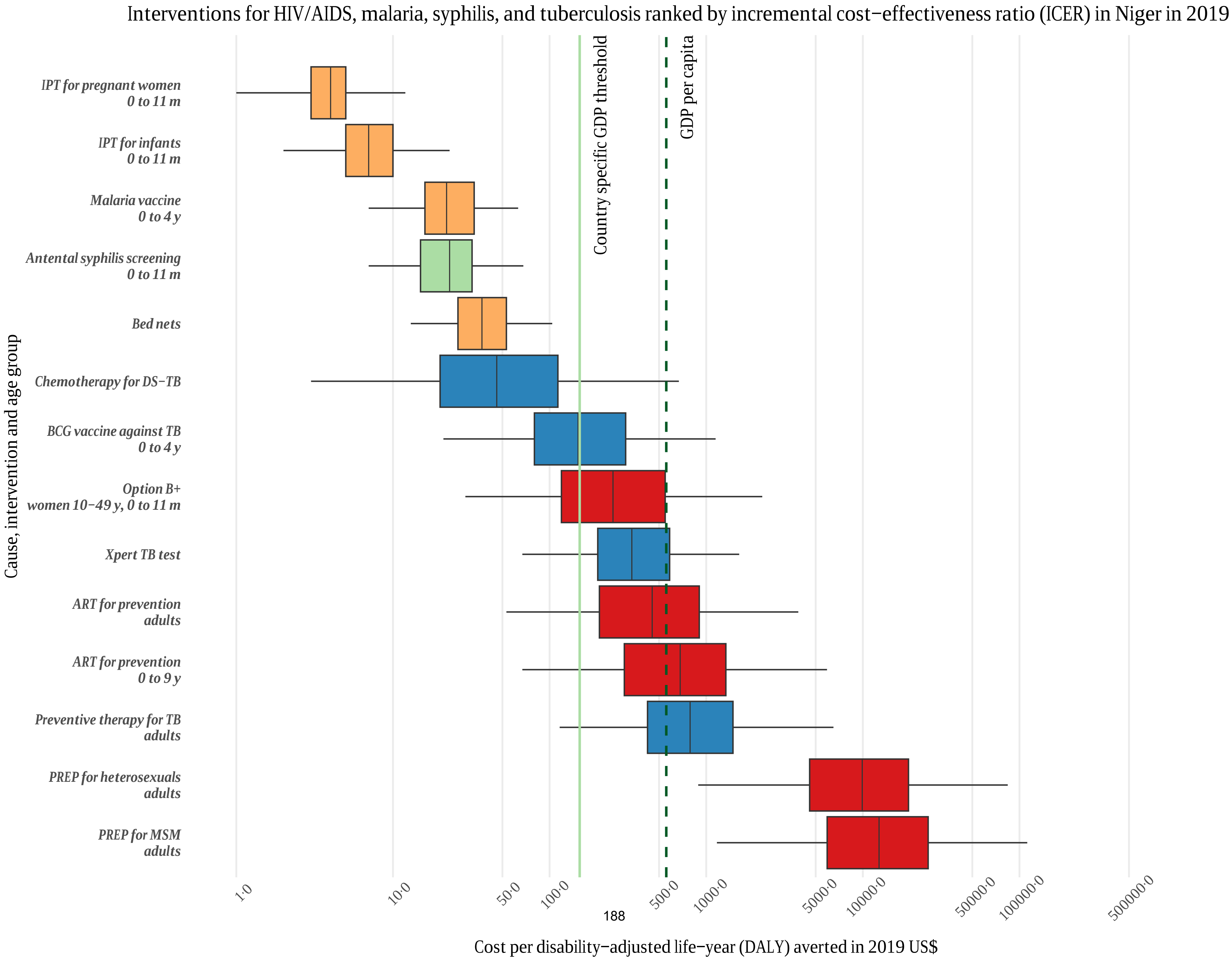
Interventions for HIV/AIDS, malaria, syphilis, and tuberculosis ranked by incremental cost−effectiveness ratio (ICER) in Niger in 2019
Country specific GDP threshold
GDP per capita
IPT for pregnant women 0 to 11 m
IPT for infants 0 to 11 m
Malaria vaccine 0 to 4 y
Antental syphilis screening 0 to 11 m
Bed nets
Chemotherapy for DS−TB
BCG vaccine against TB 0 to 4 y
Option B+ women 10−49 y, 0 to 11 m
Xpert TB test
ART for prevention adults
ART for prevention 0 to 9 y
Preventive therapy for TB adults
PREP for heterosexuals adults
PREP for MSM adults
Cause, intervention and age group
1·0
10·0
50·0
100·0
500·0
1000·0
5000·0
10000·0
50000·0
100000·0
500000·0
Cost per disability−adjusted life−year (DALY) averted in 2019 US$

188

Interventions for HIV/AIDS, malaria, syphilis, and tuberculosis ranked by incremental cost−effectiveness ratio (ICER) in Nigeria in 2019

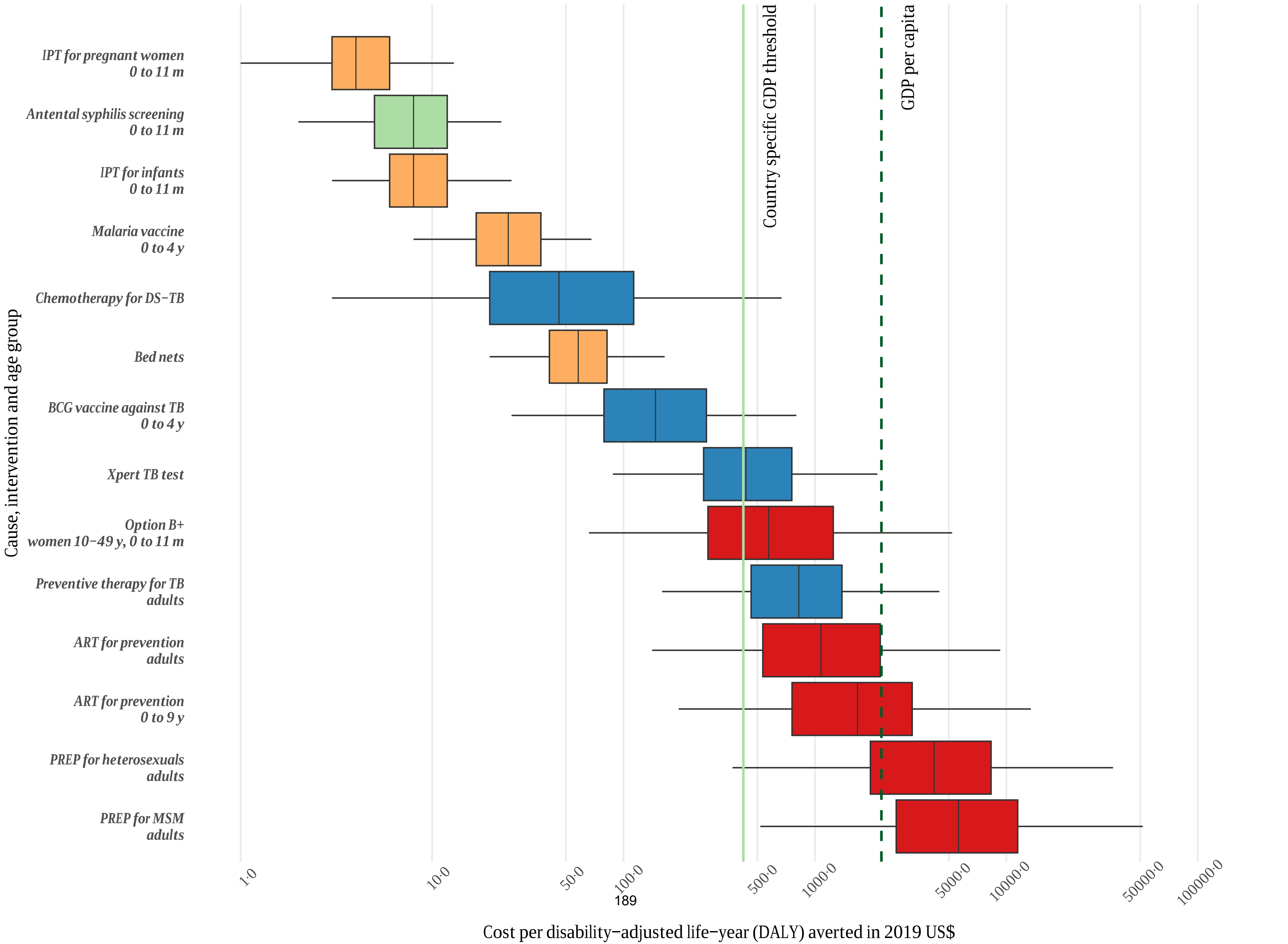

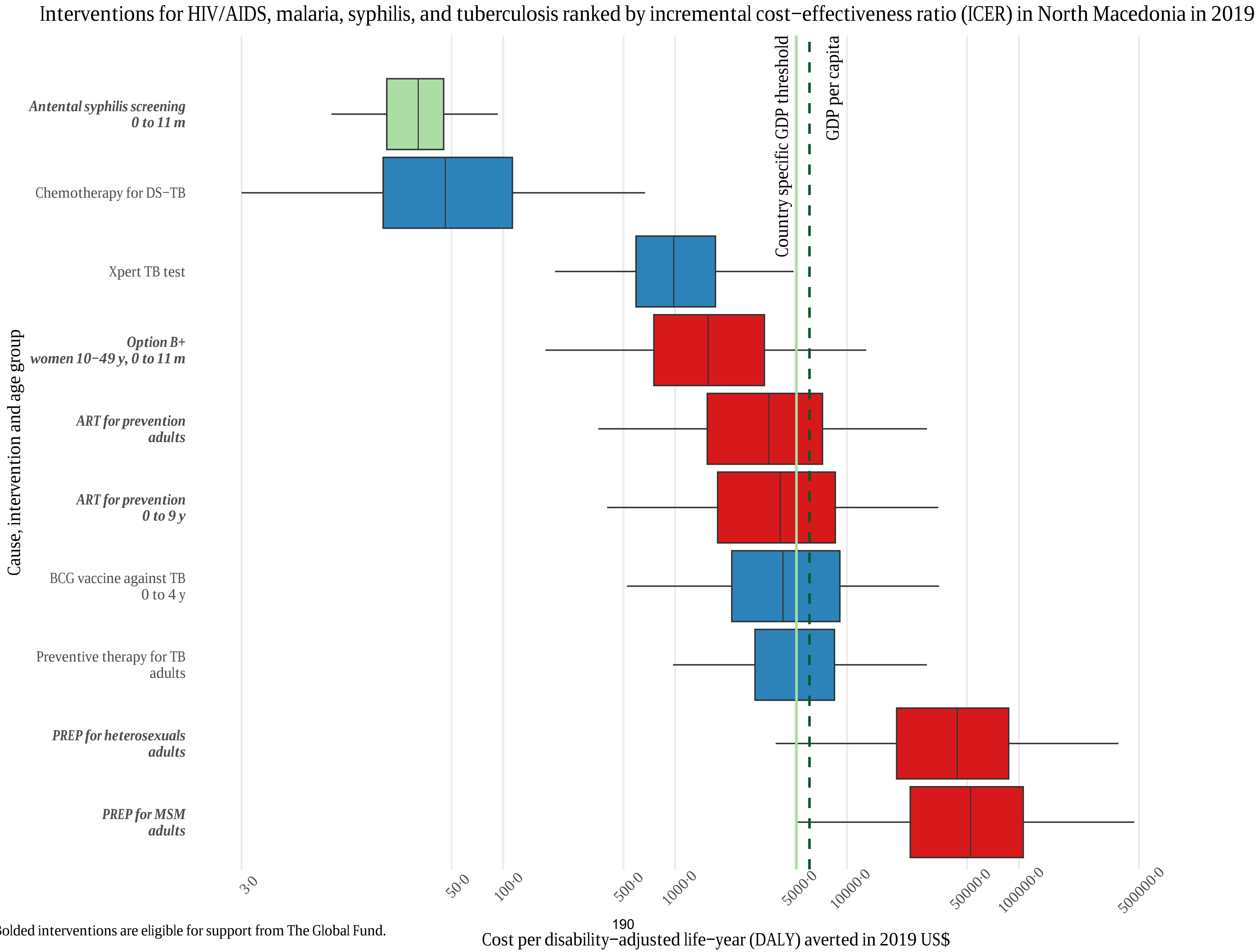

Bolded interventions are eligible for support from The Global Fund.

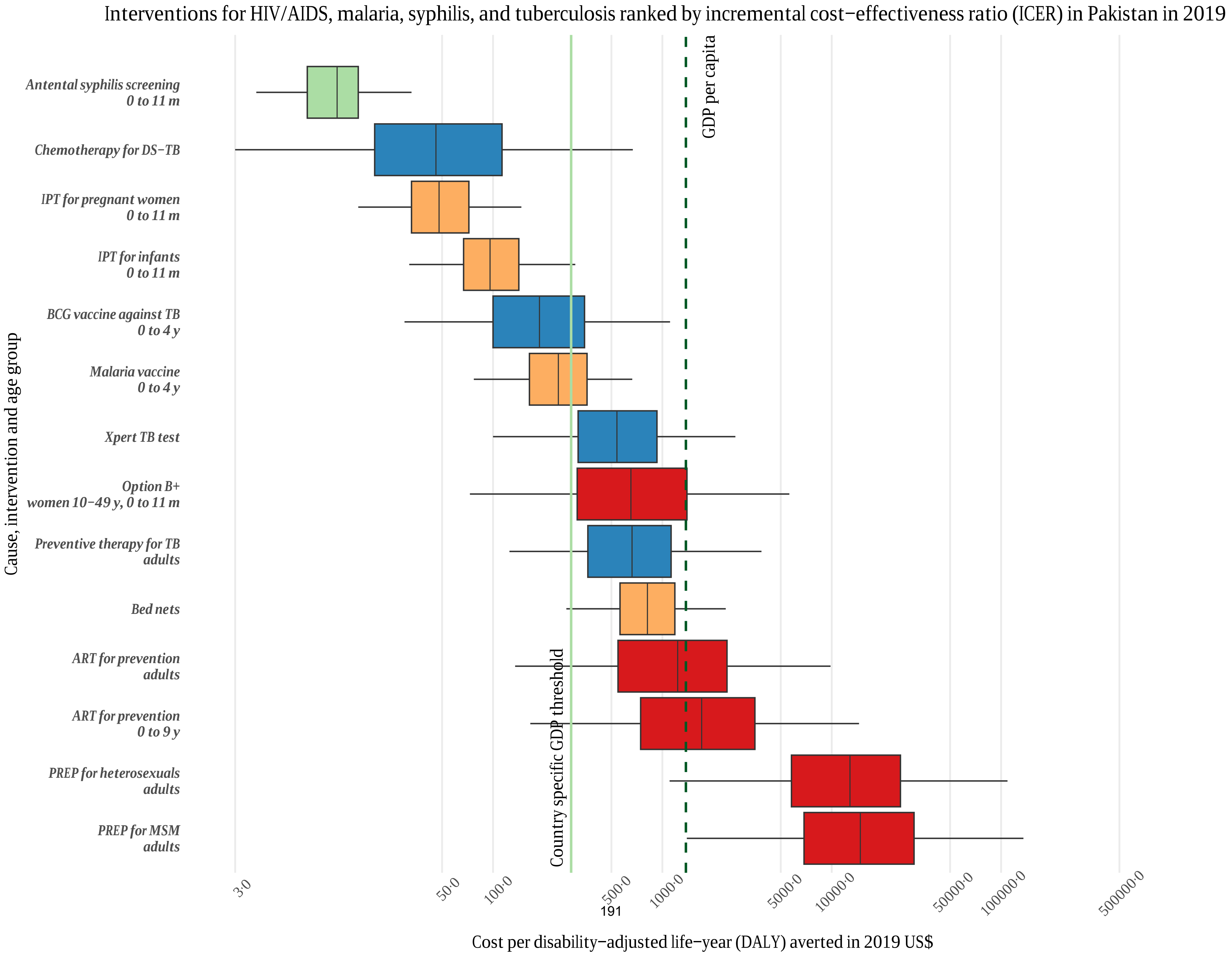

Interventions for HIV/AIDS, malaria, syphilis, and tuberculosis ranked by incremental cost−effectiveness ratio (ICER) in Pakistan in 2019
Antental syphilis screening 0 to 11 m
Chemotherapy for DS−TB
IPT for pregnant women 0 to 11 m
IPT for infants 0 to 11 m
BCG vaccine against TB 0 to 4 y
Malaria vaccine 0 to 4 y
Xpert TB test
Option B+ women 10−49 y, 0 to 11 m
Preventive therapy for TB adults
Bed nets
ART for prevention adults
ART for prevention 0 to 9 y
PREP for heterosexuals adults
PREP for MSM adults
GDP per capita
Country specific GDP threshold
Cause, intervention and age group
3·0
50·0
100·0
500·0
1000·0
5000·0
10000·0
50000·0
100000·0
500000·0
191
Cost per disability−adjusted life−year (DALY) averted in 2019 US$

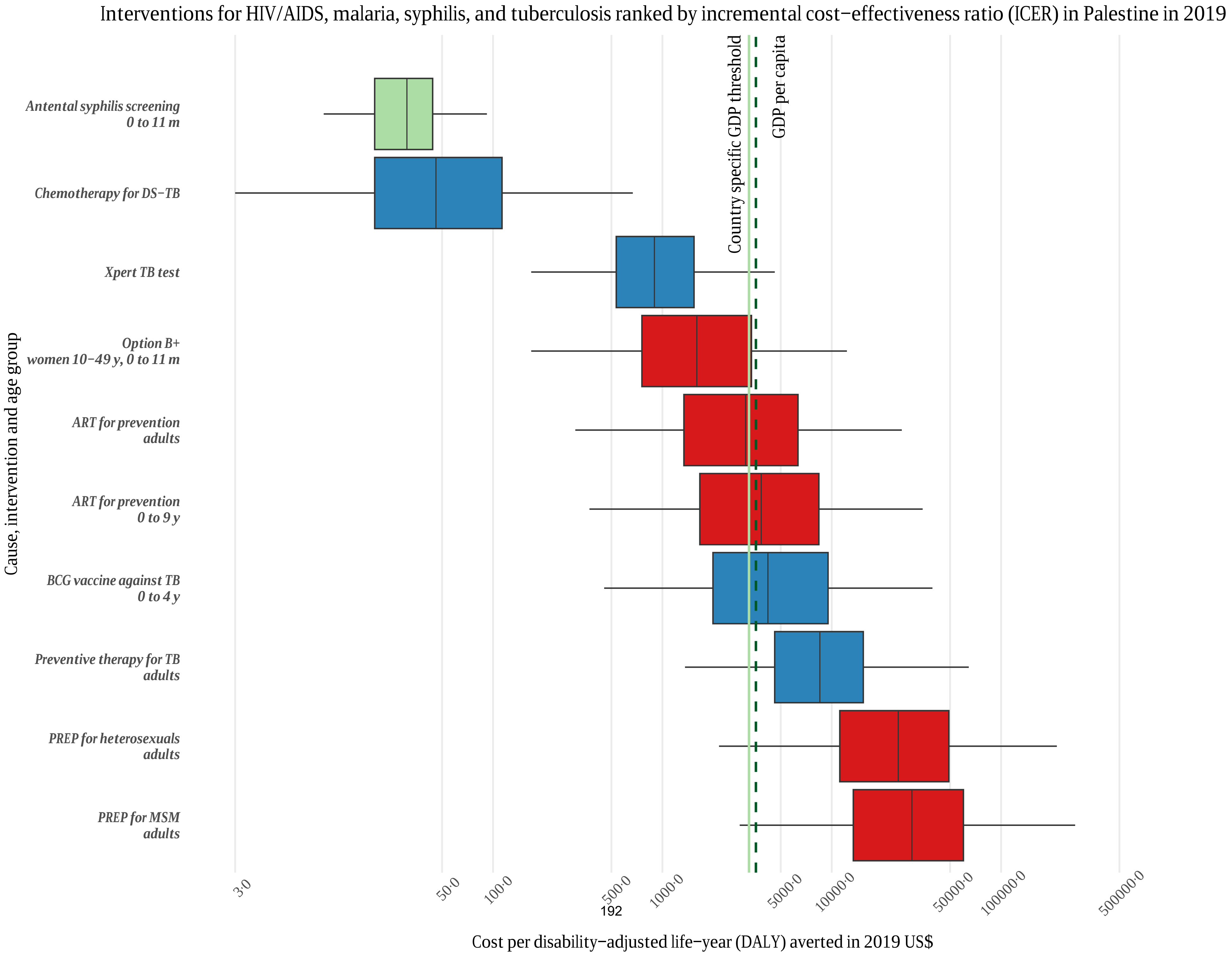
Interventions for HIV/AIDS, malaria, syphilis, and tuberculosis ranked by incremental cost−effectiveness ratio (ICER) in Palestine in 2019
Country specific GDP threshold
GDP per capita
Antental syphilis screening
0 to 11 m
Chemotherapy for DS−TB
Xpert TB test
Option B+
women 10−49 y, 0 to 11 m
ART for prevention
adults
ART for prevention
0 to 9 y
BCG vaccine against TB
0 to 4 y
Preventive therapy for TB
adults
PREP for heterosexuals
adults
PREP for MSM
adults
Cause, intervention and age group
3·0
50·0
100·0
500·0
1000·0
5000·0
10000·0
50000·0
100000·0
500000·0
Cost per disability−adjusted life−year (DALY) averted in 2019 US$

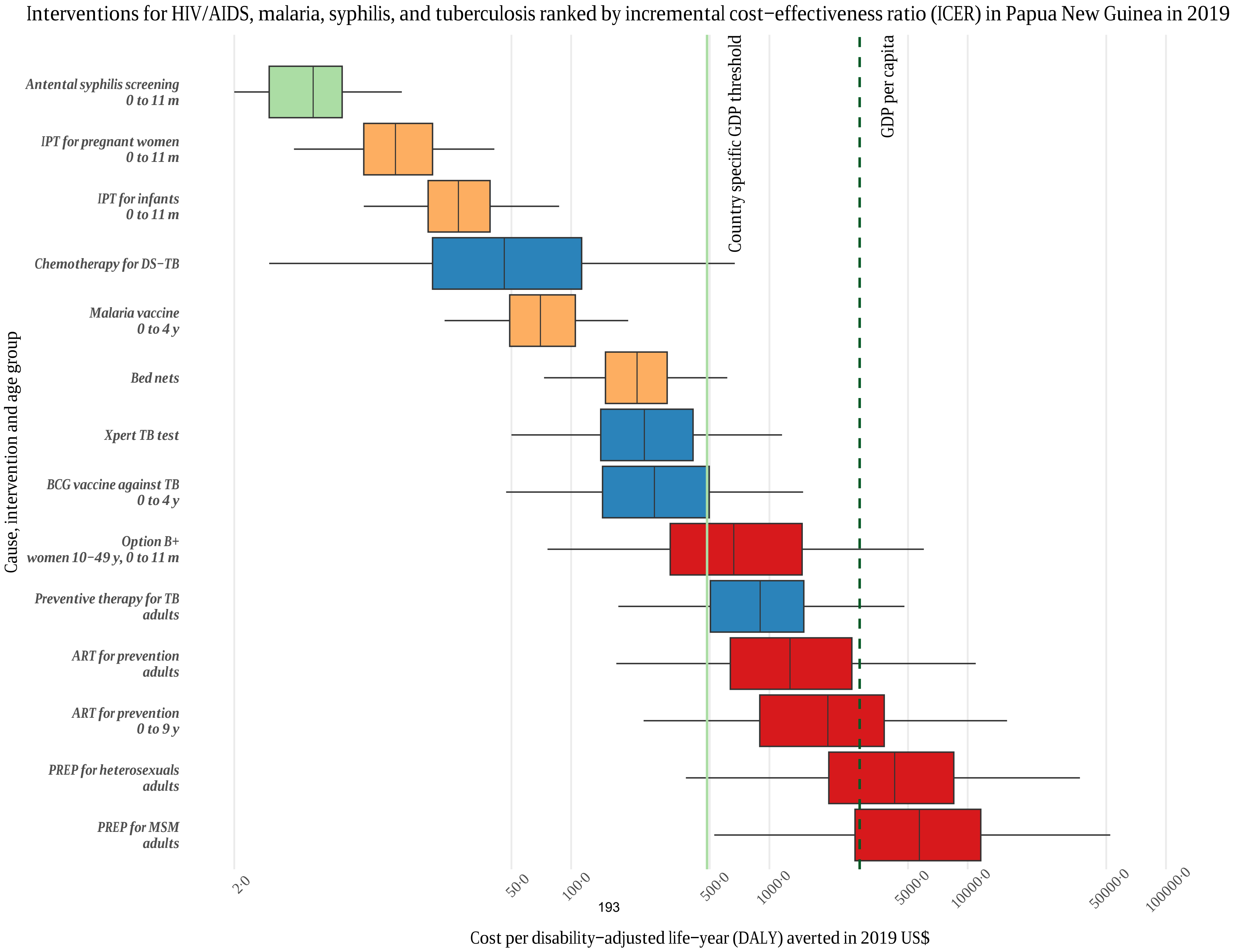

Interventions for HIV/AIDS, malaria, syphilis, and tuberculosis ranked by incremental cost−effectiveness ratio (ICER) in Papua New Guinea in 2019
Antental syphilis screening
0 to 11 m
IPT for pregnant women
0 to 11 m
IPT for infants
0 to 11 m
Chemotherapy for DS−TB
Malaria vaccine
0 to 4 y
Bed nets
Xpert TB test
BCG vaccine against TB
0 to 4 y
Option B+
women 10−49 y, 0 to 11 m
Preventive therapy for TB
adults
ART for prevention
adults
ART for prevention
0 to 9 y
PREP for heterosexuals
adults
PREP for MSM
adults
Country specific GDP threshold
GDP per capita
Cause, intervention and age group
2·0
50·0
100·0
500·0
1000·0
5000·0
10000·0
50000·0
100000·0
Cost per disability−adjusted life−year (DALY) averted in 2019 US$

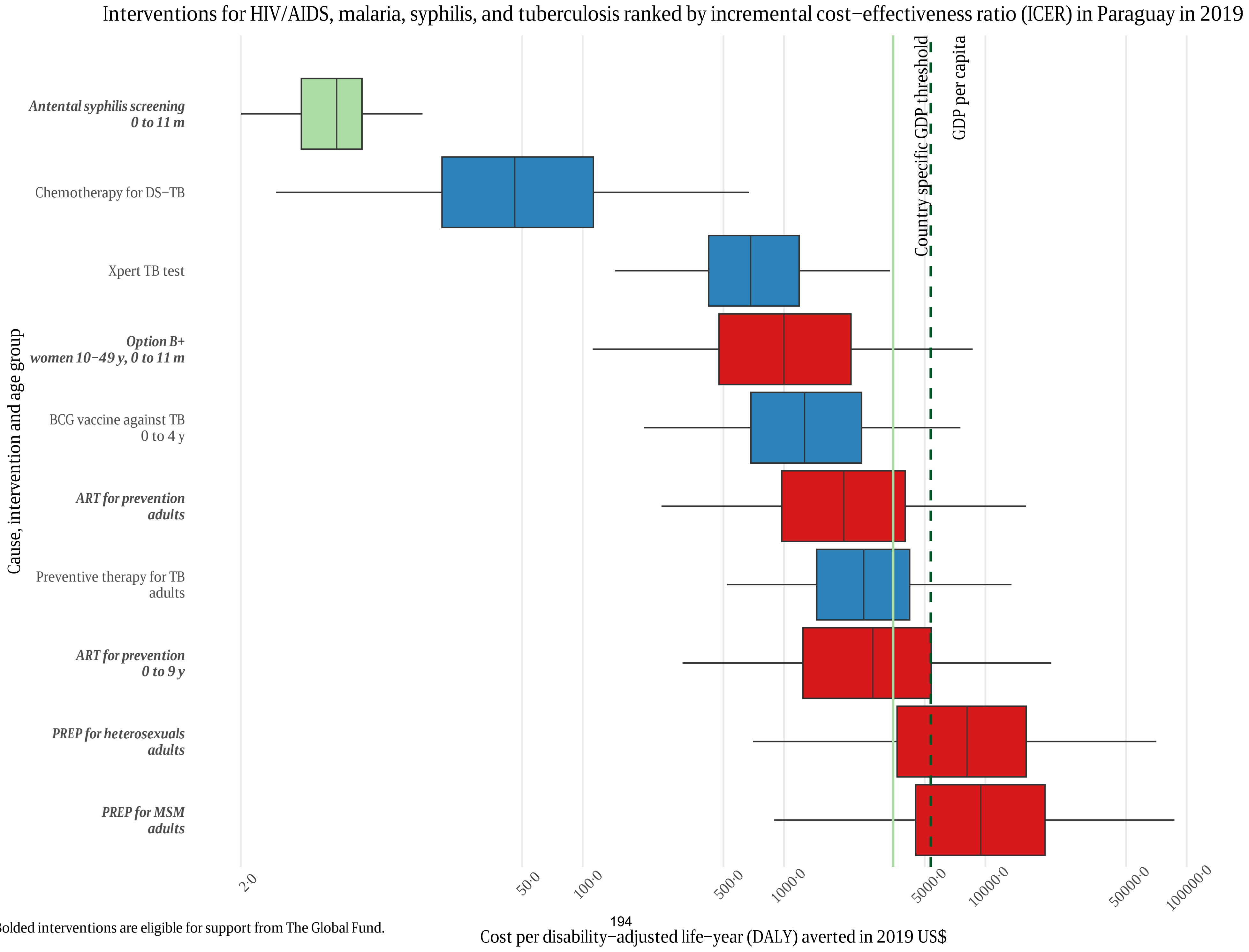

Bolded interventions are eligible for support from The Global Fund.

Interventions for HIV/AIDS, malaria, syphilis, and tuberculosis ranked by incremental cost−effectiveness ratio (ICER) in Peru in 2019

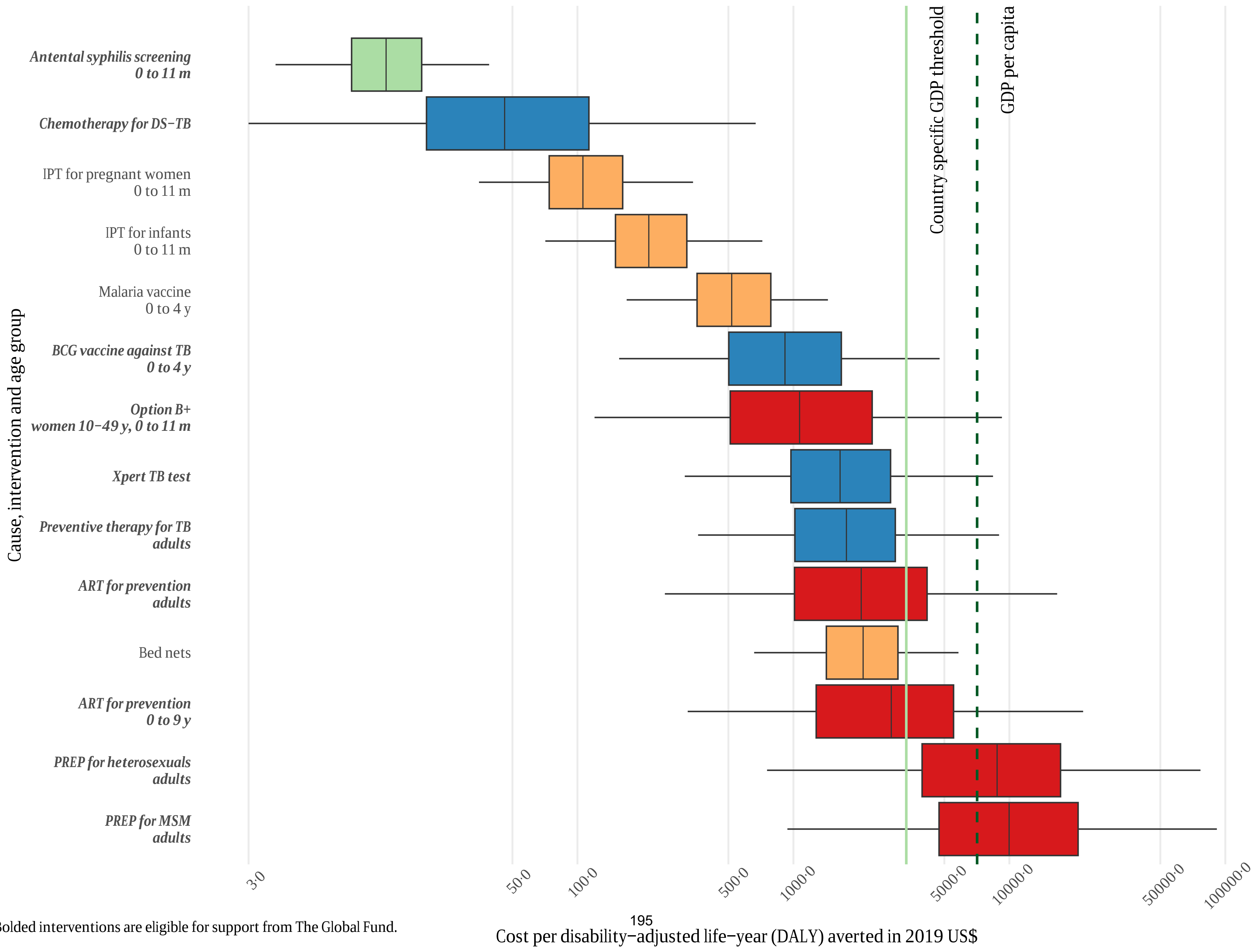

Bolded interventions are eligible for support from The Global Fund.

Interventions for HIV/AIDS, malaria, syphilis, and tuberculosis ranked by incremental cost−effectiveness ratio (ICER) in Philippines in 2019

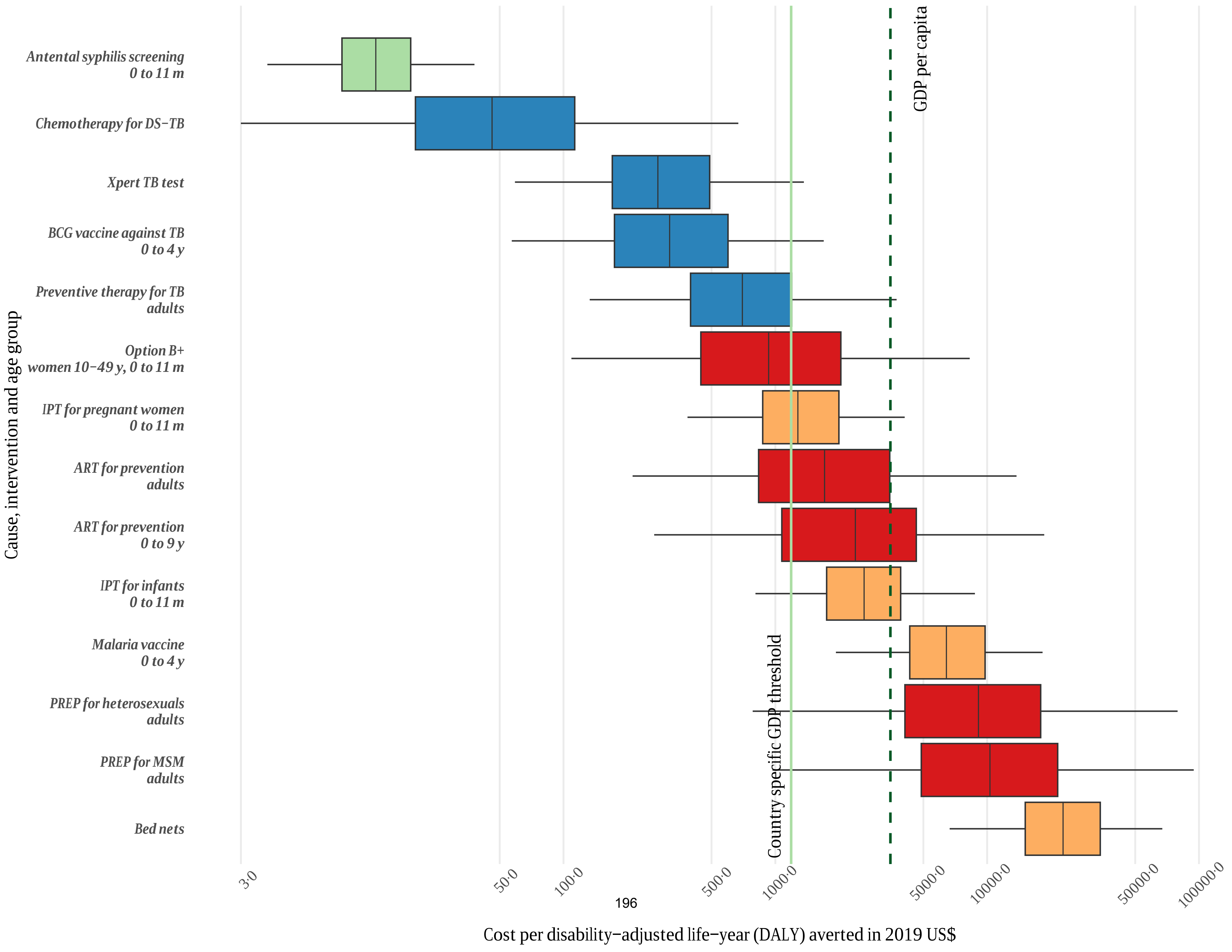

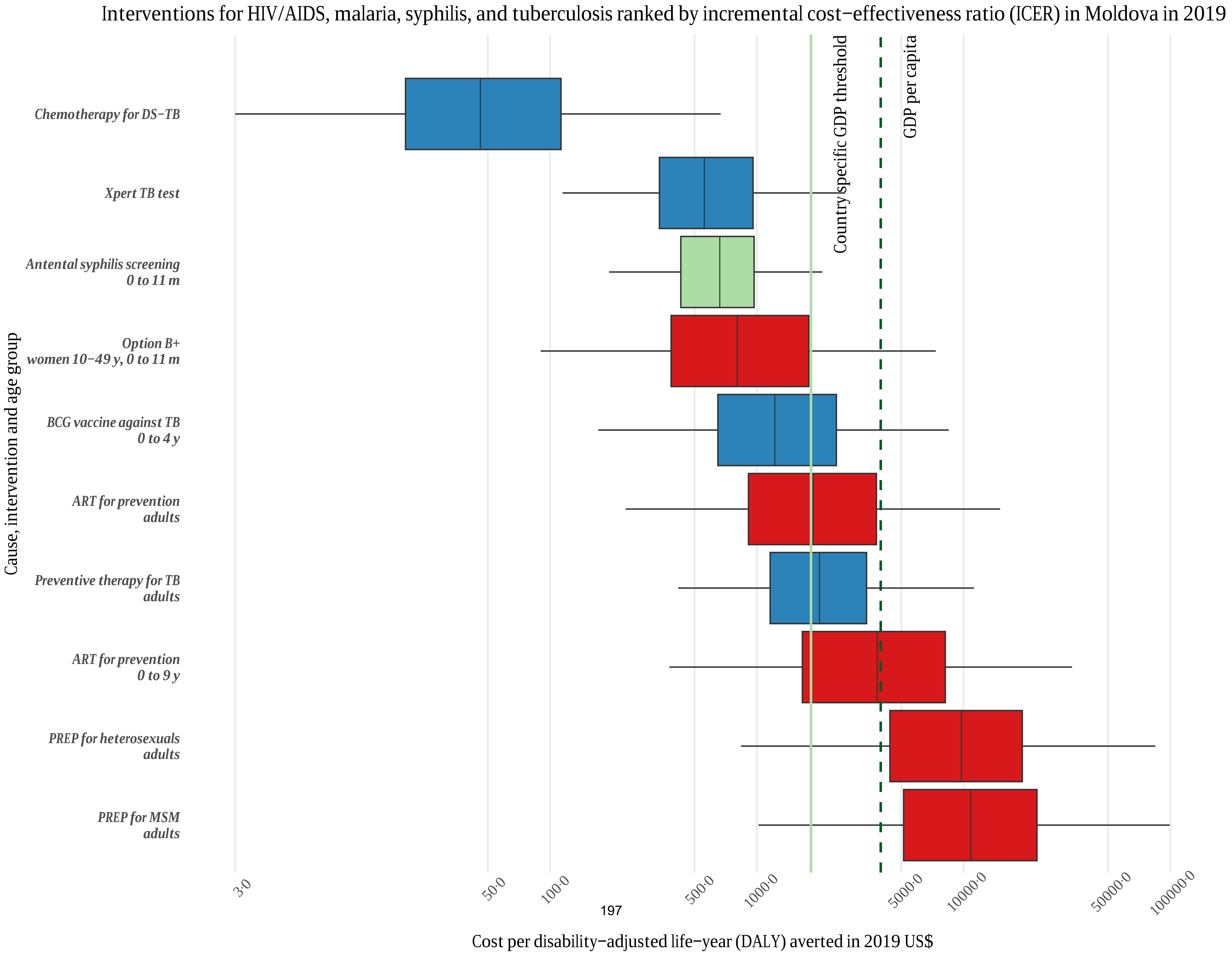
Interventions for HIV/AIDS, malaria, syphilis, and tuberculosis ranked by incremental cost−effectiveness ratio (ICER) in Moldova in 2019
Country specific GDP threshold
GDP per capita
Chemotherapy for DS−TB
Xpert TB test
Antental syphilis screening 0 to 11 m
Option B+ women 10−49 y, 0 to 11 m
BCG vaccine against TB 0 to 4 y
ART for prevention adults
Preventive therapy for TB adults
ART for prevention 0 to 9 y
PREP for heterosexuals adults
PREP for MSM adults
Cause, intervention and age group
3·0
50·0
100·0
500·0
1000·0
5000·0
10000·0
50000·0
100000·0
Cost per disability−adjusted life−year (DALY) averted in 2019 US$

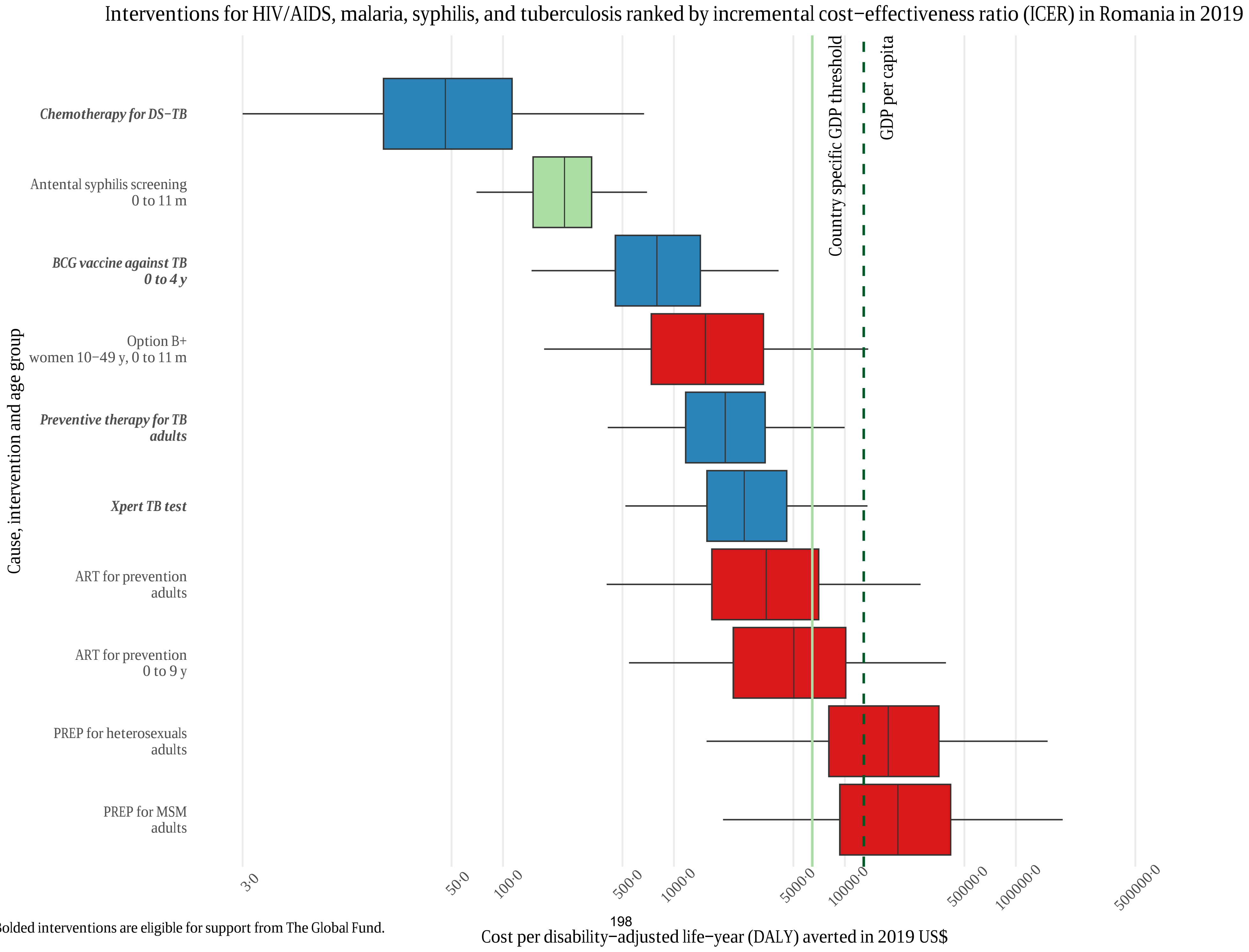

Bolded interventions are eligible for support from The Global Fund.

Interventions for HIV/AIDS, malaria, syphilis, and tuberculosis ranked by incremental cost−effectiveness ratio (ICER) in Russia in 2019

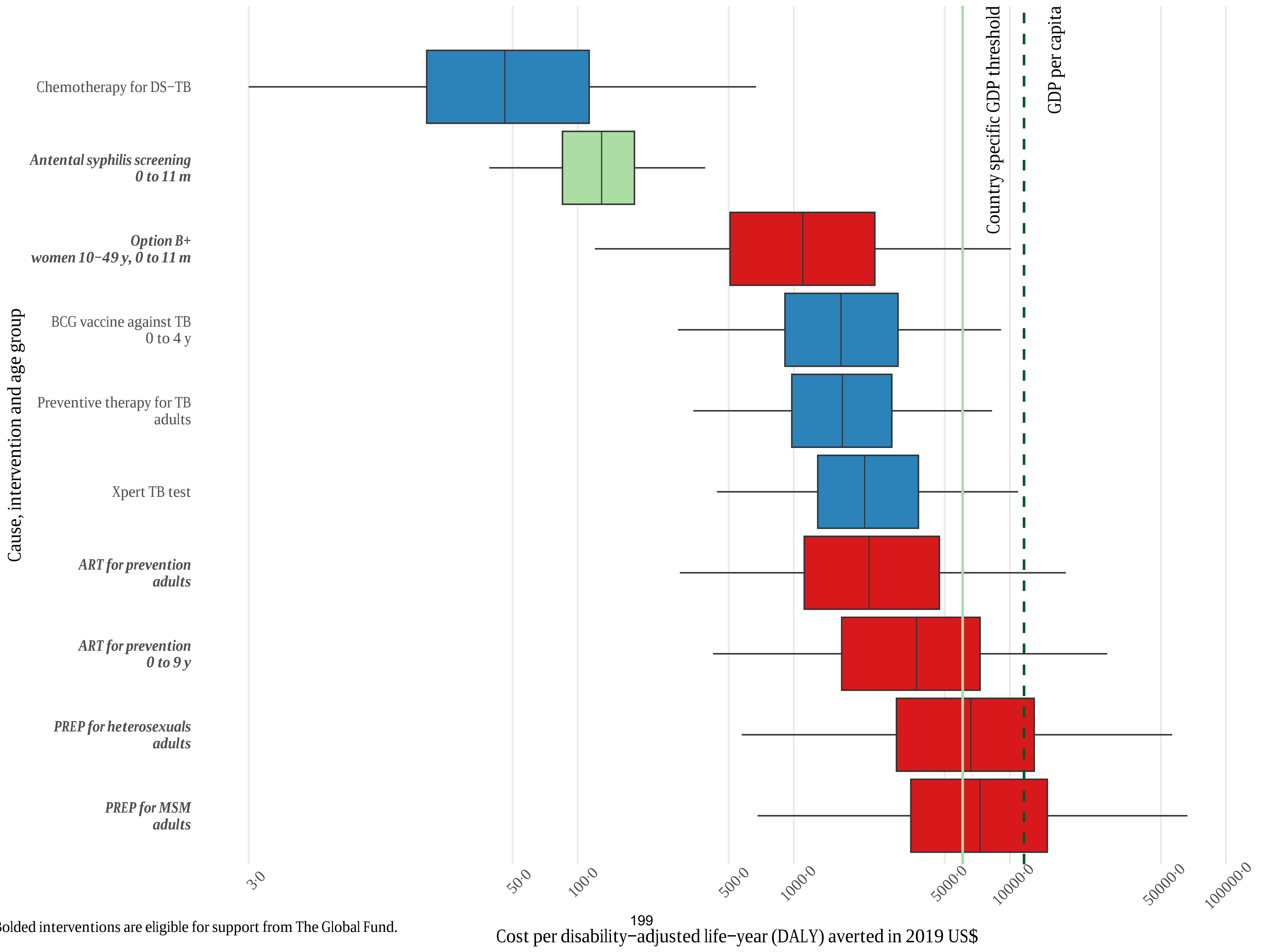

Bolded interventions are eligible for support from The Global Fund.

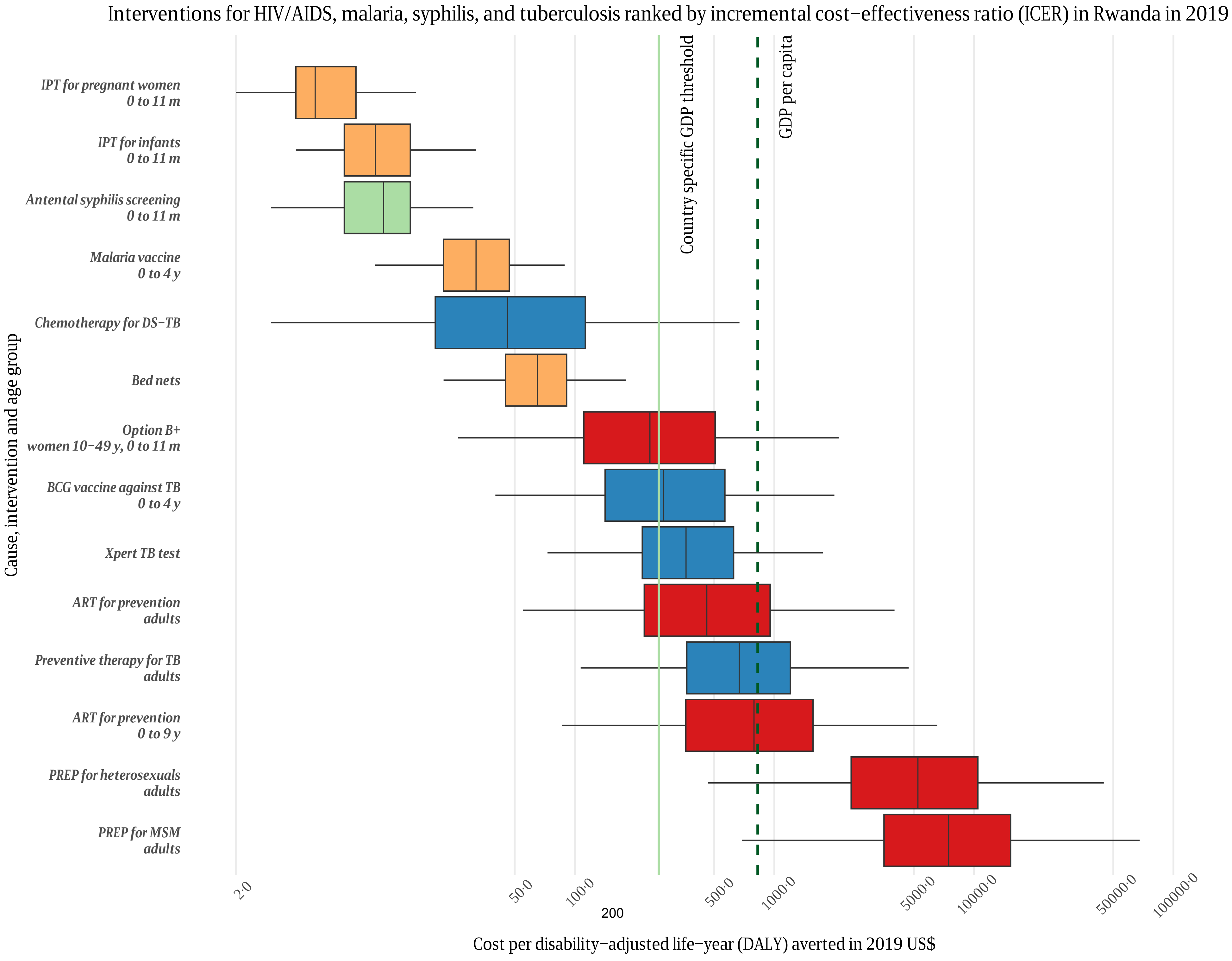
Interventions for HIV/AIDS, malaria, syphilis, and tuberculosis ranked by incremental cost−effectiveness ratio (ICER) in Rwanda in 2019
Country specific GDP threshold
GDP per capita
IPT for pregnant women
0 to 11 m
IPT for infants
0 to 11 m
Antental syphilis screening
0 to 11 m
Malaria vaccine
0 to 4 y
Chemotherapy for DS−TB
Bed nets
Option B+
women 10−49 y, 0 to 11 m
BCG vaccine against TB
0 to 4 y
Xpert TB test
ART for prevention
adults
Preventive therapy for TB
adults
ART for prevention
0 to 9 y
PREP for heterosexuals
adults
PREP for MSM
adults
Cause, intervention and age group
2·0
50·0
100·0
200
500·0
1000·0
5000·0
10000·0
50000·0
100000·0
Cost per disability−adjusted life−year (DALY) averted in 2019 US$

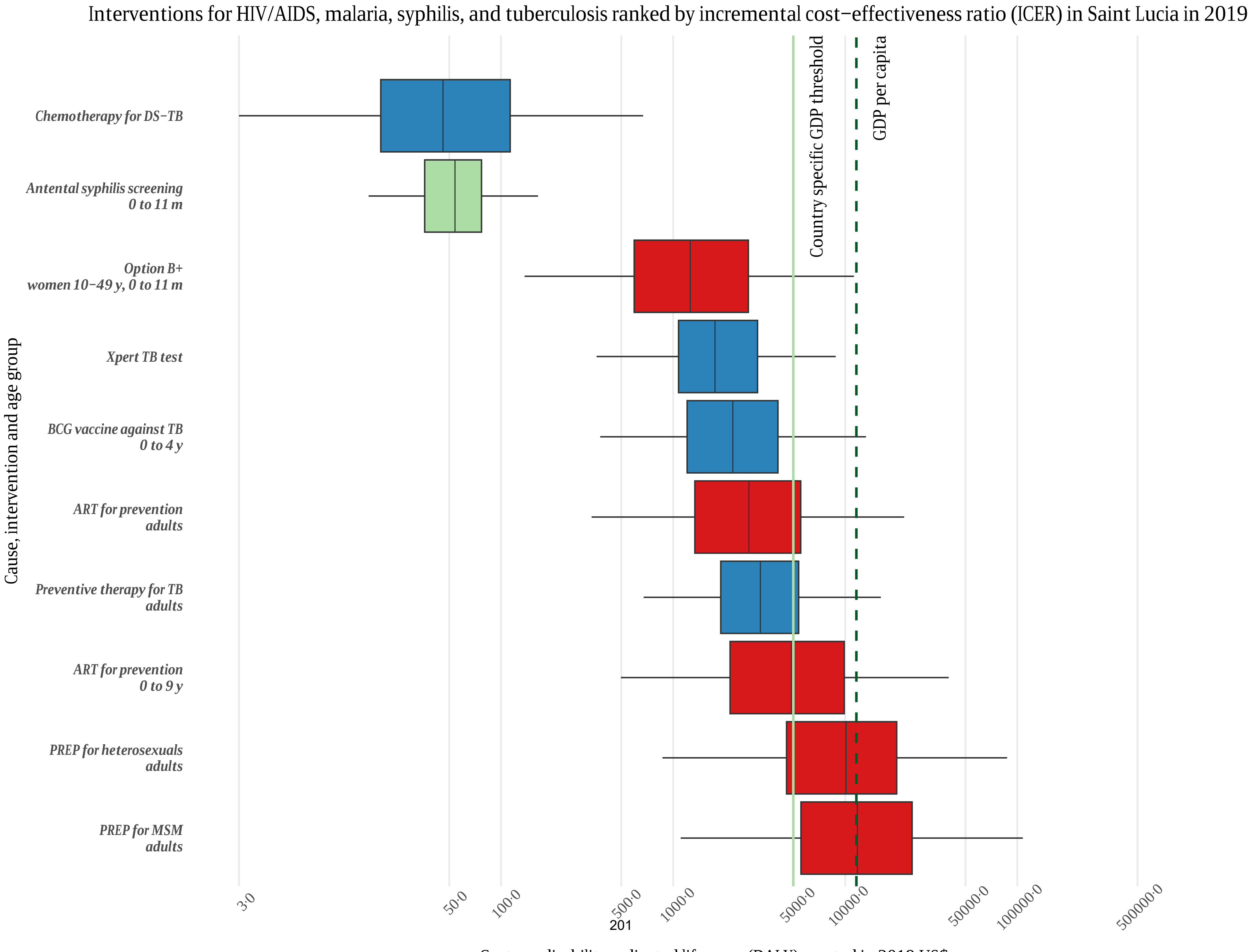
Interventions for HIV/AIDS, malaria, syphilis, and tuberculosis ranked by incremental cost−effectiveness ratio (ICER) in Saint Lucia in 2019
Country specific GDP threshold
GDP per capita
Cause, intervention and age group
Chemotherapy for DS−TB
Antental syphilis screening
0 to 11 m
Option B+
women 10−49 y, 0 to 11 m
Xpert TB test
BCG vaccine against TB
0 to 4 y
ART for prevention
adults
Preventive therapy for TB
adults
ART for prevention
0 to 9 y
PREP for heterosexuals
adults
PREP for MSM
adults
3·0
50·0
100·0
500·0
1000·0
5000·0
10000·0
50000·0
100000·0
500000·0
Cost per disability−adjusted life−year (DALY) averted in 2019 US$

erventions for HIV/AIDS, malaria, syphilis, and tuberculosis ranked by incremental cost−effectiveness ratio (ICER) in Saint Vincent and the Grenadines in 2019

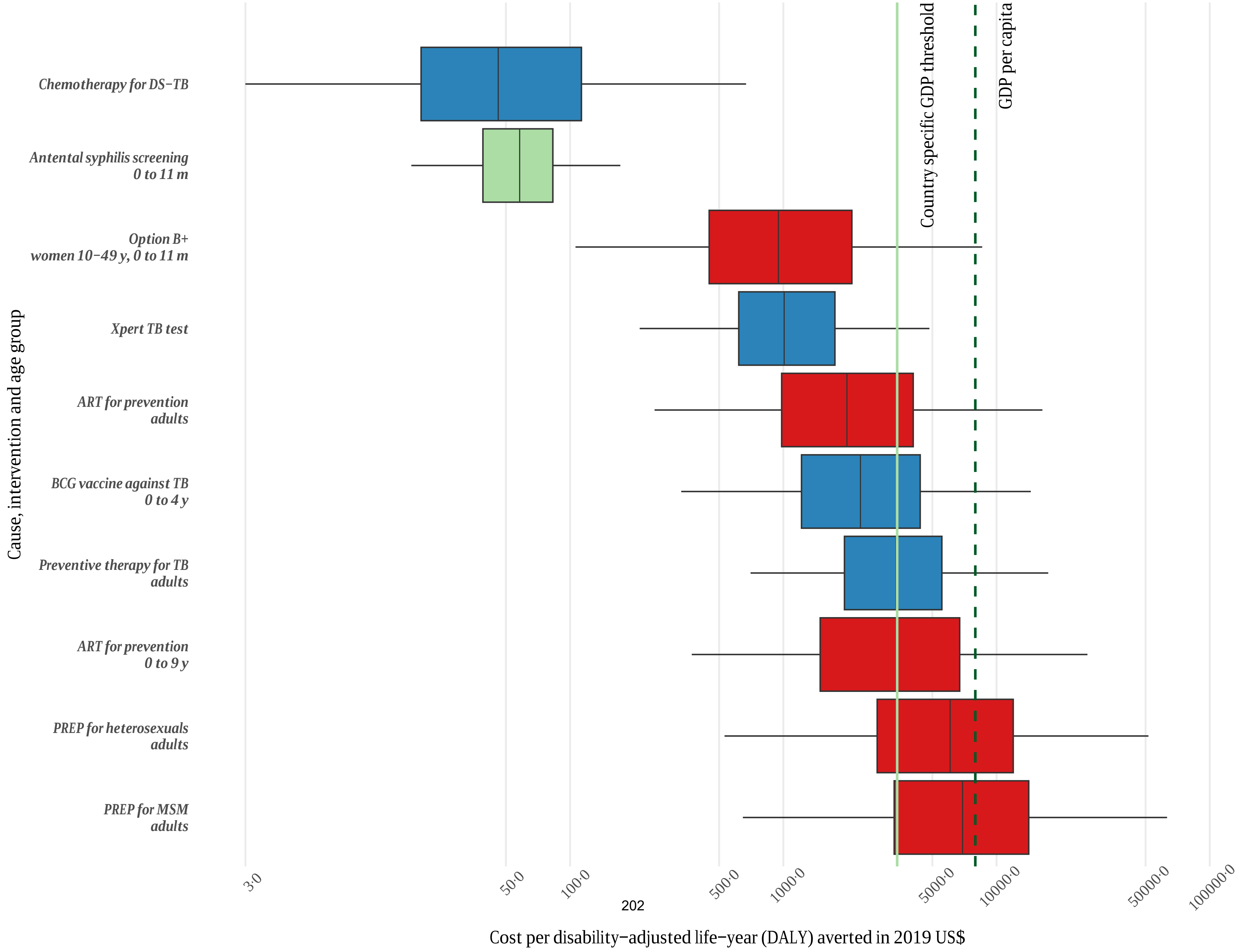

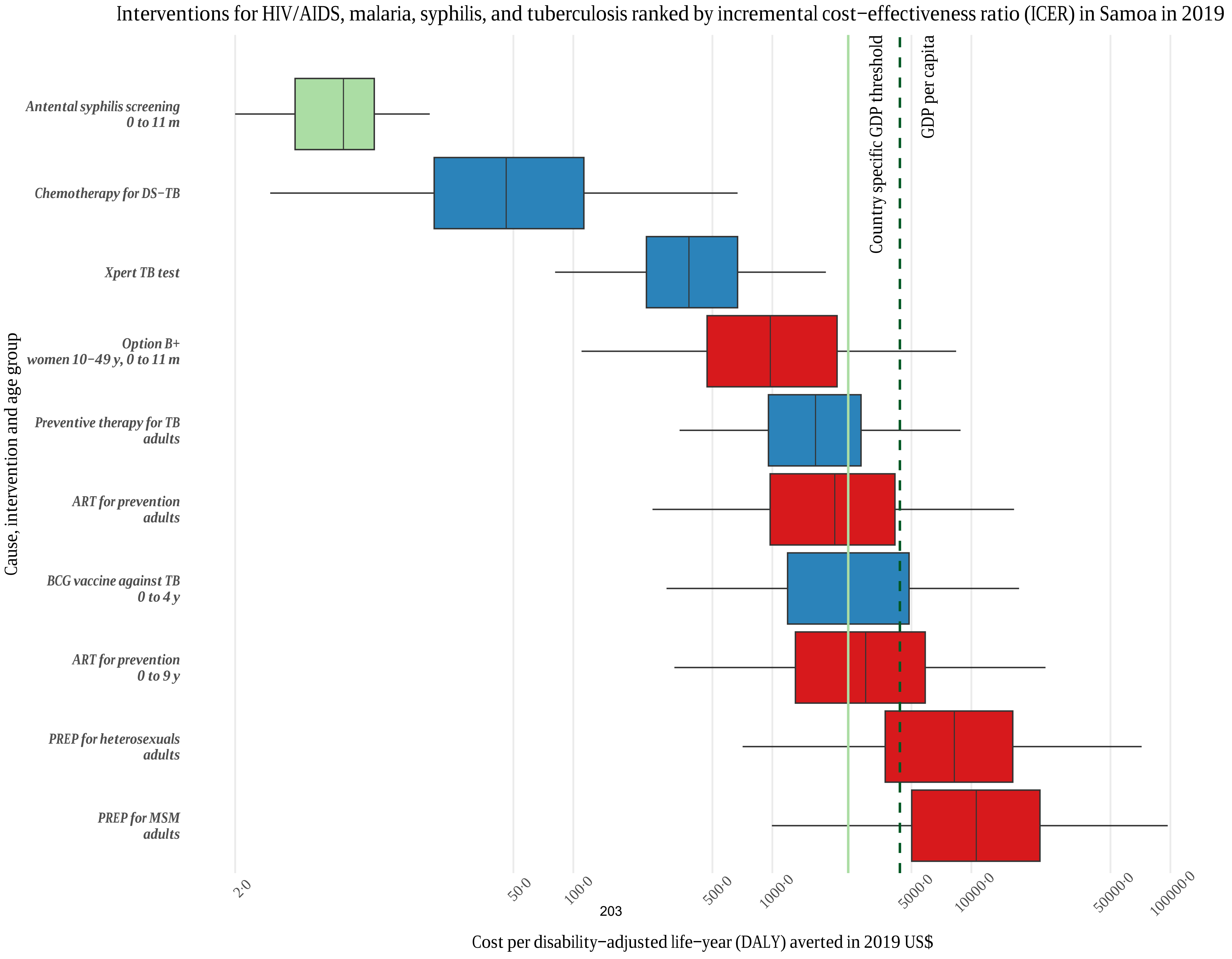
Interventions for HIV/AIDS, malaria, syphilis, and tuberculosis ranked by incremental cost−effectiveness ratio (ICER) in Samoa in 2019
Cause, intervention and age group
Antental syphilis screening
0 to 11 m
Chemotherapy for DS−TB
Xpert TB test
Option B+
women 10−49 y, 0 to 11 m
Preventive therapy for TB
adults
ART for prevention
adults
BCG vaccine against TB
0 to 4 y
ART for prevention
0 to 9 y
PREP for heterosexuals
adults
PREP for MSM
adults
Country specific GDP threshold
GDP per capita
2·0
50·0
100·0
500·0
1000·0
5000·0
10000·0
50000·0
100000·0
Cost per disability−adjusted life−year (DALY) averted in 2019 US$

Interventions for HIV/AIDS, malaria, syphilis, and tuberculosis ranked by incremental cost−effectiveness ratio (ICER) in São Tomé and PrÍncipe in 2019

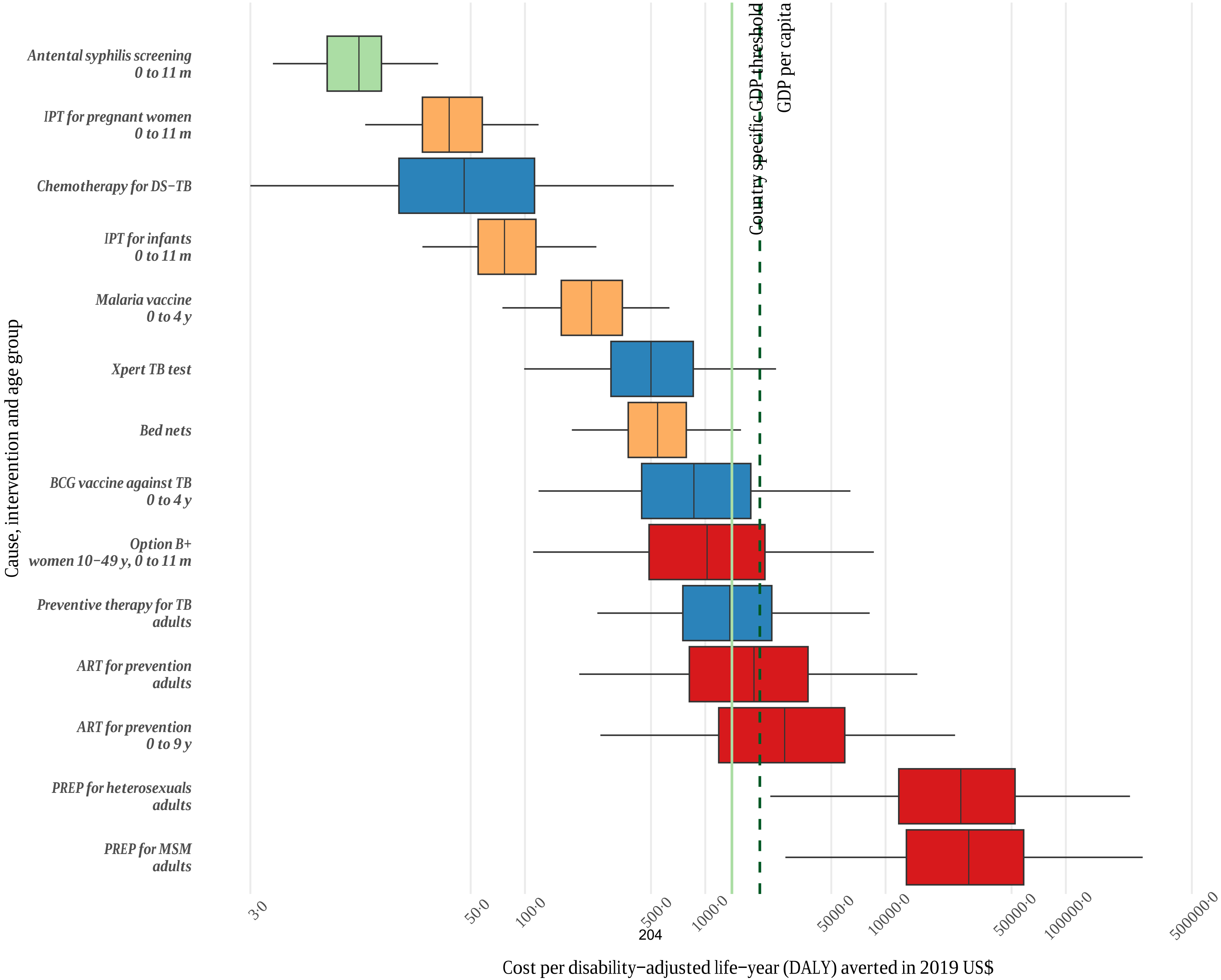

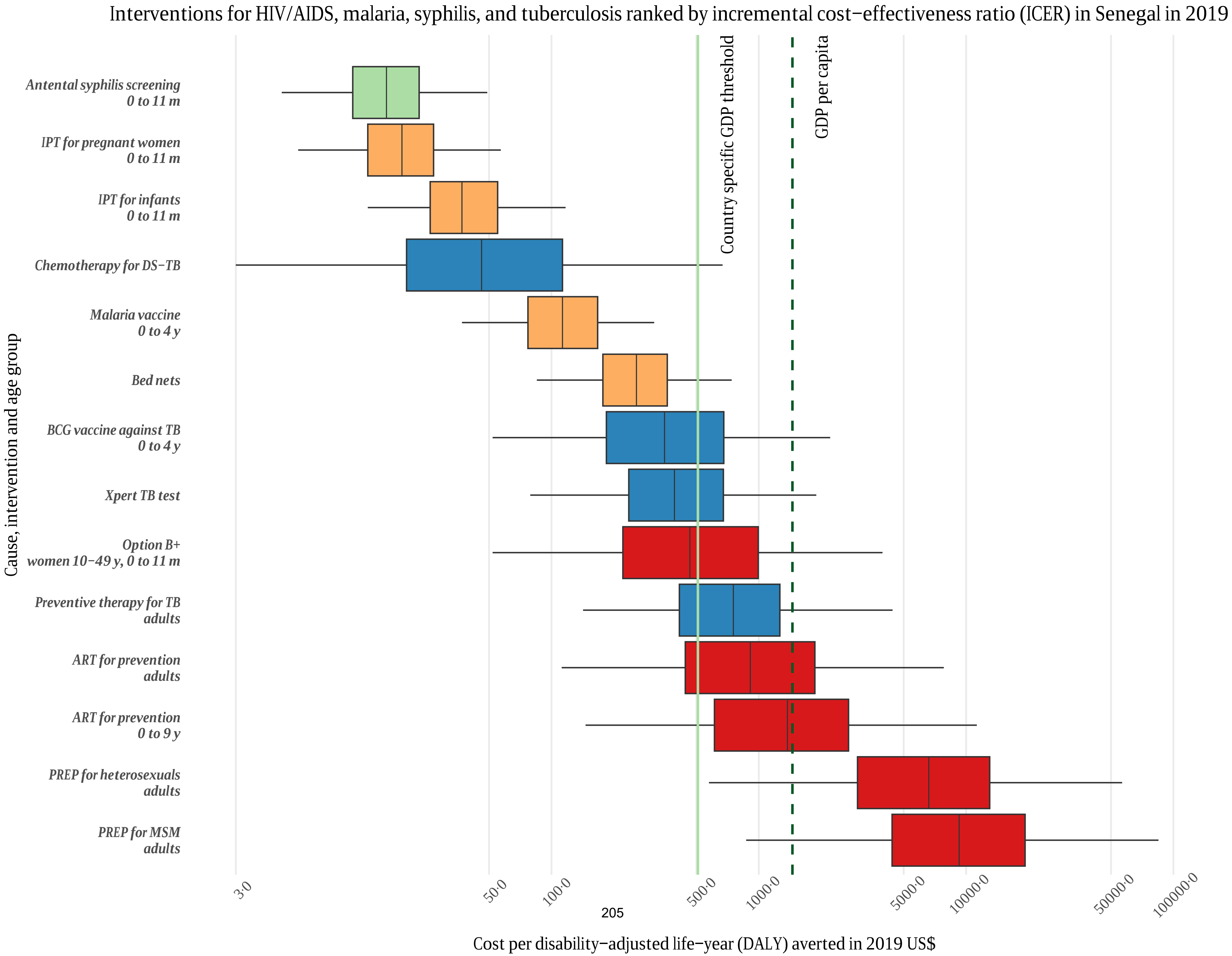

Interventions for HIV/AIDS, malaria, syphilis, and tuberculosis ranked by incremental cost−effectiveness ratio (ICER) in Senegal in 2019
Country specific GDP threshold
GDP per capita
Antental syphilis screening
0 to 11 m
IPT for pregnant women
0 to 11 m
IPT for infants
0 to 11 m
Chemotherapy for DS−TB
Malaria vaccine
0 to 4 y
Bed nets
BCG vaccine against TB
0 to 4 y
Xpert TB test
Option B+
women 10−49 y, 0 to 11 m
Preventive therapy for TB
adults
ART for prevention
adults
ART for prevention
0 to 9 y
PREP for heterosexuals
adults
PREP for MSM
adults
Cause, intervention and age group
3·0
50·0
100·0
500·0
1000·0
5000·0
10000·0
50000·0
100000·0
Cost per disability−adjusted life−year (DALY) averted in 2019 US$

Interventions for HIV/AIDS, malaria, syphilis, and tuberculosis ranked by incremental cost−effectiveness ratio (ICER) in Serbia in 2019

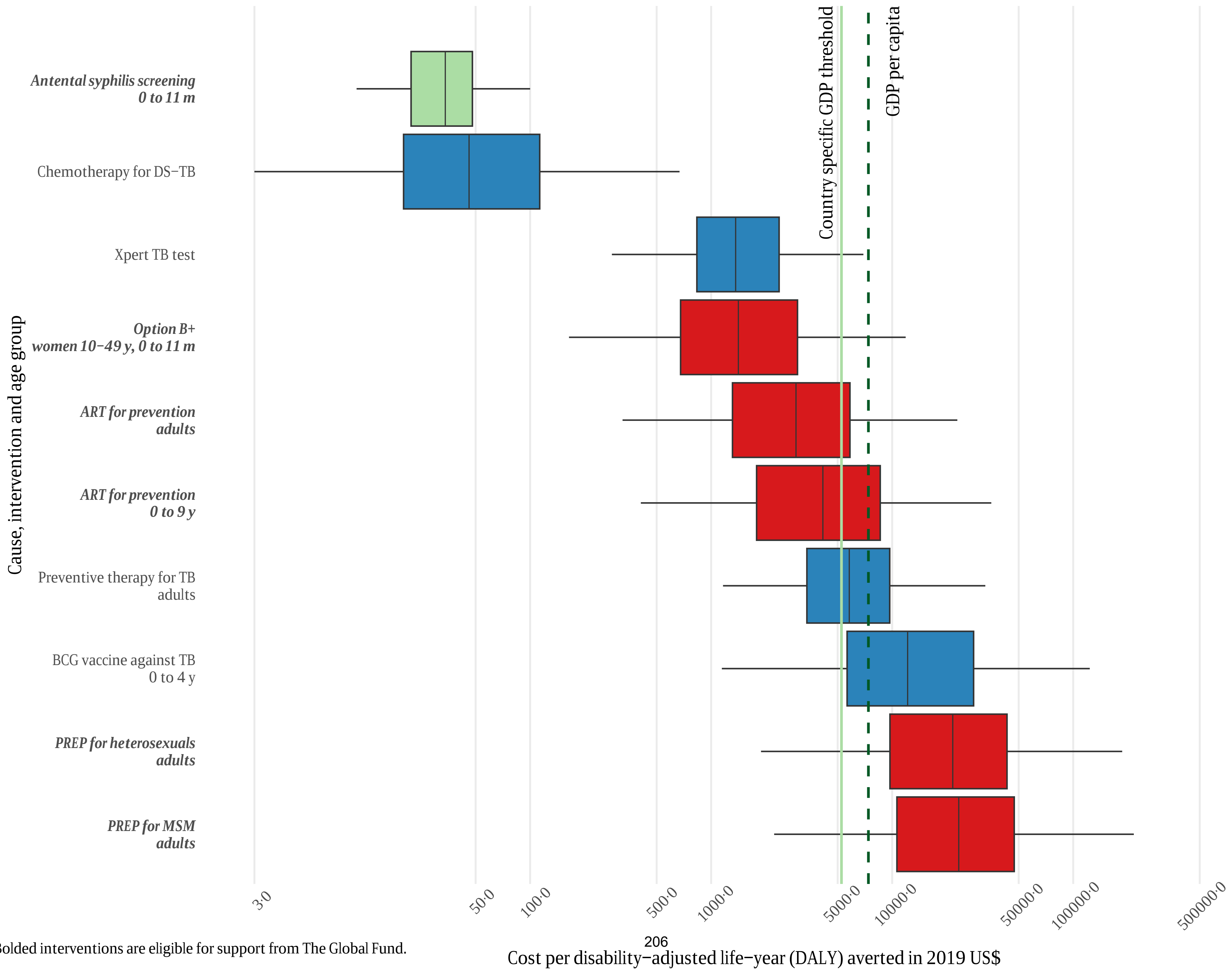

Bolded interventions are eligible for support from The Global Fund.

Interventions for HIV/AIDS, malaria, syphilis, and tuberculosis ranked by incremental cost−effectiveness ratio (ICER) in Sierra Leone in 2019

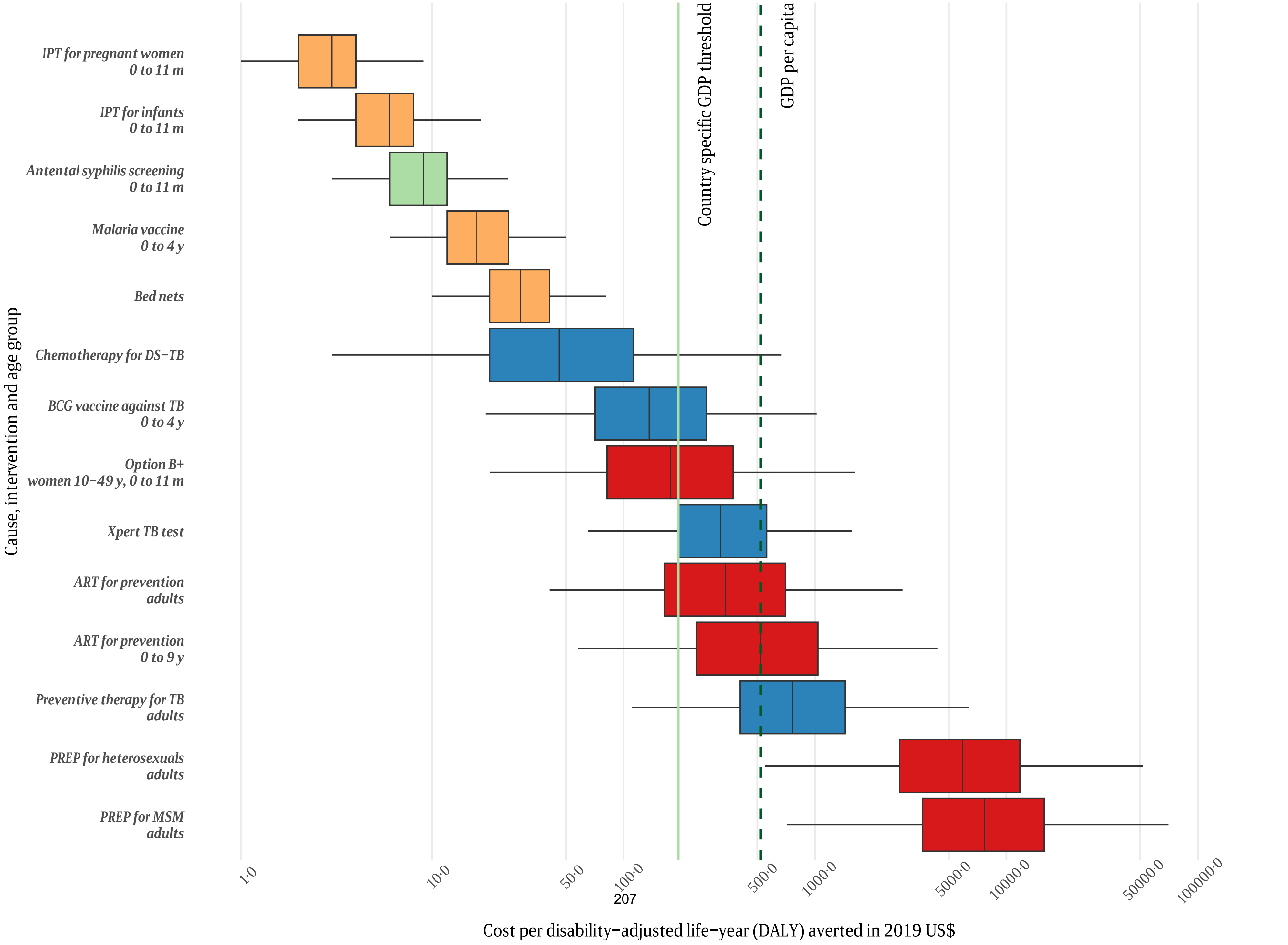

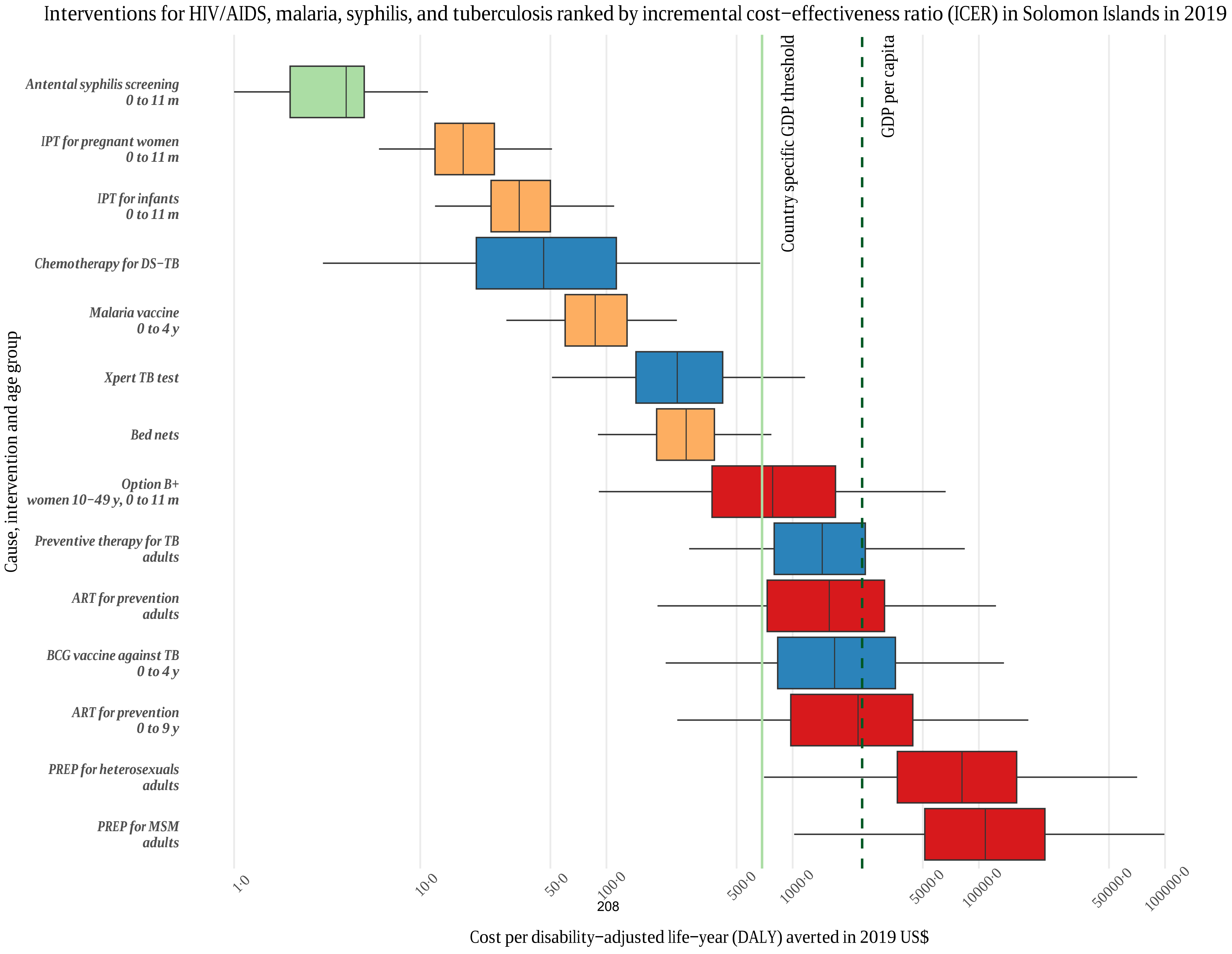
Interventions for HIV/AIDS, malaria, syphilis, and tuberculosis ranked by incremental cost−effectiveness ratio (ICER) in Solomon Islands in 2019
Antental syphilis screening
0 to 11 m
IPT for pregnant women
0 to 11 m
IPT for infants
0 to 11 m
Chemotherapy for DS−TB
Malaria vaccine
0 to 4 y
Xpert TB test
Bed nets
Option B+
women 10−49 y, 0 to 11 m
Preventive therapy for TB
adults
ART for prevention
adults
BCG vaccine against TB
0 to 4 y
ART for prevention
0 to 9 y
PREP for heterosexuals
adults
PREP for MSM
adults
Country specific GDP threshold
GDP per capita
Cause, intervention and age group
1·0
10·0
50·0
100·0
500·0
1000·0
5000·0
10000·0
50000·0
100000·0
Cost per disability−adjusted life−year (DALY) averted in 2019 US$

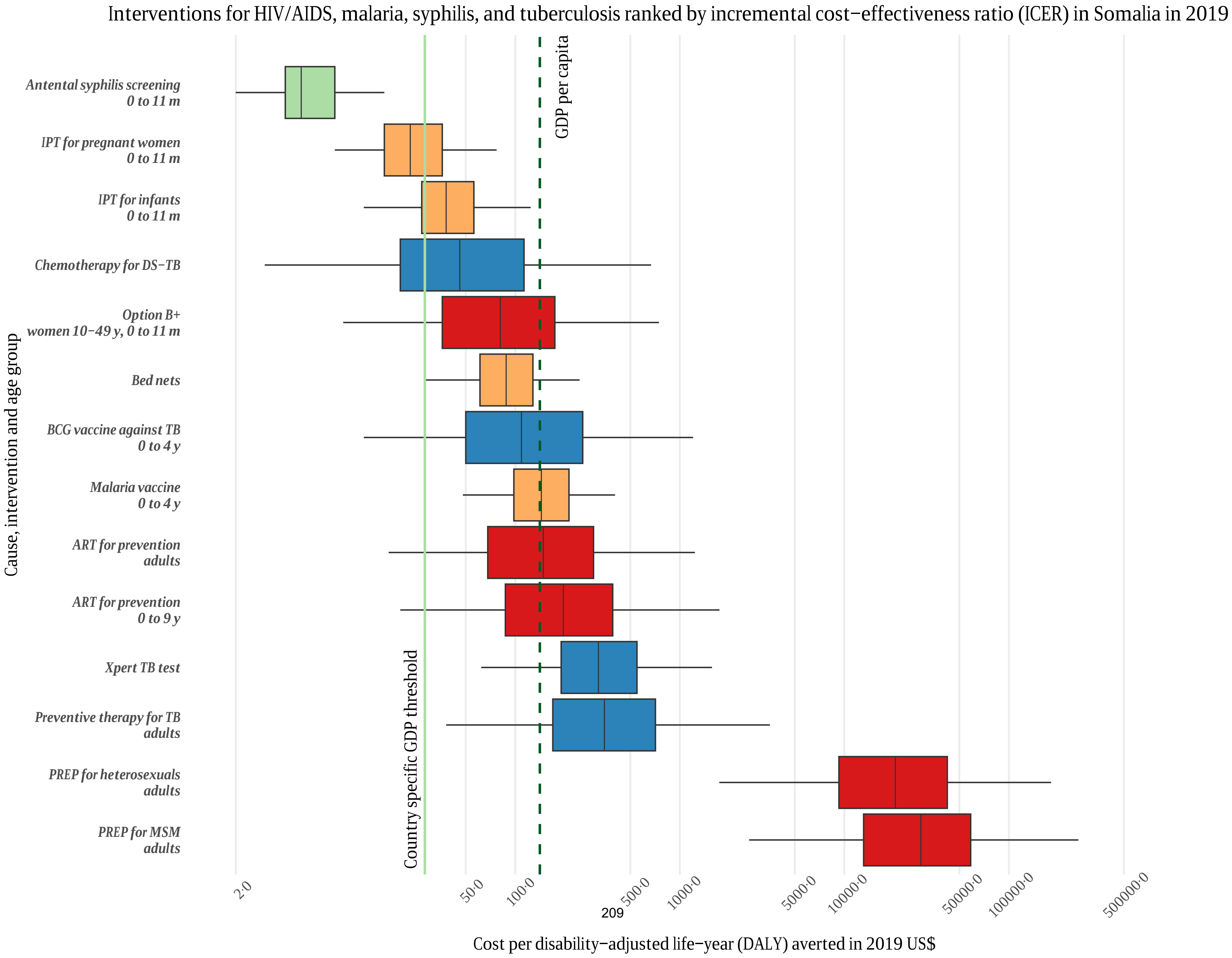

Interventions for HIV/AIDS, malaria, syphilis, and tuberculosis ranked by incremental cost−effectiveness ratio (ICER) in Somalia in 2019
Cause, intervention and age group
Antental syphilis screening 0 to 11 m
IPT for pregnant women 0 to 11 m
IPT for infants 0 to 11 m
Chemotherapy for DS−TB
Option B+ women 10−49 y, 0 to 11 m
Bed nets
BCG vaccine against TB 0 to 4 y
Malaria vaccine 0 to 4 y
ART for prevention adults
ART for prevention 0 to 9 y
Xpert TB test
Preventive therapy for TB adults
PREP for heterosexuals adults
PREP for MSM adults
GDP per capita
Country specific GDP threshold
2·0
50·0
100·0
500·0
1000·0
5000·0
10000·0
50000·0
100000·0
500000·0
Cost per disability−adjusted life−year (DALY) averted in 2019 US$

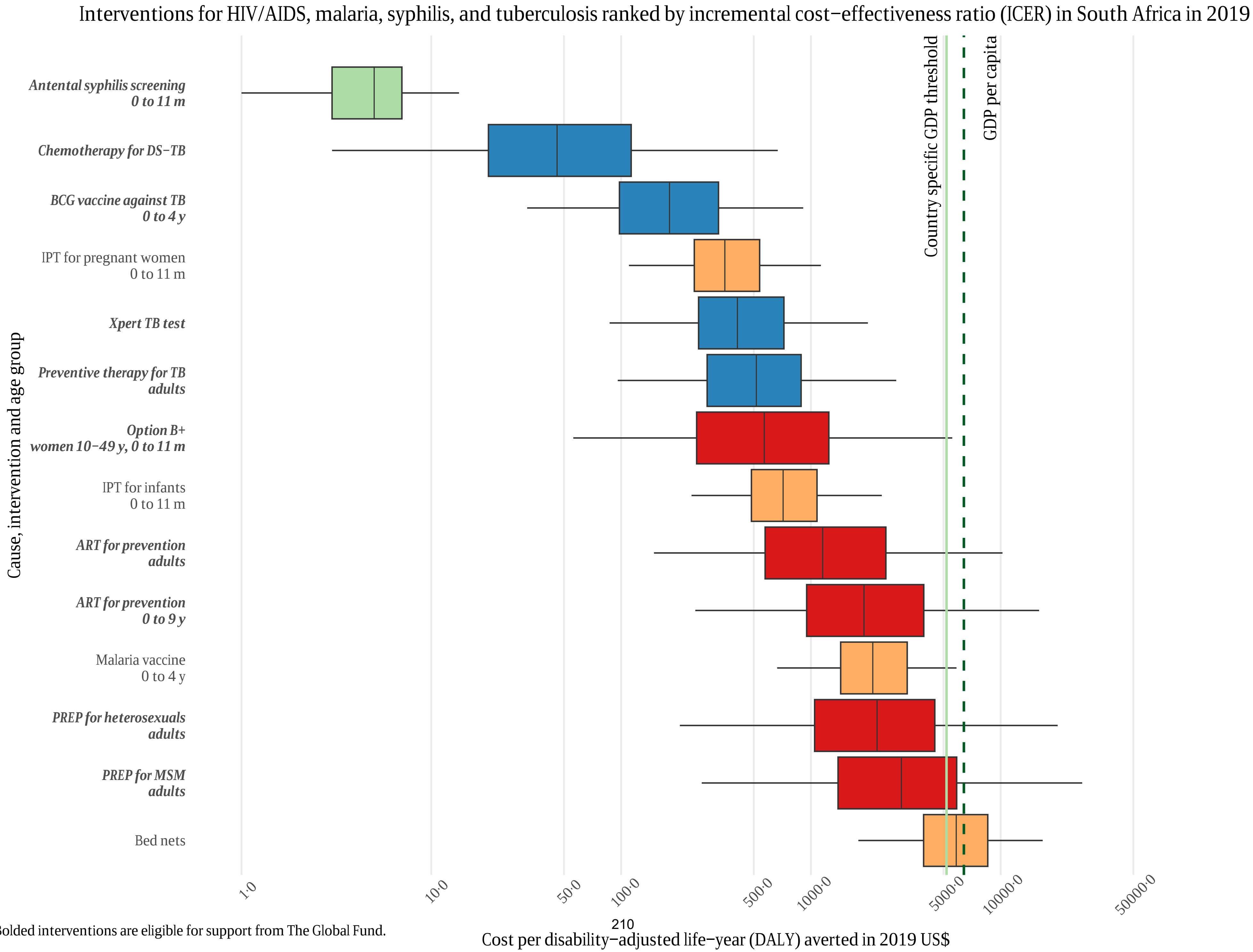

Bolded interventions are eligible for support from The Global Fund.

Interventions for HIV/AIDS, malaria, syphilis, and tuberculosis ranked by incremental cost−effectiveness ratio (ICER) in South Sudan in 2019

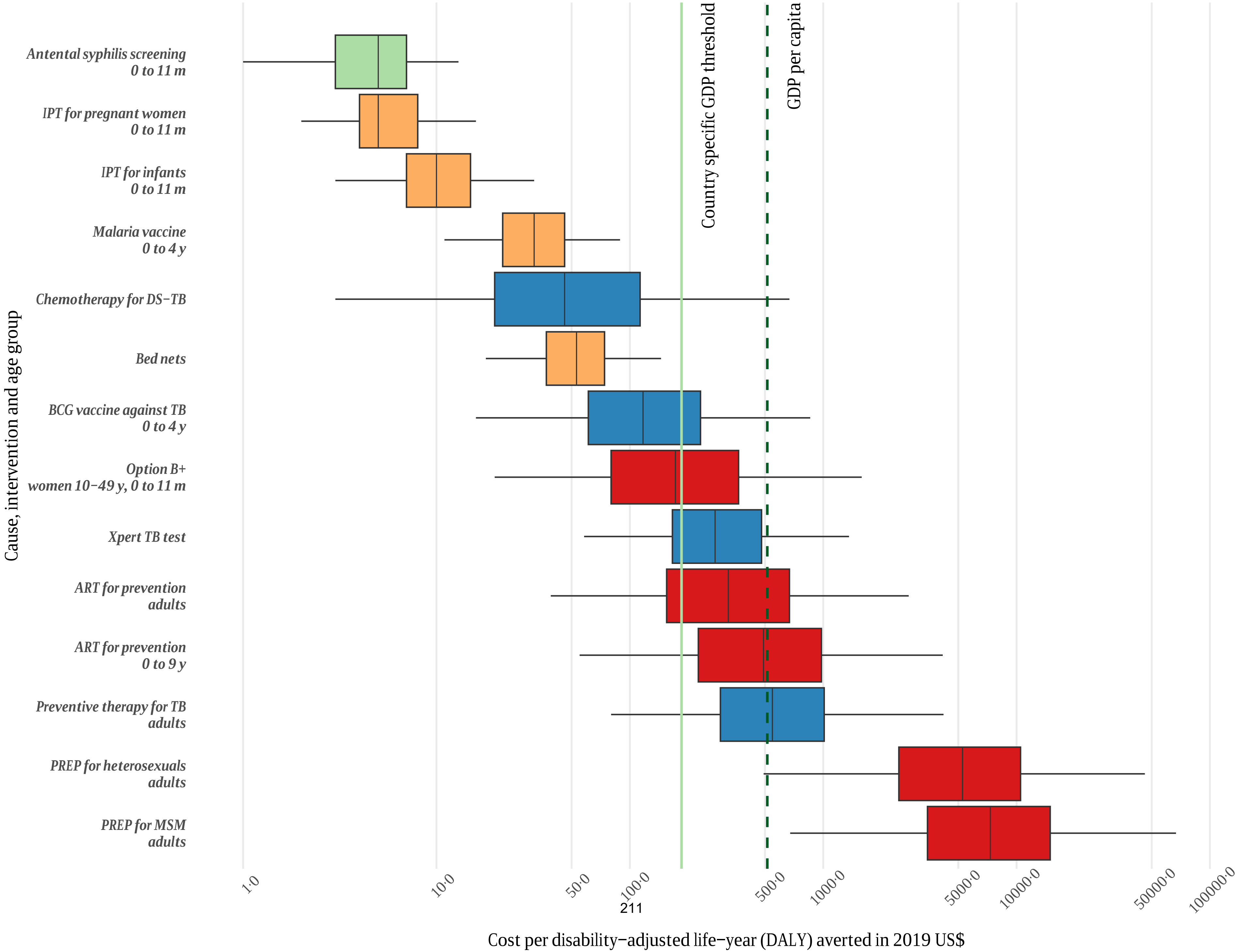

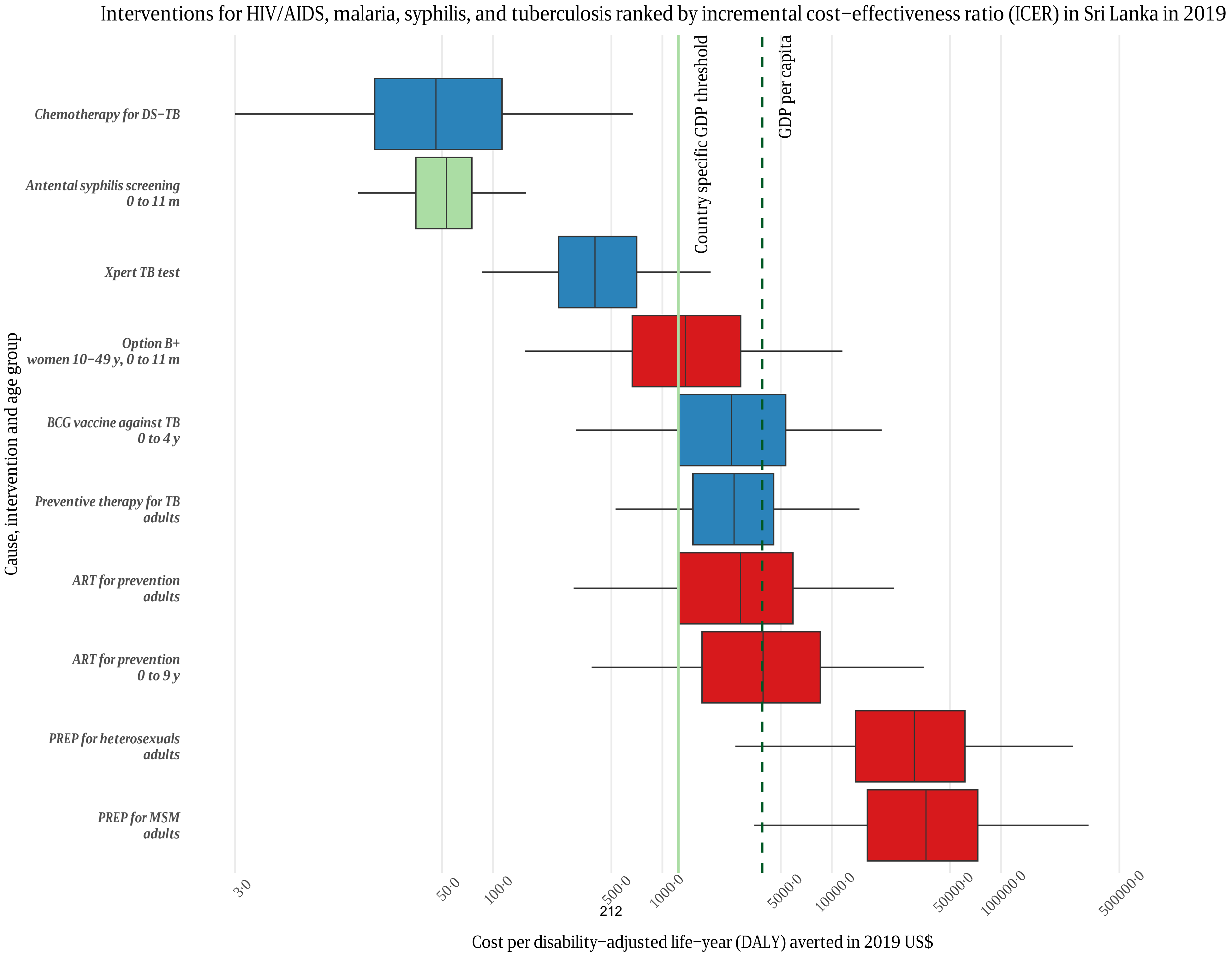
Interventions for HIV/AIDS, malaria, syphilis, and tuberculosis ranked by incremental cost−effectiveness ratio (ICER) in Sri Lanka in 2019
Country specific GDP threshold
GDP per capita
Chemotherapy for DS−TB
Antental syphilis screening
0 to 11 m
Xpert TB test
Option B+
women 10−49 y, 0 to 11 m
BCG vaccine against TB
0 to 4 y
Preventive therapy for TB
adults
ART for prevention
adults
ART for prevention
0 to 9 y
PREP for heterosexuals
adults
PREP for MSM
adults
Cause, intervention and age group
3·0
50·0
100·0
500·0
1000·0
5000·0
10000·0
50000·0
100000·0
500000·0
Cost per disability−adjusted life−year (DALY) averted in 2019 US$

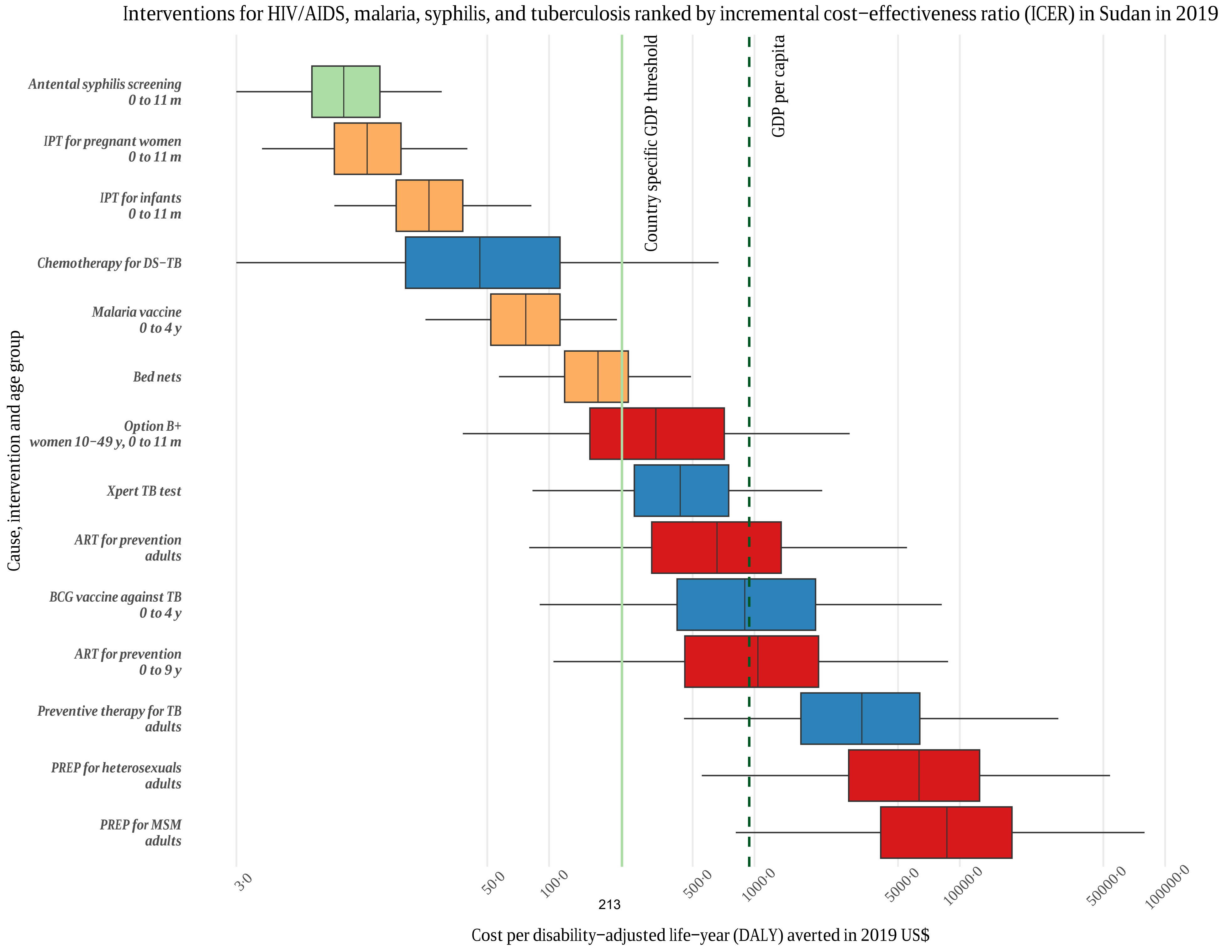
Interventions for HIV/AIDS, malaria, syphilis, and tuberculosis ranked by incremental cost−effectiveness ratio (ICER) in Sudan in 2019
Country specific GDP threshold
GDP per capita
Cause, intervention and age group
Antental syphilis screening 0 to 11 m
IPT for pregnant women 0 to 11 m
IPT for infants 0 to 11 m
Chemotherapy for DS−TB
Malaria vaccine 0 to 4 y
Bed nets
Option B+ women 10−49 y, 0 to 11 m
Xpert TB test
ART for prevention adults
BCG vaccine against TB 0 to 4 y
ART for prevention 0 to 9 y
Preventive therapy for TB adults
PREP for heterosexuals adults
PREP for MSM adults
3·0
50·0
100·0
213
500·0
1000·0
5000·0
10000·0
50000·0
100000·0
Cost per disability−adjusted life−year (DALY) averted in 2019 US$

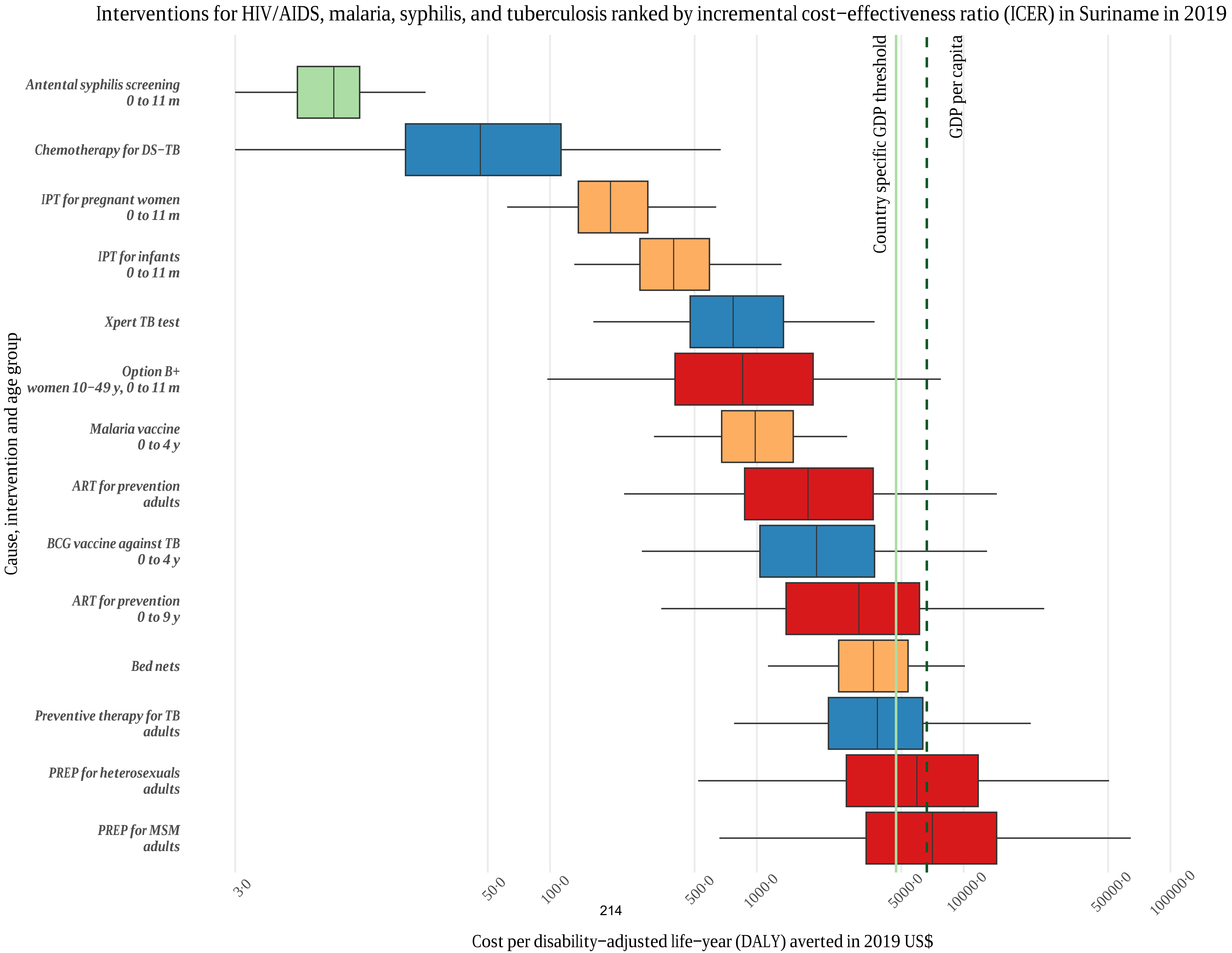
Interventions for HIV/AIDS, malaria, syphilis, and tuberculosis ranked by incremental cost−effectiveness ratio (ICER) in Suriname in 2019
Antental syphilis screening 0 to 11 m
Chemotherapy for DS−TB
IPT for pregnant women 0 to 11 m
IPT for infants 0 to 11 m
Xpert TB test
Option B+ women 10−49 y, 0 to 11 m
Malaria vaccine 0 to 4 y
ART for prevention adults
BCG vaccine against TB 0 to 4 y
ART for prevention 0 to 9 y
Bed nets
Preventive therapy for TB adults
PREP for heterosexuals adults
PREP for MSM adults
Cause, intervention and age group
Country specific GDP threshold
GDP per capita
3·0
50·0
100·0
500·0
1000·0
5000·0
10000·0
50000·0
100000·0
Cost per disability−adjusted life−year (DALY) averted in 2019 US$

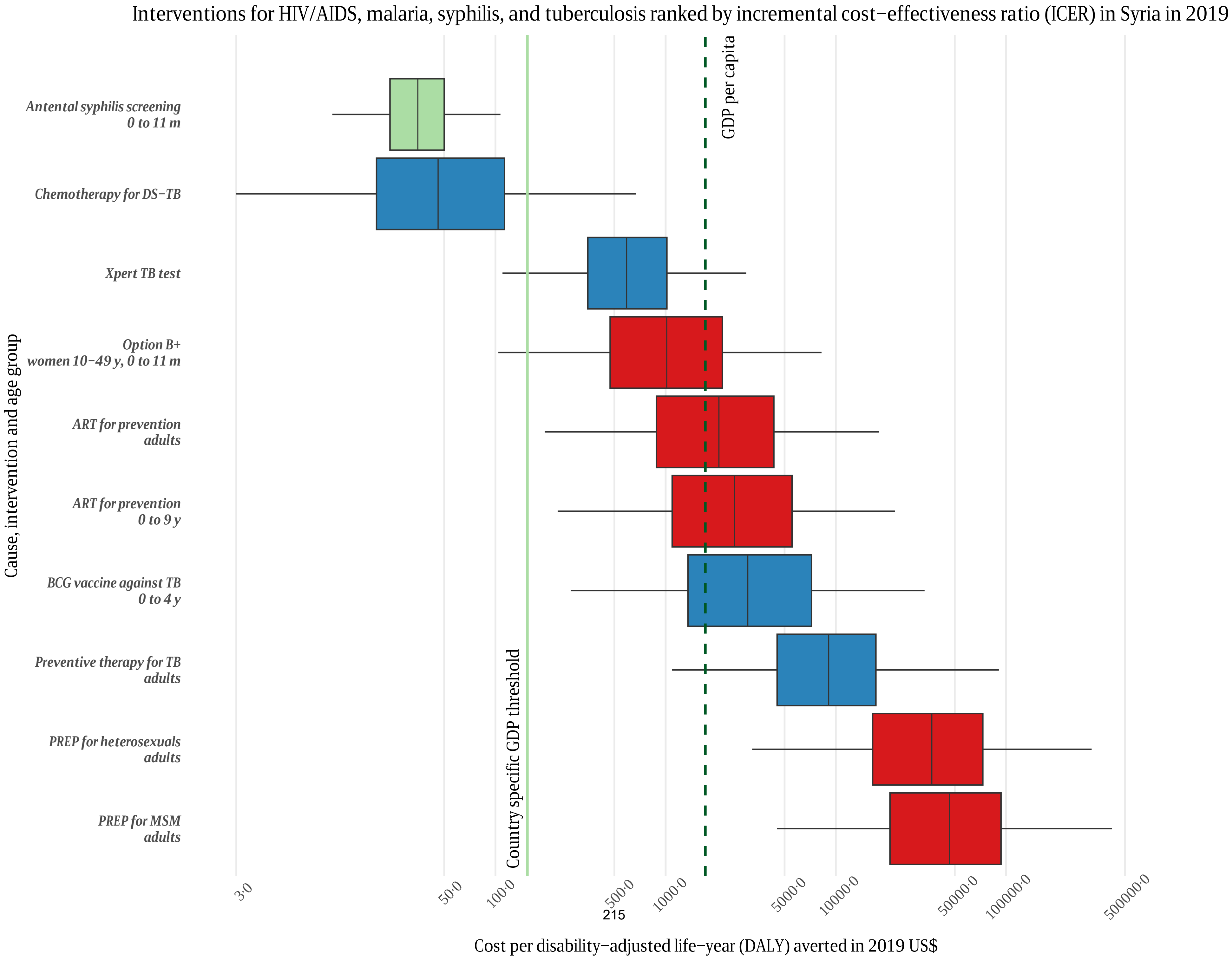

Interventions for HIV/AIDS, malaria, syphilis, and tuberculosis ranked by incremental cost−effectiveness ratio (ICER) in Syria in 2019
Cause, intervention and age group
Antental syphilis screening
0 to 11 m
Chemotherapy for DS−TB
Xpert TB test
Option B+
women 10−49 y, 0 to 11 m
ART for prevention
adults
ART for prevention
0 to 9 y
BCG vaccine against TB
0 to 4 y
Preventive therapy for TB
adults
PREP for heterosexuals
adults
PREP for MSM
adults
GDP per capita
Country specific GDP threshold
3·0
50·0
100·0
500·0
215
1000·0
5000·0
10000·0
50000·0
100000·0
500000·0
Cost per disability−adjusted life−year (DALY) averted in 2019 US$

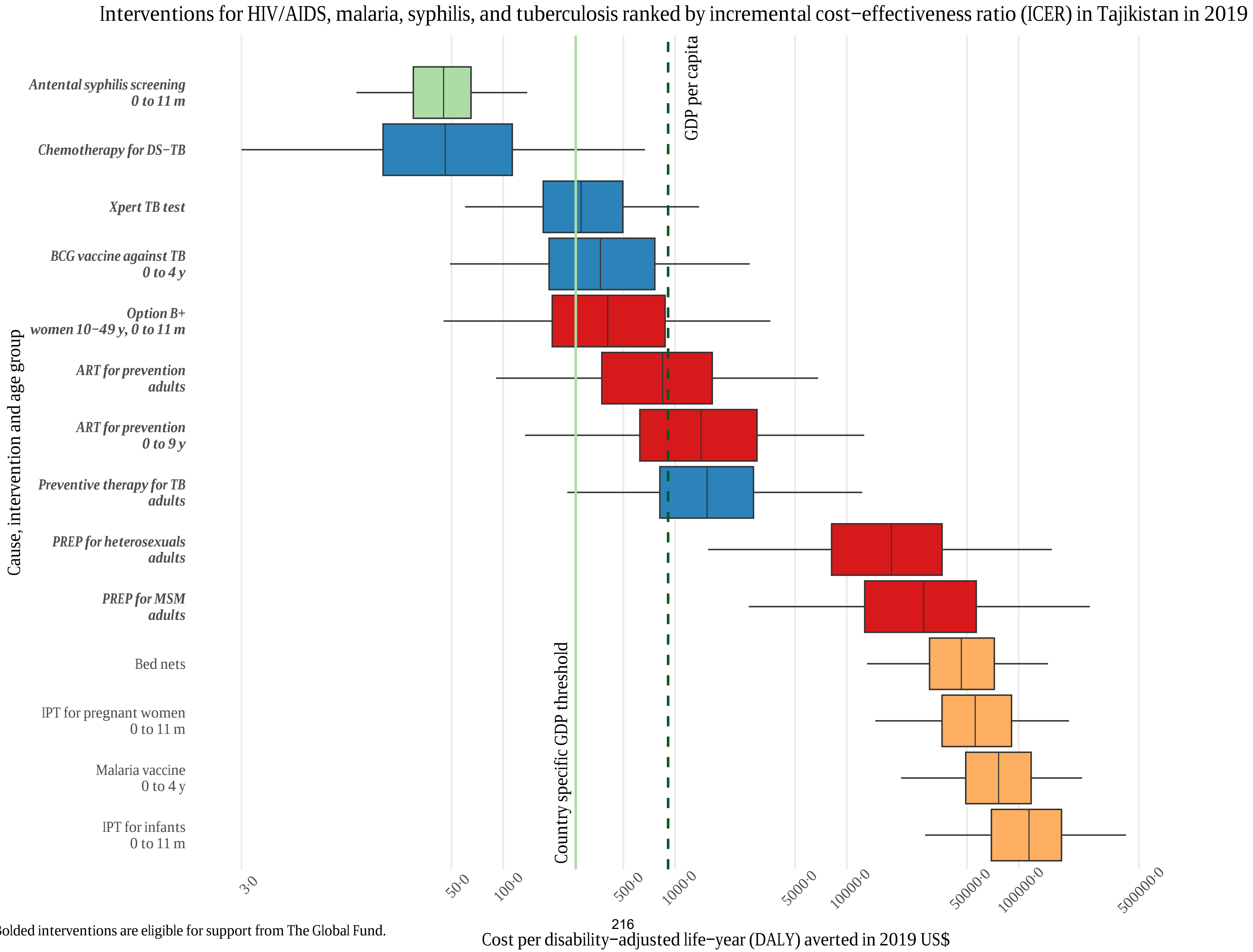

Bolded interventions are eligible for support from The Global Fund.

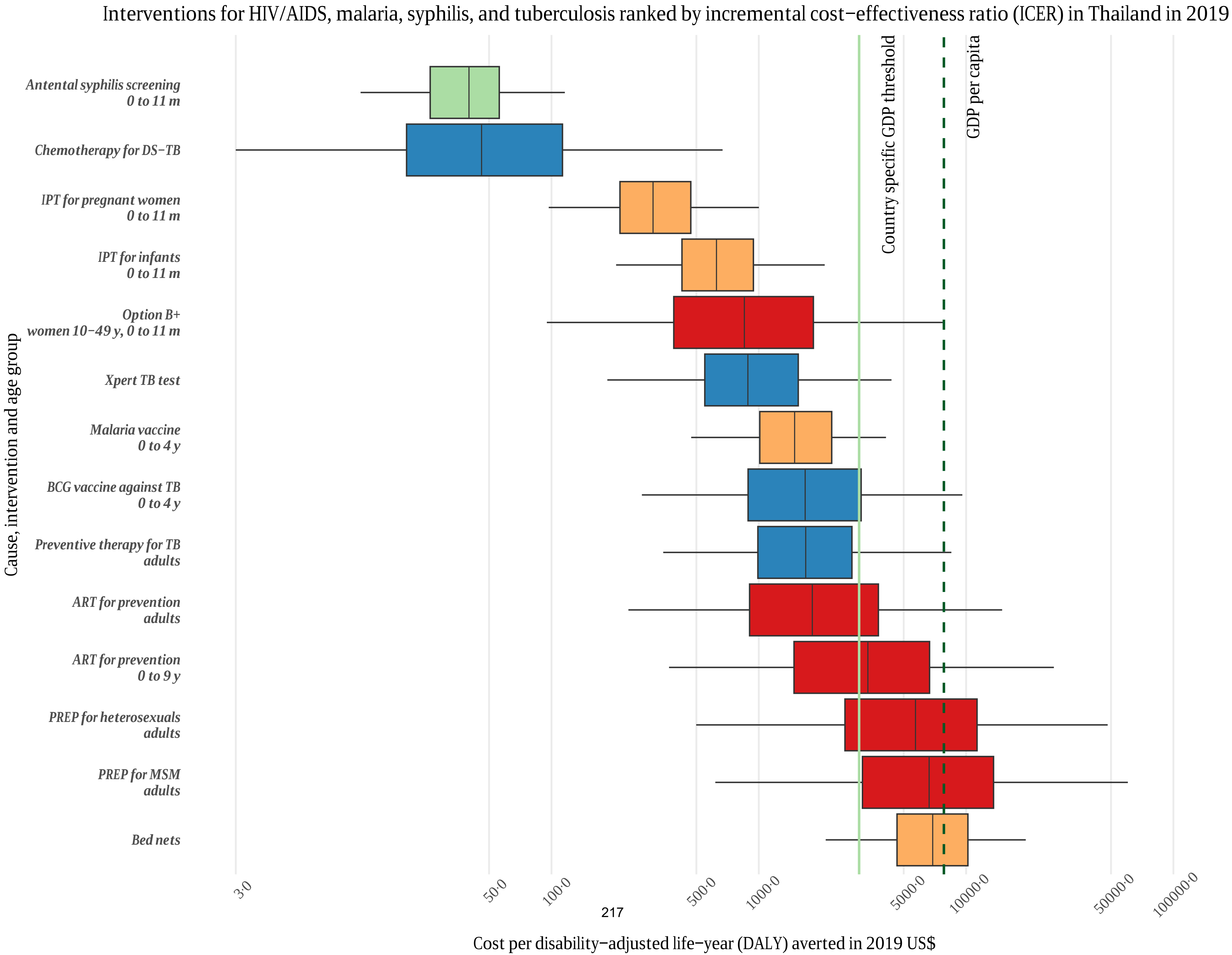

Interventions for HIV/AIDS, malaria, syphilis, and tuberculosis ranked by incremental cost−effectiveness ratio (ICER) in Thailand in 2019
Country specific GDP threshold
GDP per capita
Antental syphilis screening
0 to 11 m
Chemotherapy for DS−TB
IPT for pregnant women
0 to 11 m
IPT for infants
0 to 11 m
Option B+
women 10−49 y, 0 to 11 m
Xpert TB test
Malaria vaccine
0 to 4 y
BCG vaccine against TB
0 to 4 y
Preventive therapy for TB
adults
ART for prevention
adults
ART for prevention
0 to 9 y
PREP for heterosexuals
adults
PREP for MSM
adults
Bed nets
Cause, intervention and age group
3·0
50·0
100·0
500·0
1000·0
5000·0
10000·0
50000·0
100000·0
Cost per disability−adjusted life−year (DALY) averted in 2019 US$

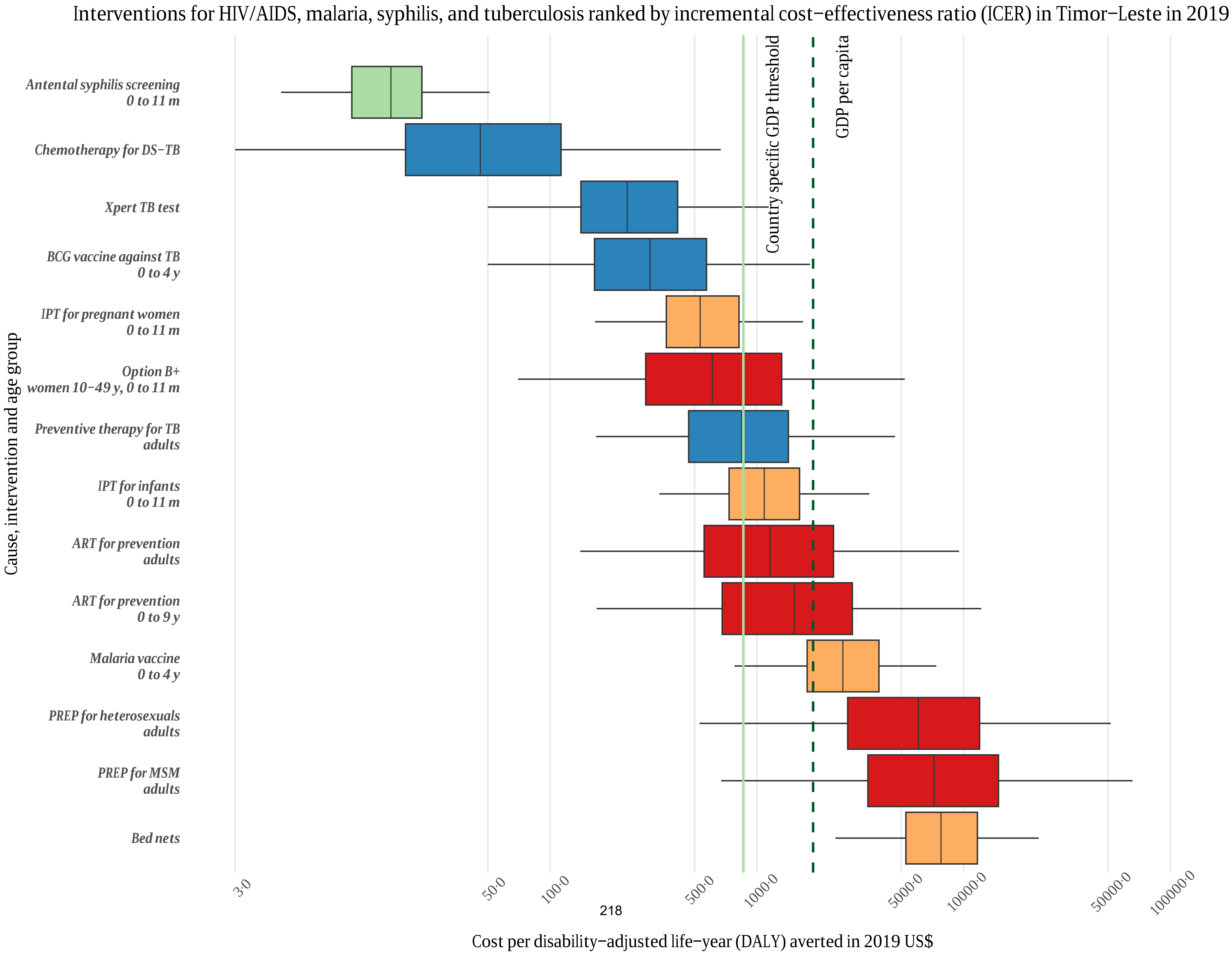

Interventions for HIV/AIDS, malaria, syphilis, and tuberculosis ranked by incremental cost−effectiveness ratio (ICER) in Timor−Leste in 2019
Country specific GDP threshold
GDP per capita
Antental syphilis screening
0 to 11 m
Chemotherapy for DS−TB
Xpert TB test
BCG vaccine against TB
0 to 4 y
IPT for pregnant women
0 to 11 m
Option B+
women 10−49 y, 0 to 11 m
Preventive therapy for TB
adults
IPT for infants
0 to 11 m
ART for prevention
adults
ART for prevention
0 to 9 y
Malaria vaccine
0 to 4 y
PREP for heterosexuals
adults
PREP for MSM
adults
Bed nets
Cause, intervention and age group
3·0
50·0
100·0
500·0
1000·0
5000·0
10000·0
50000·0
100000·0
Cost per disability−adjusted life−year (DALY) averted in 2019 US$

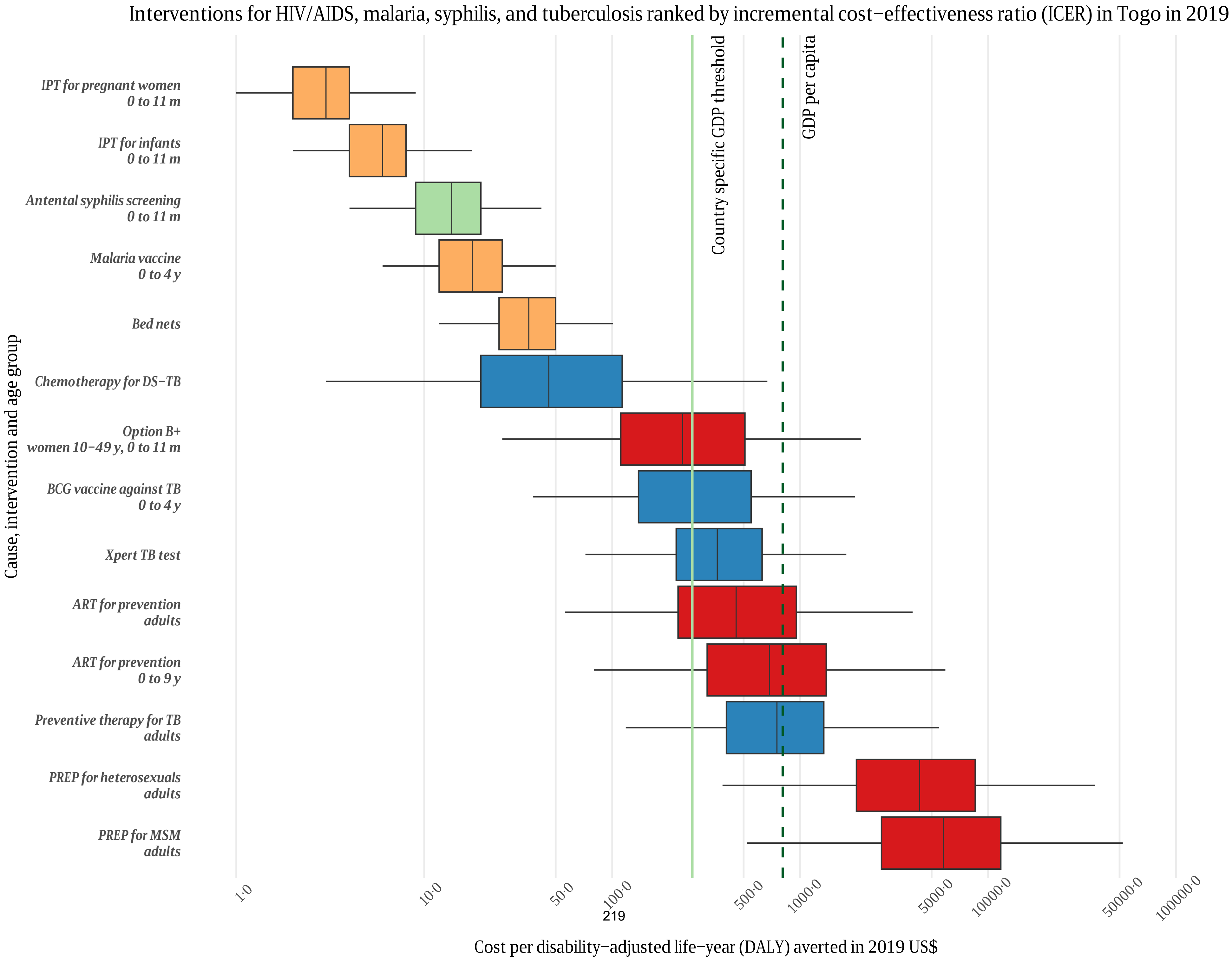

Interventions for HIV/AIDS, malaria, syphilis, and tuberculosis ranked by incremental cost−effectiveness ratio (ICER) in Togo in 2019
Cause, intervention and age group
IPT for pregnant women 0 to 11 m
IPT for infants 0 to 11 m
Antental syphilis screening 0 to 11 m
Malaria vaccine 0 to 4 y
Bed nets
Chemotherapy for DS−TB
Option B+ women 10−49 y, 0 to 11 m
BCG vaccine against TB 0 to 4 y
Xpert TB test
ART for prevention adults
ART for prevention 0 to 9 y
Preventive therapy for TB adults
PREP for heterosexuals adults
PREP for MSM adults
Country specific GDP threshold
GDP per capita
1·0
10·0
50·0
100·0
500·0
1000·0
5000·0
10000·0
50000·0
100000·0
Cost per disability−adjusted life−year (DALY) averted in 2019 US$

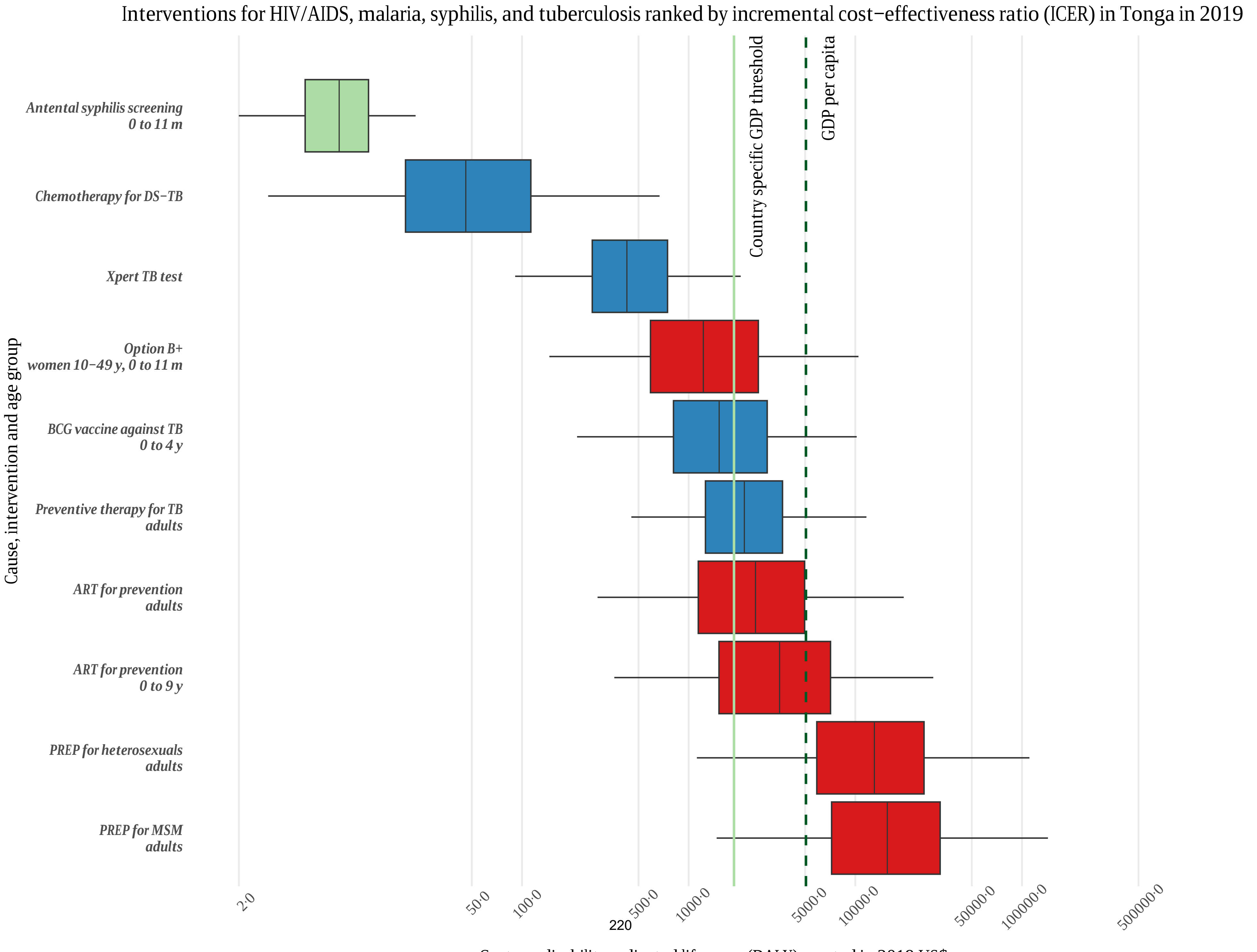
Interventions for HIV/AIDS, malaria, syphilis, and tuberculosis ranked by incremental cost−effectiveness ratio (ICER) in Tonga in 2019
Cause, intervention and age group
Antental syphilis screening
0 to 11 m
Chemotherapy for DS−TB
Xpert TB test
Option B+
women 10−49 y, 0 to 11 m
BCG vaccine against TB
0 to 4 y
Preventive therapy for TB
adults
ART for prevention
adults
ART for prevention
0 to 9 y
PREP for heterosexuals
adults
PREP for MSM
adults
Country specific GDP threshold
GDP per capita
2·0
50·0
100·0
500·0
1000·0
5000·0
10000·0
50000·0
100000·0
500000·0
Cost per disability−adjusted life−year (DALY) averted in 2019 US$

Interventions for HIV/AIDS, malaria, syphilis, and tuberculosis ranked by incremental cost−effectiveness ratio (ICER) in Tunisia in 2019

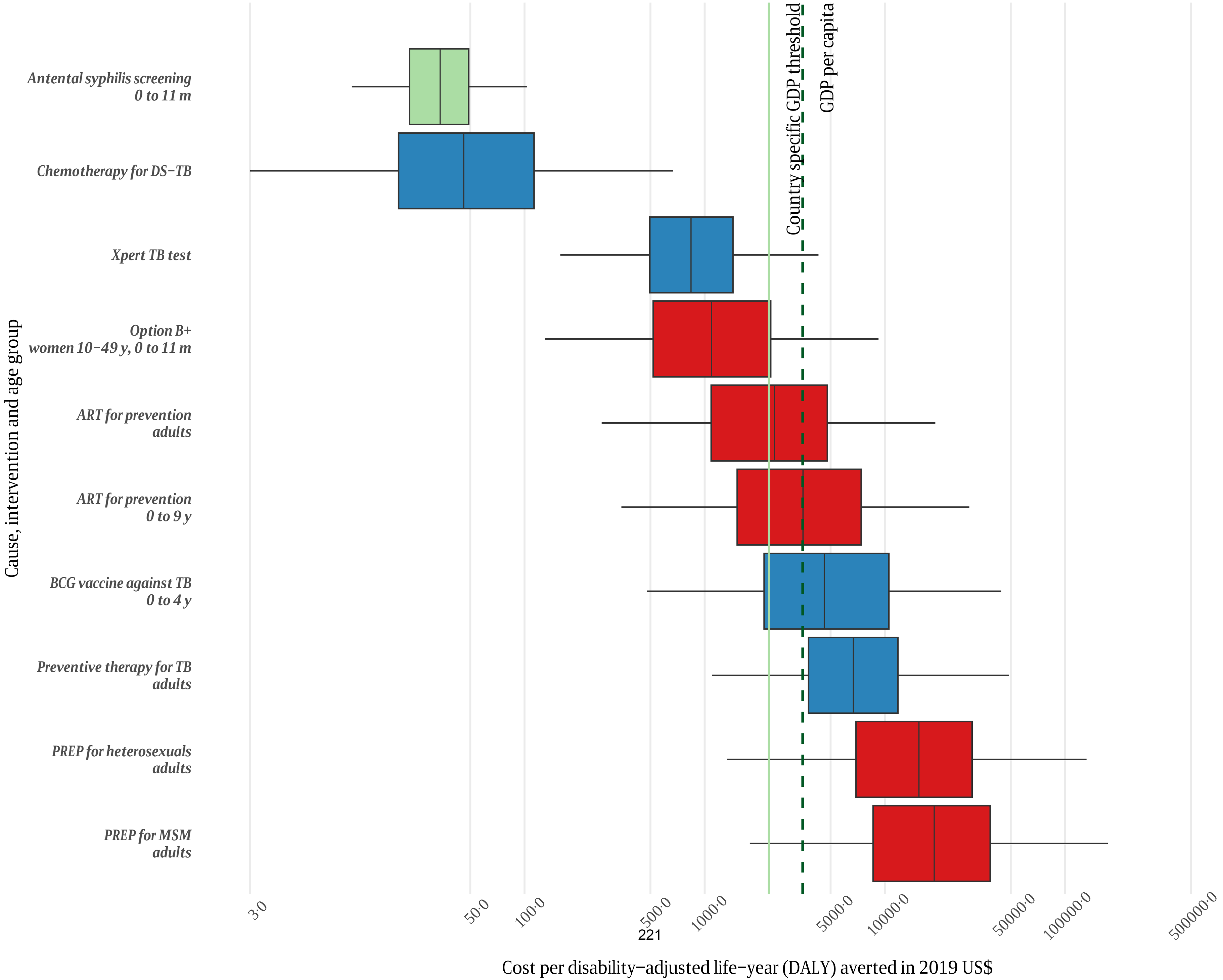

221

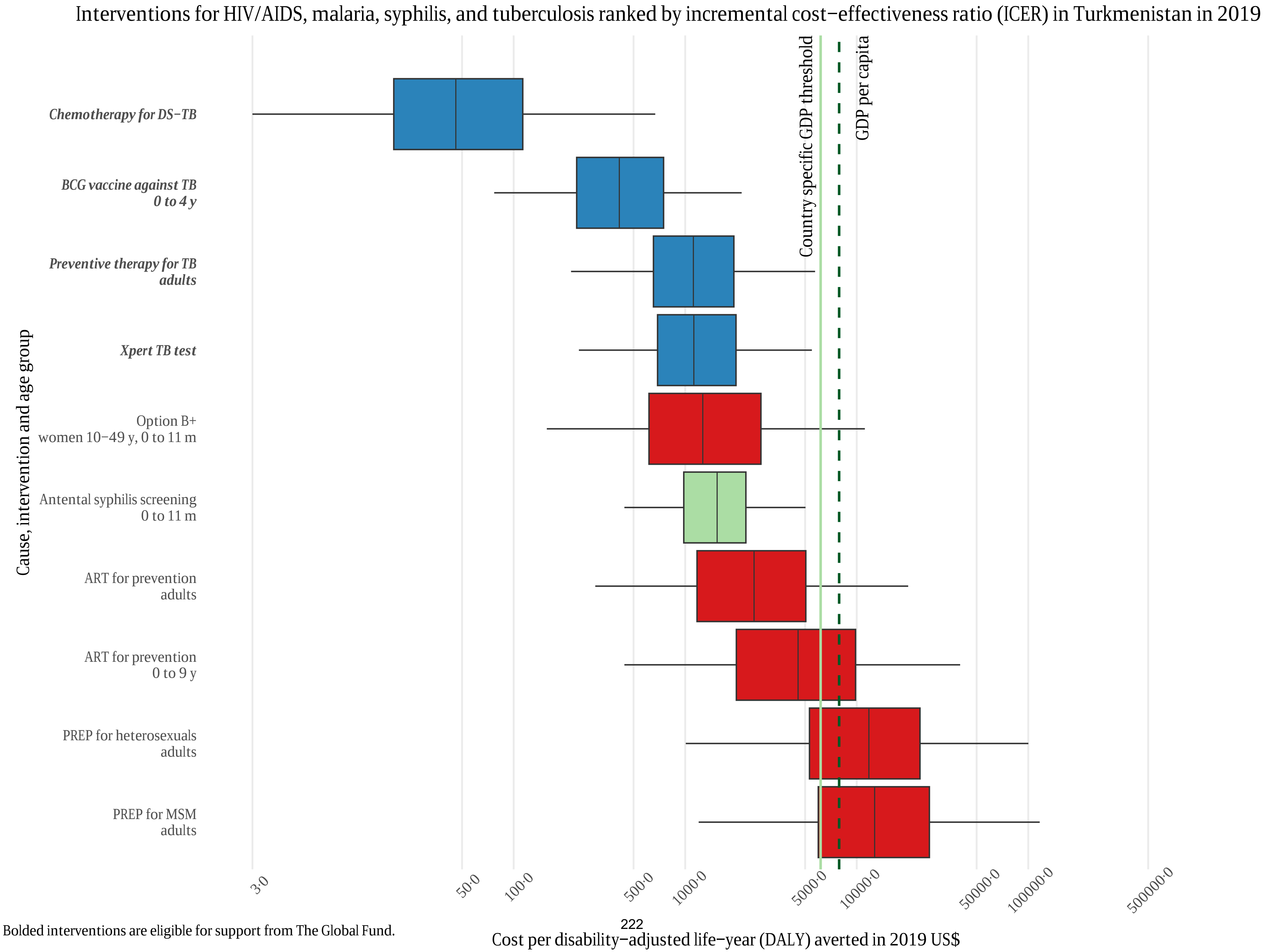
Interventions for HIV/AIDS, malaria, syphilis, and tuberculosis ranked by incremental cost−effectiveness ratio (ICER) in Turkmenistan in 2019
Cause, intervention and age group
Chemotherapy for DS−TB
BCG vaccine against TB
0 to 4 y
Preventive therapy for TB
adults
Xpert TB test
Option B+
women 10−49 y, 0 to 11 m
Antental syphilis screening
0 to 11 m
ART for prevention
adults
ART for prevention
0 to 9 y
PREP for heterosexuals
adults
PREP for MSM
adults
Country specific GDP threshold
GDP per capita
3·0
50·0
100·0
500·0
1000·0
5000·0
10000·0
50000·0
100000·0
500000·0
Cost per disability−adjusted life−year (DALY) averted in 2019 US$
Bolded interventions are eligible for support from The Global Fund.

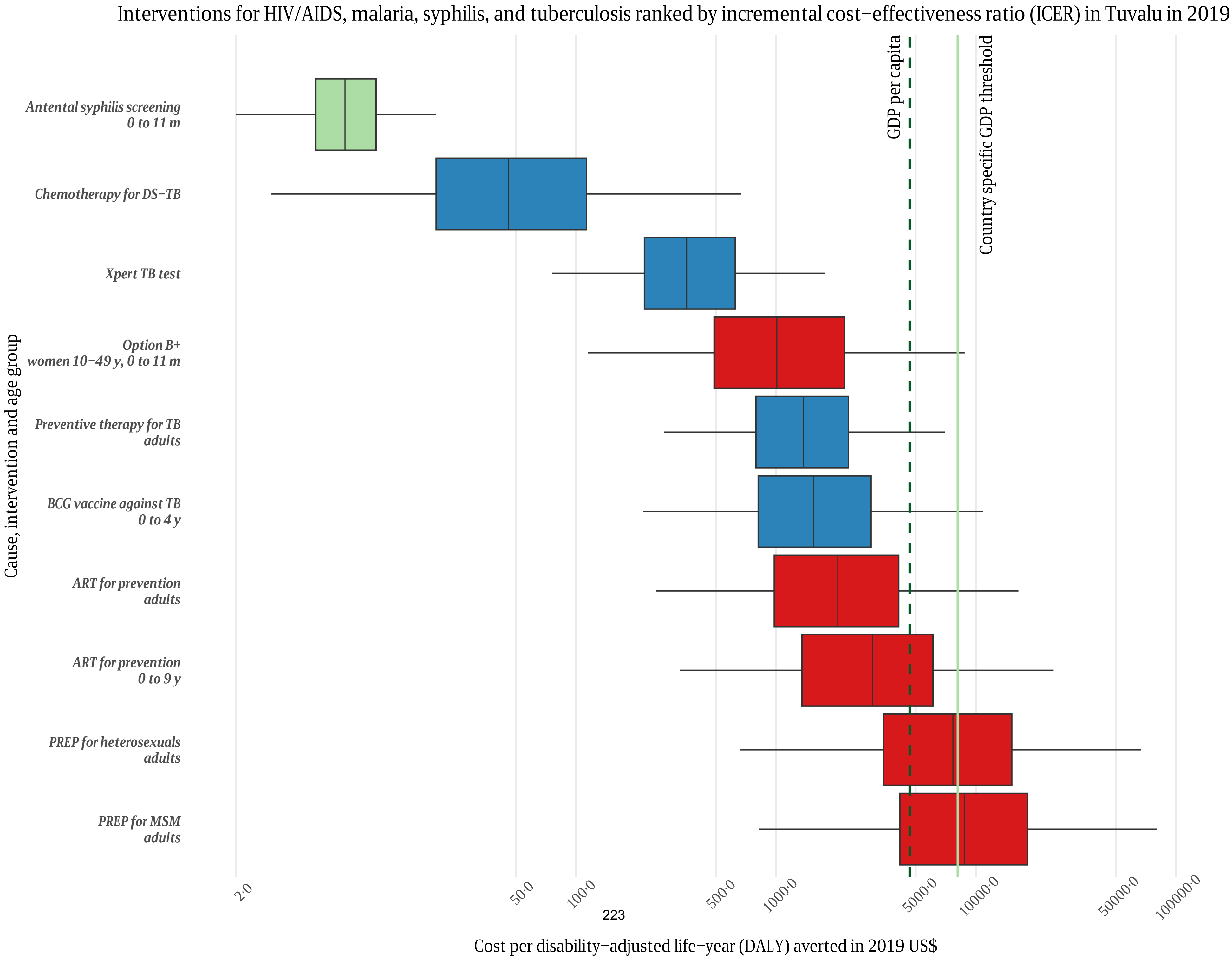

Interventions for HIV/AIDS, malaria, syphilis, and tuberculosis ranked by incremental cost−effectiveness ratio (ICER) in Tuvalu in 2019
GDP per capita
Country specific GDP threshold
Cause, intervention and age group
Antental syphilis screening
0 to 11 m
Chemotherapy for DS−TB
Xpert TB test
Option B+
women 10−49 y, 0 to 11 m
Preventive therapy for TB
adults
BCG vaccine against TB
0 to 4 y
ART for prevention
adults
ART for prevention
0 to 9 y
PREP for heterosexuals
adults
PREP for MSM
adults
2·0
50·0
100·0
500·0
1000·0
5000·0
10000·0
50000·0
100000·0
Cost per disability−adjusted life−year (DALY) averted in 2019 US$

Interventions for HIV/AIDS, malaria, syphilis, and tuberculosis ranked by incremental cost−effectiveness ratio (ICER) in Uganda in 2019

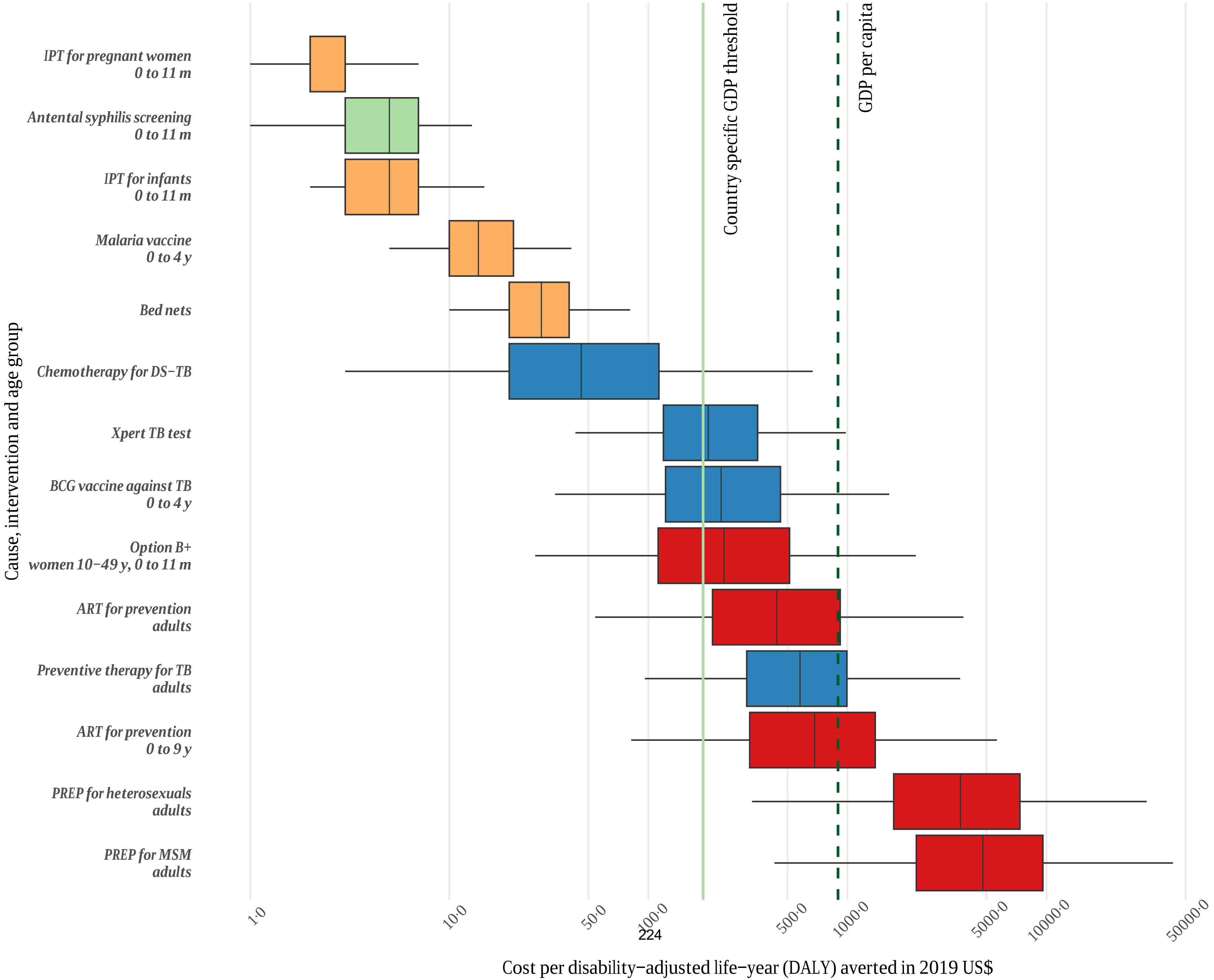

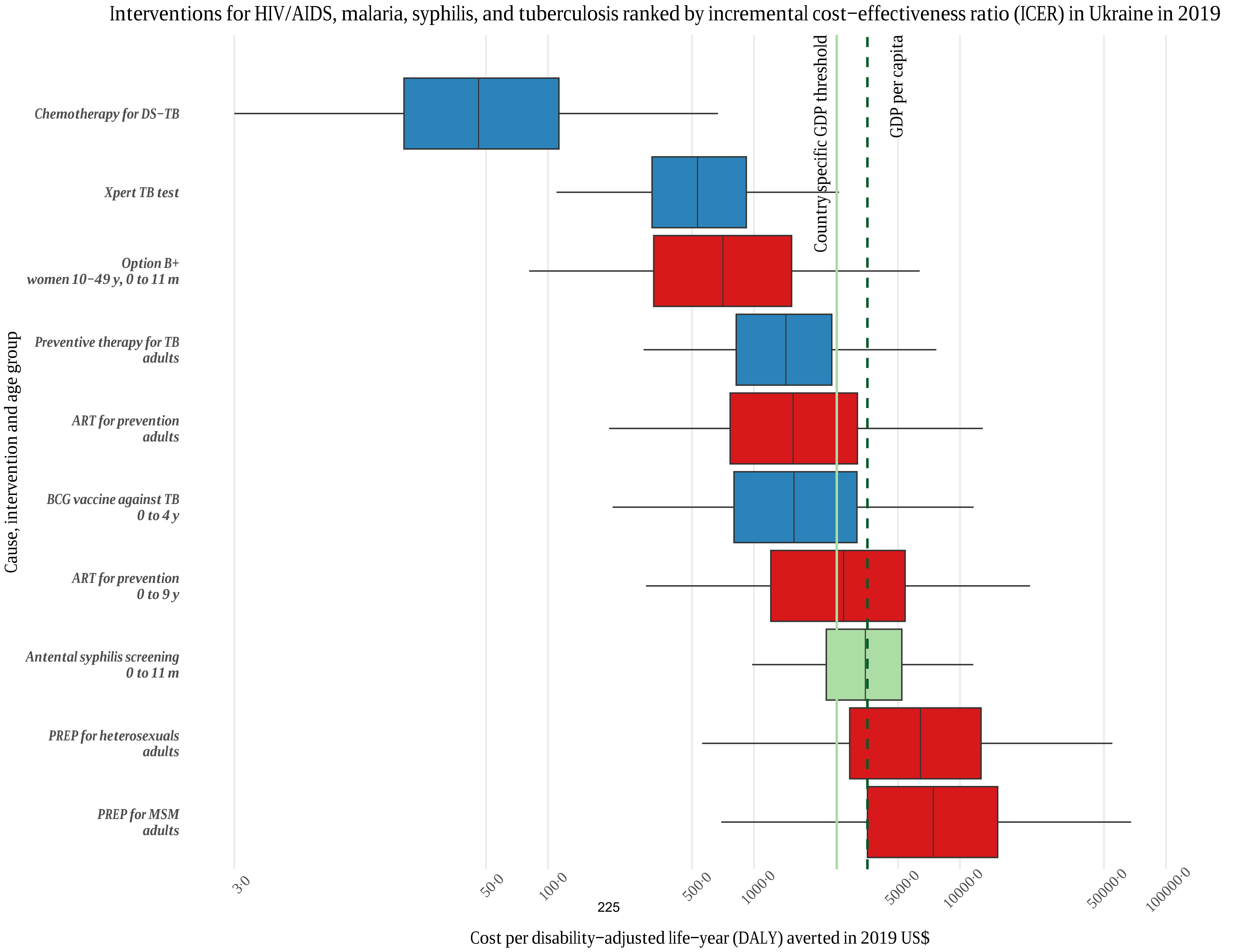

Interventions for HIV/AIDS, malaria, syphilis, and tuberculosis ranked by incremental cost−effectiveness ratio (ICER) in Ukraine in 2019
Country specific GDP threshold
GDP per capita
Chemotherapy for DS−TB
Xpert TB test
Option B+
women 10−49 y, 0 to 11 m
Preventive therapy for TB
adults
ART for prevention
adults
BCG vaccine against TB
0 to 4 y
ART for prevention
0 to 9 y
Antental syphilis screening
0 to 11 m
PREP for heterosexuals
adults
PREP for MSM
adults
Cause, intervention and age group
3·0
50·0
100·0
500·0
1000·0
5000·0
10000·0
50000·0
100000·0
Cost per disability−adjusted life−year (DALY) averted in 2019 US$

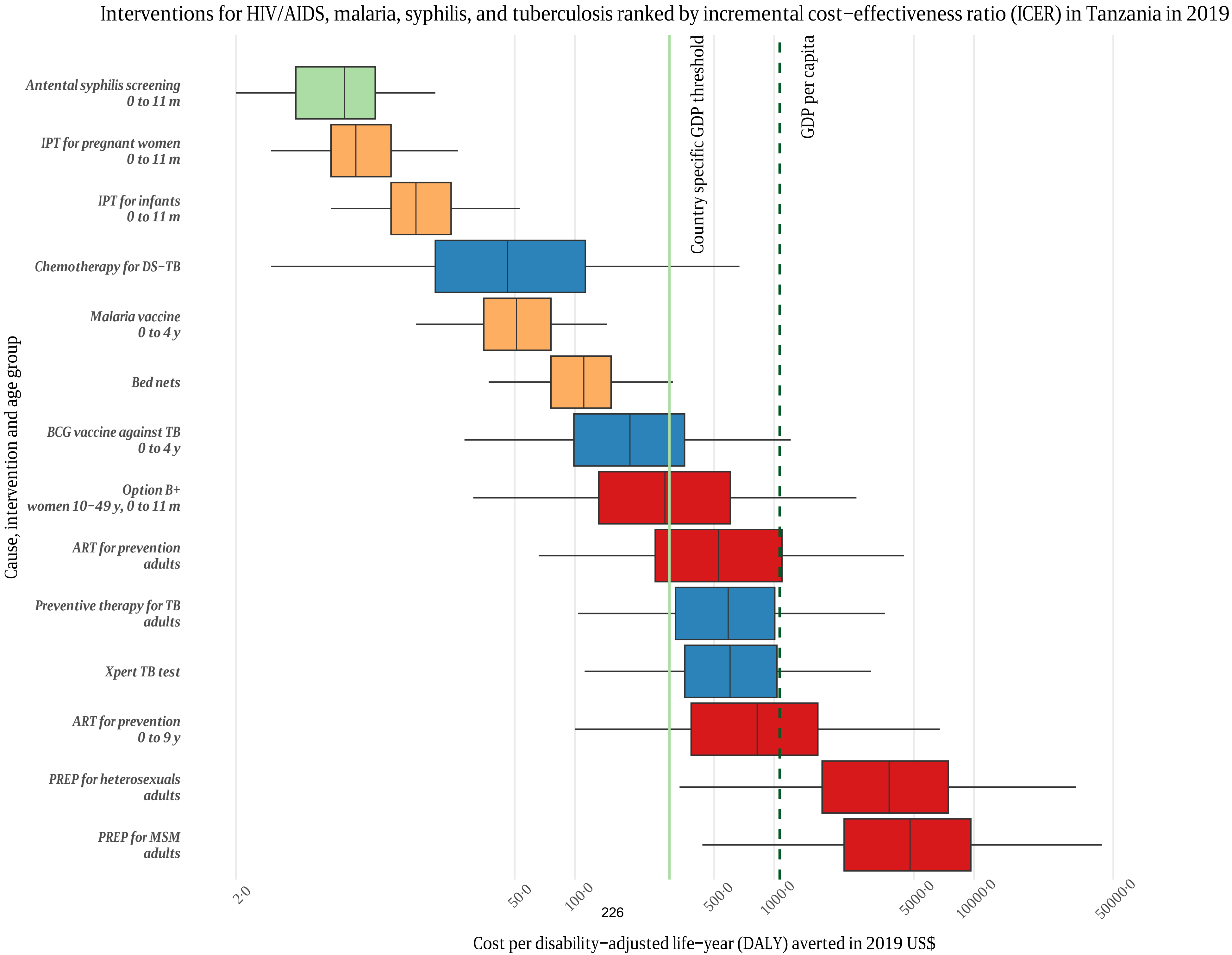
Interventions for HIV/AIDS, malaria, syphilis, and tuberculosis ranked by incremental cost−effectiveness ratio (ICER) in Tanzania in 2019
Antental syphilis screening
0 to 11 m
IPT for pregnant women
0 to 11 m
IPT for infants
0 to 11 m
Chemotherapy for DS−TB
Malaria vaccine
0 to 4 y
Bed nets
BCG vaccine against TB
0 to 4 y
Option B+
women 10−49 y, 0 to 11 m
ART for prevention
adults
Preventive therapy for TB
adults
Xpert TB test
ART for prevention
0 to 9 y
PREP for heterosexuals
adults
PREP for MSM
adults
Country specific GDP threshold
GDP per capita
Cause, intervention and age group
2·0
50·0
100·0
500·0
1000·0
5000·0
10000·0
50000·0
Cost per disability−adjusted life−year (DALY) averted in 2019 US$

Interventions for HIV/AIDS, malaria, syphilis, and tuberculosis ranked by incremental cost−effectiveness ratio (ICER) in Uzbekistan in 2019

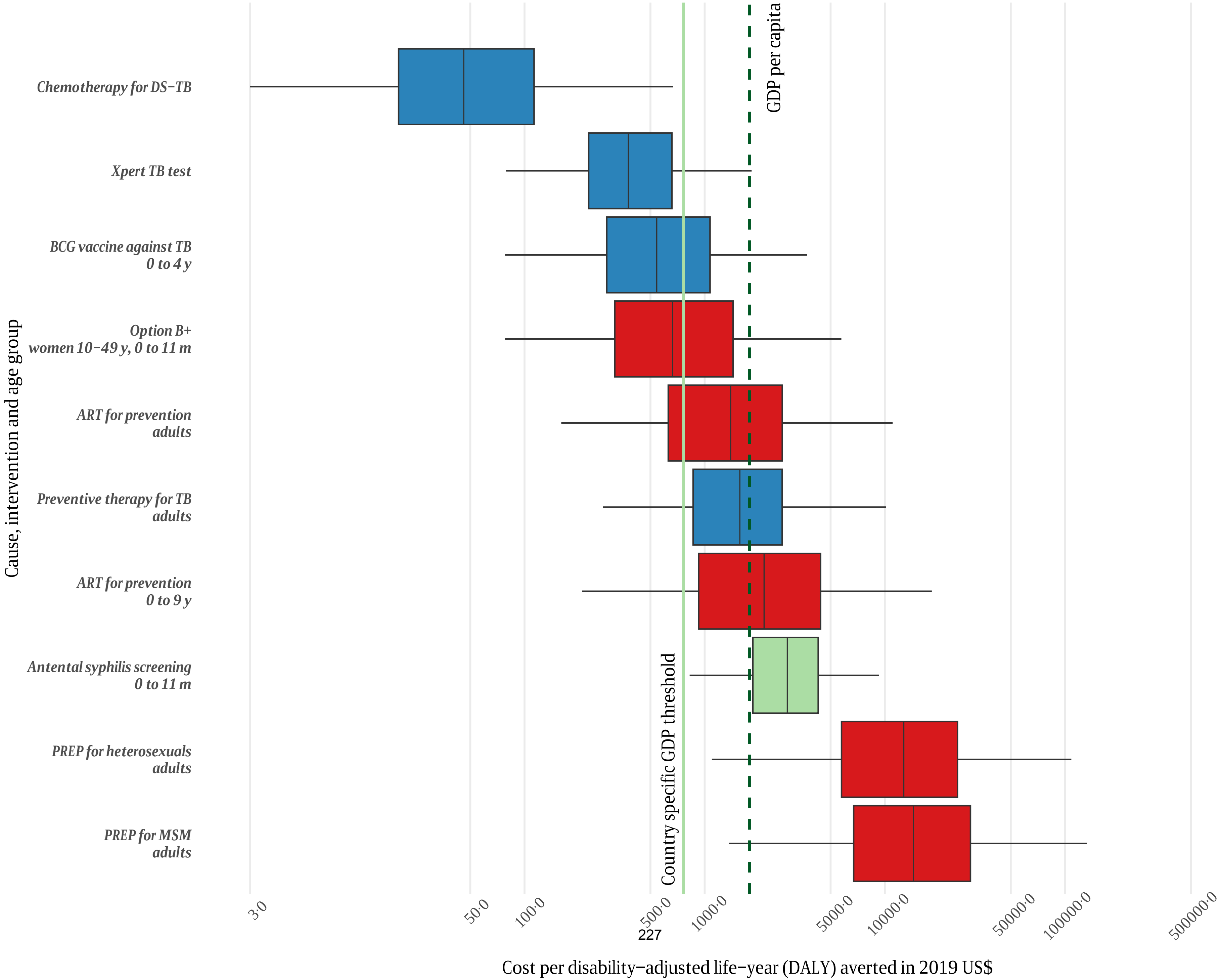

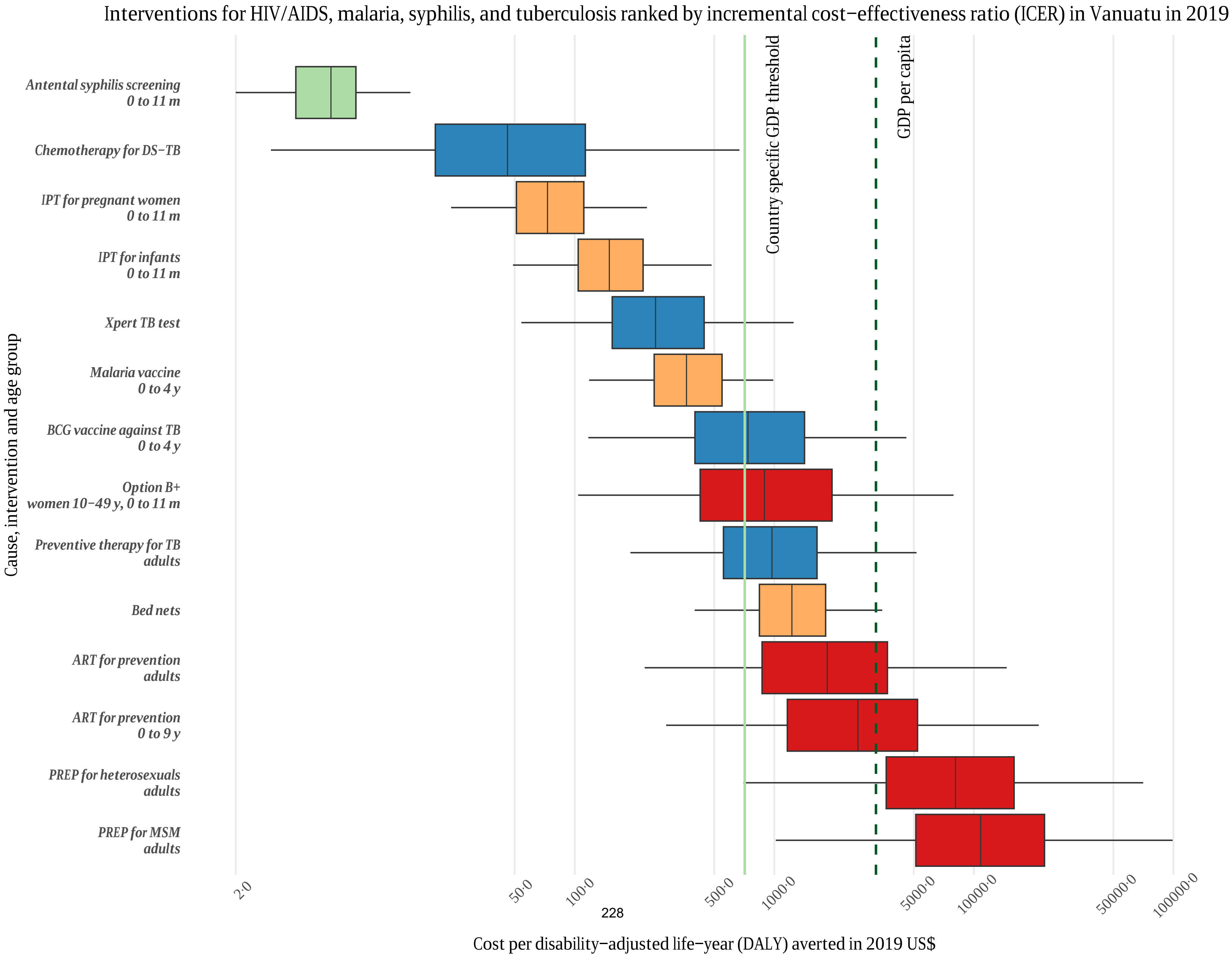

Interventions for HIV/AIDS, malaria, syphilis, and tuberculosis ranked by incremental cost−effectiveness ratio (ICER) in Vanuatu in 2019
Cause, intervention and age group
Antental syphilis screening 0 to 11 m
Chemotherapy for DS−TB
IPT for pregnant women 0 to 11 m
IPT for infants 0 to 11 m
Xpert TB test
Malaria vaccine 0 to 4 y
BCG vaccine against TB 0 to 4 y
Option B+ women 10−49 y, 0 to 11 m
Preventive therapy for TB adults
Bed nets
ART for prevention adults
ART for prevention 0 to 9 y
PREP for heterosexuals adults
PREP for MSM adults
Country specific GDP threshold
GDP per capita
2·0
50·0
100·0
500·0
1000·0
5000·0
10000·0
50000·0
100000·0
Cost per disability−adjusted life−year (DALY) averted in 2019 US$

Interventions for HIV/AIDS, malaria, syphilis, and tuberculosis ranked by incremental cost−effectiveness ratio (ICER) in Venezuela in 2019

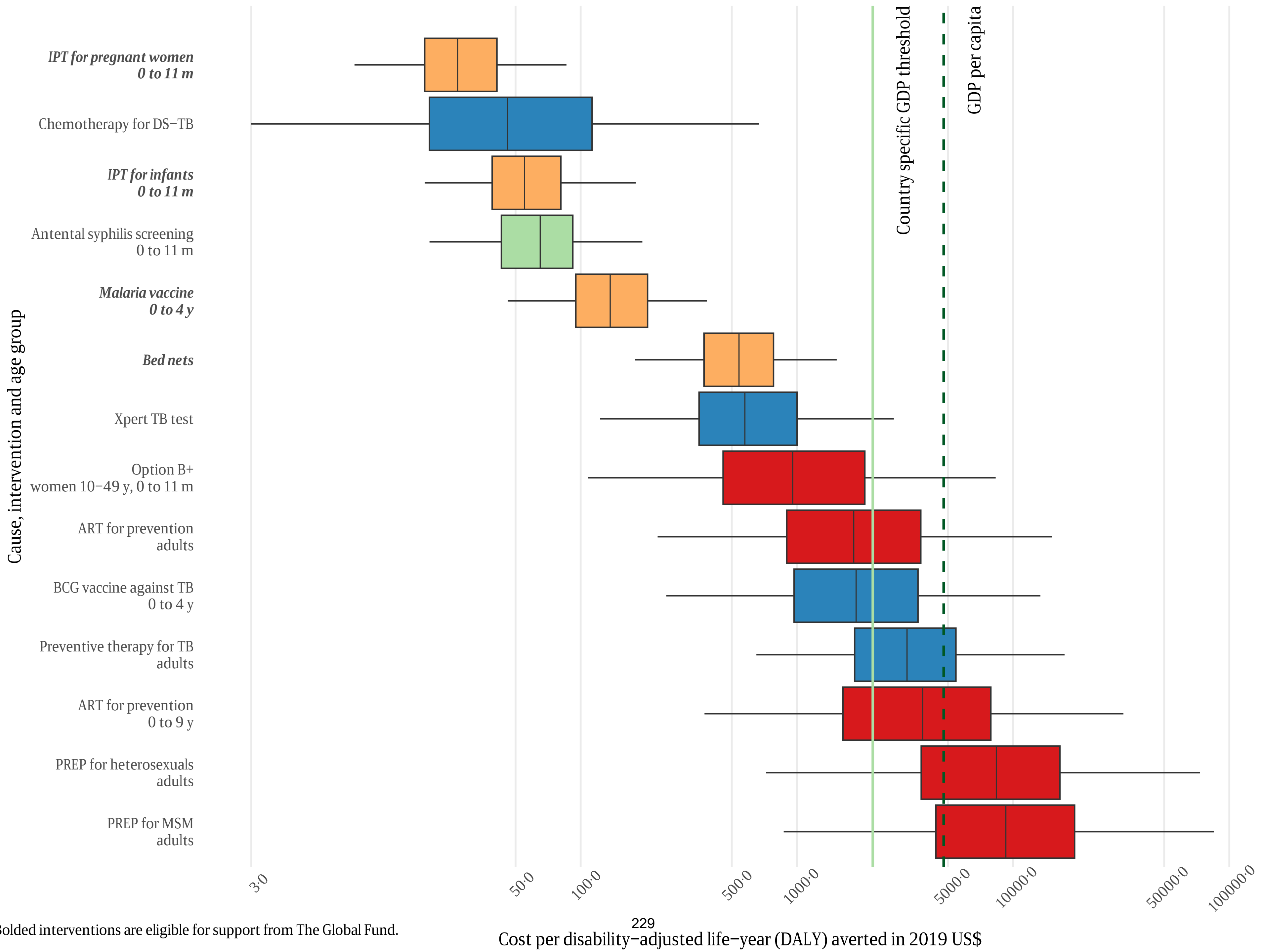

Bolded interventions are eligible for support from The Global Fund.

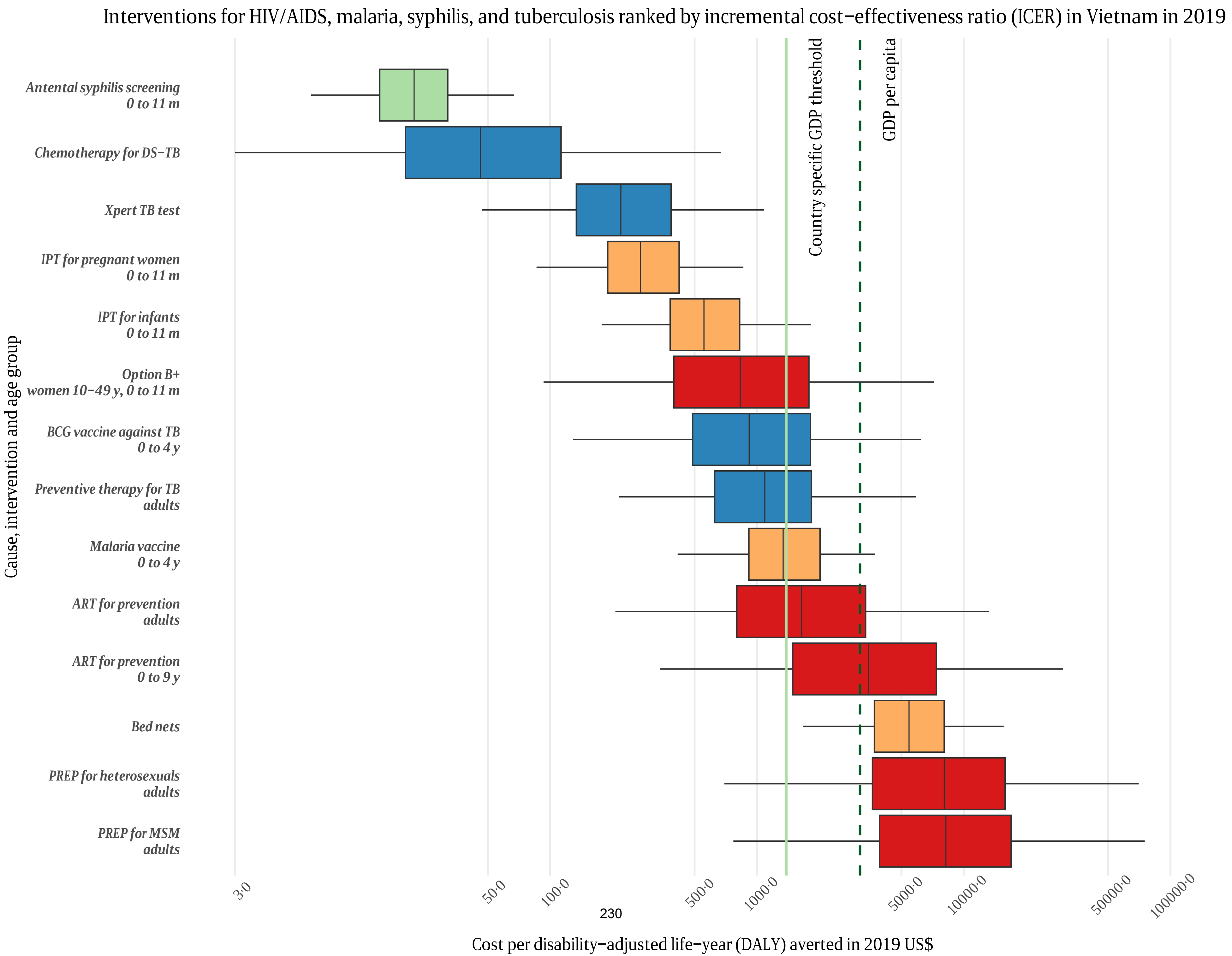

Interventions for HIV/AIDS, malaria, syphilis, and tuberculosis ranked by incremental cost−effectiveness ratio (ICER) in Vietnam in 2019
Country specific GDP threshold
GDP per capita
Antental syphilis screening 0 to 11 m
Chemotherapy for DS−TB
Xpert TB test
IPT for pregnant women 0 to 11 m
IPT for infants 0 to 11 m
Option B+ women 10−49 y, 0 to 11 m
BCG vaccine against TB 0 to 4 y
Preventive therapy for TB adults
Malaria vaccine 0 to 4 y
ART for prevention adults
ART for prevention 0 to 9 y
Bed nets
PREP for heterosexuals adults
PREP for MSM adults
Cause, intervention and age group
3·0
50·0
100·0
500·0
1000·0
5000·0
10000·0
50000·0
100000·0
Cost per disability−adjusted life−year (DALY) averted in 2019 US$

Interventions for HIV/AIDS, malaria, syphilis, and tuberculosis ranked by incremental cost−effectiveness ratio (ICER) in Yemen in 2019

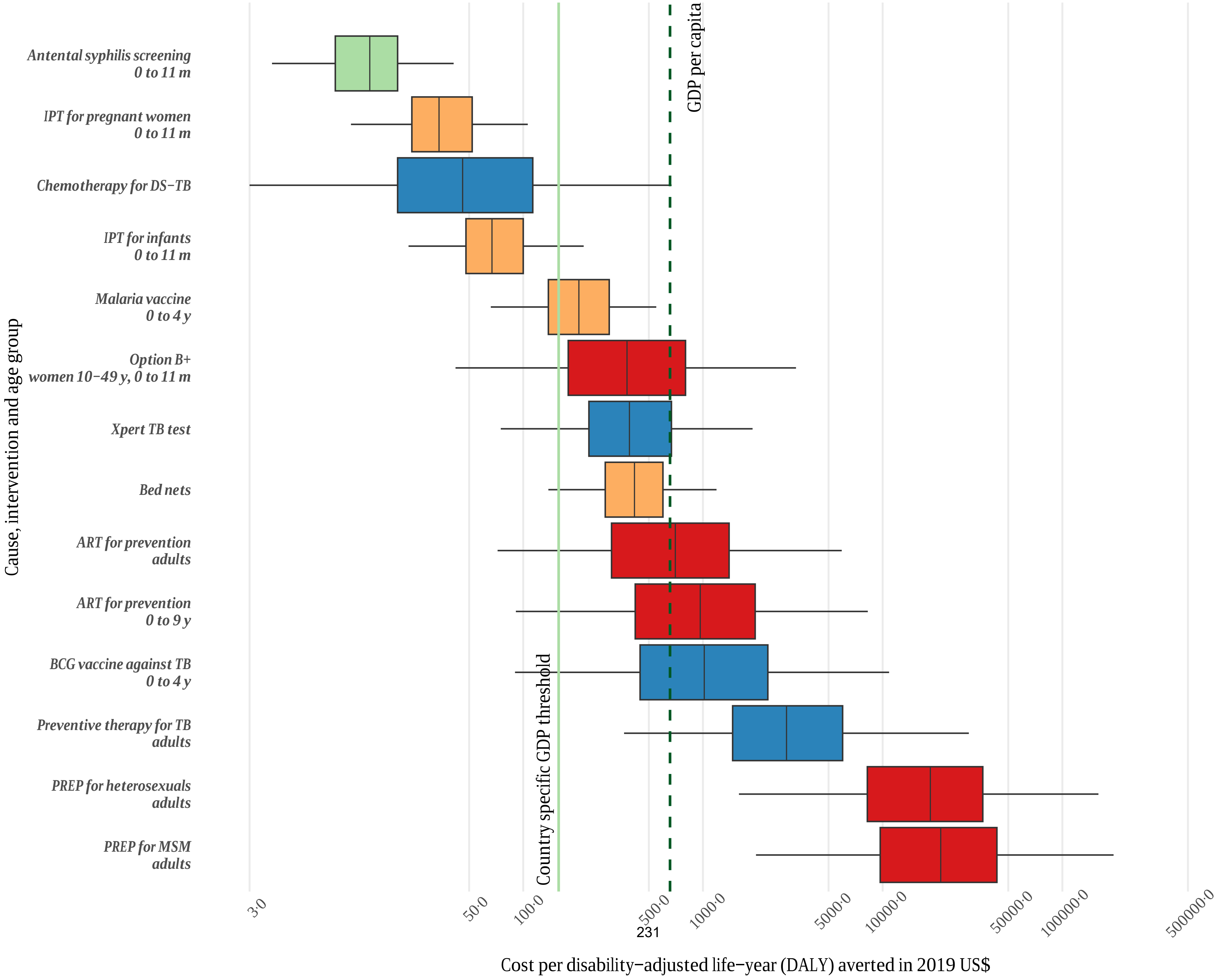

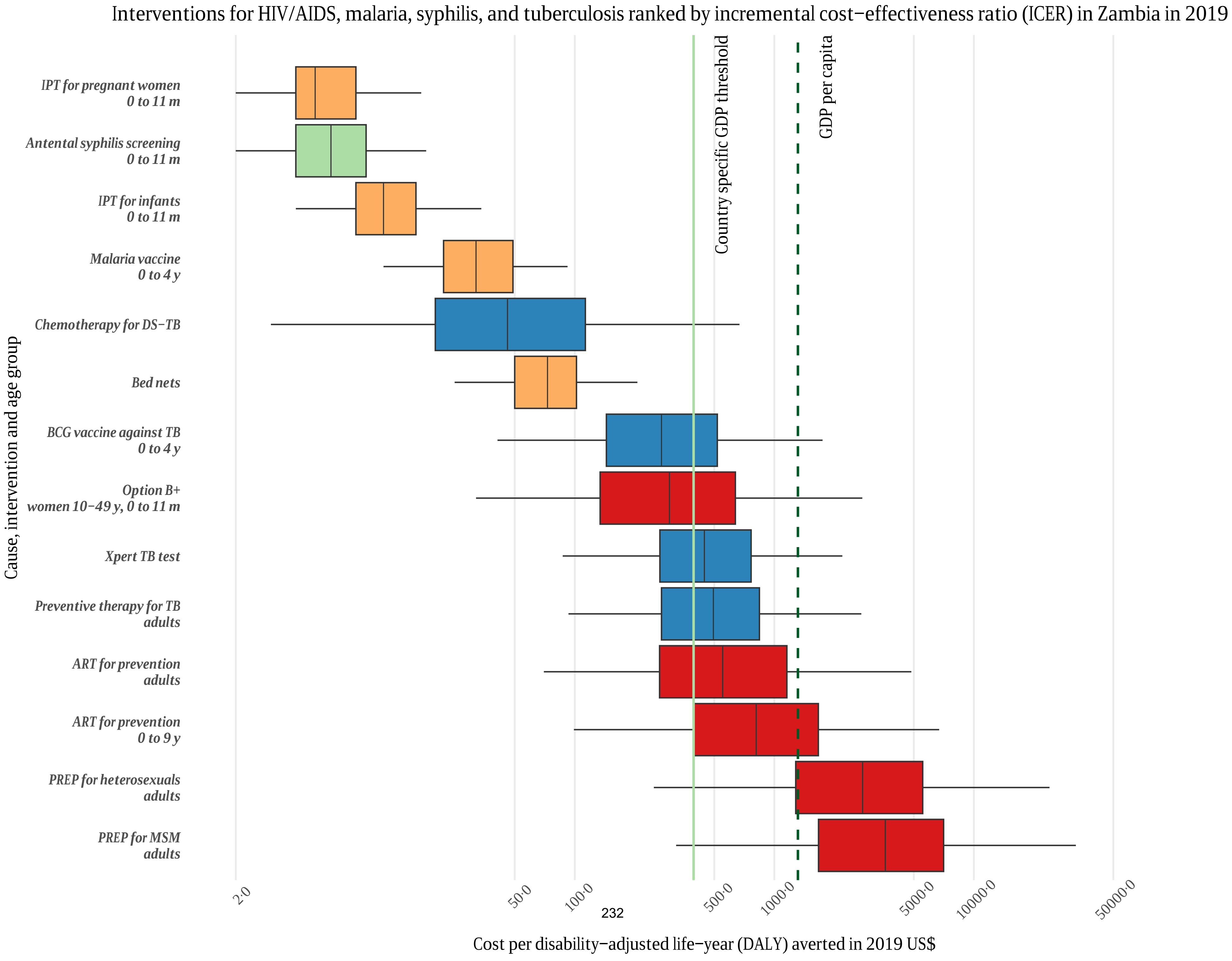
Interventions for HIV/AIDS, malaria, syphilis, and tuberculosis ranked by incremental cost−effectiveness ratio (ICER) in Zambia in 2019
Country specific GDP threshold
GDP per capita
IPT for pregnant women
0 to 11 m
Antental syphilis screening
0 to 11 m
IPT for infants
0 to 11 m
Malaria vaccine
0 to 4 y
Chemotherapy for DS−TB
Bed nets
BCG vaccine against TB
0 to 4 y
Option B+
women 10−49 y, 0 to 11 m
Xpert TB test
Preventive therapy for TB
adults
ART for prevention
adults
ART for prevention
0 to 9 y
PREP for heterosexuals
adults
PREP for MSM
adults
Cause, intervention and age group
2·0
50·0
100·0
500·0
1000·0
5000·0
10000·0
50000·0
Cost per disability−adjusted life−year (DALY) averted in 2019 US$

Interventions for HIV/AIDS, malaria, syphilis, and tuberculosis ranked by incremental cost−effectiveness ratio (ICER) in Zimbabwe in 2019

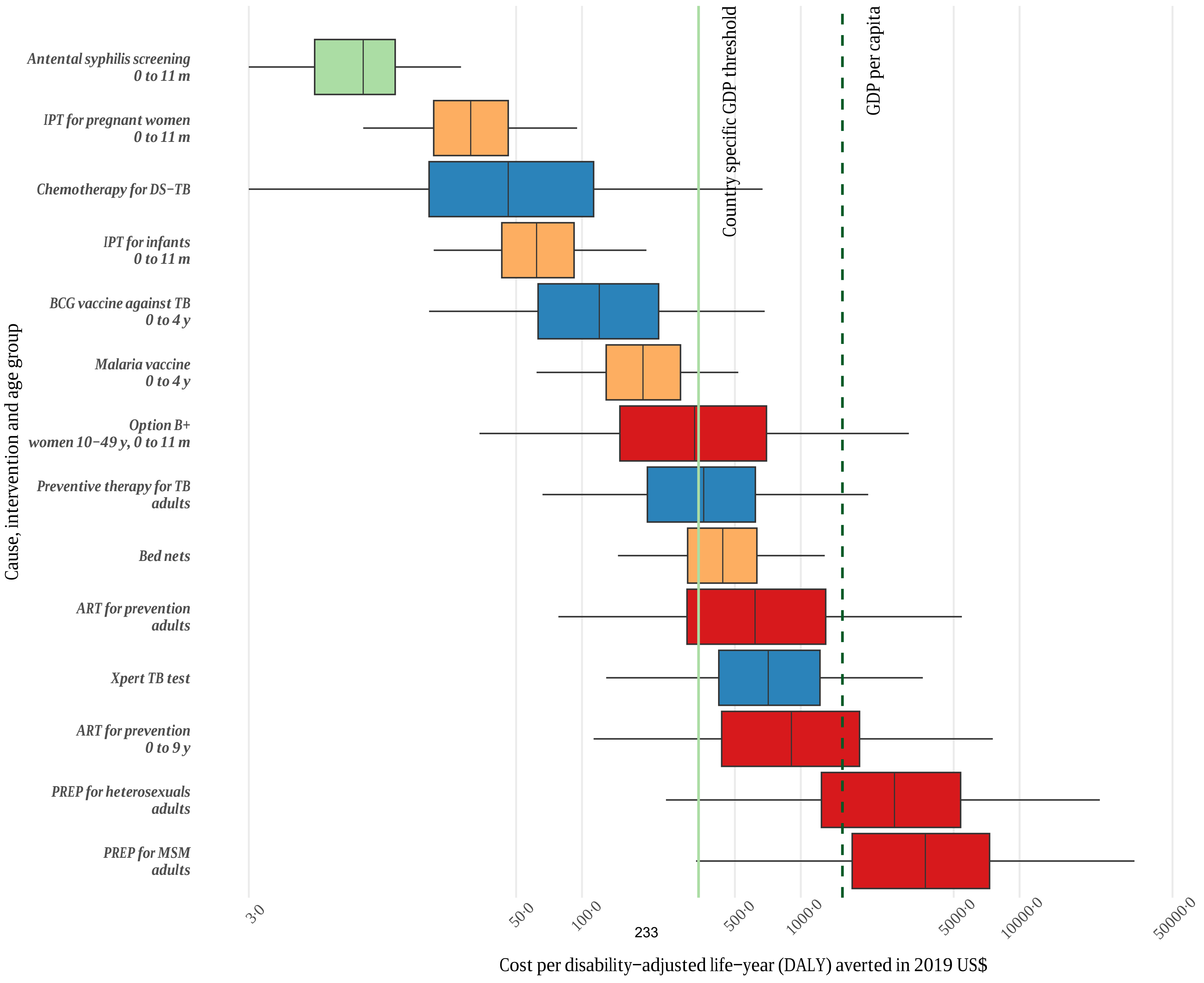

Supplement: Supplementary appendix [file mmc1.pdf]
